# Supplementary material for: A Critical View of the Application of the APEX Software (Aqueous Photochemistry of Environmentally-Occurring Xenobiotics) to Predict Photoreaction Kinetics in Surface Freshwaters
Source: Molecules. 2019 Dec 18;25(1):9. doi: 10.3390/molecules25010009 (PMC7017383; doi:10.3390/molecules25010009)
Supplement: Supplementary file 1 [file molecules-25-00009-s001.zip › 3.2.4_gcc-4.4.0.zip!/3.2.4_gcc-4.4.0/3.2.4_gcc-4.4.0/doc/gnuplot/gnuplot.pdf]

gnuplot 4.4

An Interactive Plotting Program

Thomas Williams & Colin Kelley

Version 4.4 organized by: Hans-Bernhard Bröker, Ethan A Merritt, and others

Major contributors (alphabetic order):
Hans-Bernhard Bröker
John Campbell
Robert Cunningham
David Denholm
Gershon Elber
Roger Fearick
Carsten Grammes
Lucas Hart
Lars Hecking
Thomas Koenig
David Kotz
Ed Kubaitis
Russell Lang
Timothée Lecomte
Alexander Lehmann
Alexander Mai
Ethan A Merritt
Petr Mikulík
Carsten Steger
Tom Tkacik
Jos Van der Woude
Alex Woo
James R. Van Zandt
Johannes Zellner
Copyright © 1986 - 1993, 1998, 2004 Thomas Williams, Colin Kelley
Copyright © 2004 - 2009 various authors

Mailing list for comments: `gnuplot-info@lists.sourceforge.net`
Mailing list for bug reports: `gnuplot-bugs@lists.sourceforge.net`
Web access (preferred): `http://sourceforge.net/projects/gnuplot`

This manual was originally prepared by Dick Crawford.

05 Mar 2010

Contents

I Gnuplot 14

1 Copyright 14

2 Introduction 14

3 Seeking-assistance 15

4 New features introduced in version 4.4 16

4.1 Internationalization . . . 16
4.2 Transparency . . . 16
4.3 Volatile Data . . . 16
4.4 Canvas size . . . 16
4.5 New plot elements . . . 17
4.6 New or revised terminal drivers . . . 17
4.7 New smoothing algorithms . . . 17

5 Backwards compatibility 17

6 Batch/Interactive Operation 18

7 Command-line-editing 18

8 Comments 19

9 Coordinates 19

10 Datastrings 19

11 Enhanced text mode 20

12 Environment 21

13 Expressions 21

13.1 Functions . . . 22
13.1.1 Elliptic integrals . . . 24
13.1.2 Random number generator . . . 24
13.2 Operators . . . 24
13.2.1 Unary . . . 24
13.2.2 Binary . . . 25
13.2.3 Ternary . . . 25
13.3 Gnuplot-defined variables . . . 26
13.4 User-defined variables and functions . . . 26

14 Fonts 27

14.1 Cairo (pdfcairo, pngcairo, wxt terminals) . . . 27
14.2 Gd (png, gif, jpeg terminals) . . . 27
14.3 Postscript (also encapsulated postscript *.eps) . . . 28

15 Glossary **28**

16 Linetype, colors, and styles **29**
16.1 Colorspec . . . 29
16.1.1 Rgbcolor variable . . . 30
16.1.2 Linecolor variable . . . 30

17 Mouse input **31**
17.1 Bind . . . 31
17.1.1 Bind space . . . 32
17.2 Mouse variables . . . 32

18 Plotting **33**

19 Start-up **33**

20 String constants and string variables **33**

21 Substitution and Command line macros **34**
21.1 Substitution of system commands in backquotes . . . 34
21.2 Substitution of string variables as macros . . . 34
21.3 String variables, macros, and command line substitution . . . 35

22 Syntax **35**
22.1 Quote Marks . . . 36

23 Time/Date data **36**

II Plotting styles **37**

24 Boxerrorbars **37**

25 Boxes **38**

26 Boxxyerrorbars **39**

27 Candlesticks **39**

28 Circles **40**

29 Dots **40**

30 Filledcurves **40**

31 Financebars **41**

32 Fsteps **41**

33 Histeps **41**

34 Histograms **42**
34.1 Newhistogram . . . 44

34.2 Automated iteration over multiple columns 44

35 Image **44**

35.1 Transparency 45

35.2 Image failsafe 45

36 Impulses **45**

37 Labels **46**

38 Lines **46**

39 Linespoints **47**

40 Points **47**

41 Steps **47**

42 Rgbalpha **47**

43 Rgbimage **47**

44 Vectors **47**

45 Xerrorbars **48**

46 Xyerrorbars **48**

47 Yerrorbars **48**

48 Xerrorlines **49**

49 Xyerrorlines **49**

50 Yerrorlines **49**

51 3D (surface) plots **50**

III Commands **50**

52 Cd **51**

53 Call **51**

54 Clear **51**

55 Evaluate **52**

56 Exit **52**

57 Fit **52**

57.1 Adjustable parameters 54

57.2 Short introduction 54

57.3 Error estimates . . . 55
57.3.1 Statistical overview . . . 55
57.3.2 Practical guidelines . . . 56
57.4 Control . . . 56
57.4.1 Control variables . . . 56
57.4.2 Environment variables . . . 57
57.5 Multi-branch . . . 57
57.6 Starting values . . . 57
57.7 Tips . . . 58

58 Help **58**

59 History **59**

60 If **59**

61 Iteration **59**

62 Load **60**

63 Lower **60**

64 Pause **60**

65 Plot **61**
65.1 Axes . . . 62
65.2 Data . . . 62
65.2.1 Binary . . . 63
65.2.2 Binary general . . . 64
65.2.2.1 Array . . . 64
65.2.2.2 Record . . . 64
65.2.2.3 Skip . . . 64
65.2.2.4 Format . . . 65
65.2.2.5 Endian . . . 65
65.2.2.6 Filetype . . . 65
65.2.2.6.1 Avs . . . 65
65.2.2.6.2 Edf . . . 65
65.2.2.6.3 Png . . . 65
65.2.2.7 Keywords . . . 65
65.2.2.7.1 Scan . . . 66
65.2.2.7.2 Transpose . . . 66
65.2.2.7.3 Dx, dy, dz . . . 66
65.2.2.7.4 Flipx, flipy, flipz . . . 66
65.2.2.7.5 Origin . . . 66
65.2.2.7.6 Center . . . 66
65.2.2.7.7 Rotate . . . 66
65.2.2.7.8 Perpendicular . . . 66
65.2.2.8 Binary examples . . . 67

65.2.3 Every . . . 67
65.2.4 Example datafile . . . 68
65.2.5 Index . . . 68
65.2.6 Smooth . . . 69
65.2.6.1 Acsplines . . . 69
65.2.6.2 Bezier . . . 69
65.2.6.3 Csplines . . . 69
65.2.6.4 Sbezier . . . 69
65.2.6.5 Unique . . . 69
65.2.6.6 Frequency . . . 69
65.2.6.7 Cumulative . . . 70
65.2.6.8 Kdensity . . . 70
65.2.7 Special-filenames . . . 70
65.2.8 Thru . . . 71
65.2.9 Using . . . 71
65.2.9.1 Using_examples . . . 72
65.2.9.2 Pseudocolumns . . . 73
65.2.9.3 Xticlabels . . . 73
65.2.9.4 X2ticlabels . . . 73
65.2.9.5 Yticlabels . . . 73
65.2.9.6 Y2ticlabels . . . 73
65.2.9.7 Zticlabels . . . 73
65.3 Errorbars . . . 73
65.4 Errorlines . . . 74
65.5 Parametric . . . 74
65.6 Ranges . . . 75
65.7 Iteration . . . 76
65.8 Title . . . 76
65.9 With . . . 77

66 Print **79**

67 Pwd **79**

68 Quit **79**

69 Raise **80**

70 Refresh **80**

71 Replot **80**

72 Reread **81**

73 Reset **81**

74 Save **81**

75 Set-show **82**

75.1 Angles . . . 82
75.2 Arrow . . . 83
75.3 Autoscale . . . 84
75.3.1 Parametric mode . . . 85
75.3.2 Polar mode . . . 86
75.4 Bars . . . 86
75.5 Bind . . . 86
75.6 Bmargin . . . 86
75.7 Border . . . 86
75.8 Boxwidth . . . 87
75.9 Clabel . . . 88
75.10 Clip . . . 88
75.11 Cntrparam . . . 89
75.12 Color box . . . 90
75.13 Colornames . . . 91
75.14 Contour . . . 91
75.15 Data style . . . 92
75.16 Datafile . . . 92
75.16.1 Set datafile fortran . . . 92
75.16.2 Set datafile nofpe_trap . . . 92
75.16.3 Set datafile missing . . . 92
75.16.4 Set datafile separator . . . 93
75.16.5 Set datafile commentschars . . . 93
75.16.6 Set datafile binary . . . 94
75.17 Decimalsign . . . 94
75.18 Dgrid3d . . . 95
75.19 Dummy . . . 96
75.20 Encoding . . . 96
75.21 Fit . . . 97
75.22 Fontpath . . . 97
75.23 Format . . . 97
75.23.1 Gprintf . . . 98
75.23.2 Format specifiers . . . 98
75.23.3 Time/date specifiers . . . 99
75.24 Function style . . . 100
75.25 Functions . . . 100
75.26 Grid . . . 100
75.27 Hidden3d . . . 101
75.28 Historysize . . . 102
75.29 Isosamples . . . 102
75.30 Key . . . 103
75.30.1 Key placement . . . 104
75.30.2 Key samples . . . 105
75.31 Label . . . 105
75.32 Lmargin . . . 107

75.33 Loadpath . . . 107
75.34 Locale . . . 108
75.35 Logscale . . . 108
75.36 Macros . . . 108
75.37 Mapping . . . 108
75.38 Margin . . . 109
75.39 Mouse . . . 109
75.39.1 X11 mouse . . . 111
75.40 Multiplot . . . 111
75.41 Mx2tics . . . 112
75.42 Mxtics . . . 112
75.43 My2tics . . . 113
75.44 Mytics . . . 113
75.45 Mztics . . . 113
75.46 Object . . . 113
75.46.1 Rectangle . . . 113
75.46.2 Ellipse . . . 114
75.46.3 Circle . . . 114
75.46.4 Polygon . . . 114
75.47 Offsets . . . 115
75.48 Origin . . . 115
75.49 Output . . . 115
75.50 Parametric . . . 116
75.51 Plot . . . 116
75.52 Pm3d . . . 116
75.52.1 Depthorder . . . 119
75.53 Palette . . . 119
75.53.1 Rgbformulae . . . 121
75.53.2 Defined . . . 121
75.53.3 Functions . . . 122
75.53.4 File . . . 122
75.53.5 Gamma correction . . . 123
75.53.6 Postscript . . . 123
75.54 Pointsize . . . 124
75.55 Polar . . . 124
75.56 Print . . . 124
75.57 Rmargin . . . 125
75.58 Rrange . . . 125
75.59 Samples . . . 125
75.60 Size . . . 125
75.61 Style . . . 126
75.61.1 Set style arrow . . . 126
75.61.2 Set style data . . . 127
75.61.3 Set style fill . . . 127
75.61.3.1 Set style fill transparent . . . 128

75.61.4 Set style function . . . 128
75.61.5 Set style increment . . . 128
75.61.6 Set style line . . . 129
75.61.7 Set style rectangle . . . 130
75.62 Surface . . . 130
75.63 Table . . . 130
75.64 Terminal . . . 131
75.65 Termoption . . . 131
75.66 Tics . . . 131
75.67 Ticslevel . . . 132
75.68 Ticscale . . . 132
75.69 Timestamp . . . 132
75.70 Timefmt . . . 132
75.71 Title . . . 133
75.72 Tmargin . . . 134
75.73 Trange . . . 134
75.74 Urange . . . 134
75.75 Variables . . . 134
75.76 Version . . . 134
75.77 View . . . 134
75.77.1 Equal_axes . . . 135
75.78 Vrange . . . 135
75.79 X2data . . . 135
75.80 X2dtics . . . 135
75.81 X2label . . . 135
75.82 X2mtics . . . 135
75.83 X2range . . . 135
75.84 X2tics . . . 136
75.85 X2zeroaxis . . . 136
75.86 Xdata . . . 136
75.87 Xdtics . . . 136
75.88 Xlabel . . . 136
75.89 Xmtics . . . 137
75.90 Xrange . . . 138
75.91 Xtics . . . 139
75.91.1 Xtics time_data . . . 140
75.91.2 Xtics rangelimited . . . 141
75.92 Xyplane . . . 141
75.93 Xzeroaxis . . . 141
75.94 Y2data . . . 141
75.95 Y2dtics . . . 142
75.96 Y2label . . . 142
75.97 Y2mtics . . . 142
75.98 Y2range . . . 142
75.99 Y2tics . . . 142

75.100Y2zeroaxis . . . 142
75.101Ydata . . . 142
75.102Ydtics . . . 142
75.103Ylabel . . . 142
75.104Ymtics . . . 142
75.105Yrange . . . 142
75.106Ytics . . . 142
75.107Yzeroaxis . . . 143
75.108Zdata . . . 143
75.109Zdtics . . . 143
75.110Zzeroaxis . . . 143
75.111Cbdata . . . 143
75.112Cbdtics . . . 143
75.113Zero . . . 143
75.114Zeroaxis . . . 143
75.115Zlabel . . . 144
75.116Zmtics . . . 144
75.117Zrange . . . 144
75.118Ztics . . . 144
75.119Cblabel . . . 144
75.120Cbmtics . . . 144
75.121Cbrange . . . 144
75.122Cbtics . . . 144

76 Shell **144**

77 Splot **145**
77.1 Data-file . . . 145
77.1.1 Binary matrix . . . 146
77.1.2 Example datafile . . . 147
77.1.3 Matrix_ascii . . . 147
77.1.4 Matrix . . . 148
77.2 Grid data . . . 148
77.3 Splot overview . . . 148

78 System **149**

79 Test **149**

80 Undefine **149**

81 Unset **149**

82 Update **150**

IV Terminal types **150**

83 Complete list of terminals 150
83.1 Aed767 . . . 150
83.2 Aifm . . . 150
83.3 Amiga . . . 151
83.4 Apollo . . . 151
83.5 Aqua . . . 151
83.6 Be . . . 151
83.6.1 Command-line_options . . . 152
83.6.2 Monochrome_options . . . 152
83.6.3 Color_resources . . . 152
83.6.4 Grayscale_resources . . . 153
83.6.5 Line_resources . . . 153
83.7 Canvas . . . 153
83.8 Cgi . . . 154
83.9 Cgm . . . 154
83.9.1 Cgm font . . . 155
83.9.2 Cgm fontsize . . . 156
83.9.3 Cgm linewidth . . . 156
83.9.4 Cgm rotate . . . 156
83.9.5 Cgm solid . . . 156
83.9.6 Cgm size . . . 156
83.9.7 Cgm width . . . 156
83.9.8 Cgm nofontlist . . . 157
83.10 Corel . . . 157
83.11 Debug . . . 157
83.12 Dospc . . . 157
83.13 Dumb . . . 157
83.14 Dxf . . . 158
83.15 Dxy800a . . . 158
83.16 Eepic . . . 158
83.17 Emf . . . 159
83.18 Emxvga . . . 159
83.19 Epslatex . . . 159
83.20 Epson_180dpi . . . 162
83.21 Excl . . . 162
83.22 Fig . . . 163
83.23 Ggi . . . 164
83.24 Gif . . . 164
83.24.1 Examples . . . 165
83.25 Gnugraph(GNU plotutils) . . . 165
83.26 Gpic . . . 166
83.27 Gpr . . . 166
83.28 Grass . . . 166
83.29 Hercules . . . 167
83.30 Hp2623a . . . 167

83.31 Hp2648 . . . 167
83.32 Hp500c . . . 167
83.33 Hpgl . . . 167
83.34 Hpljii . . . 168
83.35 Hppj . . . 168
83.36 Imagen . . . 168
83.37 Jpeg . . . 168
83.38 Kyo . . . 169
83.39 Latex . . . 169
83.40 Linux . . . 170
83.41 Macintosh . . . 170
83.42 Mf . . . 170
83.42.1 METAFONT Instructions . . . 170
83.43 Mgr . . . 171
83.44 Mif . . . 171
83.45 Mp . . . 172
83.45.1 Metapost Instructions . . . 173
83.46 Next . . . 174
83.47 Openstep (next) . . . 174
83.48 Pbm . . . 175
83.49 Pdf . . . 175
83.50 Pdfcairo . . . 175
83.51 Pm . . . 176
83.52 Png . . . 177
83.52.1 Examples . . . 177
83.53 Pngcairo . . . 178
83.54 Postscript . . . 178
83.54.1 Editing postscript . . . 180
83.54.2 Postscript fontfile . . . 180
83.54.3 Postscript prologue . . . 181
83.54.4 Postscript adobeglyphnames . . . 182
83.55 Pslatex and pstex . . . 182
83.56 Pstricks . . . 183
83.57 Qms . . . 184
83.58 Regis . . . 184
83.59 Rgip . . . 184
83.60 Sun . . . 184
83.61 Svg . . . 184
83.62 Svga . . . 185
83.63 Tek40 . . . 185
83.64 Tek410x . . . 185
83.65 Texdraw . . . 185
83.66 Tgif . . . 185
83.67 Tkcanvas . . . 186
83.68 Tpic . . . 187

83.69 Unixpc . . . 187
83.70 Unixplot . . . 187
83.71 Vgagl . . . 187
83.72 VWS . . . 188
83.73 Vx384 . . . 188
83.74 Windows . . . 188
83.74.1 Graph-menu . . . 189
83.74.2 Printing . . . 189
83.74.3 Text-menu . . . 189
83.74.4 Wgnuplot.ini . . . 190
83.75 Wxt . . . 190
83.76 X11 . . . 192
83.76.1 X11_fonts . . . 193
83.76.2 Command-line_options . . . 194
83.76.3 Monochrome_options . . . 194
83.76.4 Color_resources . . . 194
83.76.5 Grayscale_resources . . . 195
83.76.6 Line_resources . . . 195
83.76.7 X11 pm3d_resources . . . 196
83.76.8 X11 other_resources . . . 196
83.77 Xlib . . . 196

V Graphical User Interfaces 197

VI Bugs 197

84 Gnuplot limitations 197

85 External libraries 197

VII Index 198

Part I

Gnuplot

1 Copyright


```
Copyright (C) 1986 - 1993, 1998, 2004, 2007  Thomas Williams, Colin Kelley
```

Permission to use, copy, and distribute this software and its documentation for any purpose with or without fee is hereby granted, provided that the above copyright notice appear in all copies and that both that copyright notice and this permission notice appear in supporting documentation.

Permission to modify the software is granted, but not the right to distribute the complete modified source code. Modifications are to be distributed as patches to the released version. Permission to distribute binaries produced by compiling modified sources is granted, provided you

```
1. distribute the corresponding source modifications from the
 released version in the form of a patch file along with the binaries,
2. add special version identification to distinguish your version
 in addition to the base release version number,
3. provide your name and address as the primary contact for the
 support of your modified version, and
4. retain our contact information in regard to use of the base
 software.
```

Permission to distribute the released version of the source code along with corresponding source modifications in the form of a patch file is granted with same provisions 2 through 4 for binary distributions.

This software is provided "as is" without express or implied warranty to the extent permitted by applicable law.


```
AUTHORS

Original Software:
   Thomas Williams,  Colin Kelley.

Gnuplot 2.0 additions:
   Russell Lang, Dave Kotz, John Campbell.

Gnuplot 3.0 additions:
   Gershon Elber and many others.

Gnuplot 4.0 additions:
   See list of contributors at head of this document.
```

2 Introduction

Gnuplot is a portable command-line driven graphing utility for Linux, OS/2, MS Windows, OSX, VMS, and many other platforms. The source code is copyrighted but freely distributed (i.e., you don't have to pay for it). It was originally created to allow scientists and students to visualize mathematical functions and data interactively, but has grown to support many non-interactive uses such as web scripting. It is also used as a plotting engine by third-party applications like Octave. Gnuplot has been supported and under active development since 1986.

Gnuplot supports many types of plots in either 2D and 3D. It can draw using lines, points, boxes, contours, vector fields, surfaces, and various associated text. It also supports various specialized plot types.

Gnuplot supports many different types of output: interactive screen terminals (with mouse and hotkey input), direct output to pen plotters or modern printers, and output to many file formats (eps, fig, jpeg, LaTeX, metafont, pbm, pdf, png, postscript, svg, ...). Gnuplot is easily extensible to include new output modes. Recent additions include interactive terminals based on aquaterm (OSX) and wxWidgets (multiple platforms).

The command language of **gnuplot** is case sensitive, i.e. commands and function names written in lowercase are not the same as those written in capitals. All command names may be abbreviated as long as the abbreviation is not ambiguous. Any number of commands may appear on a line, separated by semicolons (;). Strings may be set off by either single or double quotes, although there are some subtle differences. See **syntax (p. 35)** and **quotes (p. 36)** for more details. Examples:

```
load "filename"
cd 'dir'
```

Many **gnuplot** commands have multiple options. Version 4 is less sensitive to the order of these options than earlier versions, but some order-dependence remains. If you see error messages about unrecognized options, please try again using the exact order listed in the documentation.

Commands may extend over several input lines by ending each line but the last with a backslash (\). The backslash must be the *last* character on each line. The effect is as if the backslash and newline were not there. That is, no white space is implied, nor is a comment terminated. Therefore, commenting out a continued line comments out the entire command (see **comments (p. 19)**). But note that if an error occurs somewhere on a multi-line command, the parser may not be able to locate precisely where the error is and in that case will not necessarily point to the correct line.

In this document, curly braces ({}) denote optional arguments and a vertical bar (|) separates mutually exclusive choices. **Gnuplot** keywords or **help** topics are indicated by backquotes or **boldface** (where available). Angle brackets (<>) are used to mark replaceable tokens. In many cases, a default value of the token will be taken for optional arguments if the token is omitted, but these cases are not always denoted with braces around the angle brackets.

For built-in help on any topic, type **help** followed by the name of the topic or **help ?** to get a menu of available topics.

The new **gnuplot** user should begin by reading about **plotting** (if in an interactive session, type **help plotting**).

See the simple.dem demo, also available together with other demos on the web page

http://www.gnuplot.info/demo/

Gnuplot can be started from a command line or from an icon according to the desktop environment. Running it from command line can take the syntax

```
gnuplot {OPTIONS} file1 file2 ...
```

where file1, file2, etc. are input file as in the **load** command. On X11-based systems, you can use

```
gnuplot {X11OPTIONS} {OPTIONS} file1 file2 ...
```

see your X11 documentation or rather **x11 (p. 192)** in this document.

Options interpreted by gnuplot may come anywhere on the line. Files are executed in the order specified, as are commands supplied by the -e option, for example

```
gnuplot   file1.in   -e "reset"   file2.in
```

The special filename "-" is used to force reading from stdin. **Gnuplot** exits after the last file is processed. If no load files are named, **Gnuplot** takes interactive input from stdin. See help **batch/interactive (p. 18)** for more details. The options specific to gnuplot can be listed by typing

```
gnuplot --help
```

See **command line options (p. 18)** for more details.

Hit 'h' for help about **hotkeys** and **mousing** features in interactive screen terminals (**pm**, **windows**, **wxt**, **x11**).

Section **seeking-assistance** will help you to find further information, help and FAQ.

3 Seeking-assistance

The canonical gnuplot web page can be found at

http://www.gnuplot.info

Before seeking help, please check file FAQ.pdf or the above website for

FAQ (Frequently Asked Questions) list.

If you need help as a gnuplot user, please use the newsgroup

```
comp.graphics.apps.gnuplot
```

We prefer that you read the messages through the newsgroup rather than subscribing to the mailing list which is also available and carries the same set of messages. Instructions for subscribing to gnuplot mailing lists may be found via the gnuplot development website on SourceForge

http://sourceforge.net/projects/gnuplot

The address for mailing to list members is:

```
gnuplot-info@lists.sourceforge.net
```

Bug reports and code contributions should be uploaded to the trackers at

```
http://sourceforge.net/projects/gnuplot/support
```

Please check previous bug reports if the bug you want to report has not been already fixed in a newer version of gnuplot.

The list of those interested in development version of gnuplot is:

```
gnuplot-beta@lists.sourceforge.net
```

When posting a question, please include full details of the version of **gnuplot**, the machine, and operating system you are using. A *small* script demonstrating the problem may be useful. Function plots are preferable to datafile plots. If email-ing to gnuplot-info, please state whether or not you are subscribed to the list, so that users who use news will know to email a reply to you. There is a form for such postings on the website.

4 New features introduced in version 4.4

Gnuplot version 4.4 offers many new features introduced since the preceding official version 4.2. This section lists major additions and gives a partial list of changes and minor new features. For a more exhaustive list, see the NEWS file.

4.1 Internationalization

Gnuplot 4.4 contains significantly improved support for locale settings and for UTF-8 character encodings. See **set locale (p. 108)**, **set encoding (p. 96)**, **set decimalsign (p. 94)**.

4.2 Transparency

Gnuplot now supports several forms of transparency. Any object or plot element that uses a fill style can be assigned a transparency from fully opaque to fully transparent. Image or matrix data can be plotted with an alpha channel using the new plot style **with rgbalpha**. See **fillstyle (p. 127)**, **rgbalpha (p. 44)**.

4.3 Volatile Data

The new command **refresh** is similar to **replot** except that it uses the previously-stored input data values rather than rereading the input data file. Mouse operations (zoom, rotate) will automatically use **refresh** rather than **replot** if the input data stream is marked **volatile**. Piped or in-line data is automatically treated as volatile. See **refresh (p. 80)**, **plot datafile volatile (p. 62)**.

4.4 Canvas size

In earlier versions of gnuplot, some terminal types used the values from **set size** to control also the size of the output canvas; others did not. The use of 'set size' for this purpose was deprecated in version 4.2. In version 4.4 almost all terminals now behave as follows:

set term <terminal_type> size <XX>, <YY> controls the size of the output file, or "canvas". Please see individual terminal documentation for allowed values of the size parameters. By default, the plot will fill this canvas.

set size <XX>, <YY> scales the plot itself relative to the size of the canvas. Scale values less than 1 will cause the plot to not fill the entire canvas. Scale values larger than 1 will cause only a portion of the plot to fit on the canvas. Please be aware that setting scale values larger than 1 may cause problems on some terminal types.

The major exception to this convention is the PostScript driver, which by default continues to act as it has in earlier versions. Be warned that the next version of gnuplot may change the default behaviour of the PostScript driver as well.

Example:

```
set size 0.5, 0.5
set term png size 600, 400
set output "figure.png"
plot "data" with lines
```

These commands will produce an output file "figure.png" that is 600 pixels wide and 400 pixels tall. The plot will fill the lower left quarter of this canvas. This is consistent with the way multiplot mode has always worked, however it is a change in the way the png driver worked for single plots in version 4.0.

4.5 New plot elements

The **set object** command can now be used to define fixed circles, ellipses, and polygons as well as rectangles. There is a corresponding new plot style **plot with circles**. See **circle (p. 114)**, **ellipse (p. 114)** and **polygon (p. 114)**.

4.6 New or revised terminal drivers

Two new drivers based on the cairo and pango libraries are included, **pngcairo** and **pdfcairo**. These are alternatives to the older libgd-based png driver and the older PDFLib-based pdf driver. The figures in the pdf version of this manual were prepared using the pdfcairo terminal driver.

The **canvas** terminal driver produces javascript output that draws onto the HTML canvas element of a web page. It can produce either a complete web page containing a single plot, or a script that can be embedded as part of an externally generated HTML document that perhaps contains multiple plots. The embedded plots support browser-side mousing, including zoom/unzoom.

The **lua** terminal driver creates an data intended to be further processed by a script in the lua programming language. At this point only one such lua script, gnuplot-tikz.lua, is available. It produces a TeX document suitable for use with the latex TikZ package. Other lua scripts could be written to process the gnuplot output for use with other TeX packages, or with other non-TeX tools.

Set term tikz is shorthand for **set term lua tikz**. As decribed above, it uses the generic lua terminal and an external lua script to produce a latex document.

4.7 New smoothing algorithms

New smoothing algorithms have been added for both 2- and 3-dimensional plots. **smooth kdensity** and **smooth cumul** can be used with **plot** to draw smooth histograms and cumulative distribution functions, resp. For use with **splot** several new smoothing kernels have been added to **dgrid3d**. See **smooth (p. 69) dgrid3d (p. 95)**.

5 Backwards compatibility

Gnuplot version 4.0 deprecated certain syntax used in earlier versions, but continued to recognize it. This is now under the control of a configuration option, and can be disabled as follows:

```
./configure --disable-backwards-compatibility
```

Notice: Deprecated syntax items may be disabled permanently in some future version of gnuplot.

One major difference is the introduction of keywords to disambiguate complex commands, particularly commands containing string variables. A notable issue was the use of bare numbers to specify offsets, line and point types. Illustrative examples:

Deprecated:

```
set title "Old" 0,-1
set data linespoints
plot 1 2 4               # horizontal line at y=1
```

New:

```
TITLE = "New"
```

```
set title TITLE offset char 0, char -1
set style data linespoints
plot 1 linetype 2 pointtype 4
```

6 Batch/Interactive Operation

Gnuplot may be executed in either batch or interactive modes, and the two may even be mixed together on many systems.

Any command-line arguments are assumed to be either program options (first character is -) or names of files containing **gnuplot** commands. The option -e "command" may be used to force execution of a gnuplot command. Each file or command string will be executed in the order specified. The special filename "-" is indicates that commands are to be read from stdin. **Gnuplot** exits after the last file is processed. If no load files and no command strings are specified, **gnuplot** accepts interactive input from stdin.

Both the **exit** and **quit** commands terminate the current command file and **load** the next one, until all have been processed.

Examples:

To launch an interactive session:

```
gnuplot
```

To launch a batch session using two command files "input1" and "input2":

```
gnuplot input1 input2
```

To launch an interactive session after an initialization file "header" and followed by another command file "trailer":

```
gnuplot header - trailer
```

To give **gnuplot** commands directly in the command line, using the "-persist" option so that the plot remains on the screen afterwards:

```
gnuplot -persist -e "set title 'Sine curve'; plot sin(x)"
```

To set user-defined variables a and s prior to executing commands from a file:

```
gnuplot -e "a=2; s='file.png'" input.gpl
```

7 Command-line-editing

Command-line editing and command history are supported using either an external gnu readline library, an external BSD libedit library, or a built-in equivalent. This choice is a configuration option at the time gnuplot is built.

The editing commands of the built-in version are given below. The gnu readline and BSD libedit libraries have their own documentation.

Command-line Editing Commands	
Character	Function
	Line Editing
^B	move back a single character.
^F	move forward a single character.
^A	move to the beginning of the line.
^E	move to the end of the line.
^H, DEL	delete the previous character.
^D	delete the current character.
^K	delete from current position to the end of line.
^L, ^R	redraw line in case it gets trashed.
^U	delete the entire line.
^W	delete from the current word to the end of line.
	History
^P	move back through history.
^N	move forward through history.

8 Comments

Comments are supported as follows: a # may appear in most places in a line and **gnuplot** will ignore the rest of the line. It will not have this effect inside quotes, inside numbers (including complex numbers), inside command substitutions, etc. In short, it works anywhere it makes sense to work.

See also **set datafile commentschars (p. 93)** for specifying comment characters in data files. Note that if a comment line ends in '\' then the subsequent line is also treated as a comment.

9 Coordinates

The commands **set arrow**, **set key**, **set label** and **set object** allow you to draw something at an arbitrary position on the graph. This position is specified by the syntax:

```
{<system>} <x>, {<system>} <y> {,{<system>} <z>}
```

Each <system> can either be **first**, **second**, **graph**, **screen**, or **character**.

first places the x, y, or z coordinate in the system defined by the left and bottom axes; **second** places it in the system defined by the second axes (top and right); **graph** specifies the area within the axes — 0,0 is bottom left and 1,1 is top right (for splot, 0,0,0 is bottom left of plotting area; use negative z to get to the base — see **set xyplane (p. 141)**); **screen** specifies the screen area (the entire area — not just the portion selected by **set size**), with 0,0 at bottom left and 1,1 at top right; and **character** gives the position in character widths and heights from the bottom left of the screen area (screen 0,0), **character** coordinates depend on the chosen font size.

If the coordinate system for x is not specified, **first** is used. If the system for y is not specified, the one used for x is adopted.

In some cases, the given coordinate is not an absolute position but a relative value (e.g., the second position in **set arrow** ... **rto**). In most cases, the given value serves as difference to the first position. If the given coordinate resides in a logarithmic axis the value is interpreted as factor. For example,

```
set logscale x
set arrow 100,5 rto 10,2
```

plots an arrow from position 100,5 to position 1000,7 since the x axis is logarithmic while the y axis is linear.

If one (or more) axis is timeseries, the appropriate coordinate should be given as a quoted time string according to the **timefmt** format string. See **set xdata (p. 136)** and **set timefmt (p. 132)**. **Gnuplot** will also accept an integer expression, which will be interpreted as seconds from 1 January 2000.

10 Datastrings

Data files may contain string data consisting of either an arbitrary string of printable characters containing no whitespace or an arbitrary string of characters, possibly including whitespace, delimited by double quotes. The following sample line from a datafile is interpreted to contain four columns, with a text field in column 3:

```
1.000 2.000 "Third column is all of this text" 4.00
```

Text fields can be positioned within a 2-D or 3-D plot using the commands:

```
plot 'datafile' using 1:2:4 with labels
splot 'datafile using 1:2:3:4 with labels
```

A column of text data can also be used to label the ticmarks along one or more of the plot axes. The example below plots a line through a series of points with (X,Y) coordinates taken from columns 3 and 4 of the input datafile. However, rather than generating regularly spaced tics along the x axis labeled numerically, gnuplot will position a tic mark along the x axis at the X coordinate of each point and label the tic mark with text taken from column 1 of the input datafile.

```
set xtics
plot 'datafile' using 3:4:xticlabels(1) with linespoints
```

There is also an option that will interpret the first entry in a column of input data (i.e. the column heading) as a text field, and use it as the key title for data plotted from that column. The example given below will use the first entry in column 2 to generate a title in the key box, while processing the remainder of columns 2 and 4 to draw the required line:

```
plot 'datafile' using 1:(f($2)/$4) with lines title columnhead(2)
```

See **set style labels (p. 46)**, **using xticlabels (p. 73)**, **plot title (p. 76)**, **using (p. 71)**.

11 Enhanced text mode

Many terminal types support an enhanced text mode in which additional formatting information is embedded in the text string. For example, "x^2" will write x-squared as we are used to seeing it, with a superscript 2. This mode is normally selected when you set the terminal, e.g. "set term png enhanced", but may also be toggled afterward using "set termoption enhanced", or by marking individual strings as in "set label 'x_2' noenhanced".

Enhanced Text Control Codes			
Control	Example	Result	Explanation
`^`	`a^x`	a^x	superscript
`_`	`a_x`	a_x	subscript
`@`	`a@^b_{cd}`	a^b_{cd}	phantom box (occupies no width)
`&`	`d&{space}b`	d␣␣␣␣␣b	inserts space of specified length
`~`	`~a{.8-}`	$\tilde{a}$	overprints '-' on 'a', raised by .8 times the current fontsize

Braces can be used to place multiple-character text where a single character is expected (e.g., 2^{10}). To change the font and/or size, use the full form: {/[fontname][=fontsize | *fontscale] text}. Thus {/Symbol=20 G} is a 20 pt GAMMA and {/*0.75 K} is a K at three-quarters of whatever fontsize is currently in effect. (The '/' character MUST be the first character after the '{'.)

The phantom box is useful for a@^b_c to align superscripts and subscripts but does not work well for overwriting an accent on a letter. For the latter, it is much better to use an encoding (e.g. iso_8859_1 or utf8) that contains a large variety of letters with accents or other diacritical marks. See **set encoding (p. 96)**. Since the box is non-spacing, it is sensible to put the shorter of the subscript or superscript in the box (that is, after the @).

Space equal in length to a string can be inserted using the '&' character. Thus

```
'abc&{def}ghi'
```

would produce

```
'abc   ghi'.
```

The '~ ' character causes the next character or bracketed text to be overprinted by the following character or bracketed text. The second text will be horizontally centered on the first. Thus '~ a/' will result in an 'a' with a slash through it. You can also shift the second text vertically by preceding the second text with a number, which will define the fraction of the current fontsize by which the text will be raised or lowered. In this case the number and text must be enclosed in brackets because more than one character is necessary. If the overprinted text begins with a number, put a space between the vertical offset and the text ('~ {abc}{.5 000}'); otherwise no space is needed ('~ {abc}{.5 — }'). You can change the font for one or both strings ('~ a{.5 /*.2 o}' — an 'a' with a one-fifth-size 'o' on top — and the space between the number and the slash is necessary), but you can't change it after the beginning of the string. Neither can you use any other special syntax within either string. You can, of course, use control characters by escaping them (see below), such as '~ a{\^}'

You can access special symbols numerically by specifying \character-code (in octal), e.g., {/Symbol \245} is the symbol for infinity. This does not work for multibyte encodings like UTF-8, however. In a UTF-8 environment, you should be able to enter multibyte sequences implicitly by typing or otherwise selecting the character you want.

You can escape control characters using \, e.g., \\, \{, and so on.

But be aware that strings in double-quotes are parsed differently than those enclosed in single-quotes. The major difference is that backslashes may need to be doubled when in double-quoted strings.

Examples (these are hard to describe in words — try them!):

```
set xlabel 'Time (10^6 {/Symbol m}s)'
set title '{/Symbol=18 \\362@_{/=9.6 0}^{/=12 x}} \\
           {/Helvetica e^{-{/Symbol m}^2/2} d}{/Symbol m}'
```

The file "ps_guide.ps" in the /docs/psdoc subdirectory of the gnuplot source distribution contains more examples of the enhanced syntax.

12 Environment

A number of shell environment variables are understood by **gnuplot**. None of these are required, but may be useful.

If GNUTERM is defined, it is used as the name of the terminal type to be used. This overrides any terminal type sensed by **gnuplot** on start-up, but is itself overridden by the .gnuplot (or equivalent) start-up file (see **start-up (p. 33)**) and, of course, by later explicit changes.

GNUHELP may be defined to be the pathname of the HELP file (gnuplot.gih).

On VMS, the logical name GNUPLOT$HELP should be defined as the name of the help library for **gnuplot**. The **gnuplot** help can be put inside any system help library, allowing access to help from both within and outside **gnuplot** if desired.

On Unix, HOME is used as the name of a directory to search for a .gnuplot file if none is found in the current directory. On AmigaOS, MS-DOS, Windows and OS/2, GNUPLOT is used. On Windows, the NT-specific variable USERPROFILE is tried, too. VMS, SYS$LOGIN: is used. Type **help start-up**.

On Unix, PAGER is used as an output filter for help messages.

On Unix and AmigaOS, SHELL is used for the **shell** command. On MS-DOS and OS/2, COMSPEC is used for the **shell** command.

FIT_SCRIPT may be used to specify a **gnuplot** command to be executed when a fit is interrupted — see **fit (p. 52)**. FIT_LOG specifies the default filename of the logfile maintained by fit.

GNUPLOT_LIB may be used to define additional search directories for data and command files. The variable may contain a single directory name, or a list of directories separated by a platform-specific path separator, eg. ':' on Unix, or ';' on DOS/Windows/OS/2/Amiga platforms. The contents of GNUPLOT_LIB are appended to the **loadpath** variable, but not saved with the **save** and **save set** commands.

Several gnuplot terminal drivers access TrueType fonts via the gd library. For these drivers the font search path is controlled by the environmental variable GDFONTPATH. Furthermore, a default font for these drivers may be set via the environmental variable GNUPLOT_DEFAULT_GDFONT.

The postscript terminal uses its own font search path. It is controlled by the environmental variable GNUPLOT_FONTPATH. The format is the same as for GNUPLOT_LIB. The contents of GNUPLOT_FONTPATH are appended to the **fontpath** variable, but not saved with the **save** and **save set** commands.

GNUPLOT_PS_DIR is used by the postscript driver to use external prologue files. Depending on the build process, gnuplot contains either a builtin copy of those files or simply a default hardcoded path. Use this variable to test the postscript terminal with custom prologue files. See **postscript prologue (p. 181)**.

13 Expressions

In general, any mathematical expression accepted by C, FORTRAN, Pascal, or BASIC is valid. The precedence of these operators is determined by the specifications of the C programming language. White space (spaces and tabs) is ignored inside expressions.

Complex constants are expressed as {<real>,<imag>}, where <real> and <imag> must be numerical constants. For example, {3,2} represents 3 + 2i; {0,1} represents 'i' itself. The curly braces are explicitly required here.

Note that gnuplot uses both "real" and "integer" arithmetic, like FORTRAN and C. Integers are entered as "1", "-10", etc; reals as "1.0", "-10.0", "1e1", 3.5e-1, etc. The most important difference between the two forms is in division: division of integers truncates: 5/2 = 2; division of reals does not: 5.0/2.0 = 2.5. In mixed expressions, integers are "promoted" to reals before evaluation: 5/2e0 = 2.5. The result of division of a negative integer by a positive one may vary among compilers. Try a test like "print -5/2" to determine if your system chooses -2 or -3 as the answer.

The integer expression "1/0" may be used to generate an "undefined" flag, which causes a point to ignored; the **ternary** operator gives an example. Or you can use the pre-defined variable NaN to achieve the same result.

The real and imaginary parts of complex expressions are always real, whatever the form in which they are entered: in {3,2} the "3" and "2" are reals, not integers.

Gnuplot can also perform simple operations on strings and string variables. For example, the expression ("A" . "B" eq "AB") evaluates as true, illustrating the string concatenation operator and the string equality operator.

A string which contains a numerical value is promoted to the corresponding integer or real value if used in a numerical expression. Thus ("3" + "4" == 7) and (6.78 == "6.78") both evaluate to true. An integer, but not a real or complex value, is promoted to a string if used in string concatenation. A typical case is the use of integers to construct file names or other strings; e.g. ("file" . 4 eq "file4") is true.

Substrings can be specified using a postfixed range descriptor [beg:end]. For example, "ABCDEF"[3:4] == "CD" and "ABCDEF"[4:*] == "DEF" The syntax "string"[beg:end] is exactly equivalent to calling the built-in string-valued function substr("string",beg,end), except that you cannot omit either beg or end from the function call.

13.1 Functions

The functions in **gnuplot** are the same as the corresponding functions in the Unix math library, except that all functions accept integer, real, and complex arguments, unless otherwise noted.

For those functions that accept or return angles that may be given in either degrees or radians (sin(x), cos(x), tan(x), asin(x), acos(x), atan(x), atan2(x) and arg(z)), the unit may be selected by **set angles**, which defaults to radians.

Math library functions		
Function	Arguments	Returns
abs(x)	any	absolute value of x, $\|x\|$; same type
abs(x)	complex	length of x, $\sqrt{\text{real}(x)^2 + \text{imag}(x)^2}$
acos(x)	any	$\cos^{-1} x$ (inverse cosine)
acosh(x)	any	$\cosh^{-1} x$ (inverse hyperbolic cosine) in radians
arg(x)	complex	the phase of x
asin(x)	any	$\sin^{-1} x$ (inverse sin)
asinh(x)	any	$\sinh^{-1} x$ (inverse hyperbolic sin) in radians
atan(x)	any	$\tan^{-1} x$ (inverse tangent)
atan2(y,x)	int or real	$\tan^{-1}(y/x)$ (inverse tangent)
atanh(x)	any	$\tanh^{-1} x$ (inverse hyperbolic tangent) in radians
EllipticK(k)	real k ∈ (-1:1)	$K(k)$ complete elliptic integral of the first kind
EllipticE(k)	real k ∈ [-1:1]	$E(k)$ complete elliptic integral of the second kind
EllipticPi(n,k)	real n<1, real k ∈ (-1:1)	$\Pi(n,k)$ complete elliptic integral of the third kind
besj0(x)	int or real	j_0 Bessel function of x, in radians
besj1(x)	int or real	j_1 Bessel function of x, in radians
besy0(x)	int or real	y_0 Bessel function of x, in radians
besy1(x)	int or real	y_1 Bessel function of x, in radians
ceil(x)	any	$\lceil x \rceil$, smallest integer not less than x (real part)
cos(x)	any	$\cos x$, cosine of x
cosh(x)	any	$\cosh x$, hyperbolic cosine of x in radians
erf(x)	any	erf(real(x)), error function of real(x)
erfc(x)	any	erfc(real(x)), 1.0 - error function of real(x)
exp(x)	any	e^x, exponential function of x
floor(x)	any	$\lfloor x \rfloor$, largest integer not greater than x (real part)
gamma(x)	any	gamma(real(x)), gamma function of real(x)
ibeta(p,q,x)	any	ibeta(real(p,q,x)), ibeta function of real(p,q,x)
inverf(x)	any	inverse error function of real(x)
igamma(a,x)	any	igamma(real(a,x)), igamma function of real(a,x)
imag(x)	complex	imaginary part of x as a real number
invnorm(x)	any	inverse normal distribution function of real(x)
int(x)	real	integer part of x, truncated toward zero
lambertw(x)	real	Lambert W function
lgamma(x)	any	lgamma(real(x)), lgamma function of real(x)
log(x)	any	$\log_e x$, natural logarithm (base e) of x
log10(x)	any	$\log_{10} x$, logarithm (base 10) of x
norm(x)	any	normal distribution (Gaussian) function of real(x)
rand(x)	any	rand(x), pseudo random number generator
real(x)	any	real part of x
sgn(x)	any	1 if $x > 0$, -1 if $x < 0$, 0 if $x = 0$. imag(x) ignored
sin(x)	any	$\sin x$, sine of x
sinh(x)	any	$\sinh x$, hyperbolic sine of x in radians
sqrt(x)	any	$\sqrt{x}$, square root of x
tan(x)	any	$\tan x$, tangent of x
tanh(x)	any	$\tanh x$, hyperbolic tangent of x in radians

String functions		
Function	Arguments	Returns
gprintf("format",x)	any	string result from applying gnuplot's format parser
sprintf("format",x,...)	multiple	string result from C-language sprintf
strlen("string")	string	int length of string
strstrt("string","key")	strings	int index of first character of substring "key"
substr("string",beg,end)	multiple	string "string"[beg:end]
strftime("timeformat",t)	any	string result from applying gnuplot's time parser
strptime("timeformat",s)	string	seconds since year 2000 as given in string s
system("command")	string	string containing output stream of shell command
word("string",n)	string, int	returns the nth word in "string"
words("string")	string	returns the number of words in "string"

other **gnuplot** functions		
Function	Arguments	Returns
column(x)	int	column x during datafile manipulation.
defined(X)	variable name	[DEPRECATED] returns 1 if X is defined, 0 otherwise.
exists("X")	"variable name"	returns 1 if a variable named X is defined, 0 otherwise.
stringcolumn(x)	int	content of column x as a string.
timecolumn(x)	int	timecolumn x during datafile manipulation.
tm_hour(x)	int	the hour
tm_mday(x)	int	the day of the month
tm_min(x)	int	the minute
tm_mon(x)	int	the month
tm_sec(x)	int	the second
tm_wday(x)	int	the day of the week
tm_yday(x)	int	the day of the year
tm_year(x)	int	the year
valid(x)	int	test validity of column(x) during datafile manip.

13.1.1 Elliptic integrals

The **EllipticK(k)** function returns the complete elliptic integral of the first kind, i.e. the definite integral between 0 and pi/2 of the function **(1-(k*sin(p))**2)**(-0.5)**. The domain of **k** is -1 to 1 (exclusive).

The **EllipticE(k)** function returns the complete elliptic integral of the second kind, i.e. the definite integral between 0 and pi/2 of the function **(1-(k*sin(p))**2)**0.5**. The domain of **k** is -1 to 1 (inclusive).

The **EllipticPi(n,k)** function returns the complete elliptic integral of the third kind, i.e. the definite integral between 0 and pi/2 of the function **(1-(k*sin(p))**2)**(-0.5)/(1-n*sin(p)**2)**. The parameter **n** must be less than 1, while **k** must lie between -1 and 1 (exclusive). Note that by definition EllipticPi(0,k) == EllipticK(k) for all possible values of **k**.

13.1.2 Random number generator

Some older versions of gnuplot used rand(x>0) to produce sequential pseudo-random numbers. The current behavior is as follows:

```
`rand(0)`  returns a pseudo random number in the interval [0:1] generated
           from the current value of two internal 32-bit seeds.
`rand(-1)` resets both seeds to a standard value.
`rand(x)`  for x>0 sets both seeds to a value based on the value of x.
`rand({x,y})` for x>0 sets seed1 to x and seed2 to y.
```

13.2 Operators

The operators in **gnuplot** are the same as the corresponding operators in the C programming language, except that all operators accept integer, real, and complex arguments, unless otherwise noted. The ** operator (exponentiation) is supported, as in FORTRAN.

Parentheses may be used to change order of evaluation.

13.2.1 Unary

The following is a list of all the unary operators and their usages:

Unary Operators		
Symbol	Example	Explanation
`-`	`-a`	unary minus
`+`	`+a`	unary plus (no-operation)
`~`	`~a`	* one's complement
`!`	`!a`	* logical negation
`!`	`a!`	* factorial
`$`	`$3`	* call arg/column during 'using' manipulation

(*) Starred explanations indicate that the operator requires an integer argument.

Operator precedence is the same as in Fortran and C. As in those languages, parentheses may be used to change the order of operation. Thus -2**2 = -4, but (-2)**2 = 4.

The factorial operator returns a real number to allow a greater range.

13.2.2 Binary

The following is a list of all the binary operators and their usages:

<table>
<tr><th colspan="3">Binary Operators</th></tr>
<tr><th>Symbol</th><th>Example</th><th>Explanation</th></tr>
<tr><td>**</td><td>a**b</td><td>exponentiation</td></tr>
<tr><td>*</td><td>a*b</td><td>multiplication</td></tr>
<tr><td>/</td><td>a/b</td><td>division</td></tr>
<tr><td>%</td><td>a%b</td><td>* modulo</td></tr>
<tr><td>+</td><td>a+b</td><td>addition</td></tr>
<tr><td>-</td><td>a-b</td><td>subtraction</td></tr>
<tr><td>==</td><td>a==b</td><td>equality</td></tr>
<tr><td>!=</td><td>a!=b</td><td>inequality</td></tr>
<tr><td><</td><td>a<b</td><td>less than</td></tr>
<tr><td><=</td><td>a<=b</td><td>less than or equal to</td></tr>
<tr><td>></td><td>a>b</td><td>greater than</td></tr>
<tr><td>>=</td><td>a>=b</td><td>greater than or equal to</td></tr>
<tr><td>&</td><td>a&b</td><td>* bitwise AND</td></tr>
<tr><td>^</td><td>a^b</td><td>* bitwise exclusive OR</td></tr>
<tr><td>|</td><td>a|b</td><td>* bitwise inclusive OR</td></tr>
<tr><td>&&</td><td>a&&b</td><td>* logical AND</td></tr>
<tr><td>||</td><td>a||b</td><td>* logical OR</td></tr>
<tr><td>=</td><td>a = b</td><td>assignment</td></tr>
<tr><td>,</td><td>(a,b)</td><td>serial evaluation</td></tr>
<tr><td>.</td><td>A.B</td><td>string concatenation</td></tr>
<tr><td>eq</td><td>A eq B</td><td>string equality</td></tr>
<tr><td>ne</td><td>A ne B</td><td>string inequality</td></tr>
</table>

(*) Starred explanations indicate that the operator requires integer arguments. Capital letters A and B indicate that the operator requires string arguments.

Logical AND (&&) and OR (||) short-circuit the way they do in C. That is, the second && operand is not evaluated if the first is false; the second || operand is not evaluated if the first is true.

Serial evaluation occurs only in parentheses and is guaranteed to proceed in left to right order. The value of the rightmost subexpression is returned.

13.2.3 Ternary

There is a single ternary operator:

Ternary Operator		
Symbol	Example	Explanation
?:	a?b:c	ternary operation

The ternary operator behaves as it does in C. The first argument (a), which must be an integer, is evaluated. If it is true (non-zero), the second argument (b) is evaluated and returned; otherwise the third argument (c) is evaluated and returned.

The ternary operator is very useful both in constructing piecewise functions and in plotting points only when certain conditions are met.

Examples:

Plot a function that is to equal sin(x) for 0 <= x < 1, 1/x for 1 <= x < 2, and undefined elsewhere:

```
f(x) = 0<=x && x<1 ? sin(x) : 1<=x && x<2 ? 1/x : 1/0
plot f(x)
```

Note that **gnuplot** quietly ignores undefined values, so the final branch of the function (1/0) will produce no plottable points. Note also that f(x) will be plotted as a continuous function across the discontinuity if a line style is used. To plot it discontinuously, create separate functions for the two pieces. (Parametric functions are also useful for this purpose.)

For data in a file, plot the average of the data in columns 2 and 3 against the datum in column 1, but only if the datum in column 4 is non-negative:

```
plot 'file' using 1:( $4<0 ? 1/0 : ($2+$3)/2 )
```

For an explanation of the **using** syntax, please see **plot datafile using (p. 71)**.

13.3 Gnuplot-defined variables

Gnuplot maintains a number of read-only variables that reflect the current internal state of the program and the most recent plot. These variables begin with the prefix "GPVAL_". Examples include GPVAL_TERM, GPVAL_X_MIN, GPVAL_X_MAX, GPVAL_Y_MIN. Type **show variables all** to display the complete list and current values. Values related to axes parameters (ranges, log base) are values used during the last plot, not those currently **set**.

The read-only variable GPVAL_ERRNO is set to a non-zero value if any gnuplot command terminates early due to an error. The most recent error message is stored in the string variable GPVAL_ERRMSG. Both GPVAL_ERRNO and GPVAL_ERRMSG can be cleared using the command **reset errors**.

Interactive terminals with **mouse** functionality maintain read-only variables with the prefix "MOUSE_". See **mouse variables (p. 32)** for details.

The **fit** mechanism uses several variables with names that begin "FIT_". It is safest to avoid using such names. "FIT_LIMIT", however, is one that you may wish to redefine. Under **set fit errorvariables**, the error for each fitted parameter will be stored in a variable named like the parameter, but with "_err" appended. See the documentation on **fit (p. 52)** for details.

See **user-defined variables (p. 26)**, **reset errors (p. 81)**, **mouse variables (p. 32)**, and **fit (p. 52)**.

13.4 User-defined variables and functions

New user-defined variables and functions of one through twelve variables may be declared and used anywhere, including on the **plot** command itself.

User-defined function syntax:

```
<func-name>( <dummy1> {,<dummy2>} ... {,<dummy12>} ) = <expression>
```

where <expression> is defined in terms of <dummy1> through <dummy12>.

User-defined variable syntax:

```
<variable-name> = <constant-expression>
```

Examples:

```
w = 2
q = floor(tan(pi/2 - 0.1))
f(x) = sin(w*x)
sinc(x) = sin(pi*x)/(pi*x)
delta(t) = (t == 0)
ramp(t) = (t > 0) ? t : 0
min(a,b) = (a < b) ? a : b
comb(n,k) = n!/(k!*(n-k)!)
len3d(x,y,z) = sqrt(x*x+y*y+z*z)
plot f(x) = sin(x*a), a = 0.2, f(x), a = 0.4, f(x)

file = "mydata.inp"
file(n) = sprintf("run_%d.dat",n)
```

The final two examples illustrate a user-defined string variable and a user-defined string function.

Note that the variables **pi** (3.14159...) and **NaN** (IEEE "Not a Number") are already defined. You can redefine these to something else if you really need to. The original values can be recovered by setting:

```
NaN = GPVAL_NaN
pi  = GPVAL_pi
```

Other variables may be defined under various gnuplot operations like mousing in interactive terminals or fitting; see **gnuplot-defined variables (p. 26)** for details.

You can check for existence of a given variable V by the exists("V") expression. For example

```
a = 10
if (exists("a")) print "a is defined"
if (!exists("b")) print "b is not defined"
```

Valid names are the same as in most programming languages: they must begin with a letter, but subsequent characters may be letters, digits, or "_".

Each function definition is made available as a special string-valued variable with the prefix 'GPFUN_'.

Example:

```
set label GPFUN_sinc at graph .05,.95
```

See **show functions (p. 100)**, **functions (p. 22)**, **gnuplot-defined variables (p. 26)**, **macros (p. 34)**.

14 Fonts

Gnuplot does not provide any fonts of its own. It relies on external font handling, the details of which unfortunately vary from one terminal type to another. Brief documentation of font mechanisms that apply to more than one terminal type is given here. For information on font use by other individual terminals, see the documentation for that terminal.

14.1 Cairo (pdfcairo, pngcairo, wxt terminals)

Sorry, this section is under construction. These terminals find and access fonts using the external fontconfig tool set. Please see the

`fontconfig user manual.`

It is usually sufficient in gnuplot to request a font by a generic name and size, letting fontconfig substitute a similar font if necessary. The following will probably all work:

```
set term pdfcairo font "sans,12"
set term pdfcairo font "Times,12"
set term pdfcairo font "Times-New-Roman,12"
```

14.2 Gd (png, gif, jpeg terminals)

Font handling for the png, gif, and jpeg terminals is done by the external library libgd. Five basic fonts are provided directly by libgd. These are **tiny** (5x8 pixels), **small** (6x12 pixels), **medium**, (7x13 Bold), **large** (8x16) or **giant** (9x15 pixels). These fonts cannot be scaled or rotated. Use one of these keywords instead of the **font** keyword. E.g.

```
set term png tiny
```

On most systems libgd also provides access to Adobe Type 1 fonts (*.pfa) and TrueType fonts (*.ttf). You must give the name of the font file, not the name of the font inside it, in the form "<face> {,<pointsize>}". <face> is either the full pathname to the font file, or the first part of a filename in one of the directories listed in the GDFONTPATH environmental variable. That is, 'set term png font "Face"' will look for a font file named either <somedirectory>/Face.ttf or <somedirectory>/Face.pfa. For example, if GDFONTPATH contains **/usr/local/fonts/ttf:/usr/local/fonts/pfa** then the following pairs of commands are equivalent

```
set term png font "arial"
set term png font "/usr/local/fonts/ttf/arial.ttf"
set term png font "Helvetica"
set term png font "/usr/local/fonts/pfa/Helvetica.pfa"
```

To request a default font size at the same time:

```
set term png font "arial,11"
```

Both TrueType and Adobe Type 1 fonts are fully scalable and rotatable. If no specific font is requested in the "set term" command, gnuplot checks the environmental variable GNUPLOT_DEFAULT_GDFONT to see if there is a preferred default font.

14.3 Postscript (also encapsulated postscript *.eps)

PostScript font handling is done by the printer or viewing program. Gnuplot can create valid PostScript or encapsulated PostScript (*.eps) even if no fonts at all are installed on your computer. Gnuplot simply refers to the font by name in the output file, and assumes that the printer or viewing program will know how to find or approximate a font by that name.

All PostScript printers or viewers should know about the standard set of Adobe fonts **Times-Roman**, **Helvetica**, **Courier**, and **Symbol**. It is likely that many additional fonts are also available, but the specific set depends on your system or printer configuration. Gnuplot does not know or care about this; the output *.ps or *.eps files that it creates will simply refer to whatever font names you request.

Thus

```
set term postscript eps font "Times-Roman,12"
```

will produce output that is suitable for all printers and viewers.

On the other hand

```
set term postscript eps font "Garamond-Premier-Pro-Italic"
```

will produce an output file that contains valid PostScript, but since it refers to a specialized font, only some printers or viewers will be able to display the specific font that was requested. Most will substitute a different font.

However, it is possible to embed a specific font in the output file so that all printers will be able to use it. This requires that the a suitable font description file is available on your system. Note that some font files require specific licensing if they are to be embedded in this way. See **postscript fontfile (p. 180)** for more detailed description and examples.

15 Glossary

Throughout this document an attempt has been made to maintain consistency of nomenclature. This cannot be wholly successful because as **gnuplot** has evolved over time, certain command and keyword names have been adopted that preclude such perfection. This section contains explanations of the way some of these terms are used.

A "page" or "screen" or "canvas" is the entire area addressable by **gnuplot**. On a desktop it is a full window; on a plotter, it is a single sheet of paper; in svga mode it is the full monitor screen.

A screen may contain one or more "plots". A plot is defined by an abscissa and an ordinate, although these need not actually appear on it, as well as the margins and any text written therein.

A plot contains one "graph". A graph is defined by an abscissa and an ordinate, although these need not actually appear on it.

A graph may contain one or more "lines". A line is a single function or data set. "Line" is also a plotting style. The word will also be used in sense "a line of text". Presumably the context will remove any ambiguity.

The lines on a graph may have individual names. These may be listed together with a sample of the plotting style used to represent them in the "key", sometimes also called the "legend".

The word "title" occurs with multiple meanings in **gnuplot**. In this document, it will always be preceded by the adjective "plot", "line", or "key" to differentiate among them. A 2D graph may have up to four labelled **axes**. The names of the four axes are "x" for the axis along the bottom border of the plot, "y" for the axis along the left border, "x2" for the top border, and "y2" for the right border. See **axes (p. 62)**.

A 3D graph may have up to three labelled **axes** – "x", "y" and "z". It is not possible to say where on the graph any particular axis will fall because you can change the direction from which the graph is seen with **set view**.

When discussing data files, the term "record" will be resurrected and used to denote a single line of text in the file, that is, the characters between newline or end-of-record characters. A "point" is the datum extracted from a single record. A "datablock" is a set of points from consecutive records, delimited by blank records. A line, when referred to in the context of a data file, is a subset of a datablock.

16 Linetype, colors, and styles

Each gnuplot terminal type provides a set of distinct "linetypes". These may differ in color, in thickness, in dot/dash pattern, or in some combination of color and dot/dash. The default linetypes for a particular terminal can be previewed by issuing the **test** command after setting the terminal type. The pre-defined colors and dot/dash patterns are not guaranteed to be consistent for all terminal types, but all terminals use the special linetype -1 to mean a solid line in the primary foreground color (normally black). By default, successive functions or datafiles plotted by a single command will be assigned successive linetypes. You can override this default by specifying a particular linetype for any function, datafile, or plot element.

Examples:

```
plot "foo", "bar"                 # plot two files using linetypes 1, 2
plot sin(x) linetype 4            # terminal-specific linetype color 4
plot sin(x) lt -1                 # black
```

For many terminal types it is also possible to assign user-defined colors using explicit rgb (red, green, blue) values, named colors, or color values that refer to the current PM3D palette.

Examples:

```
plot sin(x) lt rgb "violet"       # one of gnuplot's named colors
plot sin(x) lt rgb "#FF00FF"      # explicit RGB triple in hexadecimal
plot sin(x) lt palette cb -45     # whatever color corresponds to -45
                                  # in the current cbrange of the palette
plot sin(x) lt palette frac 0.3   # fractional value along the palette
```

See **show colornames (p. 91)**, **set palette (p. 119)**, **cbrange (p. 144)**.

For terminals that support dot/dash patterns, each default linetype has both a dot-dash pattern and a default color. However, you can override the default color by using the keyword **linecolor**, abbreviated **lc**. For example, the postscript terminal provides a dashed blue line as linetype 3. The plot commands below use this same dash pattern for three plots, one in blue (the default), another in red (the default for linetype 1), and a third in gold.

Example:

```
set term postscript dashed color
plot 'foo' lt 3, 'baz' lt 3 linecolor 1, 'bar' lt 3 lc rgb 'gold'
```

Lines can have additional properties such as linewidth. You can associate these various properties, as well as equivalent properties for point symbols, into user-defined "line styles" using the command **set style line**. Once you have defined a linestyle, you can use it in a plot command to control the appearance of one or more plot elements.

Examples:

```
# define a new line style with terminal-independent color cyan,
# linewidth 3, and associated point type 6 (a circle with a dot in it).
set style line 5 lt rgb "cyan" lw 3 pt 6
plot sin(x) with linespoints ls 5          # user-defined line style 5
```

See **linestyle (p. 129)**, **set style line (p. 129)**.

16.1 Colorspec

Many commands allow you to specify a linetype with an explicit color. Terminal-independent color choice is only possible for terminals that support RGB color or pm3d palettes.

Syntax:

```
 ... {linecolor | lc} {<colorspec> | <n>}
 ... {textcolor | tc} {<colorspec> | {linetype | lt} <n>}
```

where <colorspec> has one of the following forms:

```
rgbcolor "colorname"
rgbcolor "#RRGGBB"
rgbcolor variable       # color is read from input file
palette frac <val>      # <val> runs from 0 to 1
palette cb <value>      # <val> lies within cbrange
palette z
variable                # color index is read from input file
```

The "<n>" is the linetype number the color of which is used, see **test (p. 149)**.

"colorname" refers to one of the color names built in to gnuplot. For a list of the available names, see **show colornames (p. 91)**.

"#RRGGBB" is a hexadecimal constant preceded by the "#" symbol. The RRGGBB represents the red, green, and blue components of the color, each on a scale from 0 - 255. For example, magenta = full-scale red + full-scale blue would be represented by #FF00FF, which is the hexadecimal representation of (255 << 16) + (0 << 8) + (255).

The color palette is a linear gradient of colors that smoothly maps a single numerical value onto a particular color. Two such mappings are always in effect. **palette frac** maps a fractional value between 0 and 1 onto the full range of the color palette. **palette cb** maps the range of the color axis onto the same palette. See **set cbrange (p. 144)**. See also **set colorbox (p. 90)**. You can use either of these to select a constant color from the current palette.

"palette z" maps the z value of each plot segment or plot element into the cbrange mapping of the palette. This allows smoothly-varying color along a 3d line or surface. It also allows coloring 2D plots by palette values read from an extra column of data (not all 2D plot styles allow an extra column).

16.1.1 Rgbcolor variable

Most plot commands assign a single color (linetype) to each element of the plot. If there are multiple plots on a single graph, the default color (linetype) is incremented sequentially. You can instead assign a separate color for each data point, line segment, or label based on additional information in the input data file. This is indicated by the colorspec keyword **variable**.

lc rgbcolor variable tells the program to use a 24-bit RGB color from a separate column in the data file. This requires a corresponding additional column in the **using** specifier. The extra column is interpreted as a 24-bit packed RGB triple. These are most easily specified as hexidecimal values (see **rgbcolor (p. 29)**). Text colors are similarly set using **tc rgbcolor variable**.

Example:

```
# Place colored points in 3D at the x,y,z coordinates corresponding to
# their red, green, and blue components
rgb(r,g,b) = 65536 * int(r) + 256 * int(g) + int(b)
splot "data" using 1:2:3:(rgb($1,$2,$3)) with points lc rgb variable
```

16.1.2 Linecolor variable

Most plot commands assign a single color (linetype) to each element of the plot. If there are multiple plots on a single graph, the default color (linetype) is incremented sequentially. You can instead assign a separate color for each data point, line segment, or label based on additional information in the input data file. This is indicated by the colorspec keyword **variable**.

lc variable tells the program to use the value read from one column of the input data as a linestyle index, and use the color belonging to that linestyle. This requires a corresponding additional column in the **using** specifier. Text colors can be set similarly using **tc variable**.

A single data file may contain multiple sets of data, separated by two blank lines. Each of these separate sets is assigned an index value (see **index (p. 68)**) that can be retrieved via the using specifier column(-2). See **pseudocolumns (p. 73)**. All data in the file is drawn with the same color/linestyle/pointtype properties by default. The command **lc variable** can be used to assign different colors to each data set in the file by using the index value from pseudocolumn -2.

Examples:

```
# Use the third column of data to assign colors to individual points
plot 'data' using 1:2:3 with points lc variable

# Use the data set index to choose a linestyle color
plot 'data' using 1:2:(column(-2)) with lines lc variable
```

17 Mouse input

The **x11**, **pm**, **windows**, **ggi**, and **wxt** terminals allow interaction with the current plot using the mouse. They also support the definition of hotkeys to activate pre-defined functions by hitting a single key while the mouse focus is in the active plot window. It is even possible to combine mouse input with **batch** command scripts, by invoking the command **pause mouse** and then using the mouse variables returned by mouse clicking as parameters for subsequent scripted actions. See **bind (p. 31)** and **mouse variables (p. 32)**. See also the command **set mouse (p. 109)**.

17.1 Bind

Syntax:

```
bind {allwindows} [<key-sequence>] ["<gnuplot commands>"]
bind <key-sequence> ""
reset bind
```

The **bind** allows defining or redefining a hotkey, i.e. a sequence of gnuplot commands which will be executed when a certain key or key sequence is pressed while the driver's window has the input focus. Note that **bind** is only available if gnuplot was compiled with **mouse** support and it is used by all mouse-capable terminals. A user-specified binding supersedes any builtin bindings, except that <space> and 'q' cannot normally be rebound. For an exception, see **bind space (p. 32)**.

Mouse buttons cannot be rebound.

You get the list of all hotkeys by typing **show bind** or **bind** or by typing the hotkey 'h' in the graph window.

Key bindings are restored to their default state by **reset bind**.

Note that multikey-bindings with modifiers must be given in quotes.

Normally hotkeys are only recognized when the currently active plot window has focus. **bind allwindows <key> ...** (short form: **bind all <key> ...**) causes the binding for <key> to apply to all gnuplot plot windows, active or not. In this case gnuplot variable MOUSE_KEY_WINDOW is set to the ID of the originating window, and may be used by the bound command.

Examples:

- set bindings:

```
bind a "replot"
bind "ctrl-a" "plot x*x"
bind "ctrl-alt-a" 'print "great"'
bind Home "set view 60,30; replot"
bind all Home 'print "This is window ",MOUSE_KEY_WINDOW'
```

- show bindings:

```
bind "ctrl-a"          # shows the binding for ctrl-a
bind                   # shows all bindings
show bind              # show all bindings
```

- remove bindings:

```
bind "ctrl-alt-a" ""   # removes binding for ctrl-alt-a
                         (note that builtins cannot be removed)
reset bind             # installs default (builtin) bindings
bind!                  # deprecated form of "reset bind"
```

- bind a key to toggle something:

```
v=0
bind "ctrl-r" "v=v+1;if(v%2)set term x11 noraise; else set term x11 raise"
```

Modifiers (ctrl / alt) are case insensitive, keys not:

```
ctrl-alt-a == CtRl-alT-a
ctrl-alt-a != ctrl-alt-A
```

List of modifiers (alt == meta):

```
ctrl, alt
```

List of supported special keys:

```
"BackSpace", "Tab", "Linefeed", "Clear", "Return", "Pause", "Scroll_Lock",
"Sys_Req", "Escape", "Delete", "Home", "Left", "Up", "Right", "Down",
"PageUp", "PageDown", "End", "Begin",

"KP_Space", "KP_Tab", "KP_Enter", "KP_F1", "KP_F2", "KP_F3", "KP_F4",
"KP_Home", "KP_Left", "KP_Up", "KP_Right", "KP_Down", "KP_PageUp",
"KP_PageDown", "KP_End", "KP_Begin", "KP_Insert", "KP_Delete", "KP_Equal",
"KP_Multiply", "KP_Add", "KP_Separator", "KP_Subtract", "KP_Decimal",
"KP_Divide",

"KP_1" - "KP_9", "F1" - "F12"
```

The following are window events rather than actual keys

```
"Close"
```

See also help for **mouse (p. 109)** and **if (p. 59)**.

17.1.1 Bind space

If gnuplot was built with configuration option –enable-raise-console, then typing <space> in the plot window raises gnuplot's command window. This hotkey can be changed to ctrl-space by starting gnuplot as 'gnuplot -ctrlq', or by setting the XResource 'gnuplot*ctrlq'. See **x11 command-line-options (p. 194)**.

17.2 Mouse variables

When **mousing** is active, clicking in the active window will set several user variables that can be accessed from the gnuplot command line. The coordinates of the mouse at the time of the click are stored in MOUSE_X MOUSE_Y MOUSE_X2 and MOUSE_Y2. The mouse button clicked, and any meta-keys active at that time, are stored in MOUSE_BUTTON MOUSE_SHIFT MOUSE_ALT and MOUSE_CTRL. These variables are set to undefined at the start of every plot, and only become defined in the event of a mouse click in the active plot window. To determine from a script if the mouse has been clicked in the active plot window, it is sufficient to test for any one of these variables being defined.

```
plot 'something'
pause mouse
if (defined(MOUSE_BUTTON)) call 'something_else'; \
else print "No mouse click."
```

It is also possible to track keystrokes in the plot window using the mousing code.

```
plot 'something'
pause mouse keypress
print "Keystroke ", MOUSE_KEY, " at ", MOUSE_X, " ", MOUSE_Y
```

When **pause mouse keypress** is terminated by a keypress, then MOUSE_KEY will contain the ascii character value of the key that was pressed. MOUSE_CHAR will contain the character itself as a string variable. If the pause command is terminated abnormally (e.g. by ctrl-C or by externally closing the plot window) then MOUSE_KEY will equal -1.

Note that after a zoom by mouse, you can read the new ranges as GPVAL_X_MIN, GPVAL_X_MAX, GPVAL_Y_MIN, and GPVAL_Y_MAX, see **gnuplot-defined variables (p. 26)**.

18 Plotting

There are three **gnuplot** commands which actually create a plot: **plot**, **splot** and **replot**. **plot** generates 2D plots, **splot** generates 3-d plots (actually 2D projections, of course), and **replot** appends its arguments to the previous **plot** or **splot** and executes the modified command.

Much of the general information about plotting can be found in the discussion of **plot**; information specific to 3D can be found in the **splot** section.

plot operates in either rectangular or polar coordinates – see **set polar (p. 124)** for details of the latter. **splot** operates only in rectangular coordinates, but the **set mapping** command allows for a few other coordinate systems to be treated. In addition, the **using** option allows both **plot** and **splot** to treat almost any coordinate system you'd care to define.

plot also lets you use each of the four borders – x (bottom), x2 (top), y (left) and y2 (right) – as an independent axis. The **axes** option lets you choose which pair of axes a given function or data set is plotted against. A full complement of **set** commands exists to give you complete control over the scales and labelling of each axis. Some commands have the name of an axis built into their names, such as **set xlabel**. Other commands have one or more axis names as options, such as **set logscale xy**. Commands and options controlling the z axis have no effect on 2D graphs.

splot can plot surfaces and contours in addition to points and/or lines. In addition to **splot**, see **set isosamples (p. 102)** for information about defining the grid for a 3D function; **splot datafile (p. 145)** for information about the requisite file structure for 3D data values; and **set contour (p. 91)** and **set cntrparam (p. 89)** for information about contours.

In **splot**, control over the scales and labels of the axes are the same as with **plot**, except that commands and options controlling the x2 and y2 axes have no effect whereas of course those controlling the z axis do take effect.

19 Start-up

When **gnuplot** is run, it looks for an initialization file to load. This file is called **.gnuplot** on Unix and AmigaOS systems, and **GNUPLOT.INI** on other systems. If this file is not found in the current directory, the program will look for it in the HOME directory (under AmigaOS, MS-DOS, Windows and OS/2, the environment variable **GNUPLOT** should contain the name of this directory; on Windows NT, it will use **USERPROFILE** if GNUPLOT isn't defined). Note: if NOCWDRC is defined during the installation, **gnuplot** will not read from the current directory.

If the initialization file is found, **gnuplot** executes the commands in it. These may be any legal **gnuplot** commands, but typically they are limited to setting the terminal and defining frequently-used functions or variables.

20 String constants and string variables

In addition to string constants, most gnuplot commands also accept a string variable, a string expression, or a function that returns a string. For example, the following four methods of creating a plot all result in the same plot title:

```
four = "4"
graph4 = "Title for plot #4"
graph(n) = sprintf("Title for plot #%d",n)

plot 'data.4' title "Title for plot #4"
plot 'data.4' title graph4
plot 'data.4' title "Title for plot #".four
plot 'data.4' title graph(4)
```

Since integers are promoted to strings when operated on by the string concatenation operator, the following method also works:

```
N = 4
plot 'data.'.N title "Title for plot #".N
```

In general, elements on the command line will only be evaluated as possible string variables if they are not otherwise recognizable as part of the normal gnuplot syntax. So the following sequence of commands is legal, although probably should be avoided so as not to cause confusion:

```
plot = "my_datafile.dat"
title = "My Title"
plot plot title title
```

There are three binary operators that require string operands: the string concatenation operator ".", the string equality operator "eq" and the string inequality operator "ne". The following example will print TRUE.

```
if ("A"."B" eq "AB") print "TRUE"
```

See also the two string formatting functions **gprintf (p. 98)** and **sprintf (p. 23)**.

Substrings can be specified by appending a range specifier to any string, string variable, or string-valued function. The range specifier has the form [begin:end], where begin is the index of the first character of the substring and end is the index of the last character of the substring. The first character has index 1. The begin or end fields may be empty, or contain '*', to indicate the true start or end of the original string. E.g. str[:] and str[*:*] both describe the full string str.

21 Substitution and Command line macros

When a command line to gnuplot is first read, i.e. before it is interpreted or executed, two forms of lexical substitution are performed. These are triggered by the presence of text in backquotes (ascii character 96) or preceded by @ (ascii character 64).

21.1 Substitution of system commands in backquotes

Command-line substitution is specified by a system command enclosed in backquotes. This command is spawned and the output it produces replaces the backquoted text on the command line. Some implementations also support pipes; see **plot datafile special-filenames (p. 70)**.

Command-line substitution can be used anywhere on the **gnuplot** command line, except inside strings delimited by single quotes.

Example:

This will run the program **leastsq** and replace **leastsq** (including backquotes) on the command line with its output:

```
f(x) = ‘leastsq‘
```

or, in VMS

```
f(x) = ‘run leastsq‘
```

These will generate labels with the current time and userid:

```
set label "generated on ‘date +%Y-%m-%d‘ by ‘whoami‘" at 1,1
set timestamp "generated on %Y-%m-%d by ‘whoami‘"
```

21.2 Substitution of string variables as macros

Substitution of command line macros is disabled by default, but may be enabled using the **set macros** command. If macro substitution is enabled, the character @ is used to trigger substitution of the current value of a string variable into the command line. The text in the string variable may contain any number of lexical elements. This allows string variables to be used as command line macros. Only string constants may be expanded using this mechanism, not string-valued expressions. For example:

```
set macros
style1 = "lines lt 4 lw 2"
style2 = "points lt 3 pt 5 ps 2"
range1 = "using 1:3"
range2 = "using 1:5"
plot "foo" @range1 with @style1, "bar" @range2 with @style2
```

The line containing @ symbols is expanded on input, so that by the time it is executed the effect is identical to having typed in full

```
plot "foo" using 1:3 with lines lt 4 lw 2, \
     "bar" using 1:5 with points lt 3 pt 5 ps 2
```

The function exists() may be useful in connection with macro evaluation. The following example checks that C can safely be expanded as the name of a user-defined variable:

```
C = "pi"
if (exists(C)) print C," = ", @C
```

Macro expansion does not occur inside either single or double quotes. However macro expansion does occur inside backquotes.

For execution of complete commands the **evaluate** command may also be handy.

21.3 String variables, macros, and command line substitution

The interaction of string variables, backquotes and macro substitution is somewhat complicated. Backquotes do not block macro substitution, so

```
filename = "mydata.inp"
lines = ` wc --lines @filename | sed "s/ .*//" `
```

results in the number of lines in mydata.inp being stored in the integer variable lines. And double quotes do not block backquote substitution, so

```
mycomputer = "`uname -n`"
```

results in the string returned by the system command **uname -n** being stored in the string variable mycomputer.

However, macro substitution is not performed inside double quotes, so you cannot define a system command as a macro and then use both macro and backquote substitution at the same time.

```
 machine_id = "uname -n"
 mycomputer = "`@machine_id`"  # doesn't work!!
```

This fails because the double quotes prevent @machine_id from being interpreted as a macro. To store a system command as a macro and execute it later you must instead include the backquotes as part of the macro itself. This is accomplished by defining the macro as shown below. Notice that the sprintf format nests all three types of quotes.

```
machine_id = sprintf('"`uname -n`"')
mycomputer = @machine_id
```

22 Syntax

Version 4 of gnuplot is much less sensitive than earlier versions to the order of keywords and suboptions. However, if you get error messages from specifying options that you think should work, please try rearranging them into the exact order listed by the documentation.

Options and any accompanying parameters are separated by spaces whereas lists and coordinates are separated by commas. Ranges are separated by colons and enclosed in brackets [], text and file names are enclosed in quotes, and a few miscellaneous things are enclosed in parentheses. Braces {} are used for a few special purposes.

Commas are used to separate coordinates on the **set** commands **arrow**, **key**, and **label**; the list of variables being fitted (the list after the **via** keyword on the **fit** command); lists of discrete contours or the loop parameters which specify them on the **set cntrparam** command; the arguments of the **set** commands **dgrid3d**, **dummy**, **isosamples**, **offsets**, **origin**, **samples**, **size**, **time**, and **view**; lists of tics or the loop parameters which specify them; the offsets for titles and axis labels; parametric functions to be used to calculate the x, y, and z coordinates on the **plot**, **replot** and **splot** commands; and the complete sets of keywords specifying individual plots (data sets or functions) on the **plot**, **replot** and **splot** commands.

Parentheses are used to delimit sets of explicit tics (as opposed to loop parameters) and to indicate computations in the **using** filter of the **fit**, **plot**, **replot** and **splot** commands.

(Parentheses and commas are also used as usual in function notation.)

Square brackets are used to delimit ranges given in **set**, **plot** or **splot** commands.

Colons are used to separate extrema in **range** specifications (whether they are given on **set**, **plot** or **splot** commands) and to separate entries in the **using** filter of the **plot**, **replot**, **splot** and **fit** commands.

Semicolons are used to separate commands given on a single command line.

Braces are used in text to be specially processed by some terminals, like **postscript**. They are also used to denote complex numbers: {3,2} = 3 + 2i.

At present you should not embed \n inside {} when using the PostScript terminal in **enhanced text** mode.

The EEPIC, Imagen, Uniplex, LaTeX, and TPIC drivers allow a newline to be specified by \\ in a single-quoted string or \\\\ in a double-quoted string.

22.1 Quote Marks

Gnuplot uses three forms of quote marks for delimiting text strings, double-quote (ascii 34), single-quote (ascii 39), and backquote (ascii 96).

Filenames may be entered with either single- or double-quotes. In this manual the command examples generally single-quote filenames and double-quote other string tokens for clarity.

String constants and text strings used for labels, titles, or other plot elements may be enclosed in either single quotes or double quotes. Further processing of the quoted text depends on the choice of quote marks.

Backslash processing of special characters like \n (newline) and \345 (octal character code) is performed for double-quoted strings. In single-quoted strings, backslashes are just ordinary characters. To get a single-quote (ascii 39) in a single-quoted string, it has to be doubled. Thus the strings "d\" s' b\\" and 'd" s'' b\' are completely equivalent.

Text justification is the same for each line of a multi-line string. Thus the center-justified string

```
"This is the first line of text.\nThis is the second line."
```

will produce

```
This is the first line of text.
   This is the second line.
```

but

```
'This is the first line of text.\nThis is the second line.'
```

will produce

```
This is the first line of text.\nThis is the second line.
```

Enhanced text processing is performed for both double-quoted text and single-quoted text, but only by terminals supporting this mode. See **enhanced text (p. 20)**.

Back-quotes are used to enclose system commands for substitution into the command line. See **substitution (p. 34)**.

23 Time/Date data

gnuplot supports the use of time and/or date information as input data. This feature is activated by the commands **set xdata time**, **set ydata time**, etc.

Internally all times and dates are converted to the number of seconds from the year 2000. The command **set timefmt** defines the format for all inputs: data files, ranges, tics, label positions — in short, anything that accepts a data value must receive it in this format. Since only one input format can be in force at a given time, all time/date quantities being input at the same time must be presented in the same format. Thus if both x and y data in a file are time/date, they must be in the same format.

The conversion to and from seconds assumes Universal Time (which is the same as Greenwich Standard Time). There is no provision for changing the time zone or for daylight savings. If all your data refer to the same time zone (and are all either daylight or standard) you don't need to worry about these things. But if the absolute time is crucial for your application, you'll need to convert to UT yourself.

Commands like **show xrange** will re-interpret the integer according to **timefmt**. If you change **timefmt**, and then **show** the quantity again, it will be displayed in the new **timefmt**. For that matter, if you give the deactivation command (like **set xdata**), the quantity will be shown in its numerical form.

The commands **set format** or **set tics format** define the format that will be used for tic labels, whether or not the specified axis is time/date.

If time/date information is to be plotted from a file, the **using** option *must* be used on the **plot** or **splot** command. These commands simply use white space to separate columns, but white space may be embedded within the time/date string. If you use tabs as a separator, some trial-and-error may be necessary to discover how your system treats them.

The following example demonstrates time/date plotting.

Suppose the file "data" contains records like

```
03/21/95 10:00  6.02e23
```

This file can be plotted by

```
set xdata time
set timefmt "%m/%d/%y"
set xrange ["03/21/95":"03/22/95"]
set format x "%m/%d"
set timefmt "%m/%d/%y %H:%M"
plot "data" using 1:3
```

which will produce xtic labels that look like "03/21".

See the descriptions of each command for more details.

Part II

Plotting styles

There are many plotting styles available in gnuplot. They are listed alphabetically below. The commands **set style data** and **set style function** change the default plotting style for subsequent **plot** and **splot** commands.

You also have the option to specify the plot style explicitly as part of the **plot** or **splot** command. If you want to mix plot styles within a single plot, you must specify the plot style for each component.

Example:

```
plot 'data' with boxes, sin(x) with lines
```

Each plot style has its own expected set of data entries in a data file. For example by default the **lines** style expects either a single column of y values (with implicit x ordering) or a pair of columns with x in the first and y in the second. For more information on how to fine-tune how columns in a file are interpreted as plot data, see **using (p. 71)**.

24 Boxerrorbars

The **boxerrorbars** style is only relevant to 2D data plotting. It is a combination of the **boxes** and **yerrorbars** styles. It uses 3, 4, or 5 columns of data:

```
3 columns:  x  y  ydelta
4 columns:  x  y  ydelta xdelta        # boxwidth != -2
4 columns:  x  y  ylow   yhigh         # boxwidth == -2
5 columns:  x  y  ylow   yhigh  xdelta
```

The boxwidth will come from the fourth column if the y errors are given as "ydelta" and the boxwidth was not previously set to -2.0 (**set boxwidth -2.0**) or from the fifth column if the y errors are in the form of "ylow yhigh". The special case **boxwidth = -2.0** is for four-column data with y errors in the form "ylow yhigh". In this case the boxwidth will be calculated so that each box touches the adjacent boxes. The width will also be calculated in cases where three-column data are used.

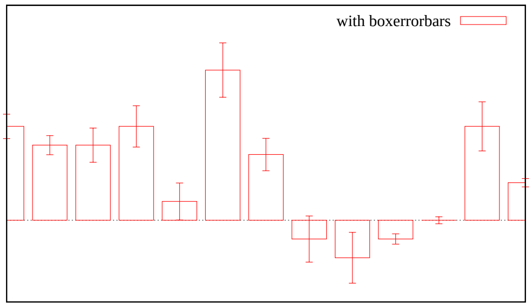

The box height is determined from the y error in the same way as it is for the **yerrorbars** style — either from y-ydelta to y+ydelta or from ylow to yhigh, depending on how many data columns are provided. See also

```
errorbar demo.
```

25 Boxes

The **boxes** style is only relevant to 2D plotting. It draws a box centered about the given x coordinate that extends from the x axis (not from the graph border) to the given y coordinate. It uses 2 or 3 columns of basic data. Additional input columns may be used to provide information such as variable line or fill color (see **rgbcolor variable (p. 30)**).

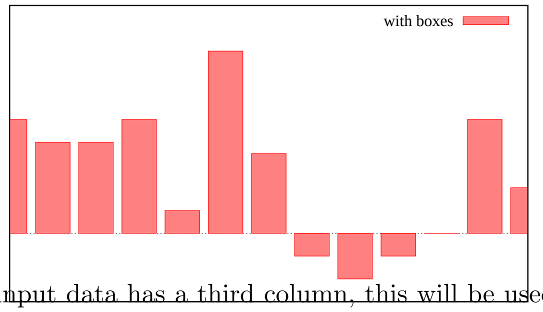


```
2 columns:  x  y
3 columns:  x  y  x_width
```

The width of the box is obtained in one of three ways. If the input data has a third column, this will be used to set the width of the box. If not, if a width has been set using the **set boxwidth** command, this will be used. If neither of these is available, the width of each box will be calculated automatically so that it touches the adjacent boxes.

The interior of the boxes is drawn according to the current fillstyle. See **set style fill (p. 127)** for details. Alternatively a new fillstyle may be specified in the plot command.

For fillstyle **empty** the box is not filled.

For fillstyle **solid** the box is filled with a solid rectangle of the current drawing color. There is an optional parameter <density> that controls the fill density; it runs from 0 (background color) to 1 (current drawing color).

For fillstyle **pattern** the box is filled in the current drawing color with a pattern, if supported by the terminal driver.

Examples:

To plot a data file with solid filled boxes with a small vertical space separating them (bargraph):

```
set boxwidth 0.9 relative
set style fill solid 1.0
plot 'file.dat' with boxes
```

To plot a sine and a cosine curve in pattern-filled boxes style:

```
set style fill pattern
plot sin(x) with boxes, cos(x) with boxes
```

The sin plot will use pattern 0; the cos plot will use pattern 1. Any additional plots would cycle through the patterns supported by the terminal driver.

To specify explicit fillstyles for each dataset:

```
plot 'file1' with boxes fs solid 0.25, \
     'file2' with boxes fs solid 0.50, \
     'file3' with boxes fs solid 0.75, \
     'file4' with boxes fill pattern 1, \
     'file5' with boxes fill empty
```

26 Boxxyerrorbars

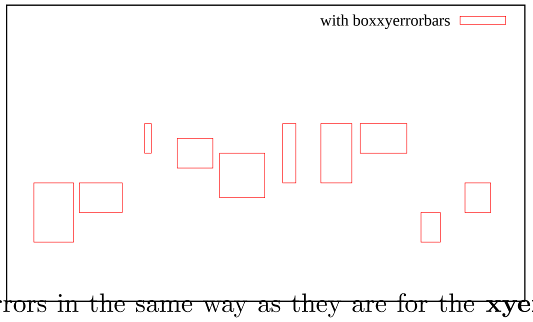

The **boxxyerrorbars** style is only relevant to 2D data plotting. It is similar to the **xyerrorbars** style except that it draws rectangular areas rather than simple crosses. It uses either 4 or 6 basic columns of input data. Additional input columns may be used to provide information such as variable line or fill color (see **rgbcolor variable (p. 30)**).

```
4 columns:  x  y  xdelta  ydelta
6 columns:  x  y  xlow  xhigh  ylow  yhigh
```

The box width and height are determined from the x and y errors in the same way as they are for the **xyerrorbars** style — either from xlow to xhigh and from ylow to yhigh, or from x-xdelta to x+xdelta and from y-ydelta to y+ydelta, depending on how many data columns are provided.

The interior of the boxes is drawn according to the current fillstyle. See **set style fill (p. 127)** and **boxes (p. 38)** for details. Alternatively a new fillstyle may be specified in the plot command.

27 Candlesticks

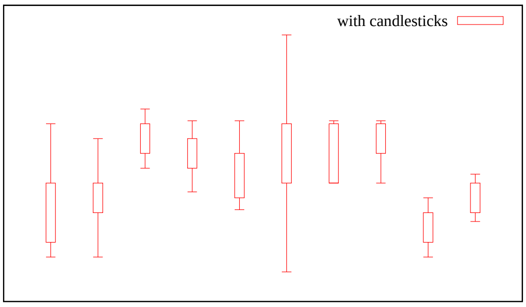

The **candlesticks** style can be used for 2D data plotting of financial data or for generating box-and-whisker plots of statistical data. The symbol is a rectangular box, centered horizontally at the x coordinate and limited vertically by the opening and closing prices. A vertical line segment at the x coordinate extends up from the top of the rectangle to the high price and another down to the low. The vertical line will be unchanged if the low and high prices are interchanged.

Five columns of basic data are required:

```
financial data:   date  open  low  high  close
whisker plot:     x  box_min  whisker_min  whisker_high  box_high
```

The width of the rectangle can be controlled by the **set boxwidth** command. For backwards compatibility with earlier gnuplot versions, when the boxwidth parameter has not been set then the width of the candlestick rectangle is controlled by **set bars <width>**.

By default the vertical line segments have no crossbars at the top and bottom. If you want crossbars, which are typically used for box-and-whisker plots, then add the keyword **whiskerbars** to the plot command. By default these whiskerbars extend the full horizontal width of the candlestick, but you can modify this by specifying a fraction of the full width.

The usual convention for financial data is that the rectangle is empty if (open < close) and solid fill if (close < open). This is the behavior you will get if the current fillstyle is set to "empty". See **fillstyle (p. 127)**. If you set the fillstyle to solid or pattern, then this will be used for all boxes independent of open and close values. See also **set bars (p. 86)** and **financebars (p. 41)**. See also the

```
candlestick
```

and

```
finance
```

demos.

Note: To place additional symbols, such as the median value, on a box-and-whisker plot requires additional plot commands as in this example:

```
# Data columns:X Min 1stQuartile Median 3rdQuartile Max
set bars 4.0
set style fill empty
plot 'stat.dat' using 1:3:2:6:5 with candlesticks title 'Quartiles', \
     ''         using 1:4:4:4:4 with candlesticks lt -1 notitle
```

```
# Plot with crossbars on the whiskers, crossbars are 50% of full width
plot 'stat.dat' using 1:3:2:6:5 with candlesticks whiskerbars 0.5
```

See **set boxwidth (p. 87)**, **set bars (p. 86)** and **set style fill (p. 127)**.

28 Circles

The **circles** style plots a circle with an explicit radius at each data point. Three columns of data are required: x, y, radius. An optional 4th column may be used to specify color information. The radius is always interpreted in the units of the plot's horizontal axis (x or x2). The scale on y and the aspect ratio of the plot are both ignored.

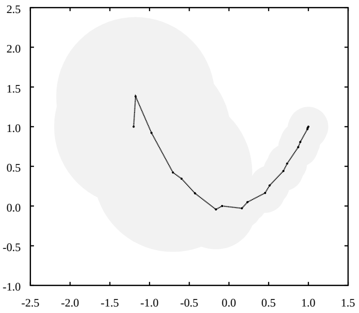

Example (draws circles whose area is proportional to the value in column 3):

```
set style fill transparent solid 0.2 noborder
plot 'data' using 1:2:(sqrt($3)) with circles, \
     'data' using 1:2 with linespoints
```

The result is similar to using a **points** plot with variable size points and pointstyle 6, except that the circles will scale with the x axis range. See also **set object circle (p. 114)** and **fillstyle (p. 127)**.

29 Dots

The **dots** style plots a tiny dot at each point; this is useful for scatter plots with many points. Either 1 or 2 columns of input data are required in 2D. Three columns are required in 3D.

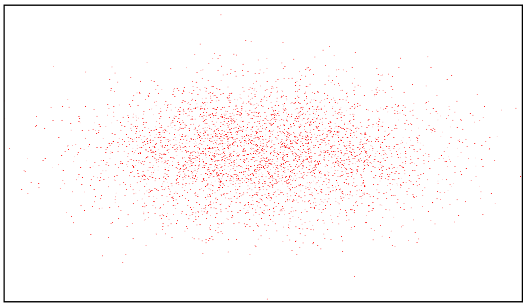

For some terminals (post, pdf) the size of the dot can be controlled by changing the linewidth.

```
1 column    y          # x is row number
2 columns:  x  y
3 columns:  x  y  z    # 3D only (splot)
```

30 Filledcurves

The **filledcurves** style is only relevant to 2D plotting. Three variants are possible. The first two variants require either a function or two columns of input data, and may be further modified by the options listed below.

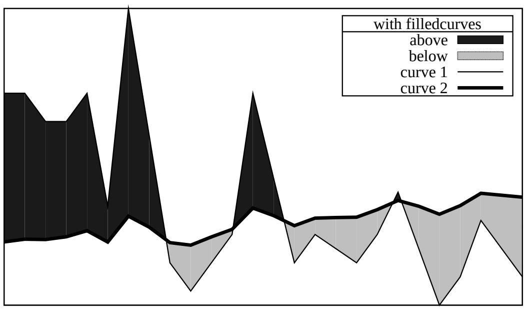

Syntax:

```
plot ... with filledcurves [option]
```

where the option can be one of the following

```
[closed | {above | below}
{x1 | x2 | y1 | y2}[=<a>] | xy=<x>,<y>]
```

The first variant, **closed**, treats the curve itself as a closed polygon. This is the default if there are two columns of input data.

The second variant is to fill the area between the curve and a given axis, a horizontal or vertical line, or a point.

```
filledcurves closed   ... just filled closed curve,
filledcurves x1       ... x1 axis,
filledcurves x2       ... x2 axis, etc for y1 and y2 axes,
filledcurves y1=0     ... line y=0 (at y1 axis) ie parallel to x1 axis,
filledcurves y2=42    ... line y=42 (at y2 axis) ie parallel to x2, etc,
filledcurves xy=10,20 ... point 10,20 of x1,y1 axes (arc-like shape).
```

The third variant requires three columns of input data: the x coordinate and two y coordinates corresponding to two curves sampled at the same set of x coordinates; the area between the two curves is filled. This is the default if there are three or more columns of input data.

```
3 columns:  x  y1  y2
```

Example of filling the area between two input curves.

fill between curves demo.

```
plot 'data' using 1:2:3 with filledcurves
```

The **above** and **below** options apply both to commands of the form

```
... filledcurves above {x1|x2|y1|y2}=<val>
```

and to commands of the form

```
... using 1:2:3 with filledcurves below
```

In either case the option limits the filled area to one side of the bounding line or curve.

Note: Not all terminal types support this plotting mode.

Zooming a filled curve drawn from a datafile may produce empty or incorrect areas because gnuplot is clipping points and lines, and not areas.

If the values of <a>, <x>, <y> are out of the drawing boundary, then they are moved to the graph boundary. Then the actually filled area in the case of option xy=<x>,<y> will depend on xrange and yrange.

31 Financebars

The **financebars** style is only relevant for 2D data plotting of financial data. It requires 1 x coordinate (usually a date) and 4 y values (prices).

```
5 columns:   date  open  low  high  close
```

The symbol is a vertical line segment, located horizontally at the x coordinate and limited vertically by the high and low prices. A horizontal tic on the left marks the opening price and one on the right marks the closing price. The length of these tics may be changed by **set bars**. The symbol will be unchanged if the high and low prices are interchanged. See **set bars (p. 86)** and **candlesticks (p. 39)**, and also the

finance demo.

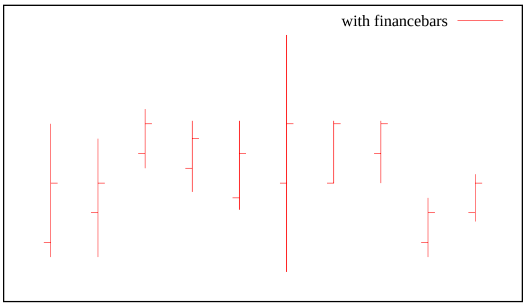

32 Fsteps

The **fsteps** style is only relevant to 2D plotting. It connects consecutive points with two line segments: the first from (x1,y1) to (x1,y2) and the second from (x1,y2) to (x2,y2). The input column requires are the same as for plot styles **lines** and **points**. The difference between **fsteps** and **steps** is that **fsteps** traces first the change in y and then the change in x. **steps** traces first the change in x and then the change in y.

See also

steps demo.

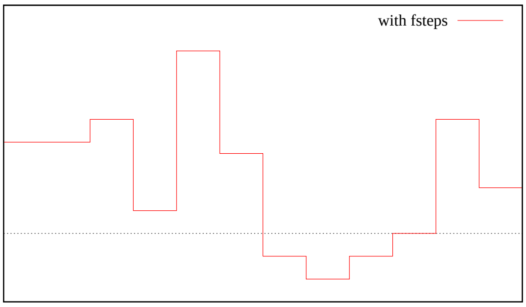

33 Histeps

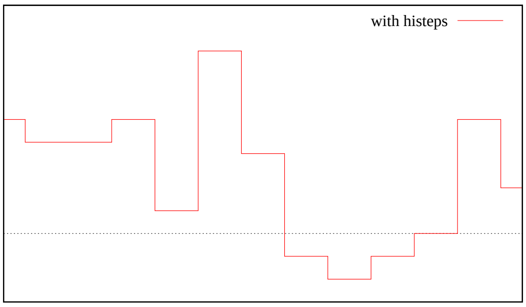

The **histeps** style is only relevant to 2D plotting. It is intended for plotting histograms. Y-values are assumed to be centered at the x-values; the point at x1 is represented as a horizontal line from ((x0+x1)/2,y1) to ((x1+x2)/2,y1). The lines representing the end points are extended so that the step is centered on at x. Adjacent points are connected by a vertical line at their average x, that is, from ((x1+x2)/2,y1) to ((x1+x2)/2,y2). The input column requires are the same as for plot styles **lines** and **points**.

If **autoscale** is in effect, it selects the xrange from the data rather than the steps, so the end points will appear only half as wide as the others. See also

```
steps demo.
```

histeps is only a plotting style; **gnuplot** does not have the ability to create bins and determine their population from some data set.

34 Histograms

The **histograms** style is only relevant to 2D plotting. It produces a bar chart from a sequence of parallel data columns. Each element of the **plot** command must specify a single input data source (e.g. one column of the input file), possibly with associated tic values or key titles. Four styles of histogram layout are currently supported.

```
set style histogram clustered {gap <gapsize>}
set style histogram errorbars {gap <gapsize>} {<linewidth>}
set style histogram rowstacked
set style histogram columnstacked
```

The default style corresponds to **set style histogram clustered gap 2**. In this style, each set of parallel data values is collected into a group of boxes clustered at the x-axis coordinate corresponding to their sequential position (row #) in the selected datafile columns. Thus if <n> datacolumns are selected, the first cluster is centered about x=1, and contains <n> boxes whose heights are taken from the first entry in the corresponding <n> data columns. This is followed by a gap and then a second cluster of boxes centered about x=2 corresponding to the second entry in the respective data columns, and so on. The default gap width of 2 indicates that the empty space between clusters is equivalent to the width of 2 boxes. All boxes derived from any one column are given the same fill color and/or pattern (see **set style fill (p. 127)**).

Each cluster of boxes is derived from a single row of the input data file. It is common in such input files that the first element of each row is a label. Labels from this column may be placed along the x-axis underneath the appropriate cluster of boxes with the **xticlabels** option to **using**.

The **errorbars** style is very similar to the **clustered** style, except that it requires additional columns of input for each entry. The first column holds the height (y value) of that box, exactly as for the **clustered** style.

```
2 columns:        y yerr          bar extends from y-yerr to y+err
3 columns:        y ymin yman     bar extends from ymin to ymax
```

The appearance of the error bars is controlled by the current value of **set bars** and by the optional <linewidth> specification.

Two styles of stacked histogram are supported, chosen by the command **set style histogram {rowstacked|columnstacked}**. In these styles the data values from the selected columns are collected into stacks of boxes. Positive values stack upwards from y=0; negative values stack downwards. Mixed positive and negative values will produce both an upward stack and a downward stack. The default stacking mode is **rowstacked**.

The **rowstacked** style places a box resting on the x-axis for each data value in the first selected column; the first data value results in a box a x=1, the second at x=2, and so on. Boxes corresponding to the second and subsequent data columns are layered on top of these, resulting in a stack of boxes at x=1 representing the first data value from each column, a stack of boxes at x=2 representing the second data value from each column, and so on. All boxes derived from any one column are given the same fill color and/or pattern (see **set style fill (p. 127)**).

The **columnstacked** style is similar, except that each stack of boxes is built up from a single data column. Each data value from the first specified column yields a box in the stack at x=1, each data value from the

second specified column yields a box in the stack at x=2, and so on. In this style the color of each box is taken from the row number, rather than the column number, of the corresponding data field.

Box widths may be modified using the **set boxwidth** command. Box fill styles may be set using the **set style fill** command.

Histograms always use the x1 axis, but may use either y1 or y2. If a plot contains both histograms and other plot styles, the non-histogram plot elements may use either the x1 or the x2 axis.

Examples:

Suppose that the input file contains data values in columns 2, 4, 6, ... and error estimates in columns 3, 5, 7, ... This example plots the values in columns 2 and 4 as a histogram of clustered boxes (the default style). Because we use iteration in the plot command, any number of data columns can be handled in a single command. See **iteration (p. 59)**.

```
set boxwidth 0.9 relative
set style data histograms
set style histogram cluster
set style fill solid 1.0 border lt -1
plot for [COL=2:4:2] 'file.dat' using COL
```

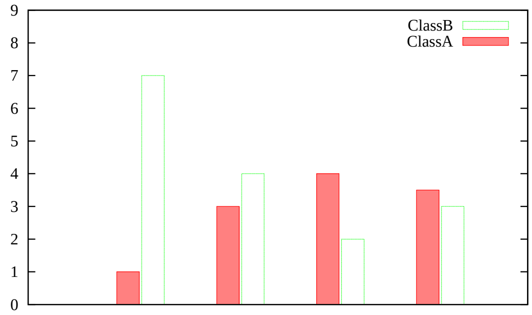

This will produce a plot with clusters of two boxes (vertical bars) centered at each integral value on the x axis. If the first column of the input file contains labels, they may be placed along the x-axis using the variant command

```
plot for [COL=2:4:2] 'file.dat' using COL:xticlabels(1)
```

If the file contains both magnitude and range information for each value, then error bars can be added to the plot. The following commands will add error bars extending from (y-<error>) to (y+<error>), capped by horizontal bar ends drawn the same width as the box itself. The error bars and bar ends are drawn with linewidth 2, using the border linetype from the current fill style.

```
set bars fullwidth
set style fill solid 1 border lt -1
set style histogram errorbars gap 2 lw 2
plot for [COL=2:4:2] 'file.dat' using COL:COL+1
```

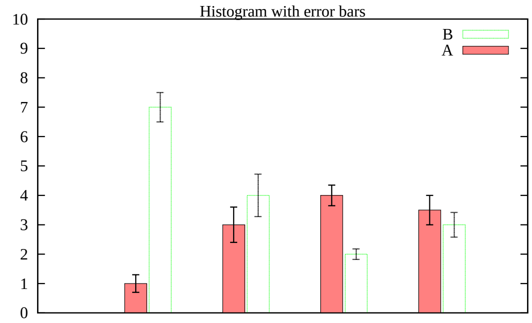

To plot the same data as a rowstacked histogram. Just to be different, this example lists the separate columns explicitly rather than using iteration.

```
set style histogram rowstacked
plot 'file.dat' using 2, '' using 4:xtic(1)
```

This will produce a plot in which each vertical bar corresponds to one row of data. Each vertical bar contains a stack of two segments, corresponding in height to the values found in columns 2 and 4 of the datafile.

Finally, the commands

```
set style histogram columnstacked
plot 'file.dat' using 2, '' using 4
```

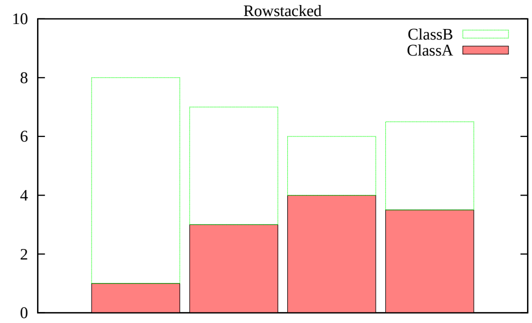

will produce two vertical stacks, one for each column of data. The stack at x=1 will contain a box for each entry in column 2 of the datafile. The stack at x=2 will contain a box for each parallel entry in column 4 of the datafile. Because this interchanges gnuplot's usual interpretation of input rows and columns, the specification of key titles and x-axis tic labels must also be modified accordingly. See the comments given below.

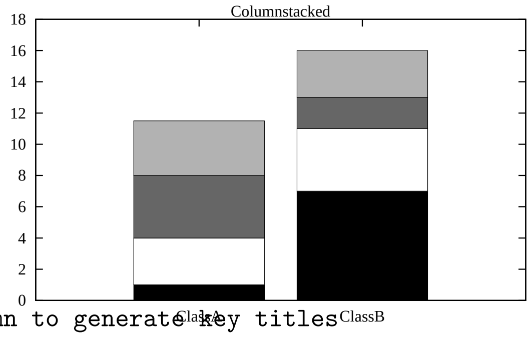


```
set style histogram columnstacked
plot '' u 5:key(1)            # uses first column to generate key titles
plot '' u 5 title columnhead  # uses first row to generate xtic labels
```

Note that the two examples just given present exactly the same data values, but in different formats.

34.1 Newhistogram

Syntax:

```
newhistogram {"<title>"} {<linetype>} {<fillstyle>} {at <x-coord>}
```

More than one set of histograms can appear in a single plot. In this case you can force a gap between them, and a separate label for each set, by using the **newhistogram** command. For example

```
set style histogram  cluster
plot newhistogram "Set A", 'a' using 1, '' using 2, '' using 3, \
     newhistogram "Set B", 'b' using 1, '' using 2, '' using 3
```

The labels "Set A" and "Set B" will appear beneath the respective sets of histograms, under the overall x axis label.

The newhistogram command can also be used to force histogram coloring to begin with a specific color (linetype). By default colors will continue to increment successively even across histogram boundaries. Here is an example using the same coloring for multiple histograms

```
plot newhistogram "Set A" lt 4, 'a' using 1, '' using 2, '' using 3, \
     newhistogram "Set B" lt 4, 'b' using 1, '' using 2, '' using 3
```

Similarly you can force the next histogram to begin with a specified fillstyle. If the fillstyle is set to **pattern**, then the pattern used for filling will be incremented automatically.

The **at** **<x-coord>** option only applies to column-stacked histograms.

34.2 Automated iteration over multiple columns

If you want to create a histogram from many columns of data in a single file, it is very convenient to use the plot iteration feature. See **iteration (p. 59)**. For example, to create stacked histograms of the data in columns 3 through 8

```
set style histogram columnstacked
plot for [i=3:8] "datafile" using i title columnhead
```

35 Image

The **image**, **rgbimage**, and **rgbalpha** plotting styles all project a uniformly sampled grid of data values onto a plane in either a 2D or 3D. The input data may be an actual bitmapped image, perhaps converted from a standard format such as PNG, or a simple array of numerical values.

This figure illustrates generation of a heat map from an array of scalar values. The current palette is used to map each value onto the color assigned to the corresponding pixel.

```
plot '-' matrix with image
5 4 3 1 0
2 2 0 0 1
0 0 0 1 0
0 1 2 4 3
e
e
```

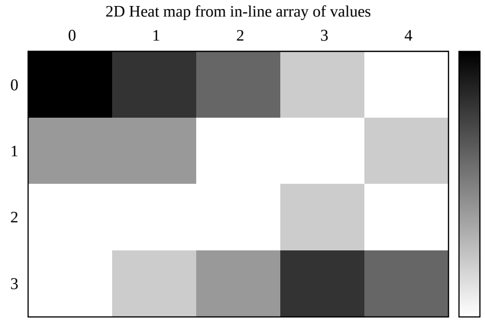

Each pixel (data point) of the input 2D image will become a rectangle or parallelipiped in the plot. The coordinates of each data point will determine the center of the parallelipiped. That is, an M x N set of data will form an image with M x N pixels. This is different from the pm3d plotting style, where an M x N set of data will form a surface of (M-1) x (N-1) elements. The scan directions for a binary image data grid can be further controlled by additional keywords. See **binary general keywords flipx (p. 66)**, **center (p. 66)**, and **rotate (p. 66)**.

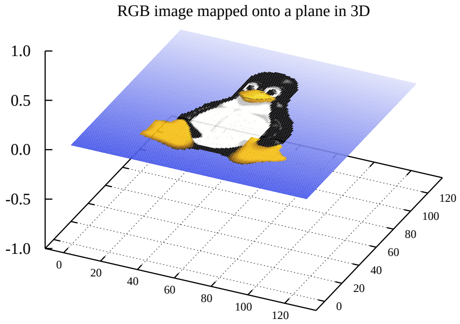

Image data can be scaled to fill a particular rectangle within a 2D plot coordinate system by specifying the x and y extent of each pixel. See **binary general keywords dx (p. 66)** and **dy (p. 66)**. To generate the figure at the right, the same input image was placed multiple times, each with a specified dx, dy, and origin. The input PNG image of a building is 50x128 pixels. The tall building was drawn by mapping this using **dx=0.5 dy=1.5**. The short building used a mapping **dx=0.5 dy=0.35**.

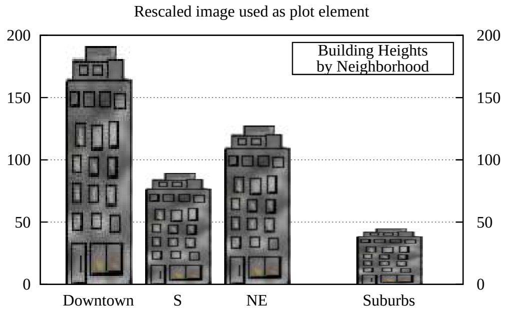

The **image** style handles input pixels containing a grayscale or color palette value. Thus 2D plots (**plot** command) require 3 columns of data (x,y,value), while 3D plots (**splot** command) require 4 columns of data (x,y,z,value).

The **rgbimage** style handles input pixels that are described by three separate values for the red, green, and blue components. Thus 5D data (x,y,r,g,b) is needed for **plot** and 6D data (x,y,z,r,g,b) for **splot**. The individual red, green, and blue components are assumed to lie in the range [0:255].

The **rgbalpha** style handles input pixels that contain alpha channel (transparency) information in addition to the red, green, and blue components. Thus 6D data (x,y,r,g,b,a) is needed for **plot** and 7D data (x,y,z,r,g,b,a) for **splot**. The r, g, b, and alpha components are assumed to lie in the range [0:255].

35.1 Transparency

The **rgbalpha** plotting style assumes that each pixel of input data contains an alpha value in the range [0:255]. A pixel with alpha = 0 is purely transparent and does not alter the underlying contents of the plot. A pixel with alpha = 255 is purely opaque. All terminal types can handle these two extreme cases. A pixel with 0 < alpha < 255 is partially transparent. Only a few terminal types can handle this correctly; other terminals will approximate this by treating alpha as being either 0 or 255.

35.2 Image failsafe

Some terminal drivers provide code to optimize rendering of image data within a rectangular 2D area. However this code is known to be imperfect. This optimized code may be disabled by using the keyword **failsafe**. E.g.

```
plot 'data' with image failsafe
```

36 Impulses

The **impulses** style displays a vertical line from the x axis to the y value of each point (2D) or from the xy plane to the z value of each point (3D). Note that the y or z values may be negative. As with most plot styles, data from additional columns can be used to control the color of each impulse. To use this style effectively in 3D plots, it is useful to use thick lines (linewidth > 1). This approximates a 3D bar chart.

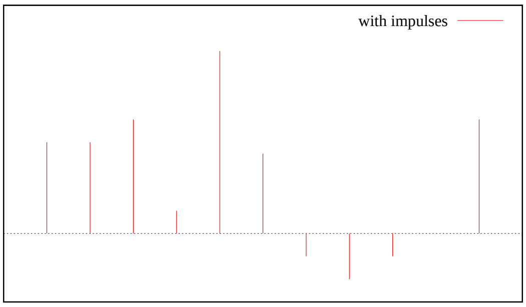


```
1 column:   y       # implicit x coordinate from row number (2D)
2 columns:  x  y    # line from [x,0] to [x,y]  (2D)
3 columns:  x  y  z # line from [x,y,0] to [x,y,z] (3D)
```

37 Labels

The **labels** style reads coordinates and text from a data file and places the text string at the corresponding 2D or 3D position. 3 or 4 input columns of basic data are required. Additional input columns may be used to provide information such as variable font size or text color (see **rgbcolor variable (p. 30)**).

```
3 columns:  x  y  string    # 2D version
4 columns:  x  y  z  string # 3D version
```

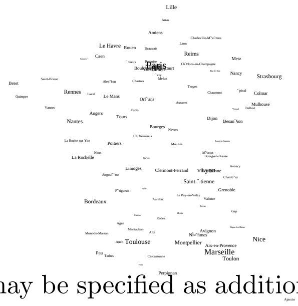

The font, color, rotation angle and other properties of the printed text may be specified as additional command options (see **set label (p. 105)**). The example below generates a 2D plot with text labels constructed from the city whose name is taken from column 1 of the input file, and whose geographic coordinates are in columns 4 and 5. The font size is calculated from the value in column 3, in this case the population.

```
CityName(String,Size) = sprintf("{/=%d %s}", Scale(Size), String)
plot 'cities.dat' using 5:4:(CityName(stringcolumn(1),$3)) with labels
```

If we did not want to adjust the font size to a different size for each city name, the command would be much simpler:

```
plot 'cities.dat' using 5:4:1 with labels font "Times,8"
```

The **labels** style can also be used in 3D plots. In this case four input column specifiers are required, corresponding to X Y Z and text.

```
splot 'datafile' using 1:2:3:4 with labels
```

See also **datastrings (p. 19)**, **set style data (p. 127)**.

38 Lines

The **lines** style connects adjacent points with straight line segments. It may be used in either 2D or 3D plots. The basic form requires 1, 2, or 3 columns of input data. Additional input columns may be used to provide information such as variable line color (see **rgbcolor variable (p. 30)**).

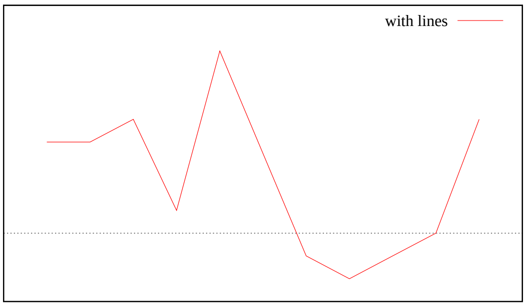

2D form

```
1 column:   y      # implicit x from row number
2 columns:  x  y
```

3D form

```
1 column:   z      # implicit x from row, y from index
3 columns:  x  y  z
```

See also **linetype (p. 29)**, **linewidth (p. 129)**, and **linestyle (p. 129)**.

39 Linespoints

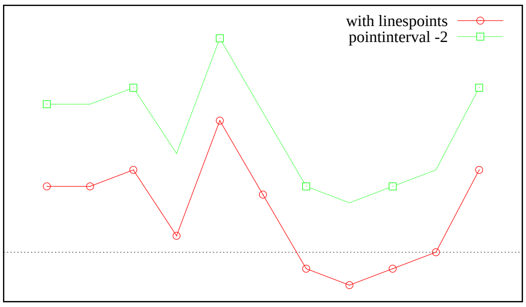

The **linespoints** style connects adjacent points with straight line segments and then goes back to draw a small symbol at each point. The command **set pointsize** may be used to change the default size of the points. 1 or 2 columns of basic input data are required in 2D plots; 1 or 3 columns are required if 3D plots. See **style lines (p. 46)**. Additional input columns may be used to provide information such as variable point size or line color.

The **pointinterval** (short form **pi**) property of the line style can be used to control whether or not every point in the plot is given a symbol. For example, 'with lp pi 3' will draw line segments through every data point, but will only place a symbol on every 3rd point. A negative value for **pointinterval** will erase the portion of line segment that passes underneath the symbol.

linespoints may be abbreviated **lp**.

40 Points

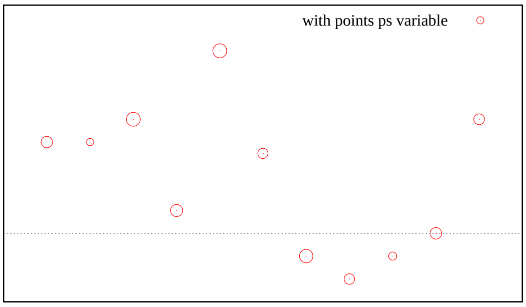

The **points** style displays a small symbol at each point. The command **set pointsize** may be used to change the default size of the points. 1 or 2 columns of basic input data are required in 2D plots; 1 or 3 columns are required if 3D plots. See **style lines (p. 46)**. Additional input columns may be used to provide information such as variable point size or line color.

41 Steps

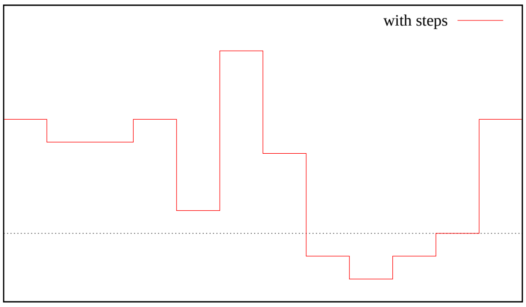

The **steps** style is only relevant to 2D plotting. It connects consecutive points with two line segments: the first from (x1,y1) to (x2,y1) and the second from (x2,y1) to (x2,y2). The input column requires are the same as for plot styles **lines** and **points**. The difference between **fsteps** and **steps** is that **fsteps** traces first the change in y and then the change in x. **steps** traces first the change in x and then the change in y. See also

```
steps demo.
```

42 Rgbalpha

See **image (p. 44)**.

43 Rgbimage

See **image (p. 44)**.

44 Vectors

The 2D **vectors** style draws a vector from (x,y) to (x+xdelta,y+ydelta). The 3D **vectors** style is similar, but requires six columns of basic data. A small arrowhead is drawn at the end of each vector.

```
4 columns:  x  y  xdelta  ydelta
6 columns:  x  y  z  xdelta  ydelta  zdelta
```

splot with vectors is supported only for **set mapping cartesian**. The keywords "with vectors" may be followed by arrow style specifications. See **arrowstyle (p. 126)** for more details.

Example:

```
plot 'file.dat' using 1:2:3:4 with vectors head filled lt 2
splot 'file.dat' using 1:2:3:(1):(1):(1) with vectors filled head lw 2
```

set clip one and **set clip two** affect vectors drawn in 2D. Please see **set clip (p. 88)** and **arrowstyle (p. 126)**.

45 Xerrorbars

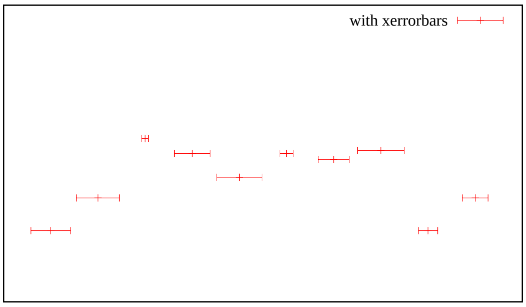

The **xerrorbars** style is only relevant to 2D data plots. **xerrorbars** is like **dots**, except that a horizontal error bar is also drawn. At each point (x,y), a line is drawn from (xlow,y) to (xhigh,y) or from (x-xdelta,y) to (x+xdelta,y), depending on how many data columns are provided. A tic mark is placed at the ends of the error bar (unless **set bars** is used — see **set bars (p. 86)** for details). The basic style requires either 3 or 4 columns:

```
3 columns:  x  y  xdelta
4 columns:  x  y  xlow  xhigh
```

46 Xyerrorbars

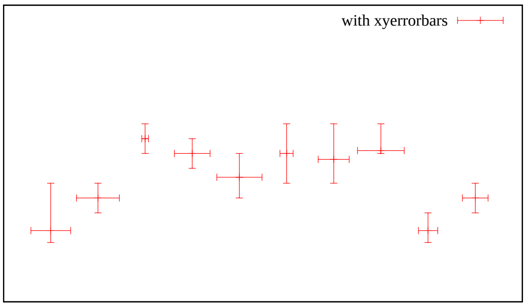

The **xyerrorbars** style is only relevant to 2D data plots. **xyerrorbars** is like **dots**, except that horizontal and vertical error bars are also drawn. At each point (x,y), lines are drawn from (x,y-ydelta) to (x,y+ydelta) and from (x-xdelta,y) to (x+xdelta,y) or from (x,ylow) to (x,yhigh) and from (xlow,y) to (xhigh,y), depending upon the number of data columns provided. A tic mark is placed at the ends of the error bar (unless **set bars** is used — see **set bars (p. 86)** for details). Either 4 or 6 input columns are required.

```
4 columns:  x  y  xdelta  ydelta
6 columns:  x  y  xlow  xhigh  ylow  yhigh
```

If data are provided in an unsupported mixed form, the **using** filter on the **plot** command should be used to set up the appropriate form. For example, if the data are of the form (x,y,xdelta,ylow,yhigh), then you can use

```
plot 'data' using 1:2:($1-$3):($1+$3):4:5 with xyerrorbars
```

47 Yerrorbars

The **yerrorbars** (or **errorbars**) style is only relevant to 2D data plots. **yerrorbars** is like **points**, except that a vertical error bar is also drawn. At each point (x,y), a line is drawn from (x,y-ydelta) to (x,y+ydelta) or from (x,ylow) to (x,yhigh), depending on how many data columns are provided. A tic mark is placed at the ends of the error bar (unless **set bars** is used — see **set bars (p. 86)** for details). Either 3 or 4 input columns are required.

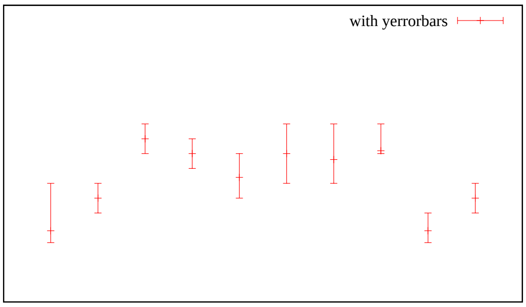


```
3 columns:  x  y  ydelta
4 columns:  x  y  ylow  yhigh
```

See also

errorbar demo.

48 Xerrorlines

The **xerrorlines** style is only relevant to 2D data plots. **xerrorlines** is like **linespoints**, except that a horizontal error line is also drawn. At each point (x,y), a line is drawn from (xlow,y) to (xhigh,y) or from (x-xdelta,y) to (x+xdelta,y), depending on how many data columns are provided. A tic mark is placed at the ends of the error bar (unless **set bars** is used — see **set bars (p. 86)** for details). The basic style requires either 3 or 4 columns:

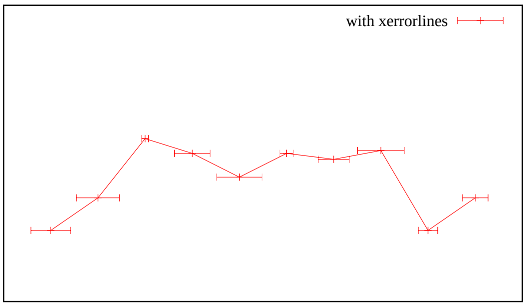


```
3 columns:  x  y  xdelta
4 columns:  x  y  xlow  xhigh
```

49 Xyerrorlines

The **xyerrorlines** style is only relevant to 2D data plots. **xyerrorlines** is like **linespoints**, except that horizontal and vertical error bars are also drawn. At each point (x,y), lines are drawn from (x,y-ydelta) to (x,y+ydelta) and from (x-xdelta,y) to (x+xdelta,y) or from (x,ylow) to (x,yhigh) and from (xlow,y) to (xhigh,y), depending upon the number of data columns provided. A tic mark is placed at the ends of the error bar (unless **set bars** is used — see **set bars (p. 86)** for details). Either 4 or 6 input columns are required.

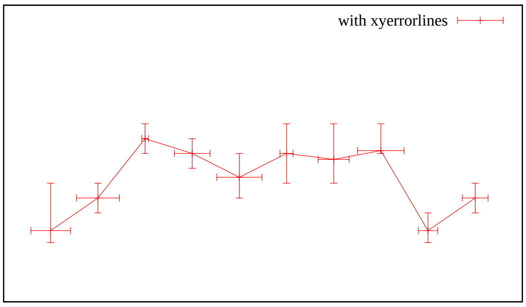


```
4 columns:  x  y  xdelta  ydelta
6 columns:  x  y  xlow  xhigh  ylow  yhigh
```

If data are provided in an unsupported mixed form, the **using** filter on the **plot** command should be used to set up the appropriate form. For example, if the data are of the form (x,y,xdelta,ylow,yhigh), then you can use

```
plot 'data' using 1:2:($1-$3):($1+$3):4:5 with xyerrorlines
```

50 Yerrorlines

The **yerrorlines** (or **errorlines**) style is only relevant to 2D data plots. **yerrorlines** is like **linespoints**, except that a vertical error line is also drawn. At each point (x,y), a line is drawn from (x,y-ydelta) to (x,y+ydelta) or from (x,ylow) to (x,yhigh), depending on how many data columns are provided. A tic mark is placed at the ends of the error bar (see **set bars (p. 86)** for details). Either 3 or 4 input columns are required.

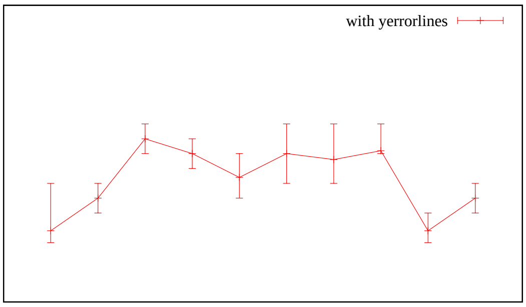


```
3 columns:  x  y  ydelta
4 columns:  x  y  ylow  yhigh
```

See also

errorbar demo.

51 3D (surface) plots

Surface plots are generated using the **splot** command rather than the **plot** command. The style **with lines** draws a surface made from a grid of lines. Solid surfaces can be drawn using the style **with pm3d**. Usually the surface is displayed at some arbitrary viewing angle, such that it clearly represents a 3D surface. In this case the X, Y, and Z axes are all visible in the plot. The illusion of 3D is enhanced by choosing hidden line removal or depth-sorted surface elements. See **hidden3d (p. 101)** and the **depthorder (p. 119)** option of **set pm3d (p. 116)**.

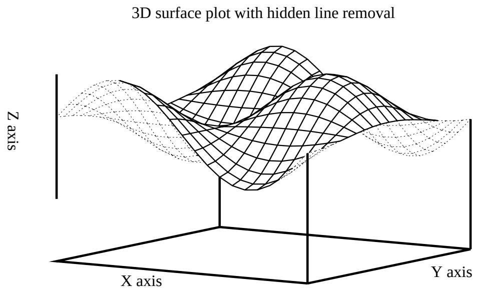

The **splot** command can also calculate and draw contour lines corresponding to constant Z values. These contour lines may be drawn onto the surface itself, or projected onto the XY plane. See **set contour (p. 91)**.

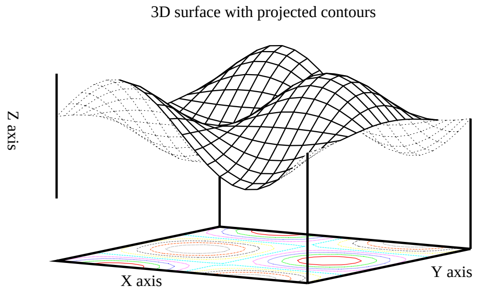

An important special case of the **splot** command is to map the Z coordinate onto a 2D surface by projecting the plot along the Z axis. See **set view map (p. 134)**. This plot mode can be used to generate contour plots and heat maps.

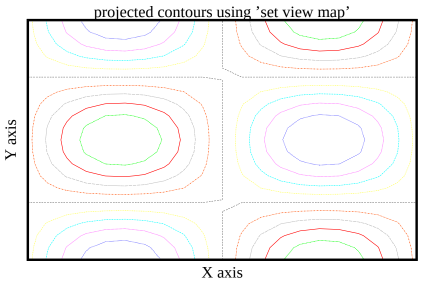

Part III

Commands

This section lists the commands acceptable to **gnuplot** in alphabetical order. Printed versions of this document contain all commands; the text available interactively may not be complete. Indeed, on some systems there may be no commands at all listed under this heading.

Note that in most cases unambiguous abbreviations for command names and their options are permissible, i.e., **"p f(x) w li"** instead of **"plot f(x) with lines"**.

In the syntax descriptions, braces ({}) denote optional arguments and a vertical bar (|) separates mutually exclusive choices.

52 Cd

The **cd** command changes the working directory.

Syntax:

```
cd '<directory-name>'
```

The directory name must be enclosed in quotes.

Examples:

```
cd 'subdir'
cd ".."
```

It is recommended that Windows users use single-quotes, because backslash [\] has special significance inside double-quotes and has to be escaped. For example,

```
cd "c:\newdata"
```

fails, but

```
cd 'c:\newdata'
cd "c:\\newdata"
```

work as expected.

53 Call

The **call** command is identical to the load command with one exception: you can have up to ten additional parameters to the command (delimited according to the standard parser rules) which can be substituted into the lines read from the file. As each line is read from the **call**ed input file, it is scanned for the sequence **$** (dollar-sign) followed by a digit (0–9). If found, the sequence is replaced by the corresponding parameter from the **call** command line. If the parameter was specified as a string in the **call** line, it is substituted without its enclosing quotes. Sequence **$#** is replaced by the number of passed parameters. **$** followed by any character will be that character; e.g. use **$$** to get a single **$**. Providing more than ten parameters on the **call** command line will cause an error. A parameter that was not provided substitutes as nothing. Files being **call**ed may themselves contain **call** or **load** commands.

Syntax:

```
call "<input-file>" <parameter-0> <parm-1> ... <parm-9>
```

The name of the input file must be enclosed in quotes, and it is recommended that parameters are similarly enclosed in quotes (future versions of gnuplot may treat quoted and unquoted arguments differently).

Example:

If the file 'calltest.gp' contains the line:

```
print "argc=$# p0=$0 p1=$1 p2=$2 p3=$3 p4=$4 p5=$5 p6=$6 p7=x$7x"
```

entering the command:

```
call 'calltest.gp' "abcd" 1.2 + "'quoted'" -- "$2"
```

will display:

```
argc=7 p0=abcd p1=1.2 p2=+ p3='quoted' p4=- p5=- p6=$2 p7=xx
```

NOTE: there is a clash in syntax with the datafile **using** callback operator. Use **$$n** or **column(n)** to access column n from a datafile inside a **call**ed datafile plot.

54 Clear

The **clear** command erases the current screen or output device as specified by **set output**. This usually generates a formfeed on hardcopy devices. Use **set terminal** to set the device type.

For some terminals **clear** erases only the portion of the plotting surface defined by **set size**, so for these it can be used in conjunction with **set multiplot** to create an inset.

Example:

```
set multiplot
plot sin(x)
set origin 0.5,0.5
set size 0.4,0.4
clear
plot cos(x)
unset multiplot
```

Please see **set multiplot (p. 111)**, **set size (p. 125)**, and **set origin (p. 115)** for details of these commands.

55 Evaluate

The **evaluate** command executes the commands given as an argument string. Newline characters are not allowed within the string.

Syntax:

```
eval <string expression>
```

This is especially useful for a repetition of similar commands.

Example:

```
set_label(x, y, text) \
  = sprintf("set label '%s' at %f, %f point pt 5", text, x, y)
eval set_label(1., 1., 'one/one')
eval set_label(2., 1., 'two/one')
eval set_label(1., 2., 'one/two')
```

Please see **substitution macros (p. 34)** for another way to execute commands from a string.

56 Exit

The commands **exit** and **quit**, as well as the END-OF-FILE character (usually Ctrl-D) terminate input from the current input stream: terminal session, pipe, and file input (pipe).

If input streams are nested (inherited **load** scripts), then reading will continue in the parent stream. When the top level stream is closed, the program itself will exit.

The command **exit gnuplot** will immediately and unconditionally cause gnuplot to exit even if the input stream is multiply nested. In this case any open output files may not be completed cleanly. Example of use:

```
bind "ctrl-x" "unset output; exit gnuplot"
```

See help for **batch/interactive (p. 18)** for more details.

57 Fit

The **fit** command can fit a user-supplied expression to a set of data points (x,z) or (x,y,z), using an implementation of the nonlinear least-squares (NLLS) Marquardt-Levenberg algorithm. Any user-defined variable occurring in the expression may serve as a fit parameter, but the return type of the expression must be real.

Syntax:

```
fit {<ranges>} <expression>
    '<datafile>' {datafile-modifiers}
    via '<parameter file>' | <var1>{,<var2>,...}
```

Ranges may be specified to temporarily limit the data which is to be fitted; any out-of-range data points are ignored. The syntax is

```
[{dummy_variable=}{<min>}{:<max>}],
```

analogous to **plot**; see **plot ranges (p. 75)**.

<expression> is any valid **gnuplot** expression, although it is usual to use a previously user-defined function of the form f(x) or f(x,y).

<datafile> is treated as in the **plot** command. All the **plot datafile** modifiers (**using**, **every**,...) except **smooth** and the deprecated **thru** are applicable to **fit**. See **plot datafile (p. 62)**.

The default data formats for fitting functions with a single independent variable, z=f(x), are z or x:z. That is, if there is only a single column then it is the dependent variable and the line numbers is the independent variable. If there are two columns, the first is the independent variable and the second is the dependent variable.

Those formats can be changed with the datafile **using** qualifier, for example to take the z value from a different column or to calculate it from several columns. A third **using** qualifier (a column number or an expression), if present, is interpreted as the standard deviation of the corresponding z value and is used to compute a weight for the datum, 1/s**2. Otherwise, all data points are weighted equally, with a weight of one. Note that if you don't specify a **using** option at all, no z standard deviations are read from the datafile even if it does have a third column, so you'll always get unit weights.

To fit a function with two independent variables, z=f(x,y), the required format is **using** with four items, x:y:z:s. The complete format must be given — no default columns are assumed for a missing token. Weights for each data point are evaluated from 's' as above. If error estimates are not available, a constant value can be specified as a constant expression (see **plot datafile using (p. 71)**), e.g., **using 1:2:3:(1)**.

The fit function may have up to five independent variables. There must be two more **using** qualifiers than there are independent variables, unless there is only one variable. The allowed formats, and the default dummy variable names, are as follows:

```
z
x:z
x:z:s
x:y:z:s
x:y:t:z:s
x:y:t:u:z:s
x:y:t:u:v:z:s
```

The dummy variable names may be changed with ranges as noted above. The first range corresponds to the first **using** spec, etc. A range may also be given for z (the dependent variable), but that name cannot be changed.

Multiple datasets may be simultaneously fit with functions of one independent variable by making y a 'pseudo-variable', e.g., the dataline number, and fitting as two independent variables. See **fit multi-branch (p. 57)**.

The **via** qualifier specifies which parameters are to be adjusted, either directly, or by referencing a parameter file.

Examples:

```
f(x) = a*x**2 + b*x + c
g(x,y) = a*x**2 + b*y**2 + c*x*y
FIT_LIMIT = 1e-6
fit f(x) 'measured.dat' via 'start.par'
fit f(x) 'measured.dat' using 3:($7-5) via 'start.par'
fit f(x) './data/trash.dat' using 1:2:3 via a, b, c
fit g(x,y) 'surface.dat' using 1:2:3:(1) via a, b, c
fit a0 + a1*x/(1 + a2*x/(1 + a3*x)) 'measured.dat' via a0,a1,a2,a3
fit a*x + b*y 'surface.dat' using 1:2:3:(1) via a,b
fit [*:*][yaks=*:*] a*x+b*yaks 'surface.dat' u 1:2:3:(1) via a,b
fit a*x + b*y + c*t 'foo.dat' using 1:2:3:4:(1) via a,b,c
h(x,y,t,u,v) = a*x + b*y + c*t + d*u + e*v
fit h(x,y,t,u,v) 'foo.dat' using 1:2:3:4:5:6:(1) via a,b,c,d,e
```

After each iteration step, detailed information about the current state of the fit is written to the display. The same information about the initial and final states is written to a log file, "fit.log". This file is always appended to, so as to not lose any previous fit history; it should be deleted or renamed as desired. By using the command **set fit logfile**, the name of the log file can be changed.

If gnuplot was built with this option, and you activated it using **set fit errorvariables**, the error for each fitted parameter will be stored in a variable named like the parameter, but with "_err" appended. Thus the errors can be used as input for further computations.

The fit may be interrupted by pressing Ctrl-C. After the current iteration completes, you have the option to (1) stop the fit and accept the current parameter values, (2) continue the fit, (3) execute a **gnuplot** command

as specified by the environment variable FIT_SCRIPT. The default for FIT_SCRIPT is **replot**, so if you had previously plotted both the data and the fitting function in one graph, you can display the current state of the fit.

Once **fit** has finished, the **update** command may be used to store final values in a file for subsequent use as a parameter file. See **update (p. 150)** for details.

57.1 Adjustable parameters

There are two ways that **via** can specify the parameters to be adjusted, either directly on the command line or indirectly, by referencing a parameter file. The two use different means to set initial values.

Adjustable parameters can be specified by a comma-separated list of variable names after the **via** keyword. Any variable that is not already defined is created with an initial value of 1.0. However, the fit is more likely to converge rapidly if the variables have been previously declared with more appropriate starting values.

In a parameter file, each parameter to be varied and a corresponding initial value are specified, one per line, in the form

```
varname = value
```

Comments, marked by '#', and blank lines are permissible. The special form

```
varname = value       # FIXED
```

means that the variable is treated as a 'fixed parameter', initialized by the parameter file, but not adjusted by **fit**. For clarity, it may be useful to designate variables as fixed parameters so that their values are reported by **fit**. The keyword **# FIXED** has to appear in exactly this form.

57.2 Short introduction

fit is used to find a set of parameters that 'best' fits your data to your user-defined function. The fit is judged on the basis of the sum of the squared differences or 'residuals' (SSR) between the input data points and the function values, evaluated at the same places. This quantity is often called 'chisquare' (i.e., the Greek letter chi, to the power of 2). The algorithm attempts to minimize SSR, or more precisely, WSSR, as the residuals are 'weighted' by the input data errors (or 1.0) before being squared; see **fit error_estimates (p. 55)** for details.

That's why it is called 'least-squares fitting'. Let's look at an example to see what is meant by 'non-linear', but first we had better go over some terms. Here it is convenient to use z as the dependent variable for user-defined functions of either one independent variable, z=f(x), or two independent variables, z=f(x,y). A parameter is a user-defined variable that **fit** will adjust, i.e., an unknown quantity in the function declaration. Linearity/non-linearity refers to the relationship of the dependent variable, z, to the parameters which **fit** is adjusting, not of z to the independent variables, x and/or y. (To be technical, the second {and higher} derivatives of the fitting function with respect to the parameters are zero for a linear least-squares problem).

For linear least-squares (LLS), the user-defined function will be a sum of simple functions, not involving any parameters, each multiplied by one parameter. NLLS handles more complicated functions in which parameters can be used in a large number of ways. An example that illustrates the difference between linear and nonlinear least-squares is the Fourier series. One member may be written as

```
z=a*sin(c*x) + b*cos(c*x).
```

If a and b are the unknown parameters and c is constant, then estimating values of the parameters is a linear least-squares problem. However, if c is an unknown parameter, the problem is nonlinear.

In the linear case, parameter values can be determined by comparatively simple linear algebra, in one direct step. However LLS is a special case which is also solved along with more general NLLS problems by the iterative procedure that **gnuplot** uses. **fit** attempts to find the minimum by doing a search. Each step (iteration) calculates WSSR with a new set of parameter values. The Marquardt-Levenberg algorithm selects the parameter values for the next iteration. The process continues until a preset criterion is met, either (1) the fit has "converged" (the relative change in WSSR is less than FIT_LIMIT), or (2) it reaches a preset iteration count limit, FIT_MAXITER (see **fit control variables (p. 56)**). The fit may also be interrupted and subsequently halted from the keyboard (see **fit (p. 52)**). The user variable FIT_CONVERGED contains 1 if the previous fit command terminated due to convergence; it contains 0 if the previous fit terminated for any other reason.

Often the function to be fitted will be based on a model (or theory) that attempts to describe or predict the behaviour of the data. Then **fit** can be used to find values for the free parameters of the model, to determine

how well the data fits the model, and to estimate an error range for each parameter. See **fit error_estimates (p. 55)**.

Alternatively, in curve-fitting, functions are selected independent of a model (on the basis of experience as to which are likely to describe the trend of the data with the desired resolution and a minimum number of parameters*functions.) The **fit** solution then provides an analytic representation of the curve.

However, if all you really want is a smooth curve through your data points, the **smooth** option to **plot** may be what you've been looking for rather than **fit**.

57.3 Error estimates

In **fit**, the term "error" is used in two different contexts, data error estimates and parameter error estimates.

Data error estimates are used to calculate the relative weight of each data point when determining the weighted sum of squared residuals, WSSR or chisquare. They can affect the parameter estimates, since they determine how much influence the deviation of each data point from the fitted function has on the final values. Some of the **fit** output information, including the parameter error estimates, is more meaningful if accurate data error estimates have been provided.

The 'statistical overview' describes some of the **fit** output and gives some background for the 'practical guidelines'.

57.3.1 Statistical overview

The theory of non-linear least-squares (NLLS) is generally described in terms of a normal distribution of errors, that is, the input data is assumed to be a sample from a population having a given mean and a Gaussian (normal) distribution about the mean with a given standard deviation. For a sample of sufficiently large size, and knowing the population standard deviation, one can use the statistics of the chisquare distribution to describe a "goodness of fit" by looking at the variable often called "chisquare". Here, it is sufficient to say that a reduced chisquare (chisquare/degrees of freedom, where degrees of freedom is the number of datapoints less the number of parameters being fitted) of 1.0 is an indication that the weighted sum of squared deviations between the fitted function and the data points is the same as that expected for a random sample from a population characterized by the function with the current value of the parameters and the given standard deviations.

If the standard deviation for the population is not constant, as in counting statistics where variance = counts, then each point should be individually weighted when comparing the observed sum of deviations and the expected sum of deviations.

At the conclusion **fit** reports 'stdfit', the standard deviation of the fit, which is the rms of the residuals, and the variance of the residuals, also called 'reduced chisquare' when the data points are weighted. The number of degrees of freedom (the number of data points minus the number of fitted parameters) is used in these estimates because the parameters used in calculating the residuals of the datapoints were obtained from the same data. These values are exported to the variables

```
FIT_NDF = Number of degrees of freedom
FIT_WSSR = Weighted sum-of-squares residual
FIT_STDFIT = sqrt(WSSR/NDF)
```

To estimate confidence levels for the parameters, one can use the minimum chisquare obtained from the fit and chisquare statistics to determine the value of chisquare corresponding to the desired confidence level, but considerably more calculation is required to determine the combinations of parameters which produce such values.

Rather than determine confidence intervals, **fit** reports parameter error estimates which are readily obtained from the variance-covariance matrix after the final iteration. By convention, these estimates are called "standard errors" or "asymptotic standard errors", since they are calculated in the same way as the standard errors (standard deviation of each parameter) of a linear least-squares problem, even though the statistical conditions for designating the quantity calculated to be a standard deviation are not generally valid for the NLLS problem. The asymptotic standard errors are generally over-optimistic and should not be used for determining confidence levels, but are useful for qualitative purposes.

The final solution also produces a correlation matrix, which gives an indication of the correlation of parameters in the region of the solution; if one parameter is changed, increasing chisquare, does changing another compensate? The main diagonal elements, autocorrelation, are all 1; if all parameters were independent, all other elements would be nearly 0. Two variables which completely compensate each other would have an off-diagonal element

of unit magnitude, with a sign depending on whether the relation is proportional or inversely proportional. The smaller the magnitudes of the off-diagonal elements, the closer the estimates of the standard deviation of each parameter would be to the asymptotic standard error.

57.3.2 Practical guidelines

If you have a basis for assigning weights to each data point, doing so lets you make use of additional knowledge about your measurements, e.g., take into account that some points may be more reliable than others. That may affect the final values of the parameters.

Weighting the data provides a basis for interpreting the additional **fit** output after the last iteration. Even if you weight each point equally, estimating an average standard deviation rather than using a weight of 1 makes WSSR a dimensionless variable, as chisquare is by definition.

Each fit iteration will display information which can be used to evaluate the progress of the fit. (An '*' indicates that it did not find a smaller WSSR and is trying again.) The 'sum of squares of residuals', also called 'chisquare', is the WSSR between the data and your fitted function; **fit** has minimized that. At this stage, with weighted data, chisquare is expected to approach the number of degrees of freedom (data points minus parameters). The WSSR can be used to calculate the reduced chisquare (WSSR/ndf) or stdfit, the standard deviation of the fit, sqrt(WSSR/ndf). Both of these are reported for the final WSSR.

If the data are unweighted, stdfit is the rms value of the deviation of the data from the fitted function, in user units.

If you supplied valid data errors, the number of data points is large enough, and the model is correct, the reduced chisquare should be about unity. (For details, look up the 'chi-squared distribution' in your favourite statistics reference.) If so, there are additional tests, beyond the scope of this overview, for determining how well the model fits the data.

A reduced chisquare much larger than 1.0 may be due to incorrect data error estimates, data errors not normally distributed, systematic measurement errors, 'outliers', or an incorrect model function. A plot of the residuals, e.g., **plot 'datafile' using 1:($2-f($1))**, may help to show any systematic trends. Plotting both the data points and the function may help to suggest another model.

Similarly, a reduced chisquare less than 1.0 indicates WSSR is less than that expected for a random sample from the function with normally distributed errors. The data error estimates may be too large, the statistical assumptions may not be justified, or the model function may be too general, fitting fluctuations in a particular sample in addition to the underlying trends. In the latter case, a simpler function may be more appropriate.

You'll have to get used to both **fit** and the kind of problems you apply it to before you can relate the standard errors to some more practical estimates of parameter uncertainties or evaluate the significance of the correlation matrix.

Note that **fit**, in common with most NLLS implementations, minimizes the weighted sum of squared distances (y-f(x))**2. It does not provide any means to account for "errors" in the values of x, only in y. Also, any "outliers" (data points outside the normal distribution of the model) will have an exaggerated effect on the solution.

57.4 Control

There are a number of **gnuplot** variables that can be defined to affect **fit**. Those which can be defined once **gnuplot** is running are listed under 'control_variables' while those defined before starting **gnuplot** are listed under 'environment_variables'.

57.4.1 Control variables

The default epsilon limit (1e-5) may be changed by declaring a value for

```
FIT_LIMIT
```

When the sum of squared residuals changes between two iteration steps by a factor less than this number (epsilon), the fit is considered to have 'converged'.

The maximum number of iterations may be limited by declaring a value for

```
FIT_MAXITER
```

A value of 0 (or not defining it at all) means that there is no limit.

If you need even more control about the algorithm, and know the Marquardt-Levenberg algorithm well, there are some more variables to influence it. The startup value of **lambda** is normally calculated automatically from the ML-matrix, but if you want to, you may provide your own one with

```
FIT_START_LAMBDA
```

Specifying FIT_START_LAMBDA as zero or less will re-enable the automatic selection. The variable

```
FIT_LAMBDA_FACTOR
```

gives the factor by which **lambda** is increased or decreased whenever the chi-squared target function increased or decreased significantly. Setting FIT_LAMBDA_FACTOR to zero re-enables the default factor of 10.0.

Other variables with the FIT_ prefix may be added to **fit**, so it is safer not to use that prefix for user-defined variables.

The variables FIT_SKIP and FIT_INDEX were used by earlier releases of **gnuplot** with a 'fit' patch called **gnufit** and are no longer available. The datafile **every** modifier provides the functionality of FIT_SKIP. FIT_INDEX was used for multi-branch fitting, but multi-branch fitting of one independent variable is now done as a pseudo-3D fit in which the second independent variable and **using** are used to specify the branch. See **fit multi-branch (p. 57)**.

57.4.2 Environment variables

The environment variables must be defined before **gnuplot** is executed; how to do so depends on your operating system.

```
FIT_LOG
```

changes the name (and/or path) of the file to which the fit log will be written from the default of "fit.log" in the working directory. The default value can be overwritten using the command **set fit logfile**.

```
FIT_SCRIPT
```

specifies a command that may be executed after an user interrupt. The default is **replot**, but a **plot** or **load** command may be useful to display a plot customized to highlight the progress of the fit.

57.5 Multi-branch

In multi-branch fitting, multiple data sets can be simultaneously fit with functions of one independent variable having common parameters by minimizing the total WSSR. The function and parameters (branch) for each data set are selected by using a 'pseudo-variable', e.g., either the dataline number (a 'column' index of -1) or the datafile index (-2), as the second independent variable.

Example: Given two exponential decays of the form, z=f(x), each describing a different data set but having a common decay time, estimate the values of the parameters. If the datafile has the format x:z:s, then

```
f(x,y) = (y==0) ? a*exp(-x/tau) : b*exp(-x/tau)
fit f(x,y) 'datafile' using  1:-2:2:3  via a, b, tau
```

For a more complicated example, see the file "hexa.fnc" used by the "fit.dem" demo.

Appropriate weighting may be required since unit weights may cause one branch to predominate if there is a difference in the scale of the dependent variable. Fitting each branch separately, using the multi-branch solution as initial values, may give an indication as to the relative effect of each branch on the joint solution.

57.6 Starting values

Nonlinear fitting is not guaranteed to converge to the global optimum (the solution with the smallest sum of squared residuals, SSR), and can get stuck at a local minimum. The routine has no way to determine that; it is up to you to judge whether this has happened.

fit may, and often will get "lost" if started far from a solution, where SSR is large and changing slowly as the parameters are varied, or it may reach a numerically unstable region (e.g., too large a number causing a floating point overflow) which results in an "undefined value" message or **gnuplot** halting.

To improve the chances of finding the global optimum, you should set the starting values at least roughly in the vicinity of the solution, e.g., within an order of magnitude, if possible. The closer your starting values are to the solution, the less chance of stopping at another minimum. One way to find starting values is to plot data and the fitting function on the same graph and change parameter values and **replot** until reasonable similarity is reached. The same plot is also useful to check whether the fit stopped at a minimum with a poor fit.

Of course, a reasonably good fit is not proof there is not a "better" fit (in either a statistical sense, characterized by an improved goodness-of-fit criterion, or a physical sense, with a solution more consistent with the model.) Depending on the problem, it may be desirable to **fit** with various sets of starting values, covering a reasonable range for each parameter.

57.7 Tips

Here are some tips to keep in mind to get the most out of **fit**. They're not very organized, so you'll have to read them several times until their essence has sunk in.

The two forms of the **via** argument to **fit** serve two largely distinct purposes. The **via "file"** form is best used for (possibly unattended) batch operation, where you just supply the startup values in a file and can later use **update** to copy the results back into another (or the same) parameter file.

The **via var1, var2, ...** form is best used interactively, where the command history mechanism may be used to edit the list of parameters to be fitted or to supply new startup values for the next try. This is particularly useful for hard problems, where a direct fit to all parameters at once won't work without good starting values. To find such, you can iterate several times, fitting only some of the parameters, until the values are close enough to the goal that the final fit to all parameters at once will work.

Make sure that there is no mutual dependency among parameters of the function you are fitting. For example, don't try to fit a*exp(x+b), because a*exp(x+b)=a*exp(b)*exp(x). Instead, fit either a*exp(x) or exp(x+b).

A technical issue: the parameters must not be too different in magnitude. The larger the ratio of the largest and the smallest absolute parameter values, the slower the fit will converge. If the ratio is close to or above the inverse of the machine floating point precision, it may take next to forever to converge, or refuse to converge at all. You will have to adapt your function to avoid this, e.g., replace 'parameter' by '1e9*parameter' in the function definition, and divide the starting value by 1e9.

If you can write your function as a linear combination of simple functions weighted by the parameters to be fitted, by all means do so. That helps a lot, because the problem is no longer nonlinear and should converge with only a small number of iterations, perhaps just one.

Some prescriptions for analysing data, given in practical experimentation courses, may have you first fit some functions to your data, perhaps in a multi-step process of accounting for several aspects of the underlying theory one by one, and then extract the information you really wanted from the fitting parameters of those functions. With **fit**, this may often be done in one step by writing the model function directly in terms of the desired parameters. Transforming data can also quite often be avoided, though sometimes at the cost of a more difficult fit problem. If you think this contradicts the previous paragraph about simplifying the fit function, you are correct.

A "singular matrix" message indicates that this implementation of the Marquardt-Levenberg algorithm can't calculate parameter values for the next iteration. Try different starting values, writing the function in another form, or a simpler function.

Finally, a nice quote from the manual of another fitting package (fudgit), that kind of summarizes all these issues: "Nonlinear fitting is an art!"

58 Help

The **help** command displays built-in help. To specify information on a particular topic use the syntax:

```
help {<topic>}
```

If <topic> is not specified, a short message is printed about **gnuplot**. After help for the requested topic is given, a menu of subtopics is given; help for a subtopic may be requested by typing its name, extending the help request. After that subtopic has been printed, the request may be extended again or you may go back one level to the previous topic. Eventually, the **gnuplot** command line will return.

If a question mark (?) is given as the topic, the list of topics currently available is printed on the screen.

59 History

history command lists or saves previous entries in the history of the command line editing, or executes an entry.

Here you find 'usage by examples':

```
history               # show the complete history
history 5             # show last 5 entries in the history
history quiet 5       # show last 5 entries without entry numbers
history "hist.gp"     # write the complete history to file hist.gp
history "hist.gp" append # append the complete history to file hist.gp
history 10 "hist.gp"  # write last 10 commands to file hist.gp
history 10 "|head -5 >>diary.gp" # write 5 history commands using pipe
history ?load         # show all history entries starting with "load"
history ?"set c"      # like above, several words enclosed in quotes
hi !reread            # execute last entry starting with "reread"
hist !"set xr"        # like above, several words enclosed in quotes
hi !hi                # guess yourself :-))
```

On systems which support a popen function (Unix), the output of history can be piped through an external program by starting the file name with a '|', as one of the above examples demonstrates.

60 If

The **if** command allows commands to be executed conditionally.

Syntax:

```
if (<condition>) <command-line> [; else if (<condition>) ...; else ...]
```

<condition> will be evaluated. If it is true (non-zero), then the command(s) of the <command-line> will be executed. If <condition> is false (zero), then the entire <command-line> is ignored until the next occurrence of **else**. Note that use of **;** to allow multiple commands on the same line will *not* end the conditionalized commands.

Examples:

```
pi=3
if (pi!=acos(-1)) print "?Fixing pi!"; pi=acos(-1); print pi
```

will display:

```
?Fixing pi!
3.14159265358979
```

but

```
if (1==2) print "Never see this"; print "Or this either"
```

will not display anything.

else:

```
v=0
v=v+1; if (v%2) print "2" ; else if (v%3) print "3"; else print "fred"
```

(repeat the last line repeatedly!)

See **reread (p. 81)** for an example of how **if (p. 59)** and **reread (p. 81)** can be used together to perform a loop.

61 Iteration

The **plot**, **splot**, **set** and **unset** commands may optionally contain an iteration clause. This has the effect of executing the basic command multiple times, each time re-evaluating any expressions that make use of the iteration control variable. Two forms of iteration clause are currently supported:

```
for [intvar = start:end{:increment}]
for [stringvar in "A B C D"]
```

Examples:

```
plot for [filename in "A.dat B.dat C.dat"] filename using 1:2 with lines
plot for [basename in "A B C"] basename.".dat" using 1:2 with lines
set for [i = 1:10] style line i lc rgb "blue"
unset for [tag = 100:200] label tag
```

See additional documentation for **plot iteration (p. 76)**.

62 Load

The **load** command executes each line of the specified input file as if it had been typed in interactively. Files created by the **save** command can later be **load**ed. Any text file containing valid commands can be created and then executed by the **load** command. Files being **load**ed may themselves contain **load** or **call** commands. See **comments (p. 19)** for information about comments in commands. To **load** with arguments, see **call (p. 51)**.

Syntax:

```
load "<input-file>"
```

The name of the input file must be enclosed in quotes.

The special filename "-" may be used to **load** commands from standard input. This allows a **gnuplot** command file to accept some commands from standard input. Please see help for **batch/interactive (p. 18)** for more details.

On some systems which support a popen function (Unix), the load file can be read from a pipe by starting the file name with a '<'.

Examples:

```
load 'work.gnu'
load "func.dat"
load "< loadfile_generator.sh"
```

The **load** command is performed implicitly on any file names given as arguments to **gnuplot**. These are loaded in the order specified, and then **gnuplot** exits.

63 Lower

Syntax:

```
lower {plot_window_nb}
```

The **lower** command lowers (opposite to **raise**) plot window(s) associated with the interactive terminal of your gnuplot session, i.e. **pm**, **win**, **wxt** or **x11**. It puts the plot window to bottom in the z-order windows stack of the window manager of your desktop.

As **x11** and **wxt** support multiple plot windows, then by default they lower these windows in descending order of most recently created on top to the least recently created on bottom. If a plot number is supplied as an optional parameter, only the associated plot window will be lowered if it exists.

The optional parameter is ignored for single plot-window terminals, i.e. **pm** and **win**.

64 Pause

The **pause** command displays any text associated with the command and then waits a specified amount of time or until the carriage return is pressed. **pause** is especially useful in conjunction with **load** files.

Syntax:

```
pause <time> {"<string>"}
pause mouse {<endcondition>}{, <endcondition>} {"<string>"}
```

<time> may be any constant or expression. Choosing -1 will wait until a carriage return is hit, zero (0) won't pause at all, and a positive number will wait the specified number of seconds. The time is rounded to an integer number of seconds if subsecond time resolution is not supported by the given platform. **pause 0** is synonymous with **print**.

If the current terminal supports **mousing**, then **pause mouse** will terminate on either a mouse click or on ctrl-C. For all other terminals, or if mousing is not active, **pause mouse** is equivalent to **pause -1**.

If one or more end conditions are given after **pause mouse**, then any one of the conditions will terminate the pause. The possible end conditions are **keypress**, **button1**, **button2**, **button3**, **close**, and **any**. If the pause terminates on a keypress, then the ascii value of the key pressed is returned in MOUSE_KEY. The character itself is returned as a one character string in MOUSE_CHAR. Hotkeys (bind command) are disabled if keypress is one of the end conditions. Zooming is disabled if button3 is one of the end conditions.

In all cases the coordinates of the mouse are returned in variables MOUSE_X, MOUSE_Y, MOUSE_X2, MOUSE_Y2. See **mouse variables (p. 32)**.

Note: Since **pause** communicates with the operating system rather than the graphics, it may behave differently with different device drivers (depending upon how text and graphics are mixed).

Examples:

```
pause -1    # Wait until a carriage return is hit
pause 3     # Wait three seconds
pause -1  "Hit return to continue"
pause 10  "Isn't this pretty?  It's a cubic spline."
pause mouse "Click any mouse button on selected data point"
pause mouse keypress "Type a letter from A-F in the active window"
pause mouse button1,keypress
pause mouse any "Any key or button will terminate"
```

The variant "pause mouse key" will resume after any keypress in the active plot window. If you want to wait for a particular key to be pressed, you can use a reread loop such as:

```
print "I will resume after you hit the Tab key in the plot window"
load "wait_for_tab"
```

File "wait_for_tab" contains the lines

```
pause mouse key
if (MOUSE_KEY != 9) reread
```

65 Plot

plot is the primary command for drawing plots with **gnuplot**. It creates plots of functions and data in many, many ways. **plot** is used to draw 2-d functions and data; **splot** draws 2D projections of 3D surfaces and data. **plot** and **splot** contain many common features; see **splot (p. 145)** for differences. Note specifically that although the **binary <binary list>** variation does work for both **plot** and **splot**, there are small differences between these modes.

Syntax:

```
plot {<ranges>}
     {<iteration>}
     {<function> | {"<datafile>" {datafile-modifiers}}}
     {axes <axes>} {<title-spec>} {with <style>}
     {, {definitions{,}} <function> ...}
```

where either a <function> or the name of a data file enclosed in quotes is supplied. A function is a mathematical expression or a pair of mathematical expressions in parametric mode. The expressions may be defined completely or in part earlier in the stream of **gnuplot** commands (see **user-defined (p. 26)**).

It is also possible to define functions and parameters on the **plot** command itself. This is done merely by isolating them from other items with commas.

Examples:

```
plot sin(x)
```

```
plot sin(x), cos(x)
plot f(x) = sin(x*a), a = .2, f(x), a = .4, f(x)
plot "datafile.1" with lines, "datafile.2" with points
plot [t=1:10] [-pi:pi*2] tan(t), \
     "data.1" using (tan($2)):($3/$4) smooth csplines \
              axes x1y2 notitle with lines 5
plot for [datafile in "spinach.dat broccoli.dat"] datafile
```

See also **show plot (p. 116)**.

65.1 Axes

There are four possible sets of axes available; the keyword <axes> is used to select the axes for which a particular line should be scaled. **x1y1** refers to the axes on the bottom and left; **x2y2** to those on the top and right; **x1y2** to those on the bottom and right; and **x2y1** to those on the top and left. Ranges specified on the **plot** command apply only to the first set of axes (bottom left).

65.2 Data

Discrete data contained in a file can be displayed by specifying the name of the data file (enclosed in single or double quotes) on the **plot** command line.

Syntax:

```
plot '<file_name>' {binary <binary list>}
                   {matrix}
                   {index <index list> | index "<name>"}
                   {every <every list>}
                   {thru <thru expression>}
                   {using <using list>}
                   {smooth <option>}
                   {volatile} {noautoscale}
```

The modifiers **binary**, **index**, **every**, **thru**, **using**, and **smooth** are discussed separately. In brief, **binary** allows data entry from a binary file (default is ASCII), **index** selects which data sets in a multi-data-set file are to be plotted, **every** specifies which points within a single data set are to be plotted, **using** determines how the columns within a single record are to be interpreted (**thru** is a special case of **using**), and **smooth** allows for simple interpolation and approximation. (**splot** has a similar syntax, but does not support the **smooth** and **thru** options.)

The **volatile** keyword indicates that the contents of the data file may be different if the file is re-read. This tells the program to use **refresh** rather than **replot** commands whenever possible. See **refresh (p. 80)**.

The **noautoscale** keyword means that the points making up this plot will be ignored when automatically determining axis range limits.

ASCII DATA FILES:

Data files should contain at least one data point per record (**using** can select one data point from the record). Records beginning with # (and also with **!** on VMS) will be treated as comments and ignored. Each data point represents an (x,y) pair. For **plot**s with error bars or error bars with lines (see **set style errorbars (p. 48)** or **set style errorlines (p. 49)**), each data point is (x,y,ydelta), (x,y,ylow,yhigh), (x,y,xdelta), (x,y,xlow,xhigh), or (x,y,xlow,xhigh,ylow,yhigh).

In all cases, the numbers of each record of a data file must be separated by white space (one or more blanks or tabs) unless a format specifier is provided by the **using** option. This white space divides each record into columns. However, whitespace inside a pair of double quotes is ignored when counting columns, so the following datafile line has three columns:

```
1.0 "second column" 3.0
```

Data may be written in exponential format with the exponent preceded by the letter e or E. The fortran exponential specifiers d, D, q, and Q may also be used if the command **set datafile fortran** is in effect.

Only one column (the y value) need be provided. If x is omitted, **gnuplot** provides integer values starting at 0.

In datafiles, blank records (records with no characters other than blanks and a newline and/or carriage return) are significant.

Single blank records designate discontinuities in a **plot**; no line will join points separated by a blank records (if they are plotted with a line style).

Two blank records in a row indicate a break between separate data sets. See **index (p. 68)**.

If autoscaling has been enabled (**set autoscale**), the axes are automatically extended to include all datapoints, with a whole number of tic marks if tics are being drawn. This has two consequences: i) For **splot**, the corner of the surface may not coincide with the corner of the base. In this case, no vertical line is drawn. ii) When plotting data with the same x range on a dual-axis graph, the x coordinates may not coincide if the x2tics are not being drawn. This is because the x axis has been autoextended to a whole number of tics, but the x2 axis has not. The following example illustrates the problem:

```
reset; plot '-', '-' axes x2y1
1 1
19 19
e
1 1
19 19
e
```

To avoid this, you can use the **fixmin/fixmax** feature of the **set autoscale** command, which turns off the automatic extension of the axis range upto the next tic mark.

Label coordinates and text can also be read from a data file (see **labels (p. 46)**).

BINARY DATA FILES:

Gnuplot can read binary data files. However, adequate information about details of the file format must be given on the command line or extracted from the file itself for a supported binary **filetype**. In particular, there are two structures for binary files, a matrix binary format and a general binary format.

The matrix binary format contains a two dimensional array of 32 bit IEEE float values with an additional column and row of coordinate values. As with ASCII matrix, in the **using** list, repetition of the coordinate row constitutes column 1, repetition of the coordinate column constitutes column 2, and the array of values constitutes column 3.

The general binary format contains an arbitrary number of columns for which information must be specified at the command line. For example, **array**, **record**, **format** and **using** can indicate the size, format and dimension of data. There are a variety of useful commands for skipping file headers and changing endianess. There are a set of commands for positioning and translating data since often coordinates are not part of the file when uniform sampling is inherent in the data. Different from matrix binary or ASCII, general binary does not treat the generated columns as 1, 2 or 3 in the **using** list. Rather, column 1 begins with column 1 of the file, or as specified in the **format** list.

There are global default settings for the various binary options which may be set using the same syntax as the options when used as part of the **(s)plot <filename> binary ...** command. This syntax is **set datafile binary ...**. The general rule is that common command-line specified parameters override file-extracted parameters which override default parameters.

Matrix binary is the default binary format when no keywords specific to general binary are given, i.e., **array**, **record**, **format**, **filetype**.

General binary data can be entered at the command line via the special file name '-'. However, this is intended for use through a pipe where programs can exchange binary data, not for keyboards. There is no "end of record" character for binary data. Gnuplot continues reading from a pipe until it has read the number of points declared in the **array** qualifier.

See **datafile binary (p. 63)** for more details.

65.2.1 Binary

The **binary** keyword allows a data file to be binary as opposed to ASCII. There are two formats for binary– matrix binary and general binary. Matrix binary is a fixed format in which data appears in a 2D array with an extra row and column for coordinate values. General binary is a flexible format for which details about the file must be given at the command line.

See **binary matrix (p. 146)** or **binary general (p. 64)** for more details.

65.2.2 Binary general

General binary data in which format information is not necessarily part of the file can be read by giving further details about the file format at the command line. Although the syntax is slightly arcane to the casual user, general binary is particularly useful for application programs using gnuplot and sending large amounts of data.

Syntax:
```
plot '<file_name>' {binary <binary list>} ...
splot '<file_name>' {binary <binary list>} ...
```

General binary format is activated by keywords in <binary list> pertaining to information about file structure, i.e., **array**, **record**, **format** or **filetype**. Otherwise, matrix binary format is assumed. (See **binary matrix** **(p. 146)** for more details.)

There are some standard file types that may be read for which details about the binary format may be extracted automatically. (Type **show datafile binary** at the command line for a list.) Otherwise, details must be specified at the command line or set in the defaults. Keywords are described below.

The keyword **filetype** in <binary list> controls the routine used to read the file, i.e., the format of the data. For a list of the supported file types, type **show datafile binary filetypes**. If no file type is given, the rule is that traditional gnuplot binary is assumed for **splot** if the **binary** keyword stands alone. In all other circumstances, for **plot** or when one of the <binary list> keywords appears, a raw binary file is assumed whereby the keywords specify the binary format.

General binary data files fall into two basic classes, and some files may be of both classes depending upon how they are treated. There is that class for which uniform sampling is assumed and point coordinates must be generated. This is the class for which full control via the <binary list> keywords applies. For this class, the settings precedence is that command line parameters override in-file parameters, which override default settings. The other class is that set of files for which coordinate information is contained within the file or there is possibly a non-uniform sampling such as gnuplot binary.

Other than for the unique data files such as gnuplot binary, one should think of binary data as conceptually the same as ASCII data. Each point has columns of information which are selected via the <**using list**> associated with **using**. When no **format** string is specified, gnuplot will retrieve a number of binary variables equal to the largest column given in the <**using list**>. For example, **using 1:3** will result in three columns being read, of which the second will be ignored. There are default using lists based upon the typical number of parameters associated with a certain plot type. For example, **with image** has a default of **using 1**, while **with rgbimage** has a default of **using 1:2:3**. Note that the special characters for **using** representing point/line/index generally should not be used for binary data. There are keywords in <binary list> that control this.

65.2.2.1 Array

Describes the sampling array dimensions associated with the binary file. The coordinates will be generated by gnuplot. A number must be specified for each dimension of the array. For example, **array=(10,20)** means the underlying sampling structure is two-dimensional with 10 points along the first (x) dimension and 20 points along the second (y) dimension. A negative number indicates that data should be read until the end of file. If there is only one dimension, the parentheses may be omitted. A colon can be used to separate the dimensions for multiple records. For example, **array=25:35** indicates there are two one-dimensional records in the file.
```
Note:  Gnuplot version 4.2 used the syntax array=128x128 rather than
       array=(128,128). The older syntax is now deprecated, but may
       still work if your copy of gnuplot was built to support
       backwards compatibility.
```

65.2.2.2 Record

This keyword serves the same function as **array**, having the same syntax. However, **record** causes gnuplot to not generate coordinate information. This is for the case where such information may be included in one of the columns of the binary data file.

65.2.2.3 Skip

This keyword allows you to skip sections of a binary file. For instance, if the file contains a 1024 byte header before the start of the data region you would probably want to use
```
plot '<file_name>' binary skip=1024 ...
```

If there are multiple records in the file, you may specify a leading offset for each. For example, to skip 512 bytes before the 1st record and 256 bytes before the second and third records
```
plot '<file_name> binary record=356:356:356 skip=512:256:256 ...
```

65.2.2.4 Format The default binary format is a float. For more flexibility, the format can include details about variable sizes. For example, **format="%uchar%int%float"** associates an unsigned character with the first using column, an int with the second column and a float with the third column. If the number of size specifications is less than the greatest column number, the size is implicitly taken to be similar to the last given variable size.

Furthermore, similar to the **using** specification, the format can include discarded columns via the * character and have implicit repetition via a numerical repeat-field. For example, **format="%*2int%3float"** causes gnuplot to discard two ints before reading three floats. To list variable sizes, type **show datafile binary datasizes**. There are a group of names that are machine dependent along with their sizes in bytes for the particular compilation. There is also a group of names which attempt to be machine independent.

65.2.2.5 Endian Often the endianess of binary data in the file does not agree with the endianess used by the platform on which gnuplot is running. Several words can direct gnuplot how to arrange bytes. For example **endian=little** means treat the binary file as having byte significance from least to greatest. The options are

```
          little:  least significant to greatest significance
             big:  greatest significance to least significance
         default:  assume file endianess is the same as compiler
     swap (swab):  Interchange the significance.  (If things
                   don't look right, try this.)
```

Gnuplot can support "middle" ("pdp") endian if it is compiled with that option.

65.2.2.6 Filetype For some standard binary file formats gnuplot can extract all the necessary information from the file in question. As an example, "format=edf" will read ESRF Header File format files. For a list of the currently supported file formats, type **show datafile binary filetypes**.

There is a special file type called **auto** for which gnuplot will check if the binary file's extension is a quasi-standard extension for a supported format.

Command line keywords may be used to override settings extracted from the file. The settings from the file override any defaults. (See **set datafile binary (p. 94)** for details.)

65.2.2.6.1 Avs **avs** is one of the automatically recognized binary file types for images. AVS is an extremely simple format, suitable mostly for streaming between applications. It consists of 2 longs (xwidth, ywidth) followed by a stream of pixels, each with four bytes of information alpha/red/green/blue.

65.2.2.6.2 Edf **edf** is one of the automatically recognized binary file types for images. EDF stands for ESRF Data Format, and it supports both edf and ehf formats (the latter means ESRF Header Format). More information on specifications can be found at

```
http://www.edfplus.info/specs
```

See also **binary (p. 63)**.

65.2.2.6.3 Png If gnuplot was configured to use the libgd library for png/gif/jpeg output, then it can also be used to read these same image types as binary files. You can use an explicit command

```
plot 'file.png' binary filetype=png
```

Or the file type will be recognized automatically from the extension if you have previously requested

```
set datafile binary filetype=auto
```

See also **binary (p. 63)**.

65.2.2.7 Keywords The following keywords apply only when generating coordinates. That is, when the keyword **array** is used.

65.2.2.7.1 Scan A great deal of confusion can arise concerning the relationship between how gnuplot scans a binary file and the dimensions seen on the plot. To lessen the confusion, conceptually think of gnuplot *always* scanning the binary file point/line/plane or fast/medium/slow. Then this keyword is used to tell gnuplot how to map this scanning convention to the Cartesian convention shown in plots, i.e., x/y/z. The qualifier for scan is a two or three letter code representing where point is assigned (first letter), line is assigned (second letter), and plane is assigned (third letter). For example, **scan=yx** means the fastest, point-by-point, increment should be mapped along the Cartesian y dimension and the middle, line-by-line, increment should be mapped along the x dimension.

When the plotting mode is **plot**, the qualifier code can include the two letters x and y. For **splot**, it can include the three letters x, y and z.

There is nothing restricting the inherent mapping from point/line/plane to apply only to Cartesian coordinates. For this reason there are cylindrical coordinate synonyms for the qualifier codes where t (theta), r and z are analogous to the x, y and z of Cartesian coordinates.

65.2.2.7.2 Transpose Shorthand notation for **scan=yx** or **scan=yxz**.

65.2.2.7.3 Dx, dy, dz When gnuplot generates coordinates, it uses the spacing described by these keywords. For example **dx=10 dy=20** would mean space samples along the x dimension by 10 and space samples along the y dimension by 20. **dy** cannot appear if **dx** does not appear. Similarly, **dz** cannot appear if **dy** does not appear. If the underlying dimensions are greater than the keywords specified, the spacing of the highest dimension given is extended to the other dimensions. For example, if an image is being read from a file and only **dx=3.5** is given gnuplot uses a delta x and delta y of 3.5.

The following keywords also apply only when generating coordinates. However they may also be used with matrix binary files.

65.2.2.7.4 Flipx, flipy, flipz Sometimes the scanning directions in a binary datafile are not consistent with that assumed by gnuplot. These keywords can flip the scanning direction along dimensions x, y, z.

65.2.2.7.5 Origin When gnuplot generates coordinates based upon transposition and flip, it attempts to always position the lower left point in the array at the origin, i.e., the data lies in the first quadrant of a Cartesian system after transpose and flip.

To position the array somewhere else on the graph, the **origin** keyword directs gnuplot to position the lower left point of the array at a point specified by a tuple. The tuple should be a double for **plot** and a triple for **splot**. For example, **origin=(100,100):(100,200)** is for two records in the file and intended for plotting in two dimensions. A second example, **origin=(0,0,3.5)**, is for plotting in three dimensions.

65.2.2.7.6 Center Similar to **origin**, this keyword will position the array such that its center lies at the point given by the tuple. For example, **center=(0,0)**. Center does not apply when the size of the array is **Inf**.

65.2.2.7.7 Rotate The transpose and flip commands provide some flexibility in generating and orienting coordinates. However, for full degrees of freedom, it is possible to apply a rotational vector described by a rotational angle in two dimensions.

The **rotate** keyword applies to the two-dimensional plane, whether it be **plot** or **splot**. The rotation is done with respect to the positive angle of the Cartesian plane.

The angle can be expressed in radians, radians as a multiple of pi, or degrees. For example, **rotate=1.5708**, **rotate=0.5pi** and **rotate=90deg** are equivalent.

If **origin** is specified, the rotation is done about the lower left sample point before translation. Otherwise, the rotation is done about the array **center**.

65.2.2.7.8 Perpendicular For **splot**, the concept of a rotational vector is implemented by a triple representing the vector to be oriented normal to the two-dimensional x-y plane. Naturally, the default is (0,0,1). Thus specifying both rotate and perpendicular together can orient data myriad ways in three-space.

The two-dimensional rotation is done first, followed by the three-dimensional rotation. That is, if R' is the rotational 2 x 2 matrix described by an angle, and P is the 3 x 3 matrix projecting (0,0,1) to (xp,yp,zp), let R

be constructed from R' at the upper left sub-matrix, 1 at element 3,3 and zeros elsewhere. Then the matrix formula for translating data is v' = P R v, where v is the 3 x 1 vector of data extracted from the data file. In cases where the data of the file is inherently not three-dimensional, logical rules are used to place the data in three-space. (E.g., usually setting the z-dimension value to zero and placing 2D data in the x-y plane.)

65.2.2.8 Binary examples

Examples:

```
# Selects two float values (second one implicit) with a float value
# discarded between them for an indefinite length of 1D data.
plot '<file_name>' binary format="%float%*float" using 1:2 with lines

# The data file header contains all details necessary for creating
# coordinates from an EDF file.
plot '<file_name>' binary filetype=edf with image
plot '<file_name>.edf' binary filetype=auto with image

# Selects three unsigned characters for components of a raw RGB image
# and flips the y-dimension so that typical image orientation (start
# at top left corner) translates to the Cartesian plane.  Pixel
# spacing is given and there are two images in the file.  One of them
# is translated via origin.
plot '<file_name>' binary array=(512,1024):(1024,512) format='%uchar' \
     dx=2:1 dy=1:2 origin=(0,0):(1024,1024) flipy u 1:2:3 w rgbimage

# Four separate records in which the coordinates are part of the
# data file.  The file was created with a endianess different from
# the system on which gnuplot is running.
splot '<file_name>' binary record=30:30:29:26 endian=swap u 1:2:3

# Same input file, but this time we skip the 1st and 3rd records
splot '<file_name>' binary record=30:26 skip=360:348 endian=swap u 1:2:3
```

See also **binary matrix (p. 146)**.

65.2.3 Every

The **every** keyword allows a periodic sampling of a data set to be plotted.

In the discussion a "point" is a datum defined by a single record in the file; "block" here will mean the same thing as "datablock" (see **glossary (p. 28)**).

Syntax:

```
plot 'file' every {<point_incr>}
                    {:{<block_incr>}
                      {:{<start_point>}
                        {:{<start_block>}
                          {:{<end_point>}
                            {:<end_block>}}}}}
```

The data points to be plotted are selected according to a loop from <**start_point**> to <**end_point**> with increment <**point_incr**> and the blocks according to a loop from <**start_block**> to <**end_block**> with increment <**block_incr**>.

The first datum in each block is numbered '0', as is the first block in the file.

Note that records containing unplottable information are counted.

Any of the numbers can be omitted; the increments default to unity, the start values to the first point or block, and the end values to the last point or block. If **every** is not specified, all points in all lines are plotted.

Examples:

```
every :::3::3    # selects just the fourth block ('0' is first)
every :::::9     # selects the first 10 blocks
every 2:2        # selects every other point in every other block
every ::5::15    # selects points 5 through 15 in each block
```

See

simple plot demos (simple.dem)

,

Non-parametric splot demos

, and

Parametric splot demos

.

65.2.4 Example datafile

This example plots the data in the file "population.dat" and a theoretical curve:

```
pop(x) = 103*exp((1965-x)/10)
plot [1960:1990] 'population.dat', pop(x)
```

The file "population.dat" might contain:

```
# Gnu population in Antarctica since 1965
   1965   103
   1970   55
   1975   34
   1980   24
   1985   10
```

65.2.5 Index

The **index** keyword allows you to select specific data sets in a multi-data-set file for plotting.

Syntax:

```
plot 'file' index { <m>{:<n>{:<p>}} | "<name>" }
```

Data sets are separated by pairs of blank records. **index <m>** selects only set <m>; **index <m>:<n>** selects sets in the range <m> to <n>; and **index <m>:<n>:<p>** selects indices <m>, <m>+<p>, <m>+2<p>, etc., but stopping at <n>. Following C indexing, the index 0 is assigned to the first data set in the file. Specifying too large an index results in an error message. If <p> is specified but <n> is left blank then every <p>-th dataset is read until the end of the file. If **index** is not specified, the entire file is plotted as a single data set.

Example:

```
plot 'file' index 4:5
```

For each point in the file, the index value of the data set it appears in is available via the pseudo-column **column(-2)**. This leads to an alternative way of distinguishing individual data sets within a file as shown below. This is more awkward than the **index** command if all you are doing is selecting one data set for plotting, but is very useful if you want to assign different properties to each data set. See **pseudocolumns (p. 73)**, **lc variable (p. 30)**.

Example:

```
plot 'file' using 1:(column(-2)==4 ? $2 : NaN)        # very awkward
plot 'file' using 1:2:(column(-2)) linecolor variable # very useful!
```

index '<name>' selects the data set with name '<name>'. Names are assigned to data sets in comment lines. The comment character and leading white space are removed from the comment line. If the resulting line starts with <name>, the following data set is now named <name> and can be selected.

Example:

```
plot 'file' index 'Population'
```

Please note that every comment that starts with <name> will name the following data set. To avoid problems it may be useful to choose a naming scheme like '== Population ==' or '[Population]'.

65.2.6 Smooth

gnuplot includes a few general-purpose routines for interpolation and approximation of data; these are grouped under the **smooth** option. More sophisticated data processing may be performed by preprocessing the data externally or by using **fit** with an appropriate model.

Syntax:

```
smooth {unique | frequency | cumulative | kdensity | csplines | acsplines | bezier | sbezier}
```

unique, **frequency**, and **cumulative** plot the data after making them monotonic. Each of the other routines uses the data to determine the coefficients of a continuous curve between the endpoints of the data. This curve is then plotted in the same manner as a function, that is, by finding its value at uniform intervals along the abscissa (see **set samples (p. 125)**) and connecting these points with straight line segments (if a line style is chosen).

If **autoscale** is in effect, the ranges will be computed such that the plotted curve lies within the borders of the graph.

If **autoscale** is not in effect, and the smooth option is either **acspline** or **cspline**, the sampling of the generated curve is done across the intersection of the x range covered by the input data and the fixed abscissa range as defined by **set xrange**.

If too few points are available to allow the selected option to be applied, an error message is produced. The minimum number is one for **unique** and **frequency**, four for **acsplines**, and three for the others.

The **smooth** options have no effect on function plots.

65.2.6.1 Acsplines

The **acsplines** option approximates the data with a "natural smoothing spline". After the data are made monotonic in x (see **smooth unique (p. 69)**), a curve is piecewise constructed from segments of cubic polynomials whose coefficients are found by the weighting the data points; the weights are taken from the third column in the data file. That default can be modified by the third entry in the **using** list, e.g.,

```
plot 'data-file' using 1:2:(1.0) smooth acsplines
```

Qualitatively, the absolute magnitude of the weights determines the number of segments used to construct the curve. If the weights are large, the effect of each datum is large and the curve approaches that produced by connecting consecutive points with natural cubic splines. If the weights are small, the curve is composed of fewer segments and thus is smoother; the limiting case is the single segment produced by a weighted linear least squares fit to all the data. The smoothing weight can be expressed in terms of errors as a statistical weight for a point divided by a "smoothing factor" for the curve so that (standard) errors in the file can be used as smoothing weights.

Example:

```
sw(x,S)=1/(x*x*S)
plot 'data_file' using 1:2:(sw($3,100)) smooth acsplines
```

65.2.6.2 Bezier

The **bezier** option approximates the data with a Bezier curve of degree n (the number of data points) that connects the endpoints.

65.2.6.3 Csplines

The **csplines** option connects consecutive points by natural cubic splines after rendering the data monotonic (see **smooth unique (p. 69)**).

65.2.6.4 Sbezier

The **sbezier** option first renders the data monotonic (**unique**) and then applies the **bezier** algorithm.

65.2.6.5 Unique

The **unique** option makes the data monotonic in x; points with the same x-value are replaced by a single point having the average y-value. The resulting points are then connected by straight line segments.

65.2.6.6 Frequency

The **frequency** option makes the data monotonic in x; points with the same x-value are replaced by a single point having the summed y-values. The resulting points are then connected by straight line segments. See also

smooth.dem

65.2.6.7 Cumulative The **cumulative** option makes the data monotonic in x; points with the same x-value are replaced by a single point containing the cumulative sum of y-values of all data points with lower x-values (i.e. to the left of the current data point). This can be used to obtain a cumulative distribution function from data. See also

smooth.dem

65.2.6.8 Kdensity The **kdensity** option is a way to plot a kernel density estimate (which is a smooth histogram) for a random collection of points, using Gaussian kernels. A Gaussian is placed at the location of each point in the first column and the sum of all these Gaussians is plotted as a function. The value in the second column is taken as weight of the Gaussian. (To obtain a normalized histogram, this should be 1/number-of-points). The value of the third column, if supplied, is taken as the bandwidth for the kernels. If only two columns have been specified, or if the value of the third column is zero or less, gnuplot calculates the bandwidth which would be optimal if the input data was normally distributed. (This will usually be a very conservative, i.e. broad bandwidth.)

65.2.7 Special-filenames

There are a few filenames that have a special meaning: '', '-', '+' and '++'.

The empty filename '' tells gnuplot to re-use the previous input file in the same plot command. So to plot two columns from the same input file:

```
plot 'filename' using 1:2, '' using 1:3
```

The special filenames '+' and '++' are a mechanism to allow the full range of **using** specifiers and plot styles with in-line functions. Normally a function plot can only have a single y (or z) value associated with each sampled point. The pseudo-file '+' treats the sampled points as column 1, and allows additional column values to be specified via a **using** specification, just as for a true input file. The number of samples returned is controlled by **set samples**. Example:

```
plot '+' using ($1):(sin($1)):(sin($1)**2) with filledcurves
```

Similarly the pseudo-file '++' returns 2 columns of data forming a regular grid of [x,y] coordinates with the number of points along x controlled by **set samples** and the number of points along y controlled by **set isosamples**. You must set xrange and yrange before plotting '++'. Examples:

```
splot '++' using 1:2:(sin($1)*sin($2)) with pm3d
plot '++' using 1:2:(sin($1)*sin($2)) with image
```

The special filename **'-'** specifies that the data are inline; i.e., they follow the command. Only the data follow the command; **plot** options like filters, titles, and line styles remain on the **plot** command line. This is similar to << in unix shell script, and $DECK in VMS DCL. The data are entered as though they are being read from a file, one data point per record. The letter "e" at the start of the first column terminates data entry. The **using** option can be applied to these data — using it to filter them through a function might make sense, but selecting columns probably doesn't!

'-' is intended for situations where it is useful to have data and commands together, e.g., when **gnuplot** is run as a sub-process of some front-end application. Some of the demos, for example, might use this feature. While **plot** options such as **index** and **every** are recognized, their use forces you to enter data that won't be used. For example, while

```
plot '-' index 0, '-' index 1
2
4
6


10
12
14
e
2
4
6
```

```
10
12
14
e
```

does indeed work,

```
plot '-', '-'
2
4
6
e
10
12
14
e
```

is a lot easier to type.

If you use **'-'** with **replot**, you may need to enter the data more than once. See **replot (p. 80)**, **refresh (p. 80)**.

A blank filename ('') specifies that the previous filename should be reused. This can be useful with things like

```
plot 'a/very/long/filename' using 1:2, '' using 1:3, '' using 1:4
```

(If you use both **'-'** and **''** on the same **plot** command, you'll need to have two sets of inline data, as in the example above.)

On systems with a popen function, the datafile can be piped through a shell command by starting the file name with a '<'. For example,

```
pop(x) = 103*exp(-x/10)
plot "< awk '{print $1-1965, $2}' population.dat", pop(x)
```

would plot the same information as the first population example but with years since 1965 as the x axis. If you want to execute this example, you have to delete all comments from the data file above or substitute the following command for the first part of the command above (the part up to the comma):

```
plot "< awk '$0 !~ /^#/ {print $1-1965, $2}' population.dat"
```

While this approach is most flexible, it is possible to achieve simple filtering with the **using** or **thru** keywords.

65.2.8 Thru

The **thru** function is provided for backward compatibility.

Syntax:

```
plot 'file' thru f(x)
```

It is equivalent to:

```
plot 'file' using 1:(f($2))
```

While the latter appears more complex, it is much more flexible. The more natural

```
plot 'file' thru f(y)
```

also works (i.e. you can use y as the dummy variable).

thru is parsed for **splot** and **fit** but has no effect.

65.2.9 Using

The most common datafile modifier is **using**.

Syntax:

```
plot 'file' using {<entry> {:<entry> {:<entry> ...}}} {'format'}
```

If a format is specified, each datafile record is read using the C library's 'scanf' function, with the specified format string. Otherwise the record is read and broken into columns. By default the separation between columns is whitespace (spaces and/or tabs), but see **datafile separator (p. 93)**.

Each <entry> may be a simple column number that selects the value from one field of the input fit, an expression enclosed in parentheses, or empty.

If the entry is an expression in parentheses, then the function column(N) may be used to indicate the value in column N. That is, column(1) refers to the first item read, column(2) to the second, and so on. The special symbols $1, $2, ... are shorthand for column(1), column(2) ... The function **valid(N)** tests whether the value in the Nth column is a valid number.

In addition to the actual columns 1...N in the input data file, gnuplot presents data from several "pseudo-columns" that hold bookkeeping information. E.g. $0 or column(0) returns the sequence number of this data record within a dataset. Please see **pseudocolumns (p. 73)**.

An empty <entry> will default to its order in the list of entries. For example, **using ::4** is interpreted as **using 1:2:4**.

If the **using** list has but a single entry, that <entry> will be used for y and the data point number (pseudo-column $0) is used for x; for example, "**plot 'file' using 1**" is identical to "**plot 'file' using 0:1**". If the **using** list has two entries, these will be used for x and y. See **set style (p. 126)** and **fit (p. 52)** for details about plotting styles that make use of data from additional columns of input.

'scanf' accepts several numerical specifications but **gnuplot** requires all inputs to be double-precision floating-point variables, so "%lf" is essentially the only permissible specifier. A format string given by the user must contain at least one such input specifier, and no more than seven of them. 'scanf' expects to see white space — a blank, tab ("\t"), newline ("\n"), or formfeed ("\f") — between numbers; anything else in the input stream must be explicitly skipped.

Note that the use of "\t", "\n", or "\f" requires use of double-quotes rather than single-quotes.

65.2.9.1 Using_examples This creates a plot of the sum of the 2nd and 3rd data against the first: The format string specifies comma- rather than space-separated columns. The same result could be achieved by specifying **set datafile separator ","**.

```
plot 'file' using 1:($2+$3) '%lf,%lf,%lf'
```

In this example the data are read from the file "MyData" using a more complicated format:

```
plot 'MyData' using "%*lf%lf%*20[^\n]%lf"
```

The meaning of this format is:

```
%*lf        ignore a number
%lf         read a double-precision number (x by default)
%*20[^\n]   ignore 20 non-newline characters
%lf         read a double-precision number (y by default)
```

One trick is to use the ternary **?:** operator to filter data:

```
plot 'file' using 1:($3>10 ? $2 : 1/0)
```

which plots the datum in column two against that in column one provided the datum in column three exceeds ten. **1/0** is undefined; **gnuplot** quietly ignores undefined points, so unsuitable points are suppressed. Or you can use the pre-defined variable NaN to achieve the same result.

In fact, you can use a constant expression for the column number, provided it doesn't start with an opening parenthesis; constructs like **using 0+(complicated expression)** can be used. The crucial point is that the expression is evaluated once if it doesn't start with a left parenthesis, or once for each data point read if it does.

If timeseries data are being used, the time can span multiple columns. The starting column should be specified. Note that the spaces within the time must be included when calculating starting columns for other data. E.g., if the first element on a line is a time with an embedded space, the y value should be specified as column three.

It should be noted that **plot 'file'**, **plot 'file' using 1:2**, and **plot 'file' using ($1):($2)** can be subtly different: 1) if **file** has some lines with one column and some with two, the first will invent x values when they are missing, the second will quietly ignore the lines with one column, and the third will store an undefined value for lines with one point (so that in a plot with lines, no line joins points across the bad point); 2) if a line contains text at the first column, the first will abort the plot on an error, but the second and third should quietly skip the garbage.

In fact, it is often possible to plot a file with lots of lines of garbage at the top simply by specifying

```
plot 'file' using 1:2
```

However, if you want to leave text in your data files, it is safer to put the comment character (#) in the first column of the text lines.

Feeble using demos.

65.2.9.2 Pseudocolumns

Expressions in the **using** clause of a plot statement can refer to additional bookkeeping values in addition to the actual data values contained in the input file. These are contained in "pseudocolumns".

```
column(0)   The sequential order of each point within a data set.
            The counter starts at 0 and is reset by two sequential blank
            records.  The shorthand form $0 is available.
column(-1)  This counter starts at 0 and is reset by a single blank line.
            This corresponds to the data line in array or grid data.
column(-2)  The index number of the current data set within a file that
            contains multiple data sets.  See `index`.
```

65.2.9.3 Xticlabels

Axis tick labels can be generated via a string function, usually taking a data column as an argument. The simplest form uses the data column itself as a string. That is, xticlabels(N) is shorthand for xticlabels(stringcolumn(N)). This example uses the contents of column 3 as x-axis tick labels.

```
plot 'datafile' using <xcol>:<ycol>:xticlabels(3) with <plotstyle>
```

Axis tick labels may be generated for any of the plot axes: x x2 y y2 z. The **ticlabels(<labelcol>)** specifiers must come after all of the data coordinate specifiers in the **using** portion of the command. For each data point which has a valid set of X,Y[,Z] coordinates, the string value given to xticlabels() is added to the list of xtic labels at the same X coordinate as the point it belongs to. **xticlabels()** may be shortened to **xtic()** and so on.

Example:

```
splot "data" using 2:4:6:xtic(1):ytic(3):ztic(6)
```

In this example the x and y axis tic labels are taken from different columns than the x and y coordinate values. The z axis tics, however, are generated from the z coordinate of the corresponding point.

Example:

```
plot "data" using 1:2:xtic( $3 > 10. ? "A" : "B" )
```

This example shows the use of a string-valued function to generate x-axis tick labels. Each point in the data file generates a tick mark on x labeled either "A" or "B" depending on the value in column 3.

65.2.9.4 X2ticlabels

See **plot using xticlabels (p. 73)**.

65.2.9.5 Yticlabels

See **plot using xticlabels (p. 73)**.

65.2.9.6 Y2ticlabels

See **plot using xticlabels (p. 73)**.

65.2.9.7 Zticlabels

See **plot using xticlabels (p. 73)**.

65.3 Errorbars

Error bars are supported for 2D data file plots by reading one to four additional columns (or **using** entries); these additional values are used in different ways by the various errorbar styles.

In the default situation, **gnuplot** expects to see three, four, or six numbers on each line of the data file — either

```
(x, y, ydelta),
(x, y, ylow, yhigh),
(x, y, xdelta),
(x, y, xlow, xhigh),
(x, y, xdelta, ydelta), or
(x, y, xlow, xhigh, ylow, yhigh).
```

The x coordinate must be specified. The order of the numbers must be exactly as given above, though the **using** qualifier can manipulate the order and provide values for missing columns. For example,

```
plot 'file' with errorbars
plot 'file' using 1:2:(sqrt($1)) with xerrorbars
plot 'file' using 1:2:($1-$3):($1+$3):4:5 with xyerrorbars
```

The last example is for a file containing an unsupported combination of relative x and absolute y errors. The **using** entry generates absolute x min and max from the relative error.

The y error bar is a vertical line plotted from (x, ylow) to (x, yhigh). If ydelta is specified instead of ylow and yhigh, ylow = y - ydelta and yhigh = y + ydelta are derived. If there are only two numbers on the record, yhigh and ylow are both set to y. The x error bar is a horizontal line computed in the same fashion. To get lines plotted between the data points, **plot** the data file twice, once with errorbars and once with lines (but remember to use the **notitle** option on one to avoid two entries in the key). Alternately, use the errorlines command (see **errorlines (p. 74)**).

The error bars have crossbars at each end unless **set bars** is used (see **set bars (p. 86)** for details).

If autoscaling is on, the ranges will be adjusted to include the error bars.

See also

errorbar demos.

See **plot using (p. 71)**, **plot with (p. 77)**, and **set style (p. 126)** for more information.

65.4 Errorlines

Lines with error bars are supported for 2D data file plots by reading one to four additional columns (or **using** entries); these additional values are used in different ways by the various errorlines styles.

In the default situation, **gnuplot** expects to see three, four, or six numbers on each line of the data file — either

```
(x, y, ydelta),
(x, y, ylow, yhigh),
(x, y, xdelta),
(x, y, xlow, xhigh),
(x, y, xdelta, ydelta), or
(x, y, xlow, xhigh, ylow, yhigh).
```

The x coordinate must be specified. The order of the numbers must be exactly as given above, though the **using** qualifier can manipulate the order and provide values for missing columns. For example,

```
plot 'file' with errorlines
plot 'file' using 1:2:(sqrt($1)) with xerrorlines
plot 'file' using 1:2:($1-$3):($1+$3):4:5 with xyerrorlines
```

The last example is for a file containing an unsupported combination of relative x and absolute y errors. The **using** entry generates absolute x min and max from the relative error.

The y error bar is a vertical line plotted from (x, ylow) to (x, yhigh). If ydelta is specified instead of ylow and yhigh, ylow = y - ydelta and yhigh = y + ydelta are derived. If there are only two numbers on the record, yhigh and ylow are both set to y. The x error bar is a horizontal line computed in the same fashion.

The error bars have crossbars at each end unless **set bars** is used (see **set bars (p. 86)** for details).

If autoscaling is on, the ranges will be adjusted to include the error bars.

See **plot using (p. 71)**, **plot with (p. 77)**, and **set style (p. 126)** for more information.

65.5 Parametric

When in parametric mode (**set parametric**) mathematical expressions must be given in pairs for **plot** and in triplets for **splot**.

Examples:

```
plot sin(t),t**2
splot cos(u)*cos(v),cos(u)*sin(v),sin(u)
```

Data files are plotted as before, except any preceding parametric function must be fully specified before a data file is given as a plot. In other words, the x parametric function (**sin(t)** above) and the y parametric function (**t**2** above) must not be interrupted with any modifiers or data functions; doing so will generate a syntax error stating that the parametric function is not fully specified.

Other modifiers, such as **with** and **title**, may be specified only after the parametric function has been completed:

```
plot sin(t),t**2 title 'Parametric example' with linespoints
```

See also

Parametric Mode Demos.

65.6 Ranges

The optional ranges specify the region of the graph that will be displayed.

Syntax:

```
[{<dummy-var>=}{{<min>}:{<max>}}]
[{{<min>}:{<max>}}]
```

The first form applies to the independent variable (**xrange** or **trange**, if in parametric mode). The second form applies to the dependent variable **yrange** (and **xrange**, too, if in parametric mode). <dummy-var> is a new name for the independent variable. (The defaults may be changed with **set dummy**.) The optional <min> and <max> terms can be constant expressions or *.

In non-parametric mode, the order in which ranges must be given is **xrange** and **yrange**.

In parametric mode, the order for the **plot** command is **trange**, **xrange**, and **yrange**. The following **plot** command shows setting the **trange** to [-pi:pi], the **xrange** to [-1.3:1.3] and the **yrange** to [-1:1] for the duration of the graph:

```
plot [-pi:pi] [-1.3:1.3] [-1:1] sin(t),t**2
```

Note that the x2range and y2range cannot be specified here — **set x2range** and **set y2range** must be used.

Ranges are interpreted in the order listed above for the appropriate mode. Once all those needed are specified, no further ones must be listed, but unneeded ones cannot be skipped — use an empty range [] as a placeholder.

* can be used to allow autoscaling of either of min and max. See also **set autoscale** **(p. 84)**.

Ranges specified on the **plot** or **splot** command line affect only that graph; use the **set xrange**, **set yrange**, etc., commands to change the default ranges for future graphs.

With time data, you must provide the range (in the same manner as the time appears in the datafile) within quotes. **gnuplot** uses the **timefmt** string to read the value — see **set timefmt** **(p. 132)**.

Examples:

This uses the current ranges:

```
plot cos(x)
```

This sets the x range only:

```
plot [-10:30] sin(pi*x)/(pi*x)
```

This is the same, but uses t as the dummy-variable:

```
plot [t = -10 :30]  sin(pi*t)/(pi*t)
```

This sets both the x and y ranges:

```
plot [-pi:pi] [-3:3]  tan(x), 1/x
```

This sets only the y range, and turns off autoscaling on both axes:

```
plot [ ] [-2:sin(5)*-8] sin(x)**besj0(x)
```

This sets xmax and ymin only:

```
plot [:200] [-pi:]  exp(sin(x))
```

This sets the x range for a timeseries:

```
set timefmt "%d/%m/%y %H:%M"
plot ["1/6/93 12:00":"5/6/93 12:00"] 'timedata.dat'
```

65.7 Iteration

If many similar files or functions are to be plotted together, it may be convenient to do so by iterating over a shared plot command.

Syntax:

```
plot for [<variable> = <start> : <end> {:<increment>}]
plot for [<variable> in "string of words"]
```

The scope of an iteration ends at the next comma or the end of the command, whichever comes first. Iteration can not be nested.

This will plot one curve, sin(3x), because iteration ends at the comma

```
plot for [i=1:3] j=i, sin(j*x)
```

This will plot three curves because there is no comma after the definition of j

```
plot for [i=1:3] j=i sin(j*x)
```

Example:

```
plot for [dataset in "apples bananas"] dataset."dat" title dataset
```

In this example iteration is used both to generate a file name and a corresponding title.

Example:

```
file(n) = sprintf("dataset_%d.dat",n)
splot for [i=1:10] file(i) title sprintf("dataset %d",i)
```

This example defines a string-valued function that generates file names, and plots ten such files together. The iteration variable ('i' in this example) is treated as an integer, and may be used more than once.

Example:

```
set key left
plot [0:1] for [n=1:4] x**n sprintf("%d",n)
```

This example plots a family of functions.

Example:

```
list = "apple banana cabbage daikon eggplant"
item(n) = word(list,n)
plot for [i=1:words(list)] item[i].".dat" title item(i)
list = "new stuff"
replot
```

This example steps through a list and plots once per item. Because the items are retrieved dynamically, you can change the list and then replot.

Example:

```
list = "apple banana cabbage daikon eggplant"
plot for [i in list] i.".dat" title i
list = "new stuff"
replot
```

This is example does exactly the same thing as the previous example, but uses the string iterator form of the command rather than an integer iterator.

65.8 Title

A line title for each function and data set appears in the key, accompanied by a sample of the line and/or symbol used to represent it. It can be changed by using the **title** option.

Syntax:

```
title <text> | notitle [<ignored text>]
title columnheader | title columnheader(N)
```

where <text> must either be a quoted string or a string variable. The quotes will not be shown in the key. A special character may be given as a backslash followed by its octal value ("\345"). The tab character "\t" is understood. Note that backslash processing occurs only for strings enclosed in double quotes — use single quotes to prevent such processing. The newline character "\n" is not processed in key entries in either type of string.

There is also an option that will interpret the first entry in a column of input data (i.e. the column header) as a text field, and use it as the key title. See **datastrings (p. 19)**. This can be made the default by speicifying **set key autotitle columnhead**.

The line title and sample can be omitted from the key by using the keyword **notitle**. A null title (**title ''**) is equivalent to **notitle**. If only the sample is wanted, use one or more blanks (**title ' '**). If **notitle** is followed by a string this string is ignored.

If **key autotitles** is set (which is the default) and neither **title** nor **notitle** are specified the line title is the function name or the file name as it appears on the **plot** command. If it is a file name, any datafile modifiers specified will be included in the default title.

The layout of the key itself (position, title justification, etc.) can be controlled by **set key**. Please see **set key (p. 103)** for details.

Examples:

This plots y=x with the title 'x':

```
plot x
```

This plots x squared with title "x^2" and file "data.1" with title "measured data":

```
plot x**2 title "x^2", 'data.1' t "measured data"
```

This puts an untitled circular border around a polar graph:

```
set polar; plot my_function(t), 1 notitle
```

Plot multiple columns of data, each of which contains its own title in the file

```
plot for [i=1:4] 'data' using i title columnhead
```

65.9 With

Functions and data may be displayed in one of a large number of styles. The **with** keyword provides the means of selection.

Syntax:

```
with <style> { {linestyle | ls <line_style>}
               | {{linetype  | lt <line_type>}
                  {linewidth | lw <line_width>}
                  {linecolor | lc <colorspec>}
                  {pointtype | pt <point_type>}
                  {pointsize | ps <point_size>}
                  {fill | fs <fillstyle>}
                  {nohidden3d} {nocontours} {nosurface}
                  {palette}}
             }
```

where <style> is one of

```
lines        dots       steps     errorbars     xerrorbar     xyerrorlines
points       impulses   fsteps    errorlines    xerrorlines   yerrorbars
linespoints  labels     histeps   financebars   xyerrorbars   yerrorlines
                                  vectors
```

or

```
boxes            candlesticks   image      circles
boxerrorbars     filledcurves   rgbimage
boxxyerrorbars   histograms     rgbalpha   pm3d
```

The first group of styles have associated line, point, and text properties. The second group of styles also have fill properties. See **fillstyle (p. 127)**. Some styles have further sub-styles. See **plotting styles (p. 37)** for details of each.

A default style may be chosen by **set style function** and **set style data**.

By default, each function and data file will use a different line type and point type, up to the maximum number of available types. All terminal drivers support at least six different point types, and re-use them, in order, if more are required. To see the complete set of line and point types available for the current terminal, type **test (p. 149)**.

If you wish to choose the line or point type for a single plot, <line_type> and <point_type> may be specified. These are positive integer constants (or expressions) that specify the line type and point type to be used for the plot. Use **test** to display the types available for your terminal.

You may also scale the line width and point size for a plot by using <line_width> and <point_size>, which are specified relative to the default values for each terminal. The pointsize may also be altered globally — see **set pointsize (p. 124)** for details. But note that both <point_size> as set here and as set by **set pointsize** multiply the default point size — their effects are not cumulative. That is, **set pointsize 2; plot x w p ps 3** will use points three times default size, not six.

It is also possible to specify **pointsize variable** either as part of a line style or for an individual plot. In this case one extra column of input is required, i.e. 3 columns for a 2D plot and 4 columns for a 3D splot. The size of each individual point is determined by multiplying the global pointsize by the value read from the data file.

If you have defined specific line type/width and point type/size combinations with **set style line**, one of these may be selected by setting <line_style> to the index of the desired style.

If gnuplot was built with **pm3d** support, the special keyword **palette** is allowed for smooth color change of lines, points and dots in **splots**. The color is chosen from a smooth palette which was set previously with the command **set palette**. The color value corresponds to the z-value of the point coordinates or to the color coordinate if specified by the 4th parameter in **using**. Both 2d and 3d plots (**plot** and **splot** commands) can use palette colors as specified by either their fractional value or the corresponding value mapped to the colorbox range. A palette color value can also be read from an explicitly specified input column in the **using** specifier. See **colors (p. 29)**, **set palette (p. 119)**, **linetype (p. 29)**.

The keyword **nohidden3d** applies only to plots made with the **splot** command. Normally the global option **set hidden3d** applies to all plots in the graph. You can attach the **nohidden3d** option to any individual plots that you want to exclude from the hidden3d processing. The individual elements other than surfaces (i.e. lines, dots, labels, ...) of a plot marked **nohidden3d** will all be drawn, even if they would normally be obscured by other plot elements.

Similarly, the keyword **nocontours** will turn off contouring for an individual plot even if the global property **set contour** is active.

Similarly, the keyword **nosurface** will turn off the 3D surface for an individual plot even if the global property **set surface** is active.

The keywords may be abbreviated as indicated.

Note that the **linewidth**, **pointsize** and **palette** options are not supported by all terminals.

Examples:

This plots sin(x) with impulses:

```
plot sin(x) with impulses
```

This plots x with points, x**2 with the default:

```
plot x w points, x**2
```

This plots tan(x) with the default function style, file "data.1" with lines:

```
plot [ ] [-2:5] tan(x), 'data.1' with l
```

This plots "leastsq.dat" with impulses:

```
plot 'leastsq.dat' w i
```

This plots the data file "population" with boxes:

```
plot 'population' with boxes
```

This plots "exper.dat" with errorbars and lines connecting the points (errorbars require three or four columns):

```
plot 'exper.dat' w lines, 'exper.dat' notitle w errorbars
```

Another way to plot "exper.dat" with errorlines (errorbars require three or four columns):

```
plot 'exper.dat' w errorlines
```

This plots sin(x) and cos(x) with linespoints, using the same line type but different point types:

```
plot sin(x) with linesp lt 1 pt 3, cos(x) with linesp lt 1 pt 4
```

This plots file "data" with points of type 3 and twice usual size:

```
plot 'data' with points pointtype 3 pointsize 2
```

This plots file "data" with variable pointsize read from column 4

```
plot 'data' using 1:2:4 with points pt 5 pointsize variable
```

This plots two data sets with lines differing only by weight:

```
plot 'd1' t "good" w l lt 2 lw 3, 'd2' t "bad" w l lt 2 lw 1
```

This plots filled curve of x*x and a color stripe:

```
plot x*x with filledcurve closed, 40 with filledcurve y1=10
```

This plots x*x and a color box:

```
plot x*x, (x>=-5 && x<=5 ? 40 : 1/0) with filledcurve y1=10 lt 8
```

This plots a surface with color lines:

```
splot x*x-y*y with line palette
```

This plots two color surfaces at different altitudes:

```
splot x*x-y*y with pm3d, x*x+y*y with pm3d at t
```

66 Print

The **print** command prints the value of <expression> to the screen. It is synonymous with **pause 0**. <expression> may be anything that **gnuplot** can evaluate that produces a number, or it can be a string.

Syntax:

```
print <expression> {, <expression>, ...}
```

See **expressions (p. 21)**. The output file can be set with **set print**.

67 Pwd

The **pwd** command prints the name of the working directory to the screen.

Note that if you wish to store the current directory into a string variable or use it in string expressions, then you can use variable GPVAL_PWD, see **show variables all (p. 134)**. This overcomes shell call by means of **"pwd"**.

68 Quit

The **exit** and **quit** commands and END-OF-FILE character will exit **gnuplot**. Each of these commands will clear the output device (as does the **clear** command) before exiting.

69 Raise

Syntax:

```
raise {plot_window_nb}
```

The **raise** command raises (opposite to **lower**) plot window(s) associated with the interactive terminal of your gnuplot session, i.e. **pm**, **win**, **wxt** or **x11**. It puts the plot window to front (top) in the z-order windows stack of the window manager of your desktop.

As **x11** and **wxt** support multiple plot windows, then by default they raise these windows in descending order of most recently created on top to the least recently created on bottom. If a plot number is supplied as an optional parameter, only the associated plot window will be raised if it exists.

The optional parameter is ignored for single plot-windows terminal, i.e. **pm** and **win**.

If the window is not raised under X11, then perhaps the plot window is running in a different X11 session (telnet or ssh session, for example), or perhaps raising is blocked by your window manager policy setting.

70 Refresh

The **refresh** command is similar to **replot**, with two major differences. **refresh** reformats and redraws the current plot using the data already read in. This means that you can use **refresh** for plots with in-line data (pseudo-device '-') and for plots from datafiles whose contents are volatile. You cannot use the **refresh** command to add new data to an existing plot.

Mousing operations, in particular zoom and unzoom, will use **refresh** rather than **replot** if appropriate. Example:

```
plot 'datafile' volatile with lines, '-' with labels
100 200 "Special point"
e
# Various mousing operations go here
set title "Zoomed in view"
set term post
set output 'zoom.ps'
refresh
```

71 Replot

The **replot** command without arguments repeats the last **plot** or **splot** command. This can be useful for viewing a plot with different **set** options, or when generating the same plot for several devices.

Arguments specified after a **replot** command will be added onto the last **plot** or **splot** command (with an implied ',' separator) before it is repeated. **replot** accepts the same arguments as the **plot** and **splot** commands except that ranges cannot be specified. Thus you can use **replot** to plot a function against the second axes if the previous command was **plot** but not if it was **splot**.

N.B. — use of

```
plot '-' ; ... ; replot
```

is not recommended, because it will require that you type in the data all over again. In most cases you can use the **refresh** command instead, which will redraw the plot using the data previously read in.

Note that **replot** does not work in **multiplot** mode, since it reproduces only the last plot rather than the entire screen.

See also **command-line-editing (p. 18)** for ways to edit the last **plot (p. 61)** (**splot (p. 145)**) command.

See also **show plot (p. 116)** to show the whole current plotting command, and the possibility to copy it into the **history (p. 59)**.

72 Reread

The **reread** command causes the current **gnuplot** command file, as specified by a **load** command or on the command line, to be reset to its starting point before further commands are read from it. This essentially implements an endless loop of the commands from the beginning of the command file to the **reread** command. (But this is not necessarily a disaster — **reread** can be very useful when used in conjunction with **if**. See **if** **(p. 59)** for details.) The **reread** command has no effect if input from standard input.

Examples:

Suppose the file "looper" contains the commands

```
a=a+1
plot sin(x*a)
pause -1
if(a<5) reread
```

and from within **gnuplot** you submit the commands

```
a=0
load 'looper'
```

The result will be five plots (separated by the **pause** message).

Suppose the file "data" contains six columns of numbers with a total yrange from 0 to 10; the first is x and the next are five different functions of x. Suppose also that the file "plotter" contains the commands

```
c_p = c_p+1
plot "$0" using 1:c_p with lines linetype c_p
if(c_p <  n_p) reread
```

and from within **gnuplot** you submit the commands

```
n_p=6
c_p=1
unset key
set yrange [0:10]
set multiplot
call 'plotter' 'data'
unset multiplot
```

The result is a single graph consisting of five plots. The yrange must be set explicitly to guarantee that the five separate graphs (drawn on top of each other in multiplot mode) will have exactly the same axes. The linetype must be specified; otherwise all the plots would be drawn with the same type. See animate.dem in demo directory for an animated example.

73 Reset

The **reset** command causes all graph-related options that can be set with the **set** command to take on their default values. This command is useful, e.g., to restore the default graph settings at the end of a command file, or to return to a defined state after lots of settings have been changed within a command file. Please refer to the **set** command to see the default values that the various options take.

The following are *not* affected by **reset**.

```
‘set term‘ ‘set output‘ ‘set loadpath‘ ‘set fontpath‘
‘set encoding‘ ‘set decimalsign‘ ‘set locale‘
```

reset errors clears only the error state variables GPVAL_ERRNO and GPVAL_ERRMSG.

reset bind restores all hotkey bindings to their default state.

74 Save

The **save** command saves user-defined functions, variables, the **set term** status, all **set** options, or all of these, plus the last **plot** (**splot**) command to the specified file.

Syntax:

```
save  {<option>} '<filename>'
```

where <option> is **functions**, **variables**, **terminal** or **set**. If no option is used, **gnuplot** saves functions, variables, **set** options and the last **plot** (**splot**) command.

saved files are written in text format and may be read by the **load** command. For **save** with the **set** option or without any option, the **terminal** choice and the **output** filename are written out as a comment, to get an output file that works in other installations of gnuplot, without changes and without risk of unwillingly overwriting files.

save terminal will write out just the **terminal** status, without the comment marker in front of it. This is mainly useful for switching the **terminal** setting for a short while, and getting back to the previously set terminal, afterwards, by loading the saved **terminal** status. Note that for a single gnuplot session you may rather use the other method of saving and restoring current terminal by the commands **set term push** and **set term pop**, see **set term (p. 131)**.

The filename must be enclosed in quotes.

The special filename "-" may be used to **save** commands to standard output. On systems which support a popen function (Unix), the output of save can be piped through an external program by starting the file name with a '|'. This provides a consistent interface to **gnuplot**'s internal settings to programs which communicate with **gnuplot** through a pipe. Please see help for **batch/interactive (p. 18)** for more details.

Examples:

```
save 'work.gnu'
save functions 'func.dat'
save var 'var.dat'
save set 'options.dat'
save term 'myterm.gnu'
save '-'
save '|grep title >t.gp'
```

75 Set-show

The **set** command can be used to set *lots* of options. No screen is drawn, however, until a **plot**, **splot**, or **replot** command is given.

The **show** command shows their settings; **show all** shows all the settings.

Options changed using **set** can be returned to the default state by giving the corresponding **unset** command. See also the **reset (p. 81)** command, which returns all settable parameters to default values.

If a variable contains time/date data, **show** will display it according to the format currently defined by **set timefmt**, even if that was not in effect when the variable was initially defined. The **set** and **unset** commands may optionally contain an iteration clause. See **iteration (p. 59)**.

75.1 Angles

By default, **gnuplot** assumes the independent variable in polar graphs is in units of radians. If **set angles degrees** is specified before **set polar**, then the default range is [0:360] and the independent variable has units of degrees. This is particularly useful for plots of data files. The angle setting also applies to 3D mapping as set via the **set mapping** command.

Syntax:

```
set angles {degrees | radians}
show angles
```

The angle specified in **set grid polar** is also read and displayed in the units specified by **set angles**.

set angles also affects the arguments of the machine-defined functions sin(x), cos(x) and tan(x), and the outputs of asin(x), acos(x), atan(x), atan2(x), and arg(x). It has no effect on the arguments of hyperbolic functions or Bessel functions. However, the output arguments of inverse hyperbolic functions of complex arguments are affected; if these functions are used, **set angles radians** must be in effect to maintain consistency between input and output arguments.

```
x={1.0,0.1}
```

```
set angles radians
y=sinh(x)
print y         #prints {1.16933, 0.154051}
print asinh(y)  #prints {1.0, 0.1}
```

but

```
set angles degrees
y=sinh(x)
print y         #prints {1.16933, 0.154051}
print asinh(y)  #prints {57.29578, 5.729578}
```

See also

poldat.dem: polar plot using **set angles** demo.

75.2 Arrow

Arbitrary arrows can be placed on a plot using the **set arrow** command.

Syntax:

```
set arrow {<tag>} {from <position>} {to|rto <position>}
          { {arrowstyle | as <arrow_style>}
            | { {nohead | head | backhead | heads}
                {size <length>,<angle>{,<backangle>}}
                {filled | empty | nofilled}
                {front | back}
                { {linestyle | ls <line_style>}
                  | {linetype | lt <line_type>}
                    {linewidth | lw <line_width} } } }

unset arrow {<tag>}
show arrow {<tag>}
```

<tag> is an integer that identifies the arrow. If no tag is given, the lowest unused tag value is assigned automatically. The tag can be used to delete or change a specific arrow. To change any attribute of an existing arrow, use the **set arrow** command with the appropriate tag and specify the parts of the arrow to be changed.

The <position>s are specified by either x,y or x,y,z, and may be preceded by **first**, **second**, **graph**, **screen**, or **character** to select the coordinate system. Unspecified coordinates default to 0. The end points can be specified in one of five coordinate systems — **first** or **second** axes, **graph**, **screen**, or **character**. See **coordinates (p. 19)** for details. A coordinate system specifier does not carry over from the "from" position to the "to" position. Arrows outside the screen boundaries are permitted but may cause device errors. If the end point is specified by "rto" instead of "to" it is drawn relatively to the start point. For linear axes, **graph** and **screen** coordinates, the distance between the start and the end point corresponds to the given relative coordinate. For logarithmic axes, the relative given coordinate corresponds to the factor of the coordinate between start and end point. Thus, a negative relative value or zero are not allowed for logarithmic axes.

Specifying **nohead** produces an arrow drawn without a head — a line segment. This gives you yet another way to draw a line segment on the plot. By default, an arrow has a head at its end. Specifying **backhead** draws an arrow head at the start point of the arrow while **heads** draws arrow heads on both ends of the line. Not all terminal types support double-ended arrows.

Head size can be controlled by **size <length>,<angle>** or **size <length>,<angle>,<backangle>**, where **<length>** defines length of each branch of the arrow head and **<angle>** the angle (in degrees) they make with the arrow. **<Length>** is in x-axis units; this can be changed by **first**, **second**, **graph**, **screen**, or **character** before the <length>; see **coordinates (p. 19)** for details. **<Backangle>** only takes effect when **filled** or **empty** is also used. Then, **<backangle>** is the angle (in degrees) the back branches make with the arrow (in the same direction as **<angle>**). The **fig** terminal has a restricted backangle function. It supports three different angles. There are two thresholds: Below 70 degrees, the arrow head gets an indented back angle. Above 110 degrees, the arrow head has an acute back angle. Between these thresholds, the back line is straight.

Specifying **filled** produces filled arrow heads (if heads are used). Filling is supported on filled-polygon capable terminals, see help of **pm3d (p. 116)** for their list, otherwise the arrow heads are closed but not filled. The same result (closed but not filled arrow head) is reached by specifying **empty**. Further, filling and outline is

obviously not supported on terminals drawing arrows by their own specific routines, like **metafont**, **metapost**, **latex** or **tgif**.

The line style may be selected from a user-defined list of line styles (see **set style line (p. 129)**) or may be defined here by providing values for <line_type> (an index from the default list of styles) and/or <line_width> (which is a multiplier for the default width).

Note, however, that if a user-defined line style has been selected, its properties (type and width) cannot be altered merely by issuing another **set arrow** command with the appropriate index and **lt** or **lw**.

If **front** is given, the arrow is written on top of the graphed data. If **back** is given (the default), the arrow is written underneath the graphed data. Using **front** will prevent an arrow from being obscured by dense data.

Examples:

To set an arrow pointing from the origin to (1,2) with user-defined style 5, use:

```
set arrow to 1,2 ls 5
```

To set an arrow from bottom left of plotting area to (-5,5,3), and tag the arrow number 3, use:

```
set arrow 3 from graph 0,0 to -5,5,3
```

To change the preceding arrow to end at 1,1,1, without an arrow head and double its width, use:

```
set arrow 3 to 1,1,1 nohead lw 2
```

To draw a vertical line from the bottom to the top of the graph at x=3, use:

```
set arrow from 3, graph 0 to 3, graph 1 nohead
```

To draw a vertical arrow with T-shape ends, use:

```
set arrow 3 from 0,-5 to 0,5 heads size screen 0.1,90
```

To draw an arrow relatively to the start point, where the relative distances are given in graph coordinates, use:

```
set arrow from 0,-5 rto graph 0.1,0.1
```

To draw an arrow with relative end point in logarithmic x axis, use:

```
set logscale x
set arrow from 100,-5 rto 10,10
```

This draws an arrow from 100,-5 to 1000,5. For the logarithmic x axis, the relative coordinate 10 means "factor 10" while for the linear y axis, the relative coordinate 10 means "difference 10".

To delete arrow number 2, use:

```
unset arrow 2
```

To delete all arrows, use:

```
unset arrow
```

To show all arrows (in tag order), use:

```
show arrow
```

arrows demos.

75.3 Autoscale

Autoscaling may be set individually on the x, y or z axis or globally on all axes. The default is to autoscale all axes. If you want to autoscale based on a subset of the plots in the figure, you can mark the other ones with the flag **noautoscale**. See **datafile (p. 62)**.

Syntax:

```
set autoscale {<axes>{|min|max|fixmin|fixmax|fix} | fix | keepfix}
unset autoscale {<axes>}
show autoscale
```

where <axes> is either **x**, **y**, **z**, **cb**, **x2**, **y2** or **xy**. A keyword with **min** or **max** appended (this cannot be done with **xy**) tells **gnuplot** to autoscale just the minimum or maximum of that axis. If no keyword is given, all axes are autoscaled.

A keyword with **fixmin**, **fixmax** or **fix** appended tells gnuplot to disable extension of the axis range to the next tic mark position, for autoscaled axes using equidistant tics; **set autoscale fix** sets this for all axes. Command **set autoscale keepfix** autoscales all axes while keeping the fix settings.

When autoscaling, the axis range is automatically computed and the dependent axis (y for a **plot** and z for **splot**) is scaled to include the range of the function or data being plotted.

If autoscaling of the dependent axis (y or z) is not set, the current y or z range is used.

Autoscaling the independent variables (x for **plot** and x,y for **splot**) is a request to set the domain to match any data file being plotted. If there are no data files, autoscaling an independent variable has no effect. In other words, in the absence of a data file, functions alone do not affect the x range (or the y range if plotting z = f(x,y)).

Please see **set xrange (p. 138)** for additional information about ranges.

The behavior of autoscaling remains consistent in parametric mode, (see **set parametric (p. 116)**). However, there are more dependent variables and hence more control over x, y, and z axis scales. In parametric mode, the independent or dummy variable is t for **plot**s and u,v for **splot**s. **autoscale** in parametric mode, then, controls all ranges (t, u, v, x, y, and z) and allows x, y, and z to be fully autoscaled.

Autoscaling works the same way for polar mode as it does for parametric mode for **plot**, with the extension that in polar mode **set dummy** can be used to change the independent variable from t (see **set dummy (p. 96)**).

When tics are displayed on second axes but no plot has been specified for those axes, x2range and y2range are inherited from xrange and yrange. This is done *before* xrange and yrange are autoextended to a whole number of tics, which can cause unexpected results. You can use the **fixmin** or **fixmax** options to avoid this.

Examples:

This sets autoscaling of the y axis (other axes are not affected):

```
set autoscale y
```

This sets autoscaling only for the minimum of the y axis (the maximum of the y axis and the other axes are not affected):

```
set autoscale ymin
```

This disables extension of the x2 axis tics to the next tic mark, thus keeping the exact range as found in the plotted data and functions:

```
set autoscale x2fixmin
set autoscale x2fixmax
```

This sets autoscaling of the x and y axes:

```
set autoscale xy
```

This sets autoscaling of the x, y, z, x2 and y2 axes:

```
set autoscale
```

This disables autoscaling of the x, y, z, x2 and y2 axes:

```
unset autoscale
```

This disables autoscaling of the z axis only:

```
unset autoscale z
```

75.3.1 Parametric mode

When in parametric mode (**set parametric**), the xrange is as fully scalable as the y range. In other words, in parametric mode the x axis can be automatically scaled to fit the range of the parametric function that is being plotted. Of course, the y axis can also be automatically scaled just as in the non-parametric case. If autoscaling on the x axis is not set, the current x range is used.

Data files are plotted the same in parametric and non-parametric mode. However, there is a difference in mixed function and data plots: in non-parametric mode with autoscaled x, the x range of the datafile controls the x range of the functions; in parametric mode it has no influence.

For completeness a last command **set autoscale t** is accepted. However, the effect of this "scaling" is very minor. When **gnuplot** determines that the t range would be empty, it makes a small adjustment if autoscaling is true. Otherwise, **gnuplot** gives an error. Such behavior may, in fact, not be very useful and the command **set autoscale t** is certainly questionable.

splot extends the above ideas as you would expect. If autoscaling is set, then x, y, and z ranges are computed and each axis scaled to fit the resulting data.

75.3.2 Polar mode

When in polar mode (**set polar**), the xrange and the yrange are both found from the polar coordinates, and thus they can both be automatically scaled. In other words, in polar mode both the x and y axes can be automatically scaled to fit the ranges of the polar function that is being plotted.

When plotting functions in polar mode, the rrange may be autoscaled. When plotting data files in polar mode, the trange may also be autoscaled. Note that if the trange is contained within one quadrant, autoscaling will produce a polar plot of only that single quadrant.

Explicitly setting one or two ranges but not others may lead to unexpected results. See also

    polar demos.

75.4 Bars

The **set bars** command controls the tics at the ends of error bars.

Syntax:

```
set bars {small | large | fullwidth | <size>} {front | back}
unset bars
show bars
```

small is a synonym for 0.0, and **large** for 1.0. The default is 1.0 if no size is given.

The keyword **fullwidth** is relevant only to histograms with errorbars. It sets the width of the errorbar ends to be the same as the width of the associated box in the histogram. It does not change the width of the box itself.

The **front** and **back** keywords are relevant only to errorbars attached to filled rectangles (boxes, candlesticks, histograms).

75.5 Bind

Show the current state of all hotkey bindings. See **bind (p. 31)**.

75.6 Bmargin

The command **set bmargin** sets the size of the bottom margin. Please see **set margin (p. 109)** for details.

75.7 Border

The **set border** and **unset border** commands control the display of the graph borders for the **plot** and **splot** commands. Note that the borders do not necessarily coincide with the axes; with **plot** they often do, but with **splot** they usually do not.

Syntax:

```
set border {<integer>} {front | back} {linewidth | lw <line_width>}
           {{linestyle | ls <line_style>} | {linetype | lt <line_type>}}
unset border
show border
```

With a **splot** displayed in an arbitrary orientation, like **set view 56,103**, the four corners of the x-y plane can be referred to as "front", "back", "left" and "right". A similar set of four corners exist for the top surface, of course. Thus the border connecting, say, the back and right corners of the x-y plane is the "bottom right back" border, and the border connecting the top and bottom front corners is the "front vertical". (This nomenclature is defined solely to allow the reader to figure out the table that follows.)

The borders are encoded in a 12-bit integer: the bottom four bits control the border for **plot** and the sides of the base for **splot**; the next four bits control the verticals in **splot**; the top four bits control the edges on top of the **splot**. In detail, <**integer**> should be the sum of the appropriate entries from the following table:

Graph Border Encoding		
Bit	plot	splot
1	bottom	bottom left front
2	left	bottom left back
4	top	bottom right front
8	right	bottom right back
16	no effect	left vertical
32	no effect	back vertical
64	no effect	right vertical
128	no effect	front vertical
256	no effect	top left back
512	no effect	top right back
1024	no effect	top left front
2048	no effect	top right front

Various bits or combinations of bits may be added together in the command.

The default is 31, which is all four sides for **plot**, and base and z axis for **splot**.

In 2D plots the border is normally drawn on top of all plots elements (**front**). If you want the border to be drawn behind the plot elements, use **set border back**.

Using the optional <line_style>, <line_type> and <line_width> specifiers, the way the border lines are drawn can be influenced (limited by what the current terminal driver supports).

For **plot**, tics may be drawn on edges other than bottom and left by enabling the second axes – see **set xtics (p. 139)** for details.

If a **splot** draws only on the base, as is the case with **"unset surface; set contour base"**, then the verticals and the top are not drawn even if they are specified.

The **set grid** options 'back', 'front' and 'layerdefault' also control the order in which the border lines are drawn with respect to the output of the plotted data.

Examples:

Draw default borders:

```
set border
```

Draw only the left and bottom (**plot**) or both front and back bottom left (**splot**) borders:

```
set border 3
```

Draw a complete box around a **splot**:

```
set border 4095
```

Draw a topless box around a **splot**, omitting the front vertical:

```
set border 127+256+512 # or set border 1023-128
```

Draw only the top and right borders for a **plot** and label them as axes:

```
unset xtics; unset ytics; set x2tics; set y2tics; set border 12
```

75.8 Boxwidth

The **set boxwidth** command is used to set the default width of boxes in the **boxes**, **boxerrorbars**, **candlesticks** and **histograms** styles.

Syntax:

```
set boxwidth {<width>} {absolute|relative}
show boxwidth
```

By default, adjacent boxes are extended in width until they touch each other. A different default width may be specified using the **set boxwidth** command. **Relative** widths are interpreted as being a fraction of this default width.

An explicit value for the boxwidth is interpreted as being a number of units along the current x axis (**absolute**) unless the modifier **relative** is given. If the x axis is a log-scale (see **set log (p. 108)**) then the value of boxwidth is truly "absolute" only at x=1; this physical width is maintained everywhere along the axis (i.e. the boxes do not become narrower the value of x increases). If the range spanned by a log scale x axis is far from x=1, some experimentation may be required to find a useful value of boxwidth.

The default is superseded by explicit width information taken from an extra data column in styles **boxes** or **boxerrorbars**. In a four-column data set, the fourth column will be interpreted as the box width unless the width is set to -2.0, in which case the width will be calculated automatically. See **style boxes (p. 38)** and **style boxerrorbars (p. 37)** for more details.

To set the box width to automatic use the command

```
set boxwidth
```

or, for four-column data,

```
set boxwidth -2
```

The same effect can be achieved with the **using** keyword in **plot**:

```
plot 'file' using 1:2:3:4:(-2)
```

To set the box width to half of the automatic size use

```
set boxwidth 0.5 relative
```

To set the box width to an absolute value of 2 use

```
set boxwidth 2 absolute
```

75.9 Clabel

gnuplot will vary the linetype used for each contour level when clabel is set. When this option on (the default), a legend labels each linestyle with the z level it represents. It is not possible at present to separate the contour labels from the surface key.

Syntax:

```
set clabel {'<format>'}
unset clabel
show clabel
```

The default for the format string is %8.3g, which gives three decimal places. This may produce poor label alignment if the key is altered from its default configuration.

The first contour linetype, or only contour linetype when clabel is off, is the surface linetype +1; contour points are the same style as surface points.

See also **set contour (p. 91)**.

75.10 Clip

gnuplot can clip data points and lines that are near the boundaries of a graph.

Syntax:

```
set clip <clip-type>
unset clip <clip-type>
show clip
```

Three clip types for points and lines are supported by **gnuplot**: **points**, **one**, and **two**. One, two, or all three clip types may be active for a single graph. Note that clipping of color filled quadrangles drawn by **pm3d** maps and surfaces is not controlled by this command, but by **set pm3d clip1in** and **set pm3d clip4in**.

The **points** clip type forces **gnuplot** to clip (actually, not plot at all) data points that fall within but too close to the boundaries. This is done so that large symbols used for points will not extend outside the boundary

lines. Without clipping points near the boundaries, the plot may look bad. Adjusting the x and y ranges may give similar results.

Setting the **one** clip type causes **gnuplot** to draw a line segment which has only one of its two endpoints within the graph. Only the in-range portion of the line is drawn. The alternative is to not draw any portion of the line segment.

Some lines may have both endpoints out of range, but pass through the graph. Setting the **two** clip-type allows the visible portion of these lines to be drawn.

In no case is a line drawn outside the graph.

The defaults are **noclip points**, **clip one**, and **noclip two**.

To check the state of all forms of clipping, use

```
show clip
```

For backward compatibility with older versions, the following forms are also permitted:

```
set clip
unset clip
```

set clip is synonymous with **set clip points**; **unset clip** turns off all three types of clipping.

75.11 Cntrparam

set cntrparam controls the generation of contours and their smoothness for a contour plot. **show contour** displays current settings of **cntrparam** as well as **contour**.

Syntax:

```
set cntrparam { { linear
                | cubicspline
                | bspline
                | points <n>
                | order <n>
                | levels { auto {<n>} | <n>
                         | discrete <z1> {,<z2>{,<z3>...}}
                         | incremental <start>, <incr> {,<end>}
                       }
                }
              }
show contour
```

This command has two functions. First, it sets the values of z for which contour points are to be determined (by linear interpolation between data points or function isosamples.) Second, it controls the way contours are drawn between the points determined to be of equal z. <n> should be an integral constant expression and <z1>, <z2> ... any constant expressions. The parameters are:

linear, **cubicspline**, **bspline** — Controls type of approximation or interpolation. If **linear**, then straight line segments connect points of equal z magnitude. If **cubicspline**, then piecewise-linear contours are interpolated between the same equal z points to form somewhat smoother contours, but which may undulate. If **bspline**, a guaranteed-smoother curve is drawn, which only approximates the position of the points of equal-z.

points — Eventually all drawings are done with piecewise-linear strokes. This number controls the number of line segments used to approximate the **bspline** or **cubicspline** curve. Number of cubicspline or bspline segments (strokes) = **points** * number of linear segments.

order — Order of the bspline approximation to be used. The bigger this order is, the smoother the resulting contour. (Of course, higher order bspline curves will move further away from the original piecewise linear data.) This option is relevant for **bspline** mode only. Allowed values are integers in the range from 2 (linear) to 10.

levels — Selection of contour levels, controlled by **auto** (default), **discrete**, **incremental**, and <n>, number of contour levels.

For **auto**, <n> specifies a nominal number of levels; the actual number will be adjusted to give simple labels. If the surface is bounded by zmin and zmax, contours will be generated at integer multiples of dz between zmin and zmax, where dz is 1, 2, or 5 times some power of ten (like the step between two tic marks).

For **levels discrete**, contours will be generated at z = <z1>, <z2> ... as specified; the number of discrete levels sets the number of contour levels. In **discrete** mode, any **set cntrparam levels <n>** are ignored.

For **incremental**, contours are generated at values of z beginning at <start> and increasing by <increment>, until the number of contours is reached. <end> is used to determine the number of contour levels, which will be changed by any subsequent **set cntrparam levels <n>**. If the z axis is logarithmic, <increment> will be interpreted as a factor, just like in **set ztics**.

If the command **set cntrparam** is given without any arguments specified, the defaults are used: linear, 5 points, order 4, 5 auto levels.

Examples:

```
set cntrparam bspline
set cntrparam points 7
set cntrparam order 10
```

To select levels automatically, 5 if the level increment criteria are met:

```
set cntrparam levels auto 5
```

To specify discrete levels at .1, .37, and .9:

```
set cntrparam levels discrete .1,1/exp(1),.9
```

To specify levels from 0 to 4 with increment 1:

```
set cntrparam levels incremental  0,1,4
```

To set the number of levels to 10 (changing an incremental end or possibly the number of auto levels):

```
set cntrparam levels 10
```

To set the start and increment while retaining the number of levels:

```
set cntrparam levels incremental 100,50
```

See also **set contour** **(p. 91)** for control of where the contours are drawn, and **set clabel** **(p. 88)** for control of the format of the contour labels and linetypes.

See also

```
contours demo (contours.dem)
```

and

```
contours with user defined levels demo (discrete.dem).
```

75.12 Color box

The color scheme, i.e. the gradient of the smooth color with min_z and max_z values of **pm3d**'s **palette**, is drawn in a color box unless **unset colorbox**.

```
set colorbox
set colorbox {
           { vertical | horizontal }
           { default | user }
           { origin x, y }
           { size x, y }
           { front | back }
           { noborder | bdefault | border [line style] }
         }
show colorbox
unset colorbox
```

Color box position can be **default** or **user**. If the latter is specified the values as given with the **origin** and **size** subcommands are used. The box can be drawn after (**front**) or before (**back**) the graph or the surface.

The orientation of the color gradient can be switched by options **vertical** and **horizontal**.

origin x, y and **size x, y** are used only in combination with the **user** option. The x and y values are interpreted as screen coordinates by default, and this is the only legal option for 3D plots. 2D plots, including splot with **set view map**, allow any coordinate system to be specified. Try for example:

```
set colorbox horiz user origin .1,.02 size .8,.04
```

which will draw a horizontal gradient somewhere at the bottom of the graph.

border turns the border on (this is the default). **noborder** turns the border off. If an positive integer argument is given after **border**, it is used as a line style tag which is used for drawing the border, e.g.:

```
set style line 2604 linetype -1 linewidth .4
set colorbox border 2604
```

will use line style **2604**, a thin line with the default border color (-1) for drawing the border. **bdefault** (which is the default) will use the default border line style for drawing the border of the color box.

The axis of the color box is called **cb** and it is controlled by means of the usual axes commands, i.e. **set/unset/show** with **cbrange**, **[m]cbtics**, **format cb**, **grid [m]cb**, **cblabel**, and perhaps even **cbdata**, **[no]cbdtics**, **[no]cbmtics**.

set colorbox without any parameter switches the position to default. **unset colorbox** resets the default parameters for the colorbox and switches the colorbox off.

See also help for **set pm3d (p. 116)**, **set palette (p. 119)**, **x11 pm3d (p. 196)**, and **set style line (p. 129)**.

75.13 Colornames

Gnuplot knows a limited number of color names. You can use these to define the color range spanned by a pm3d palette, or to assign a terminal-independent color to a particular linetype or linestyle. To see the list of known color names, use the command **show colornames (p. 91)**. Example:

```
set style line 1 linecolor rgb "sea-green"
```

75.14 Contour

set contour enables contour drawing for surfaces. This option is available for **splot** only. It requires grid data, see **grid_data (p. 148)** for more details. If contours are desired from non-grid data, **set dgrid3d** can be used to create an appropriate grid.

Syntax:

```
set contour {base | surface | both}
unset contour
show contour
```

The three options specify where to draw the contours: **base** draws the contours on the grid base where the x/ytics are placed, **surface** draws the contours on the surfaces themselves, and **both** draws the contours on both the base and the surface. If no option is provided, the default is **base**.

See also **set cntrparam (p. 89)** for the parameters that affect the drawing of contours, and **set clabel (p. 88)** for control of labelling of the contours.

The surface can be switched off (see **set surface (p. 130)**), giving a contour-only graph. Though it is possible to use **set size** to enlarge the plot to fill the screen, more control over the output format can be obtained by writing the contour information to a file, and rereading it as a 2D datafile plot:

```
unset surface
set contour
set cntrparam ...
set table 'filename'
splot ...
unset table
# contour info now in filename
set term <whatever>
plot 'filename'
```

In order to draw contours, the data should be organized as "grid data". In such a file all the points for a single y-isoline are listed, then all the points for the next y-isoline, and so on. A single blank line (a line containing no characters other than blank spaces and a carriage return and/or a line feed) separates one y-isoline from the next. See also **splot datafile (p. 145)**.

See also

```
contours demo (contours.dem)
```

and

```
contours with user defined levels demo (discrete.dem).
```

75.15 Data style

This form of the command is deprecated. Please see **set style data** (p. 127).

75.16 Datafile

The **set datafile** command options control interpretation of fields read from input data files by the **plot**, **splot**, and **fit** commands. Six such options are currently implemented.

75.16.1 Set datafile fortran

The **set datafile fortran** command enables a special check for values in the input file expressed as Fortran D or Q constants. This extra check slows down the input process, and should only be selected if you do in fact have datafiles containing Fortran D or Q constants. The option can be disabled again using **unset datafile fortran**.

75.16.2 Set datafile nofpe_trap

The **set datafile nofpe_trap** command tells gnuplot not to re-initialize a floating point exception handler before every expression evaluation used while reading data from an input file. This can significantly speed data input from large files at the risk of program termination if a floating-point exception is generated.

75.16.3 Set datafile missing

The **set datafile missing** command allows you to tell **gnuplot** what character string is used in a data file to denote missing data. Exactly how this missing value will be treated depends on the **using** specifier of the **plot** or **splot** command.

Syntax:

```
set datafile missing {"<string>"}
show datafile missing
unset datafile
```

Example:

```
# Ignore entries containing IEEE NaN ("Not a Number") code
set datafile missing "NaN"
```

Example:

```
set style data linespoints
plot '-'
   1 10
   2 20
   3 ?
   4 40
   5 50
   e
set datafile missing "?"
plot '-'
   1 10
   2 20
   3 ?
   4 40
   5 50
   e
```

```
plot '-' using 1:2
   1 10
   2 20
   3 ?
   4 40
   5 50
   e
plot '-' using 1:($2)
   1 10
   2 20
   3 ?
   4 40
   5 50
   e
```

The first **plot** will recognize only the first datum in the "3 ?" line. It will use the single-datum-on-a-line convention that the line number is "x" and the datum is "y", so the point will be plotted (in this case erroneously) at (2,3).

The second and third **plot** commands will correctly ignore the middle line. The plotted line will connect the points at (2,20) and (4,40).

The fourth **plot** will also correctly ignore the middle line, but the plotted line will not connect the points at (2,20) and (4,40).

There is no default character for **missing**, but in many cases any non-parsible string of characters found where a numerical value is expected will be treated as missing data.

75.16.4 Set datafile separator

The command **set datafile separator** "**<char>**" tells **gnuplot** that data fields in subsequent input files are separated by <char> rather than by whitespace. The most common use is to read in csv (comma-separated value) files written by spreadsheet or database programs. By default data fields are separated by whitespace.

Syntax:

```
set datafile separator {"<char>" | whitespace}
```

Examples:

```
# Input file contains tab-separated fields
set datafile separator "\t"

# Input file contains comma-separated values fields
set datafile separator ","
```

75.16.5 Set datafile commentschars

The **set datafile commentschars** command allows you to tell **gnuplot** what characters are used in a data file to denote comments. Gnuplot will ignore rest of the line behind the specified characters if either of them is the first non-blank character on the line.

Syntax:

```
set datafile commentschars {"<string>"}
show datafile commentschars
unset commentschars
```

Default value of the string is "#!" on VMS and "#" otherwise.

Then, the following line in a data file is completely ignored

```
# 1 2 3 4
```

but the following

```
1 # 3 4
```

produces rather unexpected plot unless

```
set datafile missing '#'
```

is specified as well.

Example:

```
set datafile commentschars "#!%"
```

75.16.6 Set datafile binary

The **set datafile binary** command is used to set the defaults when reading binary data files. The syntax matches precisely that used for commands **plot** and **splot**. See **binary (p. 63)** for details about <binary list>.

Syntax:

```
set datafile binary <binary list>
show datafile binary
show datafile
unset datafile
```

Examples:

```
set datafile binary filetype=auto
set datafile binary array=(512,512) format="%uchar"

show datafile binary   # list current settings
```

75.17 Decimalsign

The **set decimalsign** command selects a decimal sign for numbers printed into tic labels or **set label** strings.

Syntax:

```
set decimalsign {<value> | locale {"<locale>"}}
unset decimalsign
show decimalsign
```

The argument <value> is a string to be used in place of the usual decimal point. Typical choices include the period, '.', and the comma, ',', but others may be useful, too. If you omit the <value> argument, the decimal separator is not modified from the usual default, which is a period. Unsetting decimalsign has the same effect as omitting <value>.

Example:

Correct typesetting in most European countries requires:

```
set decimalsign ','
```

Please note: If you set an explicit string, this affects only numbers that are printed using gnuplot's gprintf() formatting routine, include axis tics. It does not affect the format expected for input data, and it does not affect numbers printed with the sprintf() formatting routine. To change the behavior of both input and output formatting, instead use the form

```
set decimalsign locale
```

This instructs the program to use both input and output formats in accordance with the current setting of the LC_ALL, LC_NUMERIC, or LANG environmental variables.

```
set decimalsign locale "foo"
```

This instructs the program to format all input and output in accordance with locale "foo", which must be installed. If locale "foo" is not found then an error message is printed and the decimal sign setting is unchanged. On linux systems you can get a list of the locales installed on your machine by typing "locale -a". A typical linux locale string is of the form "sl_SI.UTF-8". A typical Windows locale string is of the form "Slovenian_Slovenia.1250" or "slovenian". Please note that interpretation of the locale settings is done by the C library at runtime. Older C libraries may offer only partial support for locale settings such as the thousands grouping separator character.

```
set decimalsign locale; set decimalsign "."
```

This sets all input and output to use whatever decimal sign is correct for the current locale, but over-rides this with an explicit '.' in numbers formatted using gnuplot's internal gprintf() function.

75.18 Dgrid3d

The **set dgrid3d** command enables, and can set parameters for, non-grid to grid data mapping. See **splot grid_data (p. 148)** for more details about the grid data structure.

Syntax:

```
set dgrid3d {<rows>} {,{<cols>}}
            { splines |
              qnorm {<norm>} |
              (gauss | cauchy | exp | box | hann) {<dx>} {,dy} }
unset dgrid3d
show dgrid3d
```

By default **dgrid3d** is disabled. When enabled, 3D data read from a file are always treated as a scattered data set. A grid with dimensions derived from a bounding box of the scattered data and size as specified by the row/col_size parameters is created for plotting and contouring. The grid is equally spaced in x (rows) and in y (columns); the z values are computed as weighted averages or spline interpolations of the scattered points' z values. In other words, a regularly spaced grid is created and the a smooth approximation to the raw data is evaluated for all grid points. Only this approximation is plotted, but not the raw data.

The number of columns defaults to the number of rows, which defaults to 10.

Several algorithms are available to calculate the approximation from the raw data. Some of these algorithms can take additional parameters. These interpolations are such the closer the data point is to a grid point, the more effect it has on that grid point.

The **splines** algorithm calculates an interpolation based on "thin plate splines". It does not take additional paramaters.

The **qnorm** algorithm calculates a weighted average of the input data at each grid point. Each data point is weighted inversely by its distance from the grid point raised to the norm power. (Actually, the weights are given by the inverse of dx^norm + dy^norm, where dx and dy are the components of the separation of the grid point from each data point. For some norms that are powers of two, specifically 4, 8, and 16, the computation is optimized by using the Euclidean distance in the weight calculation, (dx^2+dy^2)^norm/2. However, any non-negative integer can be used.) The power of the norm can be specified as a single optional parameter. This algorithm is the default.

Finally, several smoothing kernels are available to calculate weighted averages: z = Sum_i w(d_i) * z_i / Sum_i w(d_i), where z_i is the value of the i-th data point and d_i is the distance between the current grid point and the location of the i-th data point. All kernels assign higher weights to data points that are close to the current grid point and lower weights to data points further away.

The following kernels are available:

```
gauss :     w(d) = exp(-d*d)
cauchy :    w(d) = 1/(1 + d*d)
exp :       w(d) = exp(-d)
box :       w(d) = 1                  if d<1
                 = 0                  otherwise
hann :      w(d) = 0.5*(1-cos(2*pi*d))  if d<1
            w(d) = 0                  otherwise
```

When using one of these five smoothing kernels, up to two additional parameter can be specified: dx and dy. These are used to rescale the coordinate differences when calculating the distance: d_i = sqrt(((x-x_i)/dx)**2 + ((y-y_i)/dy)**2), where x,y are the coordinates of the current grid point and x_i,y_i are the coordinates of the i-th data point. The value of dy defaults to the value of dx, which defaults to 1. The parameters dx and dy make it possible to control the radius over which data points contribute to a grid point IN THE UNITS OF THE DATA ITSELF.

A slightly different syntax is also supported for reasons of backwards compatibility. If no interpolation algorithm has been explicitly selected, the **qnorm** algorithm is assumed. Up to three comma-separated, optional paramaters can be specified, which are interpreted as the the number of rows, the number of columns, and the norm value, respectively.

The **dgrid3d** option is a simple scheme which replaces scattered data with weighted averages on a regular grid.More sophisticated approaches to this problem exist and should be used to preprocess the data outside **gnuplot** if this simple solution is found inadequate.

See also

    dgrid3d.dem: dgrid3d demo.

and

    scatter.dem: dgrid3d demo.

75.19 Dummy

The **set dummy** command changes the default dummy variable names.

Syntax:

```
set dummy {<dummy-var>} {,<dummy-var>}
show dummy
```

By default, **gnuplot** assumes that the independent, or "dummy", variable for the **plot** command is "t" if in parametric or polar mode, or "x" otherwise. Similarly the independent variables for the **splot** command are "u" and "v" in parametric mode (**splot** cannot be used in polar mode), or "x" and "y" otherwise.

It may be more convenient to call a dummy variable by a more physically meaningful or conventional name. For example, when plotting time functions:

```
set dummy t
plot sin(t), cos(t)
```

At least one dummy variable must be set on the command; **set dummy** by itself will generate an error message.

Examples:

```
set dummy u,v
set dummy ,s
```

The second example sets the second variable to s.

75.20 Encoding

The **set encoding** command selects a character encoding. Syntax:

```
set encoding {<value>}
set encoding locale
show encoding
```

Valid values are

```
default     - tells a terminal to use its default encoding
iso_8859_1  - the most common Western European encoding used by many
              Unix workstations and by MS-Windows. This encoding is
              known in the PostScript world as 'ISO-Latin1'.
iso_8859_15 - a variant of iso_8859_1 that includes the Euro symbol
iso_8859_2  - used in Central and Eastern Europe
iso_8859_9  - used in Turkey (also known as Latin5)
koi8r       - popular Unix cyrillic encoding
koi8u       - ukrainian Unix cyrillic encoding
cp437       - codepage for MS-DOS
cp850       - codepage for OS/2, Western Europe
cp852       - codepage for OS/2, Central and Eastern Europe
cp1250      - codepage for MS Windows, Central and Eastern Europe
cp1254      - codepage for MS Windows, Turkish (superset of Latin5)
utf8        - variable-length (multibyte) representation of Unicode
              entry point for each character
```

The command **set encoding locale** is different from the other options. It attempts to determine the current locale from the runtime environment. On most systems this is controlled by the environmental variables LC_ALL, LC_CTYPE, or LANG. This mechanism is necessary, for example, to pass multibyte character encodings such as UTF-8 or EUC_JP to the wxt and cairopdf terminals. This command does not affect the locale-specific representation of dates or numbers. See also **set locale (p. 108)** and **set decimalsign (p. 94)**.

Generally you must set the encoding before setting the terminal type. Note that encoding is not supported by all terminal drivers and that the device must be able to produce the desired non-standard characters.

75.21 Fit

The **fit** setting defines where the **fit** command writes its output. If this option was built into your version of gnuplot, it also controls whether parameter errors from the fit will be written into variables.

Syntax:

```
set fit {logfile {"<filename>"}} {{no}errorvariables}
unset fit
show fit
```

The <filename> argument must be enclosed in single or double quotes.

If no filename is given or **unset fit** is used the log file is reset to its default value "fit.log" or the value of the environmental variable **FIT_LOG**.

If the given logfile name ends with a / or \, it is interpreted to be a directory name, and the actual filename will be "fit.log" in that directory.

If the **errorvariables** option is turned on, the error of each fitted parameter computed by **fit** will be copied to a user-defined variable whose name is formed by appending "_err" to the name of the parameter itself. This is useful mainly to put the parameter and its error onto a plot of the data and the fitted function, for reference, as in:

```
set fit errorvariables
fit f(x) 'datafile' using 1:2 via a, b
print "error of a is:", a_err
set label 'a=%6.2f', a, '+/- %6.2f', a_err
plot 'datafile' using 1:2, f(x)
```

75.22 Fontpath

The **fontpath** setting defines additional locations for font files searched when including font files. Currently only the postscript terminal supports **fontpath**. If a file cannot be found in the current directory, the directories in **fontpath** are tried. Further documentation concerning the supported file formats is included in the **terminal postscript** section of the documentation.

Syntax:

```
set fontpath {"pathlist1" {"pathlist2"...}}
show fontpath
```

Path names may be entered as single directory names, or as a list of path names separated by a platform-specific path separator, eg. colon (':') on Unix, semicolon (';') on DOS/Windows/OS/2/Amiga platforms. The **show fontpath**, **save** and **save set** commands replace the platform-specific separator with a space character (' ') for maximum portability. If a directory name ends with an exclamation mark ('!') also the subdirectories of this directory are searched for font files.

If the environmental variable GNUPLOT_FONTPATH is set, its contents are appended to **fontpath**. If it is not set, a system dependent default value is used. It is set by testing several directories for existence when using the fontpath the first time. Thus, the first call of **set fontpath**, **show fontpath**, **save fontpath**, **plot**, or **splot** with embedded font files takes a little more time. If you want to save this time you may set the environmental variable GNUPLOT_FONTPATH since probing is switched off, then. You can find out which is the default fontpath by using **show fontpath**.

However, **show fontpath** prints the contents of user defined fontpath and system fontpath separately. Also, the **save** and **save set** commands save only the user specified parts of **fontpath**, for portability reasons.

Many other terminal drivers access TrueType fonts via the gd library. For these drivers the font search path is controlled by the environmental variable GDFONTPATH.

75.23 Format

The format of the tic-mark labels can be set with the **set format** command or with the **set tics format** or individual **set {axis}tics format** commands.

Syntax:

```
set format {<axes>} {"<format-string>"}
set format {<axes>} {'<format-string>'}
show format
```

where <axes> is either **x**, **y**, **xy**, **x2**, **y2**, **z**, **cb** or nothing (which applies the format to all axes). The following two commands are equivalent:

```
set format y "%.2f"
set ytics format "%.2f"
```

The length of the string is restricted to 100 characters. The default format is "% g", but other formats such as "%.2f" or "%3.0em" are often desirable. The format "$%g$" is often desirable for LaTeX. If no format string is given, the format will be returned to the default. If the empty string "" is given, tics will have no labels, although the tic mark will still be plotted. To eliminate the tic marks, use **unset xtics** or **set tics scale 0**.

Newline (\n) and enhanced text markup is accepted in the format string. Use double-quotes rather than single-quotes in this case. See also **syntax (p. 35)**. Characters not preceded by "%" are printed verbatim. Thus you can include spaces and labels in your format string, such as "%g m", which will put " m" after each number. If you want "%" itself, double it: "%g %%".

See also **set xtics (p. 139)** for more information about tic labels, and **set decimalsign (p. 94)** for how to use non-default decimal separators in numbers printed this way. See also

```
electron demo (electron.dem).
```

75.23.1 Gprintf

The string function gprintf("format",x) uses gnuplot's own format specifiers, as do the gnuplot commands **set format**, **set timestamp**, and others. These format specifiers are not the same as those used by the standard C-language routine sprintf(). gprintf() accepts only a single variable to be formatted. Gnuplot also provides an sprintf("format",x1,x2,...) routine if you prefer. For a list of gnuplot's format options, see **format specifiers (p. 98)**.

75.23.2 Format specifiers

The acceptable formats (if not in time/date mode) are:

Tic-mark label numerical format specifiers	
Format	Explanation
%f	floating point notation
%e or %E	exponential notation; an "e" or "E" before the power
%g or %G	the shorter of %e (or %E) and %f
%x or %X	hex
%o or %O	octal
%t	mantissa to base 10
%l	mantissa to base of current logscale
%s	mantissa to base of current logscale; scientific power
%T	power to base 10
%L	power to base of current logscale
%S	scientific power
%c	character replacement for scientific power
%P	multiple of pi

A 'scientific' power is one such that the exponent is a multiple of three. Character replacement of scientific powers (**"%c"**) has been implemented for powers in the range -18 to +18. For numbers outside of this range the format reverts to exponential.

Other acceptable modifiers (which come after the "%" but before the format specifier) are "-", which left-justifies the number; "+", which forces all numbers to be explicitly signed; " " (a space), which makes positive numbers have a space in front of them where negative numbers have "-"; "#", which places a decimal point after floats that have only zeroes following the decimal point; a positive integer, which defines the field width; "0" (the digit, not the letter) immediately preceding the field width, which indicates that leading zeroes are to be used instead of leading blanks; and a decimal point followed by a non-negative integer, which defines the

precision (the minimum number of digits of an integer, or the number of digits following the decimal point of a float).

Some systems may not support all of these modifiers but may also support others; in case of doubt, check the appropriate documentation and then experiment.

Examples:

```
set format y "%t"; set ytics (5,10)          # "5.0" and "1.0"
set format y "%s"; set ytics (500,1000)      # "500" and "1.0"
set format y "%+-12.3f"; set ytics(12345)    # "+12345.000  "
set format y "%.2t*10^%+03T"; set ytic(12345)# "1.23*10^+04"
set format y "%s*10^{%S}"; set ytic(12345)   # "12.345*10^{3}"
set format y "%s %cg"; set ytic(12345)       # "12.345 kg"
set format y "%.0P pi"; set ytic(6.283185)   # "2 pi"
set format y "%.0f%%"; set ytic(50)          # "50%"

set log y 2; set format y '%l'; set ytics (1,2,3)
#displays "1.0", "1.0" and "1.5" (since 3 is 1.5 * 2^1)
```

There are some problem cases that arise when numbers like 9.999 are printed with a format that requires both rounding and a power.

If the data type for the axis is time/date, the format string must contain valid codes for the 'strftime' function (outside of **gnuplot**, type "man strftime"). See **set timefmt (p. 132)** for a list of the allowed input format codes.

75.23.3 Time/date specifiers

In time/date mode, the acceptable formats are:

Tic-mark label Date/Time Format Specifiers	
Format	Explanation
%a	abbreviated name of day of the week
%A	full name of day of the week
%b or %h	abbreviated name of the month
%B	full name of the month
%d	day of the month, 01–31
%D	shorthand for "%m/%d/%y" (only output)
%F	shorthand for "%Y-%m-%d" (only output)
%k	hour, 0–23 (one or two digits)
%H	hour, 00–23 (always two digits)
%l	hour, 1–12 (one or two digits)
%I	hour, 01–12 (always two digits)
%j	day of the year, 1–366
%m	month, 01–12
%M	minute, 0–60
%p	"am" or "pm"
%r	shorthand for "%I:%M:%S %p" (only output)
%R	shorthand for %H:%M" (only output)
%S	second, 0–60
%T	shorthand for "%H:%M:%S" (only output)
%U	week of the year (week starts on Sunday)
%w	day of the week, 0–6 (Sunday = 0)
%W	week of the year (week starts on Monday)
%y	year, 0-99
%Y	year, 4-digit

Except for the non-numerical formats, these may be preceded by a "0" ("zero", not "oh") to pad the field length with leading zeroes, and a positive digit, to define the minimum field width (which will be overridden if the specified width is not large enough to contain the number). There is a 24-character limit to the length of the printed text; longer strings will be truncated.

Examples:

Suppose the text is "76/12/25 23:11:11". Then

```
set format x                  # defaults to "12/25/76" \n "23:11"
set format x "%A, %d %b %Y"   # "Saturday, 25 Dec 1976"
set format x "%r %D"          # "11:11:11 pm 12/25/76"
```

Suppose the text is "98/07/06 05:04:03". Then

```
set format x "%1y/%2m/%3d %01H:%02M:%03S"  # "98/ 7/  6 5:04:003"
```

75.24 Function style

This form of the command is deprecated. Please see **set style function (p. 128)**.

75.25 Functions

The **show functions** command lists all user-defined functions and their definitions.

Syntax:

```
show functions
```

For information about the definition and usage of functions in **gnuplot**, please see **expressions (p. 21)**. See also

splines as user defined functions (spline.dem)

and

use of functions and complex variables for airfoils (airfoil.dem).

75.26 Grid

The **set grid** command allows grid lines to be drawn on the plot.

Syntax:

```
set grid {{no}{m}xtics} {{no}{m}ytics} {{no}{m}ztics}
         {{no}{m}x2tics} {{no}{m}y2tics}
         {{no}{m}cbtics}
         {polar {<angle>}}
         {layerdefault | front | back}
         { {linestyle <major_linestyle>}
           | {linetype | lt <major_linetype>}
             {linewidth | lw <major_linewidth>}
           { , {linestyle | ls <minor_linestyle>}
               | {linetype | lt <minor_linetype>}
                 {linewidth | lw <minor_linewidth>} } }
unset grid
show grid
```

The grid can be enabled and disabled for the major and/or minor tic marks on any axis, and the linetype and linewidth can be specified for major and minor grid lines, also via a predefined linestyle, as far as the active terminal driver supports this.

Additionally, a polar grid can be selected for 2D plots — circles are drawn to intersect the selected tics, and radial lines are drawn at definable intervals. (The interval is given in degrees or radians, depending on the **set angles** setting.) Note that a polar grid is no longer automatically generated in polar mode.

The pertinent tics must be enabled before **set grid** can draw them; **gnuplot** will quietly ignore instructions to draw grid lines at non-existent tics, but they will appear if the tics are subsequently enabled.

If no linetype is specified for the minor gridlines, the same linetype as the major gridlines is used. The default polar angle is 30 degrees.

If **front** is given, the grid is drawn on top of the graphed data. If **back** is given, the grid is drawn underneath the graphed data. Using **front** will prevent the grid from being obscured by dense data. The default setup, **layerdefault**, is equivalent to **back** for 2d plots. In 3D plots the default is to split up the grid and the graph box into two layers: one behind, the other in front of the plotted data and functions. Since **hidden3d** mode

does its own sorting, it ignores all grid drawing order options and passes the grid lines through the hidden line removal machinery instead. These options actually affect not only the grid, but also the lines output by **set border** and the various ticmarks (see **set xtics (p. 139)**).

Z grid lines are drawn on the bottom of the plot. This looks better if a partial box is drawn around the plot — see **set border (p. 86)**.

75.27 Hidden3d

The **set hidden3d** command enables hidden line removal for surface plotting (see **splot (p. 145)**). Some optional features of the underlying algorithm can also be controlled using this command.

Syntax:

```
set hidden3d {defaults} |
             { {front|back}
               {{offset <offset>} | {nooffset}}
               {trianglepattern <bitpattern>}
               {{undefined <level>} | {noundefined}}
               {{no}altdiagonal}
               {{no}bentover} }
unset hidden3d
show hidden3d
```

In contrast to the usual display in gnuplot, hidden line removal actually treats the given function or data grids as real surfaces that can't be seen through, so plot elements behind the surface will be hidden by it. For this to work, the surface needs to have 'grid structure' (see **splot datafile (p. 145)** about this), and it has to be drawn **with lines** or **with linespoints**.

When **hidden3d** is set, both the hidden portion of the surface and possibly its contours drawn on the base (see **set contour (p. 91)**) as well as the grid will be hidden. Each surface has its hidden parts removed with respect to itself and to other surfaces, if more than one surface is plotted. Contours drawn on the surface (**set contour surface**) don't work.

Labels and arrows are always visible and are unaffected. The key box is never hidden by the surface. As of gnuplot version 4.2, **set hidden3d** also affects 3D plotting styles **with points**, **with labels**, and **with vectors**, even if no surface is present in the graph. Individual plots within the graph may be explicitly excluded from this processing by appending the extra option **nohidden3d** to the **with** specifier.

Hidden3d does not affect solid surfaces drawn using the pm3d mode. To achieve a similar effect purely for pm3d surfaces, use instead **set pm3d depthorder**. To mix pm3d surfaces with normal **hidden3d** processing, use the option **set hidden3d front** to force all elements included in hidden3d processing to be drawn after any remaining plot elements. Then draw the surface twice, once **with lines lt -2** and a second time **with pm3d**. The first instance will include the surface during calculation of occluded elements but will not draw the surface itself.

Functions are evaluated at isoline intersections. The algorithm interpolates linearly between function points or data points when determining the visible line segments. This means that the appearance of a function may be different when plotted with **hidden3d** than when plotted with **nohidden3d** because in the latter case functions are evaluated at each sample. Please see **set samples (p. 125)** and **set isosamples (p. 102)** for discussion of the difference.

The algorithm used to remove the hidden parts of the surfaces has some additional features controllable by this command. Specifying **defaults** will set them all to their default settings, as detailed below. If **defaults** is not given, only explicitly specified options will be influenced: all others will keep their previous values, so you can turn on/off hidden line removal via **set {no}hidden3d**, without modifying the set of options you chose.

The first option, **offset**, influences the linestyle used for lines on the 'back' side. Normally, they are drawn in a linestyle one index number higher than the one used for the front, to make the two sides of the surface distinguishable. You can specify a different line style offset to add instead of the default 1, by **offset <offset>**. Option **nooffset** stands for **offset 0**, making the two sides of the surface use the same linestyle.

Next comes the option **trianglepattern <bitpattern>**. <bitpattern> must be a number between 0 and 7, interpreted as a bit pattern. Each bit determines the visibility of one edge of the triangles each surface is split up into. Bit 0 is for the 'horizontal' edges of the grid, Bit 1 for the 'vertical' ones, and Bit 2 for the diagonals that split each cell of the original grid into two triangles. The default pattern is 3, making all horizontal and vertical lines visible, but not the diagonals. You may want to choose 7 to see those diagonals as well.

The **undefined** <level> option lets you decide what the algorithm is to do with data points that are undefined (missing data, or undefined function values), or exceed the given x-, y- or z-ranges. Such points can either be plotted nevertheless, or taken out of the input data set. All surface elements touching a point that is taken out will be taken out as well, thus creating a hole in the surface. If <level> = 3, equivalent to option **noundefined**, no points will be thrown away at all. This may produce all kinds of problems elsewhere, so you should avoid this. <level> = 2 will throw away undefined points, but keep the out-of-range ones. <level> = 1, the default, will get rid of out-of-range points as well.

By specifying **noaltdiagonal**, you can override the default handling of a special case can occur if **undefined** is active (i.e. <level> is not 3). Each cell of the grid-structured input surface will be divided in two triangles along one of its diagonals. Normally, all these diagonals have the same orientation relative to the grid. If exactly one of the four cell corners is excluded by the **undefined** handler, and this is on the usual diagonal, both triangles will be excluded. However if the default setting of **altdiagonal** is active, the other diagonal will be chosen for this cell instead, minimizing the size of the hole in the surface.

The **bentover** option controls what happens to another special case, this time in conjunction with the **trianglepattern**. For rather crumply surfaces, it can happen that the two triangles a surface cell is divided into are seen from opposite sides (i.e. the original quadrangle is 'bent over'), as illustrated in the following ASCII art:

```
                                                          C----B
  original quadrangle:  A--B      displayed quadrangle:     |\   |
    ("set view 0,0")    | /|    ("set view 75,75" perhaps)  | \  |
                        |/ |                                |  \ |
                        C--D                                |   \|
                                                          A    D
```

If the diagonal edges of the surface cells aren't generally made visible by bit 2 of the <bitpattern> there, the edge CB above wouldn't be drawn at all, normally, making the resulting display hard to understand. Therefore, the default option of **bentover** will turn it visible in this case. If you don't want that, you may choose **nobentover** instead. See also

`hidden line removal demo (hidden.dem)`

and

`complex hidden line demo (singulr.dem).`

75.28 Historysize

Note: the command **set historysize** is only available when gnuplot has been configured with the GNU readline.

Syntax:

```
set historysize <int>
unset historysize
```

When leaving gnuplot, the value of historysize is used for truncating the history to at most that much lines. The default is 500. **unset historysize** will disable history truncation and thus allow an infinite number of lines to be written to the history file.

75.29 Isosamples

The isoline density (grid) for plotting functions as surfaces may be changed by the **set isosamples** command.

Syntax:

```
set isosamples <iso_1> {,<iso_2>}
show isosamples
```

Each function surface plot will have <iso_1> iso-u lines and <iso_2> iso-v lines. If you only specify <iso_1>, <iso_2> will be set to the same value as <iso_1>. By default, sampling is set to 10 isolines per u or v axis. A higher sampling rate will produce more accurate plots, but will take longer. These parameters have no effect on data file plotting.

An isoline is a curve parameterized by one of the surface parameters while the other surface parameter is fixed. Isolines provide a simple means to display a surface. By fixing the u parameter of surface s(u,v), the iso-u lines of the form c(v) = s(u0,v) are produced, and by fixing the v parameter, the iso-v lines of the form c(u) = s(u,v0) are produced.

When a function surface plot is being done without the removal of hidden lines, **set samples** controls the number of points sampled along each isoline; see **set samples (p. 125)** and **set hidden3d (p. 101)**. The contour algorithm assumes that a function sample occurs at each isoline intersection, so change in **samples** as well as **isosamples** may be desired when changing the resolution of a function surface/contour.

75.30 Key

The **set key** command enables a key (or legend) describing plots on a plot.

The contents of the key, i.e., the names given to each plotted data set and function and samples of the lines and/or symbols used to represent them, are determined by the **title** and **with** options of the {s}**plot** command. Please see **plot title (p. 76)** and **plot with (p. 77)** for more information.

Syntax:

```
set key {on|off} {default}
        {{inside | outside} | {lmargin | rmargin | tmargin | bmargin}
          | {at <position>}}
        {left | right | center} {top | bottom | center}
        {vertical | horizontal} {Left | Right}
        {{no}reverse} {{no}invert}
        {samplen <sample_length>} {spacing <vertical_spacing>}
        {width <width_increment>}
        {height <height_increment>}
        {{no}autotitle {columnheader}}
        {title "<text>"} {{no}enhanced}
        {font "<face>,<size>"} {textcolor <colorspec>}
        {{no}box { {linestyle | ls <line_style>}
                   | {linetype | lt <line_type>}
                     {linewidth | lw <line_width>}}}
unset key
show key
```

The key contains a title and a sample (line, point, box) for each plot in the graph. The key may be turned off by requesting **set key off** or **unset key**. Individual key entries may be turned off by using the **notitle** keyword in the corresponding plot command.

Elements within the key are stacked according to **vertical** or **horizontal**. In the case of **vertical**, the key occupies as few columns as possible. That is, elements are aligned in a column until running out of vertical space at which point a new column is started. In the case of **horizontal**, the key occupies as few rows as possible.

By default the key is placed in the upper right inside corner of the graph. The keywords **left**, **right**, **top**, **bottom**, **center**, **inside**, **outside**, **lmargin**, **rmargin**, **tmargin**, **bmargin** (, **above**, **over**, **below** and **under**) may be used to automatically place the key in other positions of the graph. Also an **at** <**position**> may be given to indicate precisely where the plot should be placed. In this case, the keywords **left**, **right**, **top**, **bottom** and **center** serve an analogous purpose for alignment. For more information, see **key placement (p. 104)**.

Justification of the plot titles within the key is controlled by **Left** or **Right** (default). The text and sample can be reversed (**reverse**) and a box can be drawn around the key (**box {...}**) in a specified **linetype** and **linewidth**, or a user-defined **linestyle**.

By default the first plot label is at the top of the key and successive labels are entered below it. The **invert** option causes the first label to be placed at the bottom of the key, with successive labels entered above it. This option is useful to force the vertical ordering of labels in the key to match the order of box types in a stacked histogram.

The <height_increment> is a number of character heights to be added to or subtracted from the height of the key box. This is useful mainly when you are putting a box around the key and want larger borders around the key entries.

All plotted curves of **plot**s and **splot**s are titled according to the default option **autotitles**. The automatic generation of titles can be suppressed by **noautotitles**; then only those titles explicitly defined by **(s)plot ... title ...** will be drawn.

The command **set key autotitle columnheader** causes the first entry in each column of input data to be interpreted as a text string and used as a title for the corresponding plot. If the quantity being plotted is a

function of data from several columns, gnuplot may be confused as to which column to draw the title from. In this case it is necessary to specify the column explicitly in the plot command, e.g.

```
plot "datafile" using (($2+$3)/$4) title columnhead(3) with lines
```

An overall title can be put on the key (**title "<text>"**) — see also **syntax (p. 35)** for the distinction between text in single- or double-quotes. The key title uses the same justification as do the plot titles.

The defaults for **set key** are **on**, **right**, **top**, **vertical**, **Right**, **noreverse**, **noinvert**, **samplen 4**, **spacing 1.25**, **title ""**, and **nobox**. The default <linetype> is the same as that used for the plot borders. Entering **set key default** returns the key to its default configuration.

The key is drawn as a sequence of lines, with one plot described on each line. On the right-hand side (or the left-hand side, if **reverse** is selected) of each line is a representation that attempts to mimic the way the curve is plotted. On the other side of each line is the text description (the line title), obtained from the **plot** command. The lines are vertically arranged so that an imaginary straight line divides the left- and right-hand sides of the key. It is the coordinates of the top of this line that are specified with the **set key** command. In a **plot**, only the x and y coordinates are used to specify the line position. For a **splot**, x, y and z are all used as a 3D location mapped using the same mapping as the graph itself to form the required 2D screen position of the imaginary line.

When using the TeX or PostScript drivers, or similar drivers where formatting information is embedded in the string, **gnuplot** is unable to calculate correctly the width of the string for key positioning. If the key is to be positioned at the left, it may be convenient to use the combination **set key left Left reverse**. The box and gap in the grid will be the width of the literal string.

If **splot** is being used to draw contours, the contour labels will be listed in the key. If the alignment of these labels is poor or a different number of decimal places is desired, the label format can be specified. See **set clabel (p. 88)** for details.

Examples:

This places the key at the default location:

```
set key default
```

This disables the key:

```
unset key
```

This places a key at coordinates 2,3.5,2 in the default (first) coordinate system:

```
set key at 2,3.5,2
```

This places the key below the graph:

```
set key below
```

This places the key in the bottom left corner, left-justifies the text, gives it a title, and draws a box around it in linetype 3:

```
set key left bottom Left title 'Legend' box 3
```

75.30.1 Key placement

To understand positioning, the best concept is to think of a region, i.e., inside/outside, or one of the margins. Along with the region, keywords **left/center/right** (l/c/r) and **top/center/bottom** (t/c/b) control where within the particular region the key should be placed.

When in **inside** mode, the keywords **left** (l), **right** (r), **top** (t), **bottom** (b), and **center** (c) push the key out toward the plot boundary as illustrated:

```
t/l   t/c   t/r

c/l    c    c/r

b/l   b/c   b/r
```

When in **outside** mode, automatic placement is similar to the above illustration, but with respect to the view, rather than the graph boundary. That is, a border is moved inward to make room for the key outside of the plotting area, although this may interfere with other labels and may cause an error on some devices. The

particular plot border that is moved depends upon the position described above and the stacking direction. For options centered in one of the dimensions, there is no ambiguity about which border to move. For the corners, when the stack direction is **vertical**, the left or right border is moved inward appropriately. When the stack direction is **horizontal**, the top or bottom border is moved inward appropriately.

The margin syntax allows automatic placement of key regardless of stack direction. When one of the margins **lmargin** (lm), **rmargin** (rm), **tmargin** (tm), and **bmargin** (bm) is combined with a single, non-conflicting direction keyword, the following illustrated positions may contain the key:

```
     l/tm  c/tm  r/tm

t/lm                  t/rm

c/lm                  c/rm

b/lm                  b/rm

     l/bm  c/bm  r/bm
```

Keywords **above** and **over** are synonymous with **tmargin**. For version compatibility, **above** or **over** without an additional l/c/r or stack direction keyword uses **center** and **horizontal**. Keywords **below** and **under** are synonymous with **bmargin**. For compatibility, **below** or **under** without an additional l/c/r or stack direction keyword uses **center** and **horizontal**. A further compatibility issue is that **outside** appearing without an additional t/b/c or stack direction keyword uses **top**, **right** and **vertical** (i.e., the same as t/rm above).

The <position> can be a simple x,y,z as in previous versions, but these can be preceded by one of five keywords (**first**, **second**, **graph**, **screen**, **character**) which selects the coordinate system in which the position of the first sample line is specified. See **coordinates (p. 19)** for more details. The effect of **left**, **right**, **top**, **bottom**, and **center** when <position> is given is to align the key as though it were text positioned using the label command, i.e., **left** means left align with key to the right of <position>, etc.

75.30.2 Key samples

By default, each plot on the graph generates a corresponding entry in the key. This entry contains a plot title and a sample line/point/box of the same color and fill properties as used in the plot itself. The font and textcolor properties control the appearance of the individual plot titles that appear in the key. Setting the textcolor to "rgb variable" causes the text for each key entry to be the same color as the line or fill color for that plot. This was the default in some earlier versions of gnuplot.

The length of the sample line can be controlled by **samplen**. The sample length is computed as the sum of the tic length and <sample_length> times the character width. **samplen** also affects the positions of point samples in the key since these are drawn at the midpoint of the sample line, even if the sample line itself is not drawn.

The vertical spacing between lines is controlled by **spacing**. The spacing is set equal to the product of the pointsize, the vertical tic size, and <vertical_spacing>. The program will guarantee that the vertical spacing is no smaller than the character height.

The <width_increment> is a number of character widths to be added to or subtracted from the length of the string. This is useful only when you are putting a box around the key and you are using control characters in the text. **gnuplot** simply counts the number of characters in the string when computing the box width; this allows you to correct it.

75.31 Label

Arbitrary labels can be placed on the plot using the **set label** command.

Syntax:

```
set label {<tag>} {"<label text>"} {at <position>}
          {left | center | right}
          {norotate | rotate {by <degrees>}}
          {font "<name>{,<size>}"}
          {noenhanced}
          {front | back}
          {textcolor <colorspec>}
```

```
                {point <pointstyle> | nopoint}
                {offset <offset>}
     unset label {<tag>}
     show label
```

The <position> is specified by either x,y or x,y,z, and may be preceded by **first**, **second**, **graph**, **screen**, or **character** to select the coordinate system. See **coordinates (p. 19)** for details.

The tag is an integer that is used to identify the label. If no <tag> is given, the lowest unused tag value is assigned automatically. The tag can be used to delete or modify a specific label. To change any attribute of an existing label, use the **set label** command with the appropriate tag, and specify the parts of the label to be changed.

The <label text> can be a string constant, a string variable, or a string- valued expression. See **strings (p. 33)**, **sprintf (p. 23)**, and **gprintf (p. 98)**.

By default, the text is placed flush left against the point x,y,z. To adjust the way the label is positioned with respect to the point x,y,z, add the justification parameter, which may be **left**, **right** or **center**, indicating that the point is to be at the left, right or center of the text. Labels outside the plotted boundaries are permitted but may interfere with axis labels or other text.

If **rotate** is given, the label is written vertically (if the terminal can do so, of course). If **rotate by <degrees>** is given, conforming terminals will try to write the text at the specified angle; non-conforming terminals will treat this as vertical text.

Font and its size can be chosen explicitly by **font "<name>{,<size>}"** if the terminal supports font settings. Otherwise the default font of the terminal will be used.

Normally the enhanced text mode string interpretation, if enabled for the current terminal, is applied to all text strings including label text. The **noenhanced** property can be used to exempt a specific label from the enhanced text mode processing. The can be useful if the label contains underscores, for example. See **enhanced text (p. 20)**.

If **front** is given, the label is written on top of the graphed data. If **back** is given (the default), the label is written underneath the graphed data. Using **front** will prevent a label from being obscured by dense data.

textcolor <colorspec> changes the color of the label text. <colorspec> can be a linetype, an rgb color, or a palette mapping. See help for **colorspec (p. 29)** and **palette (p. 119)**. **textcolor** may be abbreviated **tc**.

```
  `tc default` resets the text color to its default state.
  `tc lt <n>` sets the text color to that of line type <n>.
  `tc ls <n>` sets the text color to that of line style <n>.
  `tc palette z` selects a palette color corresponding to the label z position.
  `tc palette cb <val>` selects a color corresponding to <val> on the colorbar.
  `tc palette fraction <val>`, with 0<=val<=1, selects a color corresponding to
      the mapping [0:1] to grays/colors of the `palette`.
  `tc rgb "#RRGGBB"` selects an arbitrary 24-bit RGB color.
```

If a <pointstyle> is given, using keywords **lt**, **pt** and **ps**, see **style (p. 77)**, a point with the given style and color of the given line type is plotted at the label position and the text of the label is displaced slightly. This option is used by default for placing labels in **mouse** enhanced terminals. Use **nopoint** to turn off the drawing of a point near the label (this is the default).

The displacement defaults to 1,1 in **pointsize** units if a <pointstyle> is given, 0,0 if no <pointstyle> is given. The displacement can be controlled by the optional **offset <offset>** where <offset> is specified by either x,y or x,y,z, and may be preceded by **first**, **second**, **graph**, **screen**, or **character** to select the coordinate system. See **coordinates (p. 19)** for details.

If one (or more) axis is timeseries, the appropriate coordinate should be given as a quoted time string according to the **timefmt** format string. See **set xdata (p. 136)** and **set timefmt (p. 132)**.

The EEPIC, Imagen, LaTeX, and TPIC drivers allow \\ in a string to specify a newline.

Label coordinates and text can also be read from a data file (see **labels (p. 46)**).

Examples:

To set a label at (1,2) to "y=x", use:

```
     set label "y=x" at 1,2
```

To set a Sigma of size 24, from the Symbol font set, at the center of the graph, use:

```
     set label "S" at graph 0.5,0.5 center font "Symbol,24"
```

To set a label "y=x^2" with the right of the text at (2,3,4), and tag the label as number 3, use:

```
set label 3 "y=x^2" at 2,3,4 right
```

To change the preceding label to center justification, use:

```
set label 3 center
```

To delete label number 2, use:

```
unset label 2
```

To delete all labels, use:

```
unset label
```

To show all labels (in tag order), use:

```
show label
```

To set a label on a graph with a timeseries on the x axis, use, for example:

```
set timefmt "%d/%m/%y,%H:%M"
set label "Harvest" at "25/8/93",1
```

To display a freshly fitted parameter on the plot with the data and the fitted function, do this after the **fit**, but before the **plot**:

```
set label sprintf("a = %3.5g",par_a) at 30,15
bfit = gprintf("b = %s*10^%S",par_b)
set label bfit at 30,20
```

To display a function definition along with its fitted parameters, use:

```
f(x)=a+b*x
fit f(x) 'datafile' via a,b
set label GPFUN_f at graph .05,.95
set label sprintf("a = %g", a) at graph .05,.90
set label sprintf("b = %g", b) at graph .05,.85
```

To set a label displaced a little bit from a small point:

```
set label 'origin' at 0,0 point lt 1 pt 2 ps 3 offset 1,-1
```

To set a label whose color matches the z value (in this case 5.5) of some point on a 3D splot colored using pm3d:

```
set label 'text' at 0,0,5.5 tc palette z
```

75.32 Lmargin

The command **set lmargin** sets the size of the left margin. Please see **set margin (p. 109)** for details.

75.33 Loadpath

The **loadpath** setting defines additional locations for data and command files searched by the **call**, **load**, **plot** and **splot** commands. If a file cannot be found in the current directory, the directories in **loadpath** are tried.

Syntax:

```
set loadpath {"pathlist1" {"pathlist2"...}}
show loadpath
```

Path names may be entered as single directory names, or as a list of path names separated by a platform-specific path separator, eg. colon (':') on Unix, semicolon (';') on DOS/Windows/OS/2/Amiga platforms. The **show loadpath**, **save** and **save set** commands replace the platform-specific separator with a space character (' ') for maximum portability.

If the environment variable GNUPLOT_LIB is set, its contents are appended to **loadpath**. However, **show loadpath** prints the contents of user defined loadpath and system loadpath separately. Also, the **save** and **save set** commands save only the user specified parts of **loadpath**, for portability reasons.

75.34 Locale

The **locale** setting determines the language with which **{x,y,z}{d,m}tics** will write the days and months.

Syntax:

```
set locale {"<locale>"}
```

<locale> may be any language designation acceptable to your installation. See your system documentation for the available options. The command **set locale** "" will try to determine the locale from the LC_TIME, LC_ALL, or LANG environment variables.

To change the decimal point locale, see **set decimalsign (p. 94)**. To change the character encoding to the current locale, see **set encoding (p. 96)**.

75.35 Logscale

Syntax:

```
set logscale <axes> <base>
unset logscale <axes>
show logscale
```

where <axes> may be any combinations of **x**, **x2**, **y**, **y2**, **z**, and **cb** in any order, and where <base> is the base of the log scaling. If <base> is not given, then 10 is assumed. If <axes> is not given, then all axes are assumed. The command **unset logscale** turns off log scaling for the specified axes.

Examples:

To enable log scaling in both x and z axes:

```
set logscale xz
```

To enable scaling log base 2 of the y axis:

```
set logscale y 2
```

To enable z and color log axes for a pm3d plot:

```
set logscale zcb
```

To disable z axis log scaling:

```
unset logscale z
```

75.36 Macros

If command line macro substitution is enabled, then tokens in the command line of the form @<stringvariablename> will be replaced by the text string contained in <stringvariablename>. See **substitution (p. 34)**.

Syntax:

```
set macros
```

75.37 Mapping

If data are provided to **splot** in spherical or cylindrical coordinates, the **set mapping** command should be used to instruct **gnuplot** how to interpret them.

Syntax:

```
set mapping {cartesian | spherical | cylindrical}
```

A cartesian coordinate system is used by default.

For a spherical coordinate system, the data occupy two or three columns (or **using** entries). The first two are interpreted as the azimuthal and polar angles theta and phi (or "longitude" and "latitude"), in the units specified by **set angles**. The radius r is taken from the third column if there is one, or is set to unity if there is no third column. The mapping is:

```
x = r * cos(theta) * cos(phi)
y = r * sin(theta) * cos(phi)
z = r * sin(phi)
```

Note that this is a "geographic" spherical system, rather than a "polar" one (that is, phi is measured from the equator, rather than the pole).

For a cylindrical coordinate system, the data again occupy two or three columns. The first two are interpreted as theta (in the units specified by **set angles**) and z. The radius is either taken from the third column or set to unity, as in the spherical case. The mapping is:

```
x = r * cos(theta)
y = r * sin(theta)
z = z
```

The effects of **mapping** can be duplicated with the **using** filter on the **splot** command, but **mapping** may be more convenient if many data files are to be processed. However even if **mapping** is used, **using** may still be necessary if the data in the file are not in the required order.

mapping has no effect on **plot**.

```
world.dem: mapping demos.
```

75.38 Margin

The computed margins can be overridden by the **set margin** commands. **show margin** shows the current settings.

Syntax:

```
set bmargin {{at screen} <margin>}
set lmargin {{at screen} <margin>}
set rmargin {{at screen} <margin>}
set tmargin {{at screen} <margin>}
show margin
```

The default units of <margin> are character heights or widths, as appropriate. A positive value defines the absolute size of the margin. A negative value (or none) causes **gnuplot** to revert to the computed value. For 3D plots, only the left margin can be set using character units.

The keywords **at screen** indicates that the margin is specified as a fraction of the full drawing area. This can be used to precisely line up the corners of individual 2D and 3D graphs in a multiplot. This placement ignores the current values of **set origin** and **set size**, and is intended as an alternative method for positioning graphs within a multiplot.

Normally the margins of a plot are automatically calculated based on tics, tic labels, axis labels, the plot title, the timestamp and the size of the key if it is outside the borders. If, however, tics are attached to the axes (**set xtics axis**, for example), neither the tics themselves nor their labels will be included in either the margin calculation or the calculation of the positions of other text to be written in the margin. This can lead to tic labels overwriting other text if the axis is very close to the border.

75.39 Mouse

The command **set mouse** enables mouse actions. Currently the pm, x11, ggi, windows and wxt terminals are mouse enhanced. There are two mouse modes. The 2d-graph mode works for 2d graphs and for maps (i.e. splots with **set view** having z-rotation 0, 90, 180, 270 or 360 degrees, including **set view map**) and it allows tracing the position over graph, zooming, annotating graph etc. For 3d graphs **splot**, the view and scaling of the graph can be changed with mouse buttons 1 and 2. If additionally to these buttons the modifier <ctrl> is hold down, the coordinate system only is rotated which is useful for large data sets. A vertical motion of Button 2 with the shift key hold down changes the **xyplane**.

Mousing is not available in multiplot mode. When multiplot is finished using **unset multiplot**, then the mouse will be turned on again and acts on the last plot (like replot does).

Syntax:

```
set mouse {doubleclick <ms>} {nodoubleclick} \
```

```
              {{no}zoomcoordinates} \
              {noruler | ruler {at x,y}} \
              {polardistance{deg|tan} | nopolardistance} \
              {format <string>} \
              {clipboardformat <int>/<string>} \
              {mouseformat <int>/<string>} \
              {{no}labels {"labeloptions"}} \
              {{no}zoomjump} {{no}verbose}
     unset mouse
```

The doubleclick resolution is given in milliseconds and used for Button 1 which copies the current mouse position to the **clipboard**. If you want that to be done by single clicking a value of 0 ms can be used. The default value is 300 ms.

The option **zoomcoordinates** determines if the coordinates of the zoom box are drawn at the edges while zooming. This is on by default.

The options **noruler** and **ruler** switch the ruler off and on, the latter optionally setting the origin at the given coordinates. While the ruler is on, the distance in user units from the ruler origin to the mouse is displayed continuously. By default, toggling the ruler has the key binding 'r'.

The option **polardistance** determines if the distance between the mouse cursor and the ruler is also shown in polar coordinates (distance and angle in degrees or tangent (slope)). This corresponds to the default key binding '5'.

The **format** option takes a fprintf like format string which determines how floating point numbers are printed to the drivers window and the clipboard. The default is "% #g".

clipboardformat and **mouseformat** are used for formatting the text on Button1 and Button2 actions – copying the coordinates to the clipboard and temporarily annotating the mouse position. This corresponds to the key bindings '1', '2', '3', '4' (see the driver's documentation). If the argument is a string this string is used as c format specifier and should contain two float specifiers, e.g. **set mouse mouseformat "mouse = %5.2g, %10.2f"**. Use **set mouse mouseformat ""** to turn this string off again.

The following formats are available (format 6 may only be selected if the format string was specified already):

```
0   real coordinates in  brackets e.g. [1.23, 2.45]
1   real coordinates w/o brackets e.g.  1.23, 2.45
2   x == timefmt                        [(as set by `set timefmt`), 2.45]
3   x == date                           [31. 12. 1999, 2.45]
4   x == time                           [23:59, 2.45]
5   x == date / time                    [31. 12. 1999 23:59, 2.45]
6   alt. format, specified as string    ""
```

Choose the option **labels** to define persistent gnuplot labels using Button 2. The default is **nolabels**, which makes Button 2 draw only a temporary label at the mouse position. Labels are drawn with the current setting of **mouseformat**. The **labeloptions** string is passed to the **set label** command. The default is "point pointstyle 1" which will plot a small plus at the label position. Temporary labels will disappear at the next **replot** or mouse zoom operation. Persistent labels can be removed by holding the Ctrl-Key down while clicking Button 2 on the label's point. The threshold for how close you must be to the label is also determined by the **pointsize**.

If the option **zoomjump** is on, the mouse pointer will be automatically offset a small distance after starting a zoom region with button 3. This can be useful to avoid a tiny (or even empty) zoom region. **zoomjump** is off by default.

If the option **verbose** is turned on the communication commands are shown during execution. This option can also be toggled by hitting **6** in the driver's window. **verbose** is off by default.

Press 'h' in the driver's window for a short summary of the mouse and key bindings. This will also display user defined bindings or **hotkeys** which can be defined using the **bind** command, see help for **bind** **(p. 31)**. Note, that user defined **hotkeys** may override the default bindings.

Press 'q' in the driver's window to close the window. This key cannot be overridden with the **bind** command.

See also help for **bind (p. 31)** and **label (p. 105)**.

75.39.1 X11 mouse

If multiple X11 plot windows have been opened using the **set term x11 <n>** terminal option, then only the current plot window supports the entire range of mouse commands and hotkeys. The other windows will, however, continue to display mouse coordinates at the lower left.

For consistency with other screen terminals, X11 mouse support is turned on by default, wherever the standard input comes from. However, on some UNIX flavors, special input devices as /dev/null might not be **select-able**; using such devices with the mouse turned on will hang gnuplot. Please turn off mousing with **unset mouse** if you are in this situation.

75.40 Multiplot

The command **set multiplot** places **gnuplot** in the multiplot mode, in which several plots are placed on the same page, window, or screen.

Syntax:

```
set multiplot { layout <rows>,<cols>
                {rowsfirst|columnsfirst} {downwards|upwards}
                {title <page title>}
                {scale <xscale>{,<yscale>}} {offset <xoff>{,<yoff>}}
               }
unset multiplot
```

For some terminals, no plot is displayed until the command **unset multiplot** is given, which causes the entire page to be drawn and then returns gnuplot to its normal single-plot mode. For other terminals, each separate **plot** command produces an updated display, either by redrawing all previous ones and the newly added plot, or by just adding the new plot to the existing display.

The area to be used by the next plot is not erased before doing the new plot. The **clear** command can be used to do this if wanted, as is typically the case for "inset" plots.

Any labels or arrows that have been defined will be drawn for each plot according to the current size and origin (unless their coordinates are defined in the **screen** system). Just about everything else that can be **set** is applied to each plot, too. If you want something to appear only once on the page, for instance a single time stamp, you'll need to put a **set time**/**unset time** pair around one of the **plot**, **splot** or **replot** commands within the **set multiplot**/**unset multiplot** block.

The multiplot title is separate from the individual plot titles, if any. Space is reserved for it at the top of the page, spanning the full width of the canvas.

The commands **set origin** and **set size** must be used to correctly position each plot if no layout is specified or if fine tuning is desired. See **set origin** **(p. 115)** and **set size** **(p. 125)** for details of their usage.

Example:

```
set multiplot
set size 0.4,0.4
set origin 0.1,0.1
plot sin(x)
set size 0.2,0.2
set origin 0.5,0.5
plot cos(x)
unset multiplot
```

This displays a plot of cos(x) stacked above a plot of sin(x).

set size and **set origin** refer to the entire plotting area used for each plot. Please also see **set term size** **(p. 16)**. If you want to have the axes themselves line up, you can guarantee that the margins are the same size with the **set margin** commands. See **set margin** **(p. 109)** for their use. Note that the margin settings are absolute, in character units, so the appearance of the graph in the remaining space will depend on the screen size of the display device, e.g., perhaps quite different on a video display and a printer.

With the **layout** option you can generate simple multiplots without having to give the **set size** and **set origin** commands before each plot: Those are generated automatically, but can be overridden at any time. With **layout** the display will be divided by a grid with <rows> rows and <cols> columns. This grid is filled rows first or columns first depending on whether the corresponding option is given in the multiplot command. The stack of plots can grow **downwards** or **upwards**. Default is **rowsfirst** and **downwards**.

Each plot can be scaled by **scale** and shifted with **offset**; if the y-values for scale or offset are omitted, the x-value will be used. **unset multiplot** will turn off the automatic layout and restore the values of **set size** and **set origin** as they were before **set multiplot layout**.

Example:

```
set size 1,1
set origin 0,0
set multiplot layout 3,2 columnsfirst scale 1.1,0.9
[ up to 6 plot commands here ]
unset multiplot
```

The above example will produce 6 plots in 2 columns filled top to bottom, left to right. Each plot will have a horizontal size of 1.1/2 and a vertical size of 0.9/3.

See also

multiplot demo (multiplt.dem)

75.41 Mx2tics

Minor tic marks along the x2 (top) axis are controlled by **set mx2tics**. Please see **set mxtics (p. 112)**.

75.42 Mxtics

Minor tic marks along the x axis are controlled by **set mxtics**. They can be turned off with **unset mxtics**. Similar commands control minor tics along the other axes.

Syntax:

```
set mxtics {<freq> | default}
unset mxtics
show mxtics
```

The same syntax applies to **mytics**, **mztics**, **mx2tics**, **my2tics** and **mcbtics**.

<freq> is the number of sub-intervals (NOT the number of minor tics) between major tics (the default for a linear axis is either two or five depending on the major tics, so there are one or four minor tics between major tics). Selecting **default** will return the number of minor ticks to its default value.

If the axis is logarithmic, the number of sub-intervals will be set to a reasonable number by default (based upon the length of a decade). This will be overridden if <freq> is given. However the usual minor tics (2, 3, ..., 8, 9 between 1 and 10, for example) are obtained by setting <freq> to 10, even though there are but nine sub-intervals.

To set minor tics at arbitrary positions, use the ("<label>" <pos> <level>, ...) form of **set {x|x2|y|y2|z}tics** with <label> empty and <level> set to 1.

The **set m{x|x2|y|y2|z}tics** commands work only when there are uniformly spaced major tics. If all major tics were placed explicitly by **set {x|x2|y|y2|z}tics**, then minor tic commands are ignored. Implicit major tics and explicit minor tics can be combined using **set {x|x2|y|y2|z}tics** and **set {x|x2|y|y2|z}tics add**.

Examples:

```
set xtics 0, 5, 10
set xtics add (7.5)
set mxtics 5
```

Major tics at 0,5,7.5,10, minor tics at 1,2,3,4,6,7,8,9

```
set logscale y
set ytics format ""
set ytics 1e-6, 10, 1
set ytics add ("1" 1, ".1" 0.1, ".01" 0.01, "10^-3" 0.001, \
               "10^-4" 0.0001)
set mytics 10
```

Major tics with special formatting, minor tics at log positions

By default, minor tics are off for linear axes and on for logarithmic axes. They inherit the settings for **axis|border** and **{no}mirror** specified for the major tics. Please see **set xtics (p. 139)** for information about these.

75.43 My2tics

Minor tic marks along the y2 (right-hand) axis are controlled by **set my2tics**. Please see **set mxtics (p. 112)**.

75.44 Mytics

Minor tic marks along the y axis are controlled by **set mytics**. Please see **set mxtics (p. 112)**.

75.45 Mztics

Minor tic marks along the z axis are controlled by **set mztics**. Please see **set mxtics (p. 112)**.

75.46 Object

The **set object** command defines a single object which will appear in all subsequent 2D plots. You may define as many objects as you like. Currently the supported object types are **rectangle**, **circle**, **ellipse**, and **polygon**. Rectangles inherit a default set of style properties (fill, color, border) from those set by the command **set style rectangle**, but each object can also be given individual style properties. Circles, ellipses, and polygons inherit the fill style from **set style fill**.

Syntax:

```
set object <index>
    <object-type> <object-properties>
    {front|back|behind} {fc|fillcolor <colorspec>} {fs <fillstyle>}
    {default} {lw|linewidth <width>}
```

<object-type> is either **rectangle**, **ellipse**, **circle**, or **polygon**. Each object type has its own set of characteristic properties.

Setting **front** will draw the object in front of all plot elements, but behind any labels that are also marked **front**. Setting **back** will place the object behind all plot curves and labels. Setting **behind** will place the object behind everything including the axes and **back** rectangles, thus

```
set object rectangle from screen 0,0 to screen 1,1 behind
```

can be used to provide a colored background for the entire graph or page.

The fill color of the object is taken from the <colorspec>. **fillcolor** may be abbreviated **fc**. The fill style is taken from <fillstyle>. See **colorspec (p. 29)** and **fillstyle (p. 127)**. If the keyword **default** is given, these properties are inherited from the default settings at the time a plot is drawn. See **set style rectangle (p. 130)**.

75.46.1 Rectangle

Syntax:

```
set object <index> rectangle
    {from <position> {to|rto} <position> |
     center <position> size <w>,<h> |
     at <position> size <w>,<h>}
```

The position of the rectangle may be specified by giving the position of two diagonal corners (bottom left and top right) or by giving the position of the center followed by the width and the height. In either case the positions may be given in axis, graph, or screen coordinates. See **coordinates (p. 19)**. The options **at** and **center** are synonyms.

Examples:

```
# Force the entire area enclosed by the axes to have background color cyan
set object 1 rect from graph 0, graph 0 to graph 1, graph 1 back
set object 1 rect fc rgb "cyan" fillstyle solid 1.0

# Position a red square with lower left at 0,0 and upper right at 2,3
set object 2 rect from 0,0 to 2,3 fc lt 1
```

```
# Position an empty rectangle (no fill) with a blue border
set object 3 rect from 0,0 to 2,3 fs empty border rgb "blue"

# Return fill and color to the default style but leave vertices unchanged
set object 2 rect default
```

75.46.2 Ellipse

Syntax:

```
set object <index> ellipse {at|center} <position> size <w>,<h>
    {angle <orientation>}
    {<other-object-properties>}
```

The position of the ellipse is specified by giving the center followed by the width and the height (actually the major and minor axes). The keywords **at** and **center** are synonyms. The center position may be given in axis, graph, or screen coordinates. See **coordinates (p. 19)**. The major and minor axis lengths must be given in axis coordinates. The orientation of the ellipse is specified by the angle between the horizontal axis and the first axis of the ellipse. NB: If the x and y axis scales are not equal, then the major/minor axis ratio will no longer be correct after rotation. This may be fixed in a later gnuplot version.

Note that **set object ellipse size <2r>,<2r>** does not in general produce the same result as **set object circle <r>**. The circle radius is always interpreted in terms of units along the x axis, and will always produce a circle even if the x and y axis scales are different and even if the aspect ratio of your plot is not 1. 'Set object ellipse' interprets the first '2r' in terms of x axis units and the second '2r' in terms of y axis units. This will only produce a circle if the x and y axis scales are identical and the plot aspect ratio is 1.

75.46.3 Circle

Syntax:

```
set object <index> circle {at|center} <position> size <radius>
    {arc [<begin>:<end>]}
    {<other-object-properties>}
```

The position of the circle is specified by giving the position of the center center followed by the radius. The keywords **at** and **center** are synonyms. The position and radius may be given in x-axis, graph, or canvas coordinates. See **coordinates (p. 19)**. In all cases the radius is calculated relative to the horizontal scale of the axis, graph, or canvas. Any disparity between the horizontal and vertical scaling will be corrected for so that the result is always a circle.

By default a full circle is drawn. The optional qualifier **arc** specifies a starting angle and ending angle, in degrees, for one arc of the circle. The arc is always drawn counterclockwise.

75.46.4 Polygon

Syntax:

```
set object <index> polygon
    from <position> to <position> ... {to <position>}
```

or

```
    from <position> rto <position> ... {rto <position>}
```

The position of the polygon may be specified by giving the position of a sequence of vertices. These may be given in axis, graph, or screen coordinates. If relative coordinates are used (rto) then the coordinate type must match that of the previous vertex. See **coordinates (p. 19)**.

Example:

```
set object 1 polygon from 0,0 to 1,1 to 2,0
set object 1 fc rgb "cyan" fillstyle solid 1.0 border lt -1
```

75.47 Offsets

Offsets provide a mechanism to put an empty boundary around the data inside an autoscaled graph. The offsets only affect the x1 and y1 axes, and only in 2D **plot** commands.

Syntax:

```
set offsets <left>, <right>, <top>, <bottom>
unset offsets
show offsets
```

Each offset may be a constant or an expression. Each defaults to 0. By default, the left and right offsets are given in units of the first x axis, the top and bottom offsets in units of the first y axis. Alternatively, you may specify the offsets as a fraction of the total axis range by using the keyword "graph". A positive offset expands the axis range in the specified direction, e.g., a positive bottom offset makes ymin more negative. Negative offsets, while permitted, can have unexpected interactions with autoscaling and clipping. To prevent the auto-scaling from further adjusting your axis ranges, it is useful to also specify "set auto fix".

Example:

```
set auto fix
set offsets graph 0.05, 0, 2, 2
plot sin(x)
```

This graph of sin(x) will have a y range [-3:3] because the function will be autoscaled to [-1:1] and the vertical offsets are each two. The x range will be [-11:10] because the default is [-10:10] and it has been expanded to the left by 0.05 of that total range.

75.48 Origin

The **set origin** command is used to specify the origin of a plotting surface (i.e., the graph and its margins) on the screen. The coordinates are given in the **screen** coordinate system (see **coordinates (p. 19)** for information about this system).

Syntax:

```
set origin <x-origin>,<y-origin>
```

75.49 Output

By default, screens are displayed to the standard output. The **set output** command redirects the display to the specified file or device.

Syntax:

```
set output {"<filename>"}
show output
```

The filename must be enclosed in quotes. If the filename is omitted, any output file opened by a previous invocation of **set output** will be closed and new output will be sent to STDOUT. (If you give the command **set output "STDOUT"**, your output may be sent to a file named "STDOUT"! ["May be", not "will be", because some terminals, like **x11** or **wxt**, ignore **set output**.])

When both **set terminal** and **set output** are used together, it is safest to give **set terminal** first, because some terminals set a flag which is needed in some operating systems. This would be the case, for example, if the operating system needs to know whether or not a file is to be formatted in order to open it properly.

On machines with popen functions (Unix), output can be piped through a shell command if the first non-whitespace character of the filename is '|'. For instance,

```
set output "|lpr -Plaser filename"
set output "|lp -dlaser filename"
```

On MSDOS machines, **set output "PRN"** will direct the output to the default printer. On VMS, output can be sent directly to any spooled device. It is also possible to send the output to DECnet transparent tasks, which allows some flexibility.

75.50 Parametric

The **set parametric** command changes the meaning of **plot** (**splot**) from normal functions to parametric functions. The command **unset parametric** restores the plotting style to normal, single-valued expression plotting.

Syntax:

```
set parametric
unset parametric
show parametric
```

For 2D plotting, a parametric function is determined by a pair of parametric functions operating on a parameter. An example of a 2D parametric function would be **plot sin(t),cos(t)**, which draws a circle (if the aspect ratio is set correctly — see **set size** **(p. 125)**). **gnuplot** will display an error message if both functions are not provided for a parametric **plot**.

For 3D plotting, the surface is described as x=f(u,v), y=g(u,v), z=h(u,v). Therefore a triplet of functions is required. An example of a 3D parametric function would be **cos(u)*cos(v),cos(u)*sin(v),sin(u)**, which draws a sphere. **gnuplot** will display an error message if all three functions are not provided for a parametric **splot**.

The total set of possible plots is a superset of the simple f(x) style plots, since the two functions can describe the x and y values to be computed separately. In fact, plots of the type t,f(t) are equivalent to those produced with f(x) because the x values are computed using the identity function. Similarly, 3D plots of the type u,v,f(u,v) are equivalent to f(x,y).

Note that the order the parametric functions are specified is xfunction, yfunction (and zfunction) and that each operates over the common parametric domain.

Also, the **set parametric** function implies a new range of values. Whereas the normal f(x) and f(x,y) style plotting assume an xrange and yrange (and zrange), the parametric mode additionally specifies a trange, urange, and vrange. These ranges may be set directly with **set trange**, **set urange**, and **set vrange**, or by specifying the range on the **plot** or **splot** commands. Currently the default range for these parametric variables is [-5:5]. Setting the ranges to something more meaningful is expected.

75.51 Plot

The **show plot** command shows the current plotting command as it results from the last **plot** and/or **splot** and possible subsequent **replot** commands.

In addition, the **show plot add2history** command adds this current plot command into the **history**. It is useful if you have used **replot** to add more curves to the current plot and you want to edit the whole command now.

75.52 Pm3d

pm3d is an **splot** style for drawing palette-mapped 3d and 4d data as color/gray maps and surfaces. It uses a pm3d algorithm which allows plotting gridded as well as non-gridded data without preprocessing, even when the data scans do not have the same number of points.

Drawing of color surfaces is available on terminals supporting filled colored polygons with color mapping specified by **palette**. Currently supported terminals include

```
Screen terminals:
  OS/2 Presentation Manager
  X11
  Linux VGA (vgagl)
  GGI
  Windows
  AquaTerm (Mac OS X)
  wxWidgets (wxt)
Files:
  PostScript
  pslatex, pstex, epslatex
  gif, png, jpeg
```

```
(x)fig
tgif
cgm
pdf
svg
emf
```

Let us first describe how a map/surface is drawn. The input data come from an evaluated function or from an **splot data file**. Each surface consists of a sequence of separate scans (isolines). The pm3d algorithm fills the region between two neighbouring points in one scan with another two points in the next scan by a gray (or color) according to z-values (or according to an additional 'color' column, see help for **using (p. 71)**) of these 4 corners; by default the 4 corner values are averaged, but this can be changed by the option **corners2color**. In order to get a reasonable surface, the neighbouring scans should not cross and the number of points in the neighbouring scans should not differ too much; of course, the best plot is with scans having same number of points. There are no other requirements (e.g. the data need not be gridded). Another advantage is that the pm3d algorithm does not draw anything outside of the input (measured or calculated) region.

Surface coloring works with the following input data:

1. splot of function or of data file with one or three data columns: The gray/color scale is obtained by mapping the averaged (or **corners2color**) z-coordinate of the four corners of the above-specified quadrangle into the range [min_color_z,max_color_z] of **zrange** or **cbrange** providing a gray value in the range [0:1]. This value can be used directly as the gray for gray maps. The normalized gray value can be further mapped into a color — see **set palette (p. 119)** for the complete description.

2. splot of data file with two or four data columns: The gray/color value is obtained by using the last-column coordinate instead of the z-value, thus allowing the color and the z-coordinate be mutually independent. This can be used for 4d data drawing.

Other notes:

1. The term 'scan' referenced above is used more among physicists than the term 'iso_curve' referenced in gnuplot documentation and sources. You measure maps recorded one scan after another scan, that's why.

2. The 'gray' or 'color' scale is a linear mapping of a continuous variable onto a smoothly varying palette of colors. The mapping is shown in a rectangle next to the main plot. This documentation refers to this as a "colorbox", and refers to the indexing variable as lying on the colorbox axis. See **set colorbox (p. 90)**, **set cbrange (p. 144)**.

3. To use pm3d coloring to generate a two-dimensional plot rather than a 3D surface, use **set view map** or **set pm3d map**.

Syntax (the options can be given in any order):

```
set pm3d {
           { at <bst combination> }
           { interpolate <steps/points in scan, between scans> }
           { scansautomatic | scansforward | scansbackward | depthorder }
           { flush { begin | center | end } }
           { ftriangles | noftriangles }
           { clip1in | clip4in }
           { corners2color { mean|geomean|median|min|max|c1|c2|c3|c4 } }
           { hidden3d <linestyle> | nohidden3d }
           { implicit | explicit }
           { map }
         }
show pm3d
unset pm3d
```

Color surface is drawn if data or function **style** is set to pm3d globally or via 'with' option, or if the option **implicit** is on — then the pm3d surface is combined with the line surface mesh. See bottom of this section for mode details.

Color surface can be drawn at the base or top (then it is a gray/color planar map) or at z-coordinates of surface points (gray/color surface). This is defined by the **at** option with a string of up to 6 combinations of **b**, **t** and **s**. For instance, **at b** plots at bottom only, **at st** plots firstly surface and then top map, while **at bstbst** will never by seriously used.

Colored quadrangles are plotted one after another. When plotting surfaces (**at s**), the later quadrangles overlap

(overdraw) the previous ones. (Gnuplot is not virtual reality tool to calculate intersections of filled polygon meshes.) You may try to switch between **scansforward** and **scansbackward** to force the first scan of the data to be plotted first or last. The default is **scansautomatic** where gnuplot makes a guess about scans order. On the other hand, the **depthorder** option completely reorders the quadrangles. The rendering is performed after a depth sorting, which allows to visualize even complicated surfaces; see **pm3d depthorder (p. 119)** for more details.

If two subsequent scans do not have same number of points, then it has to be decided whether to start taking points for quadrangles from the beginning of both scans (**flush begin**), from their ends (**flush end**) or to center them (**flush center**). Note, that **flush (center|end)** are incompatible with **scansautomatic**: if you specify **flush center** or **flush end** and **scansautomatic** is set, it is silently switched to **scansforward**.

If two subsequent scans do not have the same number of points, the option **ftriangles** specifies whether color triangles are drawn at the scan tail(s) where there are not enough points in either of the scan. This can be used to draw a smooth map boundary.

Clipping with respect to x, y coordinates of quadrangles can be done in two ways. **clip1in**: all 4 points of each quadrangle must be defined and at least 1 point of the quadrangle must lie in the x and y ranges. **clip4in**: all 4 points of each quadrangle must lie in the x and y ranges.

There is a single gray/color value associated to each drawn pm3d quadrangle (no smooth color change among vertices). The value is calculated from z-coordinates from the surrounding corners according to **corners2color <option>**. The options 'mean' (default), 'geomean' and 'median' produce various kinds of surface color smoothing, while options 'min' and 'max' choose minimal or maximal value, respectively. This may not be desired for pixel images or for maps with sharp and intense peaks, in which case the options 'c1', 'c2', 'c3' or 'c4' can be used instead to assign the quadrangle color based on the z-coordinate of only one corner. Some experimentation may be needed to determine which corner corresponds to 'c1', as the orientation depends on the drawing direction. Because the pm3d algorithm does not extend the colored surface outside the range of the input data points, the 'c<j>' coloring options will result in pixels along two edges of the grid not contributing to the color of any quadrangle. For example, applying the pm3d algorithm to the 4x4 grid of data points in script **demo/pm3d.dem** (please have a look) produces only (4-1)x(4-1)=9 colored rectangles.

Another drawing algorithm, which would draw quadrangles around a given node by taking corners from averaged (x,y)-coordinates of its surrounding 4 nodes while using node's color, could be implemented in the future. This is already done for drawing images (2D grids) via **image** and **rgbimage** styles.

Notice that ranges of z-values and color-values for surfaces are adjustable independently by **set zrange**, **set cbrange**, as well as **set log** for z or cb. Maps can be adjusted by the cb-axis only; see also **set view map (p. 134)** and **set colorbox (p. 90)**.

The option **hidden3d** takes as the argument a linestyle which must be created by **set style line ...**. (The style need not to be present when setting pm3d, but it must be present when plotting). If set, lines are drawn using the specified line style, taking into account hidden line removal. This is by far more efficient than using the command **set hidden3d** as it doesn't really calculate hidden line removal, but just draws the filled polygons in the correct order. So the recommended choice when using pm3d is

```
set pm3d at s hidden3d 100
set style line 100 lt 5 lw 0.5
unset hidden3d
unset surf
splot x*x+y*y
```

There used to be an option {transparent|solid} to this command. Now you get the same effect from **set grid {front|layerdefault}**, respectively.

The **set pm3d map** is an abbreviation for **set pm3d at b**; **set view map**; **set style data pm3d**; **set style func pm3d**;. It is used for backwards compatibility, when **set view map** was not available. Take care that you properly use **zrange** and **cbrange** for input data point filtering and color range scaling, respectively; and also **set (no)surface** seems to have a (side?) effect.

The option **interpolate m,n** will interpolate grid points into a finer mesh, and color each quadrangle appropriately. For data files, this will smoothen the color surface, and enhance spikes in a color surface. For functions, interpolation makes little sense, except to trade off precision for memory. It would usually make more sense to use **samples** and **isosamples** when working with functions. For positive m and n, each quadrangle or triangle is interpolated m-times and n-times in the respective direction. For negative m and n, the interpolation frequency is chosen so that there will be at least |m| and |n| points drawn; you can consider this as a special gridding function. Zeros, i.e. **interpolation 0,0**, will automatically choose an optimal number of interpolated surface points.

The coloring setup as well as the color box drawing are determined by **set palette**. There can be only one palette for the current plot. Drawing of several surfaces with different palettes can be achieved by **multiplot** with fixed **origin** and **size**; don't forget to use **set palette maxcolors** when your terminal is running out of available colors.

On gnuplot start-up, mode is **explicit**. For historical and thus compatibility reasons, commands **set pm3d;** (i.e. no options) and **set pm3d at X ...** (i.e. **at** is the first option) sets mode **implicit**. Further, **set pm3d;** sets up the other options to their default.

If the option **implicit** is on, all surface plots will be plotted additionally to the default type, e.g.

```
splot 'fred.dat' with lines, 'lola.dat' with lines
```

would give both plots (meshes) additionally to a pm3d surface. That's what you are used to after **set pm3d;**.

If the option **explicit** is on (or **implicit** is off) only plots specified by the **with pm3d** attribute are plotted with a pm3d surface, e.g.:

```
splot 'fred.dat' with lines, 'lola.dat' with pm3d
```

would plot 'fred.dat' with lines (and only lines) and 'lola.dat' with a pm3d surface.

If you set the default data or function style to **pm3d**, e.g.:

```
set style data pm3d
```

then the options **implicit** and **explicit** have no effect.

Note that when plotting several plots, they are plotted in the order given on the command line. This can be of interest especially for filled surfaces which can overwrite and therefore hide part of earlier plots.

If **with pm3d** is specified in the **splot** command line, then it accepts the 'at' option. The following plots draw three color surfaces at different altitudes:

```
set border 4095
set pm3d at s
splot 10*x with pm3d at b, x*x-y*y, x*x+y*y with pm3d at t
```

See also help for **set palette (p. 119)**, **set cbrange (p. 144)**, **set colorbox (p. 90)**, **x11 pm3d (p. 196)** and definitely the demo file **demo/pm3d.dem**.

75.52.1 Depthorder

By default the quadrangles making up a pm3d solid surface are rendered in the order they are encountered along the surface grid points. This order may be controlled by the options **scansautomatic|scansforward|scansbackward**. These scan options are not in general compatible with hidden-surface removal.

Gnuplot does not do true hidden surface removal for solid surfaces, but often it is sufficient to render the component quadrangles in order from furthest to closest. This mode may be selected using the options

```
set pm3d depthorder hidden3d
```

The **depthorder** option orders the solid quadrangles; the **hidden3d** option similarly orders the bounding lines (if drawn). Note that the global option **set hidden3d** does not affect pm3d surfaces.

75.53 Palette

Palette is a color storage for use by **pm3d**, filled color contours or polygons, color histograms, color gradient background, and whatever it is or it will be implemented... Here it stands for a palette of smooth "continuous" colors or grays, but let's call it just a palette.

Color palettes require terminal entries for filled color polygons and palettes of smooth colors, are currently available for terminals listed in help for **set pm3d**. The range of color values are adjustable independently by **set cbrange** and **set log cb**. The whole color palette is visualized in the **colorbox**.

Syntax:

```
set palette
set palette {
           { gray | color }
```

```
                { gamma <gamma> }
                {   rgbformulae <r>,<g>,<b>
                  | defined { ( <gray1> <color1> {, <grayN> <colorN>}... ) }
                  | file '<filename>' {datafile-modifiers}
                  | functions <R>,<G>,<B>
                }
                { model { RGB | HSV | CMY | YIQ | XYZ } }
                { positive | negative }
                { nops_allcF | ps_allcF }
                { maxcolors <maxcolors> }
              }
     show palette
     show palette palette <n> {{float | int}}
     show palette gradient
     show palette fit2rgbformulae
     show palette rgbformulae
     show colornames
```

set palette (i.e. without options) sets up the default values. Otherwise, the options can be given in any order. **show palette** shows the current palette properties.

show palette gradient displays the gradient defining the palette (if appropriate). **show palette rgbformulae** prints the available fixed gray –> color transformation formulae. **show colornames** prints the known color names.

show palette palette <n> prints to the screen or to the file given by **set print** a table of RGB triplets calculated for the current palette settings and a palette having <n> discrete colors. The default wide table can be limited to 3 columns of r,g,b float values [0..1] or integer values [0..255] by options float or int, respectively. This way, the current gnuplot color palette can be loaded into other imaging applications, for example Octave. Additionally to this textual list of RGB table, you can use the **test palette** command to plot the R,G,B profiles for the current palette.

The following options determine the coloring properties.

Figure using this palette can be **gray** or **color**. For instance, in **pm3d** color surfaces the gray of each small spot is obtained by mapping the averaged z-coordinate of the 4 corners of surface quadrangles into the range [min_z,max_z] providing range of grays [0:1]. This value can be used directly as the gray for gray maps. The color map requires a transformation gray –> (R,G,B), i.e. a mapping [0:1] –> ([0:1],[0:1],[0:1]).

Basically two different types of mappings can be used: Analytic formulae to convert gray to color, or discrete mapping tables which are interpolated. **palette rgbformulae** and **palette functions** use analytic formulae whereas **palette defined** and **palette file** use interpolated tables. **palette rgbformulae** reduces the size of postscript output to a minimum.

The command **show palette fit2rgbformulae** finds the best matching **set palette rgbformulae** for the current **set palette**. Naturally, it makes sense to use it for non-rgbformulae palettes. This command can be found useful mainly for external programs using the same rgbformulae definition of palettes as gnuplot, like zimg (

http://zimg.sourceforge.net

).

set palette gray switches to a gray only palette. **set palette rgbformulae**, **set palette defined**, **set palette file** and **set palette functions** switch to a color mapping. **set palette color** is an easy way to switch back from the gray palette to the last color mapping.

Automatic gamma correction via **set palette gamma <gamma>** can be done for gray maps only (**set palette gray**). Linear mapping to gray is for gamma equals 1, see **test palette (p. 149)**. Gamma is ignored for color mappings.

Most terminals support only discrete number of colors (e.g. 256 colors in gif). All entries of the palette remaining after the default gnuplot linetype colors declaration are allocated for pm3d by default. Then **multiplot** could fail if there are no more color positions in the terminal available. Then you should use **set palette maxcolors <maxcolors>** with a reasonably small value. This option can also be used to separate levels of z=constant in discrete steps, thus to emulate filled contours. Default value of 0 stays for allocating all remaining entries in the terminal palette or for to use exact mapping to RGB.

RGB color space might not be the most useful color space to work in. For that reason you may change the

color space with **model** to one of **RGB**, **HSV**, **CMY**, **YIQ** and **XYZ**. Using color names for **set palette defined** tables and a color space other than RGB will result in funny colors. All explanation have been written for RGB color space, so please note, that **R** can be **H**, **C**, **Y**, or **X**, depending on the actual color space (**G** and **B** accordingly).

All values for all color spaces are limited to [0,1].

RGB stands for Red, Green and Blue; CMY stands for Cyan, Magenta and Yellow; HSV stands for Hue, Saturation, and Value; YIQ is the color model used by the U.S. Commercial Color Television Broadcasting, it is basically an RGB recoding with downward compatibility for black and white television; XYZ are the three primary colors of the color model defined by the 'Commission Internationale de l'Eclairage' (CIE). For more information on color models see:

http://en.wikipedia.org/wiki/Color_space

75.53.1 Rgbformulae

For **rgbformulae** three suitable mapping functions have to be chosen. This is done via **rgbformulae <r>,<g>,<b>**. The available mapping functions are listed by **show palette rgbformulae**. Default is **7,5,15**, some other examples are **3,11,6**, **21,23,3** or **3,23,21**. Negative numbers, like **3,-11,-6**, mean inverted color (i.e. 1-gray passed into the formula, see also **positive (p. 121)** and **negative (p. 121)** options below).

Some nice schemes in RGB color space

```
7,5,15   ... traditional pm3d (black-blue-red-yellow)
3,11,6   ... green-red-violet
23,28,3  ... ocean (green-blue-white); try also all other permutations
21,22,23 ... hot (black-red-yellow-white)
30,31,32 ... color printable on gray (black-blue-violet-yellow-white)
33,13,10 ... rainbow (blue-green-yellow-red)
34,35,36 ... AFM hot (black-red-yellow-white)
```

A full color palette in HSV color space

```
3,2,2    ... red-yellow-green-cyan-blue-magenta-red
```

Please note that even if called **rgbformulae** the formulas might actually determine the <H>,<S>,<V> or <X>,<Y>,<Z> or ... color components as usual.

Use **positive** and **negative** to invert the figure colors.

Note that it is possible to find a set of the best matching rgbformulae for any other color scheme by the command

```
show palette fit2rgbformulae
```

75.53.2 Defined

Gray-to-rgb mapping can be manually set by use of **palette defined**: A color gradient is defined and used to give the rgb values. Such a gradient is a piecewise linear mapping from gray values in [0,1] to the RGB space [0,1]x[0,1]x[0,1]. You have to specify the gray values and the corresponding RGB values in between a linear interpolation shall take place:

Syntax:

```
set palette  defined { ( <gray1> <color1> {, <grayN> <colorN>}... ) }
```

<grayX> are gray values which are mapped to [0,1] and <colorX> are the corresponding rgb colors. The color can be specified in three different ways:

```
<color> :=  { <r> <g> <b> | '<color-name>' | '#rrggbb' }
```

Either by three numbers (each in [0,1]) for red, green and blue, separated by whitespace, or the name of the color in quotes or X style color specifiers also in quotes. You may freely mix the three types in a gradient definition, but the named color "red" will be something strange if RGB is not selected as color space. Use **show colornames** for a list of known color names.

Please note, that even if written as <r>, this might actually be the <H> component in HSV color space or <X> in CIE-XYZ space, or ... depending on the selected color model.

The <gray> values have to form an ascending sequence of real numbers; the sequence will be automatically rescaled to [0,1].

set palette defined (without a gradient definition in braces) switches to RGB color space and uses a preset full-spectrum color gradient. Use **show palette gradient** to display the gradient.

Examples:

To produce a gray palette (useless but instructive) use:

```
set palette model RGB
set palette defined ( 0 "black", 1 "white" )
```

To produce a blue yellow red palette use (all equivalent):

```
set palette defined ( 0 "blue", 1 "yellow", 2 "red" )
set palette defined ( 0 0 0 1, 1 1 1 0, 2 1 0 0 )
set palette defined ( 0 "#0000ff", 1 "#ffff00", 2 "#ff0000" )
```

To produce some rainbow-like palette use:

```
set palette defined ( 0 "blue", 3 "green", 6 "yellow", 10 "red" )
```

Full color spectrum within HSV color space:

```
set palette model HSV
set palette defined ( 0 0 1 1, 1 1 1 1 )
set palette defined ( 0 0 1 0, 1 0 1 1, 6 0.8333 1 1, 7 0.8333 0 1)
```

To produce a palette with few colors only use:

```
set palette model RGB maxcolors 4
set palette defined ( 0 "blue", 1 "green", 2 "yellow", 3 "red" )
```

'Traffic light' palette (non-smooth color jumps at gray = 1/3 and 2/3).

```
set palette model RGB
set palette defined (0 "dark-green", 1 "green", 1 "yellow", \
                     2 "dark-yellow", 2 "red", 3 "dark-red" )
```

75.53.3 Functions

Use **set palette functions <Rexpr>, <Gexpr>, <Bexpr>** to define three formulae for the R(gray), G(gray) and B(gray) mapping. The three formulae may depend on the variable **gray** which will take values in [0,1] and should also produce values in [0,1]. Please note that <Rexpr> might be a formula for the H-value if HSV color space has been chosen (same for all other formulae and color spaces).

Examples:

To produce a full color palette use:

```
set palette model HSV functions gray, 1, 1
```

A nice black to gold palette:

```
set palette model XYZ functions gray**0.35, gray**0.5, gray**0.8
```

A gamma-corrected black and white palette

```
gamma = 2.2
color(gray) = gray**(1./gamma)
set palette model RGB functions color(gray), color(gray), color(gray)
```

75.53.4 File

set palette file is basically a **set palette defined (<gradient>)** where <gradient> is read from a datafile. Either 4 columns (gray,R,G,B) or just three columns (R,G,B) have to be selected via the **using** data file modifier. In the three column case, the line number will be used as gray. The gray range is automatically rescaled to [0,1]. The file is read as a normal data file, so all datafile modifiers can be used. Please note, that **R** might actually be e.g. **H** if HSV color space is selected.

As usual <filename> may be **'-'** which means that the data follow the command inline and are terminated by a single **e** on a line of its own.

Use **show palette gradient** to display the gradient.

Examples:

Read in a palette of RGB triples each in range [0,255]:

```
set palette file 'some-palette' using ($1/255):($2/255):($3/255)
```

Equidistant rainbow (blue-green-yellow-red) palette:

```
set palette model RGB file "-"
0 0 1
0 1 0
1 1 0
1 0 0
e
```

Binary palette files are supported as well, see **binary general (p. 64)**. Example: put 64 triplets of R,G,B doubles into file palette.bin and load it by

```
set palette file "palette.bin" binary record=64 using 1:2:3
```

75.53.5 Gamma correction

For gray mappings gamma correction can be turned on by **set palette gamma <gamma>**. <gamma> defaults to 1.5 which is quite suitable for most terminals.

For color mappings no automatic gamma correction is done by gnuplot. However, you may easily implement gamma correction. Here is an example for a gray scale image by use of explicit functions for the red, green and blue component with slightly different values of gamma

Example:

```
set palette model RGB
set palette functions gray**0.64, gray**0.67, gray**0.70
```

To use gamma correction with interpolated gradients specify intermediate gray values with appropriate colors. Instead of

```
set palette defined ( 0 0 0 0, 1 1 1 1 )
```

use e.g.

```
set palette defined ( 0 0 0 0, 0.5 .73 .73 .73, 1 1 1 1 )
```

or even more intermediate points until the linear interpolation fits the "gamma corrected" interpolation well enough.

75.53.6 Postscript

In order to reduce the size of postscript files, the gray value and not all three calculated r,g,b values are written to the file. Therefore the analytical formulae are coded directly in the postscript language as a header just before the pm3d drawing, see /g and /cF definitions. Usually, it makes sense to write therein definitions of only the 3 formulae used. But for multiplot or any other reason you may want to manually edit the transformations directly in the postscript file. This is the default option **nops_allcF**. Using the option **ps_allcF** writes postscript definitions of all formulae. This you may find interesting if you want to edit the postscript file in order to have different palettes for different surfaces in one graph. Well, you can achieve this functionality by **multiplot** with fixed **origin** and **size**.

If pm3d map has been plotted from gridded or almost regular data with an output to a postscript file, then it is possible to reduce the size of this postscript file up to at about 50% by the enclosed awk script **pm3dCompress.awk**. This you may find interesting if you intend to keep the file for including it into your publication or before downloading a very large file into a slow printer. Usage:

```
awk -f pm3dCompress.awk thefile.ps >smallerfile.ps
```

If pm3d map has been plotted from rectangular gridded data with an output to a postscript file, then it is possible to reduce the file size even more by the enclosed awk script **pm3dConvertToImage.awk**. Usage:

```
awk -f pm3dConvertToImage.awk <thefile.ps >smallerfile.ps
```

You may manually change the postscript output from gray to color and vice versa and change the definition of <maxcolors>.

75.54 Pointsize

The **set pointsize** command scales the size of the points used in plots.

Syntax:

```
set pointsize <multiplier>
show pointsize
```

The default is a multiplier of 1.0. Larger pointsizes may be useful to make points more visible in bitmapped graphics.

The pointsize of a single plot may be changed on the **plot** command. See **plot with (p. 77)** for details.

Please note that the pointsize setting is not supported by all terminal types.

75.55 Polar

The **set polar** command changes the meaning of the plot from rectangular coordinates to polar coordinates.

Syntax:

```
set polar
unset polar
show polar
```

In polar coordinates, the dummy variable (t) is an angle. The default range of t is [0:2*pi], or, if degree units have been selected, to [0:360] (see **set angles (p. 82)**).

The command **unset polar** changes the meaning of the plot back to the default rectangular coordinate system.

The **set polar** command is not supported for **splot**s. See the **set mapping (p. 108)** command for similar functionality for **splot (p. 145)**s.

While in polar coordinates the meaning of an expression in t is really r = f(t), where t is an angle of rotation. The trange controls the domain (the angle) of the function, and the x and y ranges control the range of the graph in the x and y directions. Each of these ranges, as well as the rrange, may be autoscaled or set explicitly. Ffor details of all the **ranges** commands, see **set xrange (p. 138)**.

Example:

```
set polar
plot t*sin(t)
plot [-2*pi:2*pi] [-3:3] [-3:3] t*sin(t)
```

The first **plot** uses the default polar angular domain of 0 to 2*pi. The radius and the size of the graph are scaled automatically. The second **plot** expands the domain, and restricts the size of the graph to [-3:3] in both directions.

You may want to **set size square** to have **gnuplot** try to make the aspect ratio equal to unity, so that circles look circular. See also

```
polar demos (polar.dem)
```

and

```
polar data plot (poldat.dem).
```

75.56 Print

The **set print** command redirects the output of the **print** command to a file.

Syntax:

```
set print
set print "-"
```

```
set print "<filename>"
set print "<filename>" append
set print "|<shell_command>"
```

Without "<filename>", the output file is restored to <STDERR>. The <filename> "-" means <STDOUT>. The **append** flag causes the file to be opened in append mode. A <filename> starting with "|" is opened as a pipe to the <shell_command> on platforms that support piping.

75.57 Rmargin

The command **set rmargin** sets the size of the right margin. Please see **set margin (p. 109)** for details.

75.58 Rrange

The **set rrange** command sets the range of the radial coordinate for a graph in polar mode. Please see **set xrange (p. 138)** for details.

75.59 Samples

The sampling rate of functions, or for interpolating data, may be changed by the **set samples** command.

Syntax:

```
set samples <samples_1> {,<samples_2>}
show samples
```

By default, sampling is set to 100 points. A higher sampling rate will produce more accurate plots, but will take longer. This parameter has no effect on data file plotting unless one of the interpolation/approximation options is used. See **plot smooth (p. 69)** re 2D data and **set cntrparam (p. 89)** and **set dgrid3d (p. 95)** re 3D data.

When a 2D graph is being done, only the value of <samples_1> is relevant.

When a surface plot is being done without the removal of hidden lines, the value of samples specifies the number of samples that are to be evaluated for the isolines. Each iso-v line will have <sample_1> samples and each iso-u line will have <sample_2> samples. If you only specify <samples_1>, <samples_2> will be set to the same value as <samples_1>. See also **set isosamples (p. 102)**.

75.60 Size

Syntax:

```
set size {{no}square | ratio <r> | noratio} {<xscale>,<yscale>}
show size
```

The <xscale> and <yscale> values are scale factors for the size of the plot, which includes the graph, labels, and margins.

Important note:

```
In earlier versions of gnuplot, some terminal types used the values from
`set size` to control also the size of the output canvas; others did not.
In version 4.4 almost all terminals now follow the following convention:
```

set term <terminal_type> size <XX>, <YY> controls the size of the output file, or **canvas**. Please see individual terminal documentation for allowed values of the size parameters. By default, the plot will fill this canvas.

set size <XX>, <YY> scales the plot itself relative to the size of the canvas. Scale values less than 1 will cause the plot to not fill the entire canvas. Scale values larger than 1 will cause only a portion of the plot to fit on the canvas. Please be aware that setting scale values larger than 1 may cause problems on some terminal types.

ratio causes **gnuplot** to try to create a graph with an aspect ratio of <r> (the ratio of the y-axis length to the x-axis length) within the portion of the plot specified by <xscale> and <yscale>.

The meaning of a negative value for <r> is different. If <r>=-1, gnuplot tries to set the scales so that the unit has the same length on both the x and y axes (suitable for geographical data, for instance). If <r>=-2, the unit on y has twice the length of the unit on x, and so on.

The success of **gnuplot** in producing the requested aspect ratio depends on the terminal selected. The graph area will be the largest rectangle of aspect ratio <r> that will fit into the specified portion of the output (leaving adequate margins, of course).

square is a synonym for **ratio 1**.

Both **noratio** and **nosquare** return the graph to the default aspect ratio of the terminal, but do not return <xscale> or <yscale> to their default values (1.0).

ratio and **square** have no effect on 3D plots, but do affect 3D projections created using **set view map**. See also **set view equal (p. 135)**, which forces the x and y axes of a 3D onto the same scale.

Examples:

To set the size so that the plot fills the available canvas:

```
set size 1,1
```

To make the graph half size and square use:

```
set size square 0.5,0.5
```

To make the graph twice as high as wide use:

```
set size ratio 2
```

See also

airfoil demo.

75.61 Style

Default plotting styles are chosen with the **set style data** and **set style function** commands. See **plot with (p. 77)** for information about how to override the default plotting style for individual functions and data sets. See **plotting styles (p. 37)** for a complete list of styles.

Syntax:

```
set style function <style>
set style data <style>
show style function
show style data
```

Default styles for specific plotting elements may also be set.

Syntax:

```
set style arrow <n> <arrowstyle>
set style fill <fillstyle>
set style histogram <histogram style options>
set style line <n> <linestyle>
```

75.61.1 Set style arrow

Each terminal has a default set of arrow and point types, which can be seen by using the command **test**. **set style arrow** defines a set of arrow types and widths and point types and sizes so that you can refer to them later by an index instead of repeating all the information at each invocation.

Syntax:

```
set style arrow <index> default
set style arrow <index> {nohead | head | heads}
                        {size <length>,<angle>{,<backangle>}}
                        {filled | empty | nofilled}
                        {front | back}
                        { {linestyle | ls <line_style>}
                          | {linetype | lt <line_type>}
                            {linewidth | lw <line_width} }
unset style arrow
show style arrow
```

<index> is an integer that identifies the arrowstyle.

If **default** is given all arrow style parameters are set to their default values.

If the linestyle <index> already exists, only the given parameters are changed while all others are preserved. If not, all undefined values are set to the default values.

Specifying **nohead** produces arrows drawn without a head — a line segment. This gives you yet another way to draw a line segment on the plot. By default, arrows have one head. Specifying **heads** draws arrow heads on both ends of the line.

Head size can be controlled by **size <length>,<angle>** or **size <length>,<angle>,<backangle>**, where **<length>** defines length of each branch of the arrow head and **<angle>** the angle (in degrees) they make with the arrow. **<Length>** is in x-axis units; this can be changed by **first**, **second**, **graph**, **screen**, or **character** before the <length>; see **coordinates (p. 19)** for details. **<Backangle>** only takes effect when **filled** or **empty** is also used. Then, **<backangle>** is the angle (in degrees) the back branches make with the arrow (in the same direction as **<angle>**). The **fig** terminal has a restricted backangle function. It supports three different angles. There are two thresholds: Below 70 degrees, the arrow head gets an indented back angle. Above 110 degrees, the arrow head has an acute back angle. Between these thresholds, the back line is straight.

Specifying **filled** produces filled arrow heads (if heads are used). Filling is supported on filled-polygon capable terminals, see help of **pm3d (p. 116)** for their list, otherwise the arrow heads are closed but not filled. The same result (closed but not filled arrow head) is reached by specifying **empty**. Further, filling and outline is obviously not supported on terminals drawing arrows by their own specific routines, like **metafont**, **metapost**, **latex** or **tgif**.

The line style may be selected from a user-defined list of line styles (see **set style line (p. 129)**) or may be defined here by providing values for **<line_type>** (an index from the default list of styles) and/or **<line_width>** (which is a multiplier for the default width).

Note, however, that if a user-defined line style has been selected, its properties (type and width) cannot be altered merely by issuing another **set style arrow** command with the appropriate index and **lt** or **lw**.

If **front** is given, the arrows are written on top of the graphed data. If **back** is given (the default), the arrow is written underneath the graphed data. Using **front** will prevent a arrow from being obscured by dense data.

Examples:

To draw an arrow without an arrow head and double width, use:

```
set style arrow 1 nohead lw 2
set arrow arrowstyle 1
```

See also **set arrow (p. 83)** for further examples.

75.61.2 Set style data

The **set style data** command changes the default plotting style for data plots.

Syntax:

```
set style data <plotting-style>
show style data
```

See **plotting styles (p. 37)** for the choices. If no choice is given, the choices are listed. **show style data** shows the current default data plotting style.

75.61.3 Set style fill

The **set style fill** command is used to set the default style of the plot elements in plots with boxes, histograms, candlesticks and filledcurves. This default can be superseded by fillstyles attached to individual plots. See also 'set style rectangle'.

Syntax:

```
set style fill {empty
                | {transparent} solid {<density>}
                | {transparent} pattern {<n>}}
               {border {<colorspec>} | noborder}
```

The default fillstyle is **empty**.

The **solid** option causes filling with a solid color, if the terminal supports that. The <density> parameter specifies the intensity of the fill color. At a <density> of 0.0, the box is empty, at <density> of 1.0, the inner area is of the same color as the current linetype. Some terminal types can vary the density continuously; others implement only a few levels of partial fill. If no <density> parameter is given, it defaults to 1.

The **pattern** option causes filling to be done with a fill pattern supplied by the terminal driver. The kind and number of available fill patterns depend on the terminal driver. If multiple datasets using filled boxes are plotted, the pattern cycles through all available pattern types, starting from pattern <n>, much as the line type cycles for multiple line plots.

The **empty** option causes filled boxes not to be filled. This is the default.

By default, **border**, the box is bounded by a solid line of the current linetype. **border <colorspec>** allows you to change the color of the border. **noborder** specifies that no bounding lines are drawn.

75.61.3.1 Set style fill transparent

Some terminals support the attribute **transparent** for filled areas. In the case of transparent solid fill areas, the **density** parameter is interpreted as an alpha value; that is, density 0 is fully transparent, density 1 is fully opaque. In the case of transparent pattern fill, the background of the pattern is either fully transparent or fully opaque.

Terminal	solid	pattern	pm3d
gif	no	yes	no
jpeg	yes	no	yes
pdf	yes	yes	yes
png	TrueColor	index	yes
post	no	yes	no
svg	yes	no	yes
wxt	yes	yes	yes
x11	no	yes	no

Note that there may be additional limitations on the creation or viewing of graphs containing transparent fill areas. For example, the png terminal can only use transparent fill if the "truecolor" option is set. Some pdf viewers may not correctly display the fill areas even if they are correctly described in the pdf file. Ghostscript/gv does not correctly display pattern-fill areas even though actual PostScript printers generally have no problem.

75.61.4 Set style function

The **set style function** command changes the default plotting style for function plots.

Syntax:

```
set style function <plotting-style>
show style function
```

See **plotting styles (p. 37)** for the choices. If no choice is given, the choices are listed. **show style function** shows the current default function plotting style.

75.61.5 Set style increment

Syntax:

```
set style increment {default|userstyles}
show style increment
```

By default, successive plots within the same graph will use successive linetypes from the default set for the current terminal type. However, choosing **set style increment user** allows you to step through the user-defined line styles rather than through the default linetypes.

Example:

```
set style line 1 lw 2 lc rgb "gold"
set style line 2 lw 2 lc rgb "purple"
set style line 4 lw 1 lc rgb "sea-green"
set style increment user

plot f1(x), f2(x), f3(x), f4(x)
```

should plot functions f1, f2, f4 in your 3 newly defined line styles. If a user-defined line style is not found then the corresponding default linetype is used instead. E.g. in the example above, f3(x) will be plotted using the default linetype 3.

75.61.6 Set style line

Each terminal has a default set of line and point types, which can be seen by using the command **test**. **set style line** defines a set of line types and widths and point types and sizes so that you can refer to them later by an index instead of repeating all the information at each invocation.

Syntax:

```
set style line <index> default
set style line <index> {{linetype  | lt} <line_type> | <colorspec>}
                       {{linecolor | lc} <colorspec>}
                       {{linewidth | lw} <line_width>}
                       {{pointtype | pt} <point_type>}
                       {{pointsize | ps} <point_size>}
                       {{pointinterval | pi} <interval>}
                       {palette}
unset style line
show style line
```

If **default** is given all line style parameters are set to their default values.

If the linestyle <index> already exists, only the given parameters are changed while all others are preserved. If not, all undefined values are set to the default values.

The line and point types default to the index value. The exact symbol that is drawn for that index value may vary from one terminal type to another.

The line width and point size are multipliers for the current terminal's default width and size (but note that <point_size> here is unaffected by the multiplier given by the command**set pointsize**).

The **pointinterval** controls the spacing between points in a plot drawn with style **linespoints**. The default is 0 (every point is drawn). For example, **set style line N pi 3** defines a linestyle that uses pointtype N, pointsize and linewidth equal to the current defaults for the terminal, and will draw every 3rd point in plots using **with linespoints**. A negative value for the interval is treated the same as a positive value, except that some terminals will try to interrupt the line where it passes through the point symbol.

Linestyles created by this mechanism do not replace the default linetype styles; both may be used. If you want plots to use the defined styles in preference to the default linetypes, please see **set style increment (p. 128)**.

Not all terminals support the **linewidth** and **pointsize** features; if not supported, the option will be ignored.

Terminal-independent colors may be assigned using either **linecolor <colorspec>** or **linetype <colorspec>**, abbreviated **lc** or **lt**. This requires giving a RGB color triple, a known palette color name, a fractional index into the current palette, or a constant value from the current mapping of the palette onto cbrange. See **colors (p. 29)**, **colorspec (p. 29)**, **set palette (p. 119)**, **colornames (p. 91)**, **cbrange (p. 144)**.

set style line <n> linetype <lt> will set both a terminal-dependent dot/dash pattern and color. The commands**set style line <n> linecolor <colorspec>** or **set style line <n> linetype <colorspec>** will set a new line color while leaving the existing dot-dash pattern unchanged.

In 3d mode (**splot** command), the special keyword **palette** is allowed as a shorthand for "linetype palette z". The color value corresponds to the z-value (elevation) of the splot, and varies smoothly along a line or surface.

Examples: Suppose that the default lines for indices 1, 2, and 3 are red, green, and blue, respectively, and the default point shapes for the same indices are a square, a cross, and a triangle, respectively. Then

```
set style line 1 lt 2 lw 2 pt 3 ps 0.5
```

defines a new linestyle that is green and twice the default width and a new pointstyle that is a half-sized triangle. The commands

```
set style function lines
plot f(x) lt 3, g(x) ls 1
```

will create a plot of f(x) using the default blue line and a plot of g(x) using the user-defined wide green line. Similarly the commands

```
set style function linespoints
plot p(x) lt 1 pt 3, q(x) ls 1
```

will create a plot of p(x) using the default triangles connected by a red line and q(x) using small triangles connected by a green line.

```
splot sin(sqrt(x*x+y*y))/sqrt(x*x+y*y) w l pal
```

creates a surface plot using smooth colors according to **palette**. Note, that this works only on some terminals. See also **set palette (p. 119)**, **set pm3d (p. 116)**.

```
set style line 10 linetype 1 linecolor rgb "cyan"
```

will assign linestyle 10 to be a solid cyan line on any terminal that supports rgb colors.

75.61.7 Set style rectangle

Rectangles defined with the **set object** command can have individual styles. However, if the object is not assigned a private style then it inherits a default that is taken from the **set style rectangle** command.

Syntax:

```
set style rectangle {front|back} {lw|linewidth <lw>}
                    {fillcolor <colorspec>} {fs <fillstyle>}
```

See **colorspec (p. 29)** and **fillstyle (p. 127)**. **fillcolor** may be abbreviated as **fc**.

Examples:

```
set style rectangle back fc rgb "white" fs solid 1.0 border lt -1
set style rectangle fc linsestyle 3 fs pattern 2 noborder
```

The default values correspond to solid fill with the background color and a black border.

75.62 Surface

The command **set surface** controls the display of surfaces by **splot**.

Syntax:

```
set surface
unset surface
show surface
```

The surface is drawn with the style specified by **with**, or else the appropriate style, data or function.

unset surface will cause **splot** to not draw points or lines corresponding to any of the function or data file points. If you want to turn off the surface for an individual function or data file while leaving the others active, use the **nosurface** keyword in the **splot** command. Contours may still be drawn on the surface, depending on the **set contour** option. The combination **unset surface; set contour base** is useful for displaying contours on the grid base. See also **set contour (p. 91)**.

75.63 Table

When **table** mode is enabled, **plot** and **splot** commands print out a multicolumn ASCII table of X Y {Z} R values rather than creating an actual plot on the current terminal. The character R takes on one of three values: "i" if the point is in the active range, "o" if it is out-of-range, or "u" if it is undefined. The data format is determined by the format of the axis labels (see **set format (p. 97)**), and the columns are separated by single spaces. This can be useful if you want to generate contours and then save them for further use, perhaps for plotting with **plot**; see **set contour (p. 91)** for example. The same method can be used to save interpolated data (see **set samples (p. 125)** and **set dgrid3d (p. 95)**).

Syntax:

```
set table {"outfile"}
plot <whatever>
unset table
```

Tabular output is written to the named file, if any, otherwise it is written to the current value of **set output**. You must explicitly **unset table** in order to go back to normal plotting on the current terminal.

75.64 Terminal

gnuplot supports many different graphics devices. Use **set terminal** to tell **gnuplot** what kind of output to generate. Use **set output** to redirect that output to a file or device.

Syntax:

```
set terminal {<terminal-type> | push | pop}
show terminal
```

If <terminal-type> is omitted, **gnuplot** will list the available terminal types. <terminal-type> may be abbreviated.

If both **set terminal** and **set output** are used together, it is safest to give **set terminal** first, because some terminals set a flag which is needed in some operating systems.

Several terminals have many additional options. For example, see **png (p. 177)**, or **postscript (p. 178)**. The options used by a previous invocation **set term <term> <options>** of a given **<term>** are remembered, thus subsequent **set term <term>** does not reset them. This helps in printing, for instance, when switching among different terminals — previous options don't have to be repeated.

The command **set term push** remembers the current terminal including its settings while **set term pop** restores it. This is equivalent to **save term** and **load term**, but without accessing the filesystem. Therefore they can be used to achieve platform independent restoring of the terminal after printing, for instance. After gnuplot's startup, the default terminal or that from **startup** file is pushed automatically. Therefore portable scripts can rely that **set term pop** restores the default terminal on a given platform unless another terminal has been pushed explicitly.

For more information, see the **complete list of terminals (p. 150)**.

75.65 Termoption

The **set termoption** command allows you to change the behaviour of the current terminal without requiring a new **set terminal** command. Only one option can be changed per command, and only a small number of options can be changed this way. Currently the only options accepted are

```
set termoption {no}enhanced
set termoption font "<fontname>{,<fontsize>}"
set termoption {solid|dashed}
set termoption {linewidth <lw>}{lw <lw>}
```

75.66 Tics

Control of the major (labelled) tics on all axes at once is possible with the **set tics** command.

Fine control of the major (labelled) tics on all axes at once is possible with the **set tics** command. The tics may be turned off with the **unset tics** command, and may be turned on (the default state) with **set tics**. Similar commands (by preceding 'tics' by the axis name) control the major tics on a single axis.

Syntax:

```
set tics {axis | border} {{no}mirror}
         {in | out} {scale {default | <major> {,<minor>}}}
         {{no}rotate {by <ang>}} {offset <offset> | nooffset}
         { format "formatstring" } { font "name{,<size>}" }
         { textcolor <colorspec> }
set tics {front | back}
unset tics
show tics
```

The options in the first set above can be applied individually to any or all axes, i.e., x, y, z, x2, y2, and cb.

Set tics **front** or **back** applies to all axes at once, but only for 2D plots (not splot). It controls whether the tics are placed behind or in front of the plot elements, in the case that there is overlap.

axis or **border** tells **gnuplot** to put the tics (both the tics themselves and the accompanying labels) along the axis or the border, respectively. If the axis is very close to the border, the **axis** option will move the tic labels

to outside the border in case the border is printed (see **set border** **(p. 86)**). The relevant margin settings will usually be sized badly by the automatic layout algorithm in this case.

mirror tells **gnuplot** to put unlabelled tics at the same positions on the opposite border. **nomirror** does what you think it does.

in and **out** change the tic marks to be drawn inwards or outwards.

With **scale**, the size of the tic marks can be adjusted. If <minor> is not specified, it is 0.5*<major>. The default size 1.0 for major tics and 0.5 for minor tics is requested by **scale default**.

rotate asks **gnuplot** to rotate the text through 90 degrees, which will be done if the terminal driver in use supports text rotation. **norotate** cancels this. **rotate by** <ang> asks for rotation by <ang> degrees, supported by some terminal types.

The defaults are **border mirror norotate** for tics on the x and y axes, and **border nomirror norotate** for tics on the x2 and y2 axes. For the z axis, the default is **nomirror**.

The <offset> is specified by either x,y or x,y,z, and may be preceded by **first**, **second**, **graph**, **screen**, or **character** to select the coordinate system. <offset> is the offset of the tics texts from their default positions, while the default coordinate system is **character**. See **coordinates** **(p. 19)** for details. **nooffset** switches off the offset.

set tics with no options restores to place tics inwards. Every other options are retained.

See also **set xtics** **(p. 139)** for more control of major (labelled) tic marks and **set mxtics** for control of minor tic marks. These commands provide control at a axis by axis basis.

75.67 Ticslevel

Deprecated. See **set xyplane** **(p. 141)**.

75.68 Ticscale

The **set ticscale** command is deprecated, use **set tics scale** instead.

75.69 Timestamp

The command **set timestamp** places the time and date of the plot in the left margin.

Syntax:

```
set timestamp {"<format>"} {top|bottom} {{no}rotate}
              {offset <xoff>{,<yoff>}} {font "<fontspec>"}
unset timestamp
show timestamp
```

The format string allows you to choose the format used to write the date and time. Its default value is what asctime() uses: "%a %b %d %H:%M:%S %Y" (weekday, month name, day of the month, hours, minutes, seconds, four-digit year). With **top** or **bottom** you can place the timestamp at the top or bottom of the left margin (default: bottom). **rotate** lets you write the timestamp vertically, if your terminal supports vertical text. The constants <xoff> and <yoff> are offsets that let you adjust the position more finely. <font> is used to specify the font with which the time is to be written.

The abbreviation **time** may be used in place of **timestamp**.

Example:

```
set timestamp "%d/%m/%y %H:%M" offset 80,-2 font "Helvetica"
```

See **set timefmt** **(p. 132)** for more information about time format strings.

75.70 Timefmt

This command applies to timeseries where data are composed of dates/times. It has no meaning unless the command **set xdata time** is given also.

Syntax:

```
set timefmt "<format string>"
show timefmt
```

The string argument tells **gnuplot** how to read timedata from the datafile. The valid formats are:

Time Series timedata Format Specifiers	
Format	Explanation
%d	day of the month, 1–31
%m	month of the year, 1–12
%y	year, 0–99
%Y	year, 4-digit
%j	day of the year, 1–365
%H	hour, 0–24
%M	minute, 0–60
%s	seconds since the Unix epoch (1970-01-01 00:00 UTC)
%S	second, 0–60
%b	three-character abbreviation of the name of the month
%B	name of the month

Any character is allowed in the string, but must match exactly. \t (tab) is recognized. Backslash-octals (\nnn) are converted to char. If there is no separating character between the time/date elements, then %d, %m, %y, %H, %M and %S read two digits each, %Y reads four digits and %j reads three digits. %b requires three characters, and %B requires as many as it needs.

Spaces are treated slightly differently. A space in the string stands for zero or more whitespace characters in the file. That is, "%H %M" can be used to read "1220" and "12 20" as well as "12 20".

Each set of non-blank characters in the timedata counts as one column in the **using n:n** specification. Thus **11:11 25/12/76 21.0** consists of three columns. To avoid confusion, **gnuplot** requires that you provide a complete **using** specification if your file contains timedata.

Since **gnuplot** cannot read non-numerical text, if the date format includes the day or month in words, the format string must exclude this text. But it can still be printed with the "%a", "%A", "%b", or "%B" specifier: see **set format (p. 97)** for more details about these and other options for printing timedata. (**gnuplot** will determine the proper month and weekday from the numerical values.)

See also **set xdata (p. 136)** and **Time/date (p. 36)** for more information.

Example:

```
set timefmt "%d/%m/%Y\t%H:%M"
```

tells **gnuplot** to read date and time separated by tab. (But look closely at your data — what began as a tab may have been converted to spaces somewhere along the line; the format string must match what is actually in the file.) See also

```
time data demo.
```

75.71 Title

The **set title** command produces a plot title that is centered at the top of the plot. **set title** is a special case of **set label**.

Syntax:

```
set title {"<title-text>"} {offset <offset>} {font "<font>{,<size>}"}
          {{textcolor | tc} {<colorspec> | default}} {{no}enhanced}
show title
```

If <offset> is specified by either x,y or x,y,z the title is moved by the given offset. It may be preceded by **first**, **second**, **graph**, **screen**, or **character** to select the coordinate system. See **coordinates (p. 19)** for details. By default, the **character** coordinate system is used. For example, "**set title offset 0,-1**" will change only the y offset of the title, moving the title down by roughly the height of one character. The size of a character depends on both the font and the terminal.

<font> is used to specify the font with which the title is to be written; the units of the font <size> depend upon which terminal is used.

textcolor <**colorspec**> changes the color of the text. <colorspec> can be a linetype, an rgb color, or a palette mapping. See help for **colorspec (p. 29)** and **palette (p. 119)**.

noenhanced requests that the title not be processed by the enhanced text mode parser, even if enhanced text mode is currently active.

set title with no parameters clears the title.

See **syntax (p. 35)** for details about the processing of backslash sequences and the distinction between single- and double-quotes.

75.72 Tmargin

The command **set tmargin** sets the size of the top margin. Please see **set margin (p. 109)** for details.

75.73 Trange

The **set trange** command sets the parametric range used to compute x and y values when in parametric or polar modes. Please see **set xrange (p. 138)** for details.

75.74 Urange

The **set urange** and **set vrange** commands set the parametric ranges used to compute x, y, and z values when in **splot** parametric mode. Please see **set xrange (p. 138)** for details.

75.75 Variables

The **show variables** command lists the current value of user-defined and internal variables. Gnuplot internally defines variables whose names begin with GPVAL_, MOUSE_, FIT_, and TERM_.

Syntax:

```
show variables      # show variables that do not begin with GPVAL_
show variables all  # show all variables including those beginning GPVAL_
show variables NAME # show only variables beginning with NAME
```

75.76 Version

The **show version** command lists the version of gnuplot being run, its last modification date, the copyright holders, and email addresses for the FAQ, the gnuplot-info mailing list, and reporting bugs–in short, the information listed on the screen when the program is invoked interactively.

Syntax:

```
show version {long}
```

When the **long** option is given, it also lists the operating system, the compilation options used when **gnuplot** was installed, the location of the help file, and (again) the useful email addresses.

75.77 View

The **set view** command sets the viewing angle for **splot**s. It controls how the 3D coordinates of the plot are mapped into the 2D screen space. It provides controls for both rotation and scaling of the plotted data, but supports orthographic projections only. It supports both 3D projection or orthogonal 2D projection into a 2D plot-like map.

Syntax:

```
set view <rot_x>{,{<rot_z>}{,{<scale>}{,<scale_z>}}}
set view map
set view {no}equal {xy|xyz}
show view
```

where <rot_x> and <rot_z> control the rotation angles (in degrees) in a virtual 3D coordinate system aligned with the screen such that initially (that is, before the rotations are performed) the screen horizontal axis is x, screen vertical axis is y, and the axis perpendicular to the screen is z. The first rotation applied is <rot_x> around the x axis. The second rotation applied is <rot_z> around the new z axis.

Command **set view map** is used to represent the drawing as a map. It can be used for **contour** plots, or for color **pm3d** maps. In the latter, take care that you properly use **zrange** and **cbrange** for input data point filtering and color range scaling, respectively.

<rot_x> is bounded to the [0:180] range with a default of 60 degrees, while <rot_z> is bounded to the [0:360] range with a default of 30 degrees. <scale> controls the scaling of the entire **splot**, while <scale_z> scales the z axis only. Both scales default to 1.0.

Examples:

```
set view 60, 30, 1, 1
set view ,,0.5
```

The first sets all the four default values. The second changes only scale, to 0.5.

75.77.1 Equal_axes

The command **set view equal xy** forces the unit length of the x and y axes to be on the same scale, and chooses that scale so that the plot will fit on the page. The command **set view equal xyz** additionally sets the z axis scale to match the x and y axes; however there is no guarantee that the current z axis range will fit within the plot boundary. By default all three axes are scaled independently to fill the available area.

See also **set xyplane (p. 141)**.

75.78 Vrange

The **set urange** and **set vrange** commands set the parametric ranges used to compute x, y, and z values when in **splot** parametric mode. Please see **set xrange (p. 138)** for details.

75.79 X2data

The **set x2data** command sets data on the x2 (top) axis to timeseries (dates/times). Please see **set xdata (p. 136)**.

75.80 X2dtics

The **set x2dtics** command changes tics on the x2 (top) axis to days of the week. Please see **set xdtics (p. 136)** for details.

75.81 X2label

The **set x2label** command sets the label for the x2 (top) axis. Please see **set xlabel (p. 136)**.

75.82 X2mtics

The **set x2mtics** command changes tics on the x2 (top) axis to months of the year. Please see **set xmtics (p. 137)** for details.

75.83 X2range

The **set x2range** command sets the horizontal range that will be displayed on the x2 (top) axis. Please see **set xrange (p. 138)** for details.

75.84 X2tics

The **set x2tics** command controls major (labelled) tics on the x2 (top) axis. Please see **set xtics (p. 139)** for details.

75.85 X2zeroaxis

The **set x2zeroaxis** command draws a line at the origin of the x2 (top) axis (y2 = 0). For details, please see **set zeroaxis (p. 143)**.

75.86 Xdata

This command sets the datatype on the x axis to time/date. A similar command does the same thing for each of the other axes.

Syntax:

```
set xdata {time}
show xdata
```

The same syntax applies to **ydata**, **zdata**, **x2data**, **y2data** and **cbdata**.

The **time** option signals that the datatype is indeed time/date. If the option is not specified, the datatype reverts to normal.

See **set timefmt (p. 132)** to tell gnuplot how to read date or time data. The time/date is converted to seconds from start of the century. There is currently only one timefmt, which implies that all the time/date columns must conform to this format. Specification of ranges should be supplied as quoted strings according to this format to avoid interpretation of the time/date as an expression.

The function 'strftime' (type "man strftime" on unix to look it up) is used to print tic-mark labels. **gnuplot** tries to figure out a reasonable format for this unless the **set format x "string"** has supplied something that does not look like a decimal format (more than one '%' or neither %f nor %g).

See also **Time/date (p. 36)** for more information.

75.87 Xdtics

The **set xdtics** commands converts the x-axis tic marks to days of the week where 0=Sun and 6=Sat. Overflows are converted modulo 7 to dates. **set noxdtics** returns the labels to their default values. Similar commands do the same things for the other axes.

Syntax:

```
set xdtics
unset xdtics
show xdtics
```

The same syntax applies to **ydtics**, **zdtics**, **x2dtics**, **y2dtics** and **cbdtics**.

See also the **set format (p. 97)** command.

75.88 Xlabel

The **set xlabel** command sets the x axis label. Similar commands set labels on the other axes.

Syntax:

```
set xlabel {"<label>"} {offset <offset>} {font "<font>{,<size>}"}
           {{textcolor | tc} {lt <line_type> | default}} {{no}enhanced}
           {rotate by <degrees>}
show xlabel
```

The same syntax applies to **x2label**, **ylabel**, **y2label**, **zlabel** and **cblabel**.

If <offset> is specified by either x,y or x,y,z the label is moved by the given offset. It may be preceded by **first**, **second**, **graph**, **screen**, or **character** to select the coordinate system. See **coordinates (p. 19)** for details.

By default, the **character** coordinate system is used. For example, "**set xlabel offset -1,0**" will change only the x offset of the title, moving the label roughly one character width to the left. The size of a character depends on both the font and the terminal.

<font> is used to specify the font in which the label is written; the units of the font <size> depend upon which terminal is used.

textcolor lt <n> sets the text color to that of line type <n>.

noenhanced requests that the label text not be processed by the enhanced text mode parser, even if enhanced text mode is currently active.

To clear a label, put no options on the command line, e.g., "**set y2label**".

The default positions of the axis labels are as follows:

xlabel: The x-axis label is centered below the bottom axis.

ylabel: The position of the y-axis label depends on the terminal, and can be one of the following three positions:

1. Horizontal text flushed left at the top left of the plot. Terminals that cannot rotate text will probably use this method. If **set x2tics** is also in use, the ylabel may overwrite the left-most x2tic label. This may be remedied by adjusting the ylabel position or the left margin.

2. Vertical text centered vertically at the left of the plot. Terminals that can rotate text will probably use this method.

3. Horizontal text centered vertically at the left of the plot. The EEPIC, LaTeX and TPIC drivers use this method. The EEPIC driver will produce a stack of characters so as not to overwrite the plot. With other drivers (such as LaTeX and TPIC), the user probably has to insert line breaks using \\ to prevent the ylabel from overwriting the plot.

zlabel: The z-axis label is centered along the z axis and placed in the space above the grid level.

cblabel: The color box axis label is centered along the box and placed below or right according to horizontal or vertical color box gradient.

y2label: The y2-axis label is placed to the right of the y2 axis. The position is terminal-dependent in the same manner as is the y-axis label.

x2label: The x2-axis label is placed above the top axis but below the plot title. It is also possible to create an x2-axis label by using new-line characters to make a multi-line plot title, e.g.,

```
set title "This is the title\n\nThis is the x2label"
```

Note that double quotes must be used. The same font will be used for both lines, of course.

The orientation (rotation angle) of the x, x2, y and y2 axis labels can be explicitly changed from the default setting, but this applies only to 2D plots and only on terminals that support text rotation.

If you are not satisfied with the default position of an axis label, use **set label** instead–that command gives you much more control over where text is placed.

Please see **syntax (p. 35)** for further information about backslash processing and the difference between single- and double-quoted strings.

75.89 Xmtics

The **set xmtics** command converts the x-axis tic marks to months of the year where 1=Jan and 12=Dec. Overflows are converted modulo 12 to months. The tics are returned to their default labels by **unset xmtics**. Similar commands perform the same duties for the other axes.

Syntax:

```
set xmtics
unset xmtics
show xmtics
```

The same syntax applies to **x2mtics**, **ymtics**, **y2mtics**, **zmtics** and **cbmtics**.

See also the **set format (p. 97)** command.

75.90 Xrange

The **set xrange** command sets the horizontal range that will be displayed. A similar command exists for each of the other axes, as well as for the polar radius r and the parametric variables t, u, and v.

Syntax:

```
set xrange { [{{<min>}:{<max>}}] {{no}reverse} {{no}writeback} }
           | restore
show xrange
```

where <min> and <max> terms are constants, expressions or an asterisk to set autoscaling. If the data are time/date, you must give the range as a quoted string according to the **set timefmt** format. Any value omitted will not be changed.

The same syntax applies to **yrange**, **zrange**, **x2range**, **y2range**, **cbrange**, **rrange**, **trange**, **urange** and **vrange**.

The **reverse** option reverses the direction of the axis, e.g., **set xrange [0:1] reverse** will produce an axis with 1 on the left and 0 on the right. This is identical to the axis produced by **set xrange [1:0]**, of course. **reverse** is intended primarily for use with **autoscale**.

The **writeback** option essentially saves the range found by **autoscale** in the buffers that would be filled by **set xrange**. This is useful if you wish to plot several functions together but have the range determined by only some of them. The **writeback** operation is performed during the **plot** execution, so it must be specified before that command. To restore, the last saved horizontal range use **set xrange restore**. For example,

```
set xrange [-10:10]
set yrange [] writeback
plot sin(x)
set yrange restore
replot x/2
```

results in a yrange of [-1:1] as found only from the range of sin(x); the [-5:5] range of x/2 is ignored. Executing **show yrange** after each command in the above example should help you understand what is going on.

In 2-d, **xrange** and **yrange** determine the extent of the axes, **trange** determines the range of the parametric variable in parametric mode or the range of the angle in polar mode. Similarly in parametric 3-d, **xrange**, **yrange**, and **zrange** govern the axes and **urange** and **vrange** govern the parametric variables.

In polar mode, **rrange** determines the radial range plotted. <rmin> acts as an additive constant to the radius, whereas <rmax> acts as a clip to the radius — no point with radius greater than <rmax> will be plotted. **xrange** and **yrange** are affected — the ranges can be set as if the graph was of r(t)-rmin, with rmin added to all the labels.

Any range may be partially or totally autoscaled, although it may not make sense to autoscale a parametric variable unless it is plotted with data.

Ranges may also be specified on the **plot** command line. A range given on the plot line will be used for that single **plot** command; a range given by a **set** command will be used for all subsequent plots that do not specify their own ranges. The same holds true for **splot**.

Examples:

To set the xrange to the default:

```
set xrange [-10:10]
```

To set the yrange to increase downwards:

```
set yrange [10:-10]
```

To change zmax to 10 without affecting zmin (which may still be autoscaled):

```
set zrange [:10]
```

To autoscale xmin while leaving xmax unchanged:

```
set xrange [*:]
```

75.91 Xtics

Fine control of the major (labelled) tics on the x axis is possible with the **set xtics** command. The tics may be turned off with the **unset xtics** command, and may be turned on (the default state) with **set xtics**. Similar commands control the major tics on the y, z, x2 and y2 axes.

Syntax:

```
set xtics {axis | border} {{no}mirror}
          {in | out} {scale {default | <major> {,<minor>}}}
          {{no}rotate {by <ang>}} {offset <offset> | nooffset}
          {add}
          {  autofreq
           | <incr>
           | <start>, <incr> {,<end>}
           | ({"<label>"} <pos> {<level>} {,{"<label>"}...) }
          { format "formatstring" } { font "name{,<size>}" }
          { rangelimited }
          { textcolor <colorspec> }
unset xtics
show xtics
```

The same syntax applies to **ytics**, **ztics**, **x2tics**, **y2tics** and **cbtics**.

axis or **border** tells **gnuplot** to put the tics (both the tics themselves and the accompanying labels) along the axis or the border, respectively. If the axis is very close to the border, the **axis** option will move the tic labels to outside the border. The relevant margin settings will usually be sized badly by the automatic layout algorithm in this case.

mirror tells **gnuplot** to put unlabelled tics at the same positions on the opposite border. **nomirror** does what you think it does.

in and **out** change the tic marks to be drawn inwards or outwards.

With **scale**, the size of the tic marks can be adjusted. If <minor> is not specified, it is 0.5*<major>. The default size 1.0 for major tics and 0.5 for minor tics is requested by **scale default**.

rotate asks **gnuplot** to rotate the text through 90 degrees, which will be done if the terminal driver in use supports text rotation. **norotate** cancels this. **rotate by** <**ang**> asks for rotation by <ang> degrees, supported by some terminal types.

The defaults are **border mirror norotate** for tics on the x and y axes, and **border nomirror norotate** for tics on the x2 and y2 axes. For the z axis, the **{axis | border}** option is not available and the default is **nomirror**. If you do want to mirror the z-axis tics, you might want to create a bit more room for them with **set border**.

The <offset> is specified by either x,y or x,y,z, and may be preceded by **first**, **second**, **graph**, **screen**, or **character** to select the coordinate system. <offset> is the offset of the tics texts from their default positions, while the default coordinate system is **character**. See **coordinates (p. 19)** for details. **nooffset** switches off the offset.

Example:

Move xtics more closely to the plot.

```
set xtics offset 0,graph 0.05
```

set xtics with no options restores the default border or axis if xtics are being displayed; otherwise it has no effect. Any previously specified tic frequency or position {and labels} are retained.

Positions of the tics are calculated automatically by default or if the **autofreq** option is given; otherwise they may be specified in either of two forms:

The implicit <start>, <incr>, <end> form specifies that a series of tics will be plotted on the axis between the values <start> and <end> with an increment of <incr>. If <end> is not given, it is assumed to be infinity. The increment may be negative. If neither <start> nor <end> is given, <start> is assumed to be negative infinity, <end> is assumed to be positive infinity, and the tics will be drawn at integral multiples of <incr>. If the axis is logarithmic, the increment will be used as a multiplicative factor.

If you specify to a negative <start> or <incr> after a numerical value (e.g., **rotate by** <**angle**> or **offset** <**offset**>), the parser fails because it subtracts <start> or <incr> from that value. As a workaround, specify **0-**<**start**> resp. **0-**<**incr**> in that case.

Example:

```
set xtics border offset 0,0.5 -5,1,5
```

Fails with 'invalid expression' at the last comma.

```
set xtics border offset 0,0.5 0-5,1,5
```

or

```
set xtics offset 0,0.5 border -5,1,5
```

Sets tics at the border, tics text with an offset of 0,0.5 characters, and sets the start, increment, and end to -5, 1, and 5, as requested.

The **set grid** options 'front', 'back' and 'layerdefault' affect the drawing order of the xtics, too.

Examples:

Make tics at 0, 0.5, 1, 1.5, ..., 9.5, 10.

```
set xtics 0,.5,10
```

Make tics at ..., -10, -5, 0, 5, 10, ...

```
set xtics 5
```

Make tics at 1, 100, 1e4, 1e6, 1e8.

```
set logscale x; set xtics 1,100,1e8
```

The explicit ("<label>" <pos> <level>, ...) form allows arbitrary tic positions or non-numeric tic labels. In this form, the tics do not need to be listed in numerical order. Each tic has a position, optionally with a label. Note that the label is a string enclosed by quotes. It may be a constant string, such as "hello", may contain formatting information for converting the position into its label, such as "%3f clients", or may be empty, "". See **set format (p. 97)** for more information. If no string is given, the default label (numerical) is used.

An explicit tic mark has a third parameter, the "level". The default is level 0, a major tic. A level of 1 generates a minor tic. If the level is specified, then the label must also be supplied.

Examples:

```
set xtics ("low" 0, "medium" 50, "high" 100)
set xtics (1,2,4,8,16,32,64,128,256,512,1024)
set ytics ("bottom" 0, "" 10, "top" 20)
set ytics ("bottom" 0, "" 10 1, "top" 20)
```

In the second example, all tics are labelled. In the third, only the end tics are labelled. In the fourth, the unlabeled tic is a minor tic.

Normally if explicit tics are given, they are used instead of auto-generated tics. Conversely if you specify **set xtics auto** or the like it will erase any previously specified explicit tics. You can mix explicit and auto-generated tics by using the keyword **add**, which must appear before the tic style being added.

Example:

```
set xtics 0,.5,10
set xtics add ("Pi" 3.14159)
```

This will automatically generate tic marks every 0.5 along x, but will also add an explicit labeled tic mark at pi.

However they are specified, tics will only be plotted when in range.

Format (or omission) of the tic labels is controlled by **set format**, unless the explicit text of a label is included in the **set xtics ("<label>")** form.

Minor (unlabelled) tics can be added automatically by the **set mxtics** command, or at explicit positions by the **set xtics ("" <pos> 1, ...)** form.

75.91.1 Xtics time_data

In case of timeseries data, axis tic position values must be given as quoted dates or times according to the format **timefmt**. If the <start>, <incr>, <end> form is used, <start> and <end> must be given according to **timefmt**, but <incr> must be in seconds. Times will be written out according to the format given on **set format**, however.

Examples:

```
set xdata time
set timefmt "%d/%m"
set xtics format "%b %d"
set xrange ["01/12":"06/12"]
set xtics "01/12", 172800, "05/12"
```

```
set xdata time
set timefmt "%d/%m"
set xtics format "%b %d"
set xrange ["01/12":"06/12"]
set xtics ("01/12", "" "03/12", "05/12")
```

Both of these will produce tics "Dec 1", "Dec 3", and "Dec 5", but in the second example the tic at "Dec 3" will be unlabelled.

75.91.2 Xtics rangelimited

This option limits both the auto-generated axis tic labels and the corresponding plot border to the range of values actually present in the data that has been plotted. Note that this is independent of the current range limits for the plot. For example, suppose that the data in "file.dat" all lies in the range $2 < y < 4$. Then the following commands will create a plot for which the left-hand plot border (y axis) is drawn for only this portion of the total y range, and only the axis tics in this region are generated. I.e., the plot will be scaled to the full range on y, but there will be a gap between 0 and 2 on the left border and another gap between 4 and 10. This style is sometimes refered to as a **range-frame** graph.

```
set border 3
set yrange [0:10]
set ytics nomirror rangelimited
plot "file.dat"
```

75.92 Xyplane

The **set xyplane** command adjusts the position at which the xy plane is drawn in a 3D plot. The synonym "set ticslevel" is accepted for backwards compatibility.

Syntax:

```
set xyplane at <zvalue>
set xyplane relative <frac>
set ticslevel <frac>        # equivalent to set xyplane relative
show xyplane
```

The form **set xyplane relative <frac>** places the xy plane below the range in Z, where the distance from the xy plane to Zmin is given as a fraction of the total range in z. The default value is 0.5. Negative values are permitted, but tic labels on the three axes may overlap. The older, deprecated, form **set ticslevel** is retained for backwards compatibility.

To place the xy-plane at a position 'pos' on the z-axis, **ticslevel** may be set equal to (pos - zmin) / (zmin - zmax). However, this position will change if the z range is changed.

The alternative form **set xyplane at <zvalue>** fixes the placement of the xy plane at a specific Z value regardless of the current z range. Thus to force the x, y, and z axes to meet at a common origin one would specify **set xyplane at 0**.

See also **set view (p. 134)**, and **set zeroaxis (p. 143)**.

75.93 Xzeroaxis

The **set xzeroaxis** command draws a line at $y = 0$. For details, please see **set zeroaxis (p. 143)**.

75.94 Y2data

The **set y2data** command sets y2 (right-hand) axis data to timeseries (dates/times). Please see **set xdata (p. 136)**.

75.95 Y2dtics

The **set y2dtics** command changes tics on the y2 (right-hand) axis to days of the week. Please see **set xdtics (p. 136)** for details.

75.96 Y2label

The **set y2label** command sets the label for the y2 (right-hand) axis. Please see **set xlabel (p. 136)**.

75.97 Y2mtics

The **set y2mtics** command changes tics on the y2 (right-hand) axis to months of the year. Please see **set xmtics (p. 137)** for details.

75.98 Y2range

The **set y2range** command sets the vertical range that will be displayed on the y2 (right-hand) axis. Please see **set xrange (p. 138)** for details.

75.99 Y2tics

The **set y2tics** command controls major (labelled) tics on the y2 (right-hand) axis. Please see **set xtics (p. 139)** for details.

75.100 Y2zeroaxis

The **set y2zeroaxis** command draws a line at the origin of the y2 (right-hand) axis (x2 = 0). For details, please see **set zeroaxis (p. 143)**.

75.101 Ydata

The **set ydata** commands sets y-axis data to timeseries (dates/times). Please see **set xdata (p. 136)**.

75.102 Ydtics

The **set ydtics** command changes tics on the y axis to days of the week. Please see **set xdtics (p. 136)** for details.

75.103 Ylabel

This command sets the label for the y axis. Please see **set xlabel (p. 136)**.

75.104 Ymtics

The **set ymtics** command changes tics on the y axis to months of the year. Please see **set xmtics (p. 137)** for details.

75.105 Yrange

The **set yrange** command sets the vertical range that will be displayed on the y axis. Please see **set xrange (p. 138)** for details.

75.106 Ytics

The **set ytics** command controls major (labelled) tics on the y axis. Please see **set xtics (p. 139)** for details.

75.107 Yzeroaxis

The **set yzeroaxis** command draws a line at x = 0. For details, please see **set zeroaxis (p. 143)**.

75.108 Zdata

The **set zdata** command sets zaxis data to timeseries (dates/times). Please see **set xdata (p. 136)**.

75.109 Zdtics

The **set zdtics** command changes tics on the z axis to days of the week. Please see **set xdtics (p. 136)** for details.

75.110 Zzeroaxis

The **set zzeroaxis** command draws a line through (x=0,y=0). This has no effect on 2D plots, including splot with **set view map**. For details, please see **set zeroaxis (p. 143)** and **set xyplane (p. 141)**.

75.111 Cbdata

Set color box axis data to timeseries (dates/times). Please see **set xdata (p. 136)**.

75.112 Cbdtics

The **set cbdtics** command changes tics on the color box axis to days of the week. Please see **set xdtics (p. 136)** for details.

75.113 Zero

The **zero** value is the default threshold for values approaching 0.0.

Syntax:

```
set zero <expression>
show zero
```

gnuplot will not plot a point if its imaginary part is greater in magnitude than the **zero** threshold. This threshold is also used in various other parts of **gnuplot** as a (crude) numerical-error threshold. The default **zero** value is 1e-8. **zero** values larger than 1e-3 (the reciprocal of the number of pixels in a typical bitmap display) should probably be avoided, but it is not unreasonable to set **zero** to 0.0.

75.114 Zeroaxis

The x axis may be drawn by **set xzeroaxis** and removed by **unset xzeroaxis**. Similar commands behave similarly for the y, x2, y2, and z axes.

Syntax:

```
set {x|x2|y|y2|z}zeroaxis { {linestyle | ls <line_style>}
                           | { linetype | lt <line_type>}
                             { linewidth | lw <line_width>}}
unset {x|x2|y|y2|z}zeroaxis
show {x|y|z}zeroaxis
```

By default, these options are off. The selected zero axis is drawn with a line of type <line_type> and width <line_width> (if supported by the terminal driver currently in use), or a user-defined style <line_style>.

If no linetype is specified, any zero axes selected will be drawn using the axis linetype (linetype 0).

set zeroaxis is equivalent to **set xzeroaxis; set yzeroaxis**. Note that the z-axis must be set separately using **set zzeroaxis**.

Examples:

To simply have the y=0 axis drawn visibly:

```
set xzeroaxis
```

If you want a thick line in a different color or pattern, instead:

```
set xzeroaxis linetype 3 linewidth 2.5
```

75.115 Zlabel

This command sets the label for the z axis. Please see **set xlabel (p. 136)**.

75.116 Zmtics

The **set zmtics** command changes tics on the z axis to months of the year. Please see **set xmtics (p. 137)** for details.

75.117 Zrange

The **set zrange** command sets the range that will be displayed on the z axis. The zrange is used only by **splot** and is ignored by **plot**. Please see **set xrange (p. 138)** for details.

75.118 Ztics

The **set ztics** command controls major (labelled) tics on the z axis. Please see **set xtics (p. 139)** for details.

75.119 Cblabel

This command sets the label for the color box axis. Please see **set xlabel (p. 136)**.

75.120 Cbmtics

The **set cbmtics** command changes tics on the color box axis to months of the year. Please see **set xmtics (p. 137)** for details.

75.121 Cbrange

The **set cbrange** command sets the range of values which are colored using the current **palette** by styles **with pm3d**, **with image** and **with palette**. Values outside of the color range use color of the nearest extreme.

If the cb-axis is autoscaled in **splot**, then the colorbox range is taken from **zrange**. Points drawn in **splot ... pm3d|palette** can be filtered by using different **zrange** and **cbrange**.

Please see **set xrange (p. 138)** for details on **set cbrange (p. 144)** syntax. See also **set palette (p. 119)** and **set colorbox (p. 90)**.

75.122 Cbtics

The **set cbtics** command controls major (labelled) tics on the color box axis. Please see **set xtics (p. 139)** for details.

76 Shell

The **shell** command spawns an interactive shell. To return to **gnuplot**, type **logout** if using VMS, **exit** or the END-OF-FILE character if using Unix, **endcli** if using AmigaOS, or **exit** if using MS-DOS or OS/2.

There are two ways of spawning a shell command: using **system** command or via **!** ($ if using VMS). The former command takes a string as a parameter and thus it can be used anywhere among other gnuplot commands, while the latter syntax requires to be the only command on the line. Control will return immediately to **gnuplot** after this command is executed. For example, in AmigaOS, MS-DOS or OS/2,

```
! dir
```

or

```
system "dir"
```

prints a directory listing and then returns to **gnuplot**.

Other examples of the former syntax:

```
 system "date"; set time; plot "a.dat"
 print=1; if (print) replot; set out; system "lpr x.ps"
```

77 Splot

splot is the command for drawing 3D plots (well, actually projections on a 2D surface, but you knew that). It can create a plot from functions or data read from files in a manner very similar to the **plot** command. **splot** provides only a single x, y, and z axis; there is no equivalent to the x2 and y2 secondary axes provided by **plot**.

See **plot (p. 61)** for features common to the **plot (p. 61)** command; only differences are discussed in detail here.

Syntax:

```
splot {<ranges>}
      {<iteration>}
      <function> | "<datafile>" {datafile-modifiers}}
      {<title-spec>} {with <style>}
      {, {definitions{,}} <function> ...}
```

where either a <function> or the name of a data file enclosed in quotes is supplied. The function can be a mathematical expression, or a triple of mathematical expressions in parametric mode.

By default **splot** draws the xy plane completely below the plotted data. The offset between the lowest ztic and the xy plane can be changed by **set xyplane**. The orientation of a **splot** projection is controlled by **set view**. See **set view (p. 134)** and **set xyplane (p. 141)** for more information.

The syntax for setting ranges on the **splot** command is the same as for **plot**. In non-parametric mode, the order in which ranges must be given is **xrange**, **yrange**, and **zrange**. In parametric mode, the order is **urange**, **vrange**, **xrange**, **yrange**, and **zrange**.

The **title** option is the same as in **plot**. The operation of **with** is also the same as in **plot**, except that the plotting styles available to **splot** are limited to **lines**, **points**, **linespoints**, **dots**, and **impulses**; the error-bar capabilities of **plot** are not available for **splot**.

The **datafile** options have more differences.

See also **show plot (p. 116)**.

77.1 Data-file

As for **plot**, discrete data contained in a file can be displayed by specifying the name of the data file, enclosed in quotes, on the **splot** command line.

Syntax:

```
splot '<file_name>' {binary <binary list>}
                    {matrix}
                    {index <index list>}
                    {every <every list>}
                    {using <using list>}
```

The special filenames "" and "-" are permitted, as in **plot**.

In brief, **binary** and **matrix** indicate that the data are in a special form, **index** selects which data sets in a multi-data-set file are to be plotted, **every** specifies which datalines (subsets) within a single data set are to be plotted, and **using** determines how the columns within a single record are to be interpreted.

The options **index** and **every** behave the same way as with **plot**; **using** does so also, except that the **using** list must provide three entries instead of two.

The **plot** options **thru** and **smooth** are not available for **splot**, but **cntrparam** and **dgrid3d** provide limited smoothing capabilities.

Data file organization is essentially the same as for **plot**, except that each point is an (x,y,z) triple. If only a single value is provided, it will be used for z, the datablock number will be used for y, and the index of the data point in the datablock will be used for x. If two or four values are provided, **gnuplot** uses the last value for calculating the color in pm3d plots. Three values are interpreted as an (x,y,z) triple. Additional values are generally used as errors, which can be used by **fit**.

Single blank records separate datablocks in a **splot** datafile; **splot** treats datablocks as the equivalent of function y-isolines. No line will join points separated by a blank record. If all datablocks contain the same number of points, **gnuplot** will draw cross-isolines between datablocks, connecting corresponding points. This is termed "grid data", and is required for drawing a surface, for contouring (**set contour**) and hidden-line removal (**set hidden3d**). See also **splot grid_data (p. 148)**.

It is no longer necessary to specify **parametric** mode for three-column **splot**s.

77.1.1 Binary matrix

Gnuplot can read matrix binary files by use of the option **binary** appearing without keyword qualifications unique to general binary, i.e., **array**, **record**, **format**, or **filetype**. Other general binary keywords for translation should also apply to matrix binary. (See **binary general (p. 64)** for more details.)

In previous versions, **gnuplot** dynamically detected binary data files. It is now necessary to specify the keyword **binary** directly after the filename.

Single precision floats are stored in a binary file as follows:

```
<N+1>  <y0>   <y1>   <y2>  ...  <yN>
 <x0> <z0,0> <z0,1> <z0,2> ... <z0,N>
 <x1> <z1,0> <z1,1> <z1,2> ... <z1,N>
  :      :      :      :   ...    :
```

which are converted into triplets:

```
<x0> <y0> <z0,0>
<x0> <y1> <z0,1>
<x0> <y2> <z0,2>
 :    :     :
<x0> <yN> <z0,N>

<x1> <y0> <z1,0>
<x1> <y1> <z1,1>
 :    :     :
```

These triplets are then converted into **gnuplot** iso-curves and then **gnuplot** proceeds in the usual manner to do the rest of the plotting.

A collection of matrix and vector manipulation routines (in C) is provided in **binary.c**. The routine to write binary data is

```
int fwrite_matrix(file,m,nrl,nrl,ncl,nch,row_title,column_title)
```

An example of using these routines is provided in the file **bf_test.c**, which generates binary files for the demo file **demo/binary.dem**.

The **index** keyword is not supported, since the file format allows only one surface per file. The **every** and **using** filters are supported. **using** operates as if the data were read in the above triplet form.

See also **binary general (p. 64)** and

    Binary File Splot Demo.

77.1.2 Example datafile

A simple example of plotting a 3D data file is

```
splot 'datafile.dat'
```

where the file "datafile.dat" might contain:

```
# The valley of the Gnu.
   0 0 10
   0 1 10
   0 2 10


   1 0 10
   1 1 5
   1 2 10


   2 0 10
   2 1 1
   2 2 10


   3 0 10
   3 1 0
   3 2 10
```

Note that "datafile.dat" defines a 4 by 3 grid (4 rows of 3 points each). Rows (datablocks) are separated by blank records.

Note also that the x value is held constant within each dataline. If you instead keep y constant, and plot with hidden-line removal enabled, you will find that the surface is drawn 'inside-out'.

Actually for grid data it is not necessary to keep the x values constant within a datablock, nor is it necessary to keep the same sequence of y values. **gnuplot** requires only that the number of points be the same for each datablock. However since the surface mesh, from which contours are derived, connects sequentially corresponding points, the effect of an irregular grid on a surface plot is unpredictable and should be examined on a case-by-case basis.

77.1.3 Matrix_ascii

The **matrix** keyword (without a sequent **binary** keyword) in

```
{s}plot 'a.dat' matrix
```

indicates that data are stored in an ascii numbers matrix format.

The z-values are read in a row at a time, i. e.,

```
z11 z12 z13 z14 ...
z21 z22 z23 z24 ...
z31 z32 z33 z34 ...
```

and so forth.

In 3D, the x- and y-indices of the matrix surface plot correspond to column and row indices of the matrix, respectively, being enumerated from 0. You can rescale or transform the axes as usual for a data file with three columns by means of x=$1, y=$2, z=$3. For example

```
splot 'a.dat' matrix using (1+$1/100):(1+$2*10):3
```

A blank line or comment line ends the matrix, and starts a new surface mesh. You can select among the meshes inside a file by the **index** option to the **splot** command, as usual.

See **matrix (p. 148)** for examples of plotting rows and columns of the matrix in a 2D plot.

77.1.4 Matrix

Datafile can be in an ascii or binary matrix format. The **matrix** flag indicates that the file is ascii, the **binary** or **matrix binary** stands for a binary format. For details, see **matrix ascii (p. 147)** and **matrix binary (p. 146)**.

Basic usage in **splot**:

```
splot 'a.dat' matrix
splot 'a.gpbin' {matrix} binary
```

Advanced usage in **splot**:

```
splot 'a.dat' matrix using 1:2:3
splot 'a.gpbin' {matrix} binary using 1:2:3
```

allows to transform the axes coordinates and the z-data independently.

Usage in **plot**:

```
plot `a.dat` matrix
plot `a.dat` matrix using 1:3
plot 'a.gpbin' {matrix} binary using 1:3
```

will plot rows of the matrix, while using 2:3 will plot matrix columns, and using 1:2 the point coordinates (rather useless). Applying the **every** option you can specify explicit rows and columns.

Example – rescale axes of a matrix in an ascii file:

```
splot `a.dat` matrix using (1+$1):(1+$2*10):3
```

Example – plot the 3rd row of a matrix in an ascii file:

```
plot 'a.dat' matrix using 1:3 every 1:999:1:2
```

(rows are enumerated from 0, thus 2 instead of 3).

77.2 Grid data

The 3D routines are designed for points in a grid format, with one sample, datapoint, at each mesh intersection; the datapoints may originate from either evaluating a function, see **set isosamples (p. 102)**, or reading a datafile, see **splot datafile (p. 145)**. The term "isoline" is applied to the mesh lines for both functions and data. Note that the mesh need not be rectangular in x and y, as it may be parameterized in u and v, see **set isosamples (p. 102)**.

However, **gnuplot** does not require that format. In the case of functions, 'samples' need not be equal to 'isosamples', i.e., not every x-isoline sample need intersect a y-isoline. In the case of data files, if there are an equal number of scattered data points in each datablock, then "isolines" will connect the points in a datablock, and "cross-isolines" will connect the corresponding points in each datablock to generate a "surface". In either case, contour and hidden3d modes may give different plots than if the points were in the intended format. Scattered data can be converted to a {different} grid format with **set dgrid3d**.

The contour code tests for z intensity along a line between a point on a y-isoline and the corresponding point in the next y-isoline. Thus a **splot** contour of a surface with samples on the x-isolines that do not coincide with a y-isoline intersection will ignore such samples. Try:

```
set xrange [-pi/2:pi/2]; set yrange [-pi/2:pi/2]
set style function lp
set contour
set isosamples 10,10; set samples 10,10;
splot cos(x)*cos(y)
set samples 4,10; replot
set samples 10,4; replot
```

77.3 Splot overview

splot can display a surface as a collection of points, or by connecting those points. As with **plot**, the points may be read from a data file or result from evaluation of a function at specified intervals, see **set isosamples**

(p. 102). The surface may be approximated by connecting the points with straight line segments, see **set surface (p. 130)**, in which case the surface can be made opaque with **set hidden3d.** The orientation from which the 3d surface is viewed can be changed with **set view**.

Additionally, for points in a grid format, **splot** can interpolate points having a common amplitude (see **set contour (p. 91)**) and can then connect those new points to display contour lines, either directly with straight-line segments or smoothed lines (see **set cntrparam (p. 89)**). Functions are already evaluated in a grid format, determined by **set isosamples** and **set samples**, while file data must either be in a grid format, as described in **data-file**, or be used to generate a grid (see **set dgrid3d (p. 95)**).

Contour lines may be displayed either on the surface or projected onto the base. The base projections of the contour lines may be written to a file, and then read with **plot**, to take advantage of **plot**'s additional formatting capabilities.

78 System

system "command" executes "command" using the standard shell. See **shell (p. 144)**. If called as a function, **system("command")** returns the resulting character stream from stdout as a string. One optional trailing newline is ignored.

This can be used to import external functions into gnuplot scripts:

```
f(x) = real(system(sprintf("somecommand %f", x)))
```

79 Test

This command graphically tests or presents terminal and palette capabilities.

Syntax:

```
test {terminal | palette [rgb|rbg|grb|gbr|brg|bgr]}
```

test or **test terminal** creates a display of line and point styles and other useful things appropriate for and supported by the **terminal** you are just using.

test palette draws graphically profiles R(z),G(z),B(z), where 0<=z<=1, as calculated by the current color **palette**. In other words, it is a beautiful plot you would have to construct from the result of **show palette palette 256 float**. The optional parameter, a permutation of letters rgb, determines the sequence of r,g,b profiles drawn one after the other — try this yourself for **set palette gray**. The default sequence is rgb.

80 Undefine

Clear one or more previously defined user variables. This is useful in order to reset the state of a script containing an initialization test.

Example:

```
undefine foo foo1 foo2
if (!exists("foo")) load "initialize.gp"
```

81 Unset

Options set using the **set** command may be returned to their default state by the corresponding **unset** command. The **unset** command may contain an optional iteration clause. See **iteration (p. 59)**.

Examples:

```
set xtics mirror rotate by -45 0,10,100
...
unset xtics

# Unset labels numbered between 100 and 200
unset for [i=100:200] label i
```

82 Update

This command writes the current values of the fit parameters into the given file, formatted as an initial-value file (as described in the **fit**section). This is useful for saving the current values for later use or for restarting a converged or stopped fit.

Syntax:

```
update <filename> {<filename>}
```

If a second filename is supplied, the updated values are written to this file, and the original parameter file is left unmodified.

Otherwise, if the file already exists, **gnuplot** first renames it by appending **.old** and then opens a new file. That is, "**update 'fred'**" behaves the same as "**!rename fred fred.old; update 'fred.old' 'fred'**". [On DOS and other systems that use the twelve-character "filename.ext" naming convention, "ext" will be "**old**" and "filename" will be related (hopefully recognizably) to the initial name. Renaming is not done at all on VMS systems, since they use file-versioning.]

Please see **fit (p. 52)** for more information.

Part IV

Terminal types

83 Complete list of terminals

Gnuplot supports a large number of output formats. These are selected by choosing an appropriate terminal type, possibly with additional modifying options. See **set terminal (p. 131)**.

This document may describe terminal types that are not available to you because they were not configured or installed on your system. To see a list of terminals available on a particular gnuplot installation, type 'set terminal' with no modifiers.

83.1 Aed767

The **aed512** and **aed767** terminal drivers support AED graphics terminals. The two drivers differ only in their horizontal ranges, which are 512 and 768 pixels, respectively. Their vertical range is 575 pixels. There are no options for these drivers.

83.2 Aifm

NOTE: this terminal driver is outdated. Since Adobe Illustrator understands PostScript level 1 directly, you should use **set terminal post level1** instead.

Several options may be set in **aifm** — the Adobe Illustrator 3.0+ driver.

Syntax:

```
set terminal aifm {<color>} {"<fontname>"} {<fontsize>}
```

<color> is either **color** or **monochrome**; "<fontname>" is the name of a valid PostScript font; <fontsize> is the size of the font in PostScript points, before scaling by the **set size** command. Selecting **default** sets all options to their default values: **monochrome**, "Times-Roman", and 14pt.

Since AI does not really support multiple pages, multiple graphs will be drawn directly on top of one another. However, each graph will be grouped individually, making it easy to separate them inside AI (just pick them up and move them).

Examples:

```
set term aifm
set term aifm 22
set size 0.7,1.4; set term aifm color "Times-Roman" 14
```

83.3 Amiga

The **amiga** terminal, for Commodore Amiga computers, allows the user to plot either to a screen (default), or, if Kickstart 3.0 or higher is installed, to a window on the current public screen. The font and its size can also be selected.

Syntax:

```
set terminal amiga {screen | window} {"<fontname>"} {<fontsize>}
```

The default font is 8-point "topaz".

The screen option uses a virtual screen, so it is possible that the graph will be larger than the screen.

83.4 Apollo

The **apollo** terminal driver supports the Apollo Graphics Primitive Resource with rescaling after window resizing. It has no options.

If a fixed-size window is desired, the **gpr** terminal may be used instead.

83.5 Aqua

This terminal relies on AquaTerm.app for display on Mac OS X.

Syntax:

```
set terminal aqua {<n>} {title "<wintitle>"} {size <x> <y>}
                  {font "<fontname>{,<fontsize>}"}
                  {{no}enhanced} {solid|dashed} {dl <dashlength>}}
```

where <n> is the number of the window to draw in (default is 0), <wintitle> is the name shown in the title bar (default "Figure <n>"), <x> <y> is the size of the plot (default is 846x594 pt = 11.75x8.25 in).

Use <fontname> to specify the font to use (default is "Times-Roman"), and <fontsize> to specify the font size (default is 14.0 pt). The old syntax {fname "<fontname>"} {fsize <fontsize>} is still supported.

The aqua terminal supports enhanced text mode (see **enhanced (p. 20)**), except for overprint. Font support is limited to the fonts available on the system. Character encoding can be selected by **set encoding** and currently supports iso_latin_1, iso_latin_2, cp1250, and default which equals UTF8.

Lines can be drawn either solid or dashed, (default is solid) and the dash spacing can be modified by <dashlength> which is a multiplier > 0.

83.6 Be

gnuplot provides the **be** terminal type for use with X servers. This terminal type is set automatically at startup if the **DISPLAY** environment variable is set, if the **TERM** environment variable is set to **xterm**, or if the **-display** command line option is used.

Syntax:

```
set terminal be {reset} {<n>}
```

Multiple plot windows are supported: **set terminal be <n>** directs the output to plot window number n. If n>0, the terminal number will be appended to the window title and the icon will be labeled **gplt <n>**. The active window may distinguished by a change in cursor (from default to crosshair.)

Plot windows remain open even when the **gnuplot** driver is changed to a different device. A plot window can be closed by pressing the letter q while that window has input focus, or by choosing **close** from a window manager menu. All plot windows can be closed by specifying **reset**, which actually terminates the subprocess which maintains the windows (unless **-persist** was specified).

Plot windows will automatically be closed at the end of the session unless the **-persist** option was given.

The size or aspect ratio of a plot may be changed by resizing the **gnuplot** window.

Linewidths and pointsizes may be changed from within **gnuplot** with **set linestyle**.

For terminal type **be**, **gnuplot** accepts (when initialized) the standard X Toolkit options and resources such as geometry, font, and name from the command line arguments or a configuration file. See the X(1) man page (or its equivalent) for a description of such options.

A number of other **gnuplot** options are available for the **be** terminal. These may be specified either as command-line options when **gnuplot** is invoked or as resources in the configuration file ".Xdefaults". They are set upon initialization and cannot be altered during a **gnuplot** session.

83.6.1 Command-line_options

In addition to the X Toolkit options, the following options may be specified on the command line when starting **gnuplot** or as resources in your ".Xdefaults" file:

'-mono'	forces monochrome rendering on color displays.
'-gray'	requests grayscale rendering on grayscale or color displays. (Grayscale displays receive monochrome rendering by default.)
'-clear'	requests that the window be cleared momentarily before a new plot is displayed.
'-raise'	raises plot window after each plot.
'-noraise'	does not raise plot window after each plot.
'-persist'	plots windows survive after main gnuplot program exits.

The options are shown above in their command-line syntax. When entered as resources in ".Xdefaults", they require a different syntax.

Example:

```
gnuplot*gray: on
```

gnuplot also provides a command line option (**-pointsize** <**v**>) and a resource, **gnuplot*pointsize:** <**v**>, to control the size of points plotted with the **points** plotting style. The value **v** is a real number (greater than 0 and less than or equal to ten) used as a scaling factor for point sizes. For example, **-pointsize 2** uses points twice the default size, and **-pointsize 0.5** uses points half the normal size.

83.6.2 Monochrome_options

For monochrome displays, **gnuplot** does not honor foreground or background colors. The default is black-on-white. **-rv** or **gnuplot*reverseVideo: on** requests white-on-black.

83.6.3 Color_resources

For color displays, **gnuplot** honors the following resources (shown here with their default values) or the greyscale resources. The values may be color names as listed in the BE rgb.txt file on your system, hexadecimal RGB color specifications (see BE documentation), or a color name followed by a comma and an **intensity** value from 0 to 1. For example, **blue, 0.5** means a half intensity blue.

gnuplot*background: white
gnuplot*textColor: black
gnuplot*borderColor: black
gnuplot*axisColor: black
gnuplot*line1Color: red
gnuplot*line2Color: green
gnuplot*line3Color: blue
gnuplot*line4Color: magenta
gnuplot*line5Color: cyan
gnuplot*line6Color: sienna
gnuplot*line7Color: orange
gnuplot*line8Color: coral

The command-line syntax for these is, for example,

Example:

```
gnuplot -background coral
```

83.6.4 Grayscale_resources

When **-gray** is selected, **gnuplot** honors the following resources for grayscale or color displays (shown here with their default values). Note that the default background is black.

gnuplot*background: black
gnuplot*textGray: white
gnuplot*borderGray: gray50
gnuplot*axisGray: gray50
gnuplot*line1Gray: gray100
gnuplot*line2Gray: gray60
gnuplot*line3Gray: gray80
gnuplot*line4Gray: gray40
gnuplot*line5Gray: gray90
gnuplot*line6Gray: gray50
gnuplot*line7Gray: gray70
gnuplot*line8Gray: gray30

83.6.5 Line_resources

gnuplot honors the following resources for setting the width (in pixels) of plot lines (shown here with their default values.) 0 or 1 means a minimal width line of 1 pixel width. A value of 2 or 3 may improve the appearance of some plots.

gnuplot*borderWidth: 2
gnuplot*axisWidth: 0
gnuplot*line1Width: 0
gnuplot*line2Width: 0
gnuplot*line3Width: 0
gnuplot*line4Width: 0
gnuplot*line5Width: 0
gnuplot*line6Width: 0
gnuplot*line7Width: 0
gnuplot*line8Width: 0

gnuplot honors the following resources for setting the dash style used for plotting lines. 0 means a solid line. A two-digit number **jk** (**j** and **k** are >= 1 and <= 9) means a dashed line with a repeated pattern of **j** pixels on followed by **k** pixels off. For example, '16' is a "dotted" line with one pixel on followed by six pixels off. More elaborate on/off patterns can be specified with a four-digit value. For example, '4441' is four on, four off, four on, one off. The default values shown below are for monochrome displays or monochrome rendering on color or grayscale displays. For color displays, the default for each is 0 (solid line) except for **axisDashes** which defaults to a '16' dotted line.

gnuplot*borderDashes: 0
gnuplot*axisDashes: 16
gnuplot*line1Dashes: 0
gnuplot*line2Dashes: 42
gnuplot*line3Dashes: 13
gnuplot*line4Dashes: 44
gnuplot*line5Dashes: 15
gnuplot*line6Dashes: 4441
gnuplot*line7Dashes: 42
gnuplot*line8Dashes: 13

83.7 Canvas

Syntax:

```
set terminal canvas {size <xsize>, <ysize>} {fsize <fontsize>}
                    {{no}enhanced} {linewidth <lw>}
```

```
                             {standalone {mousing} | name '<funcname>'}
                             {jsdir 'URL/for/javascripts'}
                             {title '<some string>'}
```

where <xsize> and <ysize> set the size of the plot area in pixels. The default size in standalone mode is 600 by 400 pixels. The default font size is 10. NB: Only one font is available, the ascii portion of Hershey simplex Roman provided in the file canvastext.js. You can replace this with the file canvasmath.js, which contains also UTF-8 encoded Hershey simplex Greek and math symbols.

The default **standalone** mode creates an html page containing javascript code that renders the plot using the HTML 5 canvas element. The html page links to two required javascript files 'canvastext.js' and 'gnuplot_common.js'. By default these point to local files, on unix-like systems usually in directory /usr/local/share/gnuplot/<version>/js. See installation notes for other platforms. You can change this by using the **jsdir** option to specify either a different local directory or a general URL. The latter is usually appropriate if the plot is exported for viewing on remote client machines.

All plots produced by the canvas terminal are mouseable. The additional keyword **mousing** causes the **standalone** mode to add a mouse-tracking box underneath the plot. It also adds a link to a javascript file 'gnuplot_mouse.js' and to a stylesheet for the mouse box 'gnuplot_mouse.css' in the same local or URL directory as 'canvastext.js'.

The **name** option creates a file containing only javascript. Both the javascript function it contains and the id of the canvas element that it draws onto are taken from the following string parameter. The commands

```
set term canvas name 'fishplot'
set output 'fishplot.js'
```

will create a file containing a javascript function fishplot() that will draw onto a canvas with id=fishplot. An html page that invokes this javascript function must also load the canvastext.js function as described above. A minimal html file to wrap the fishplot created above might be:

```
<html>
<head>
    <script src="canvastext.js"></script>
    <script src="gnuplot_common.js"></script>
</head>
<body onload="fishplot();">
    <script src="fishplot.js"></script>
    <canvas id="fishplot" width=600 height=400>
        <div id="err_msg">No support for HTML 5 canvas element</div>
    </canvas>
</body>
</html>
```

83.8 Cgi

The **cgi** and **hcgi** terminal drivers support SCO CGI drivers. **hcgi** is for printers; the environment variable CGIPRNT must be set. **cgi** may be used for either a display or hardcopy; if the environment variable CGIDISP is set, then that display is used. Otherwise CGIPRNT is used.

These terminals have no options.

83.9 Cgm

The **cgm** terminal generates a Computer Graphics Metafile, Version 1. This file format is a subset of the ANSI X3.122-1986 standard entitled "Computer Graphics - Metafile for the Storage and Transfer of Picture Description Information".

Syntax:

```
set terminal cgm {color | monochrome} {solid | dashed} {{no}rotate}
                 {<mode>} {width <plot_width>} {linewidth <line_width>}
                 {font "<fontname>,<fontsize>"}
                 {<color0> <color1> <color2> ...}
```

solid draws all curves with solid lines, overriding any dashed patterns; <mode> is **landscape**, **portrait**, or **default**; <plot_width> is the assumed width of the plot in points; <line_width> is the line width in points (default 1); <fontname> is the name of a font (see list of fonts below) <fontsize> is the size of the font in points (default 12).

The first six options can be in any order. Selecting **default** sets all options to their default values.

Each color must be of the form 'xrrggbb', where x is the literal character 'x' and 'rrggbb' are the red, green and blue components in hex. For example, 'x00ff00' is green. The background color is set first, then the plotting colors.

Examples:

```
set terminal cgm landscape color rotate dashed width 432 \
               linewidth 1  'Helvetica Bold' 12       # defaults
set terminal cgm linewidth 2  14  # wider lines & larger font
set terminal cgm portrait "Times Italic" 12
set terminal cgm color solid      # no pesky dashes!
```

83.9.1 Cgm font

The first part of a Computer Graphics Metafile, the metafile description, includes a font table. In the picture body, a font is designated by an index into this table. By default, this terminal generates a table with the following 35 fonts, plus six more with **italic** replaced by **oblique**, or vice-versa (since at least the Microsoft Office and Corel Draw CGM import filters treat **italic** and **oblique** as equivalent):

CGM fonts
Helvetica
Helvetica Bold
Helvetica Oblique
Helvetica Bold Oblique
Times Roman
Times Bold
Times Italic
Times Bold Italic
Courier
Courier Bold
Courier Oblique
Courier Bold Oblique
Symbol
Hershey/Cartographic_Roman
Hershey/Cartographic_Greek
Hershey/Simplex_Roman
Hershey/Simplex_Greek
Hershey/Simplex_Script
Hershey/Complex_Roman
Hershey/Complex_Greek
Hershey/Complex_Script
Hershey/Complex_Italic
Hershey/Complex_Cyrillic
Hershey/Duplex_Roman
Hershey/Triplex_Roman
Hershey/Triplex_Italic
Hershey/Gothic_German
Hershey/Gothic_English
Hershey/Gothic_Italian
Hershey/Symbol_Set_1
Hershey/Symbol_Set_2
Hershey/Symbol_Math
ZapfDingbats
Script
15

The first thirteen of these fonts are required for WebCGM. The Microsoft Office CGM import filter implements the 13 standard fonts listed above, and also 'ZapfDingbats' and 'Script'. However, the script font may only be accessed under the name '15'. For more on Microsoft import filter font substitutions, check its help file which you may find here:

```
C:\Program Files\Microsoft Office\Office\Cgmimp32.hlp
```

and/or its configuration file, which you may find here:

```
C:\Program Files\Common Files\Microsoft Shared\Grphflt\Cgmimp32.cfg
```

In the **set term** command, you may specify a font name which does not appear in the default font table. In that case, a new font table is constructed with the specified font as its first entry. You must ensure that the spelling, capitalization, and spacing of the name are appropriate for the application that will read the CGM file. (Gnuplot and any MIL-D-28003A compliant application ignore case in font names.) If you need to add several new fonts, use several **set term** commands.

Example:

```
set terminal cgm 'Old English'
set terminal cgm 'Tengwar'
set terminal cgm 'Arabic'
set output 'myfile.cgm'
plot ...
set output
```

You cannot introduce a new font in a **set label** command.

83.9.2 Cgm fontsize

Fonts are scaled assuming the page is 6 inches wide. If the **size** command is used to change the aspect ratio of the page or the CGM file is converted to a different width, the resulting font sizes will be scaled up or down accordingly. To change the assumed width, use the **width** option.

83.9.3 Cgm linewidth

The **linewidth** option sets the width of lines in pt. The default width is 1 pt. Scaling is affected by the actual width of the page, as discussed under the **fontsize** and **width** options.

83.9.4 Cgm rotate

The **norotate** option may be used to disable text rotation. For example, the CGM input filter for Word for Windows 6.0c can accept rotated text, but the DRAW editor within Word cannot. If you edit a graph (for example, to label a curve), all rotated text is restored to horizontal. The Y axis label will then extend beyond the clip boundary. With **norotate**, the Y axis label starts in a less attractive location, but the page can be edited without damage. The **rotate** option confirms the default behavior.

83.9.5 Cgm solid

The **solid** option may be used to disable dashed line styles in the plots. This is useful when color is enabled and the dashing of the lines detracts from the appearance of the plot. The **dashed** option confirms the default behavior, which gives a different dash pattern to each line type.

83.9.6 Cgm size

Default size of a CGM plot is 32599 units wide and 23457 units high for landscape, or 23457 units wide by 32599 units high for portrait.

83.9.7 Cgm width

All distances in the CGM file are in abstract units. The application that reads the file determines the size of the final plot. By default, the width of the final plot is assumed to be 6 inches (15.24 cm). This distance is used

to calculate the correct font size, and may be changed with the **width** option. The keyword should be followed by the width in points. (Here, a point is 1/72 inch, as in PostScript. This unit is known as a "big point" in TeX.) Gnuplot **expressions** can be used to convert from other units.

Example:

```
set terminal cgm width 432            # default
set terminal cgm width 6*72           # same as above
set terminal cgm width 10/2.54*72     # 10 cm wide
```

83.9.8 Cgm nofontlist

The default font table includes the fonts recommended for WebCGM, which are compatible with the Computer Graphics Metafile input filter for Microsoft Office and Corel Draw. Another application might use different fonts and/or different font names, which may not be documented. The **nofontlist** (synonym **winword6**) option deletes the font table from the CGM file. In this case, the reading application should use a default table. Gnuplot will still use its own default font table to select font indices. Thus, 'Helvetica' will give you an index of 1, which should get you the first entry in your application's default font table. 'Helvetica Bold' will give you its second entry, etc.

83.10 Corel

The **corel** terminal driver supports CorelDraw.

Syntax:

```
set terminal corel {  default
                    | {monochrome | color
                         {"<font>" {<fontsize>
                            {<xsize> <ysize> {<linewidth> }}}}}
```

where the fontsize and linewidth are specified in points and the sizes in inches. The defaults are monochrome, "SwitzerlandLight", 22, 8.2, 10 and 1.2.

83.11 Debug

This terminal is provided to allow for the debugging of **gnuplot**. It is likely to be of use only for users who are modifying the source code.

83.12 Dospc

The **dospc** terminal driver supports PCs with arbitrary graphics boards, which will be automatically detected. It should be used only if you are not using the gcc or Zortec C/C++ compilers.

83.13 Dumb

The **dumb** terminal driver has an optional size specification and trailing linefeed control.

Syntax:

```
set terminal dumb {[no]feed} {<xsize> <ysize>}
                  {[no]enhanced}
```

where <xsize> and <ysize> set the size of the dumb terminals. Default is 79 by 24. The last newline is printed only if **feed** is enabled.

Examples:

```
set term dumb nofeed
set term dumb 79 49 # VGA screen---why would anyone do that?
```

83.14 Dxf

The **dxf** terminal driver creates pictures that can be imported into AutoCad (Release 10.x). It has no options of its own, but some features of its plots may be modified by other means. The default size is 120x80 AutoCad units, which can be changed by **set size**. **dxf** uses seven colors (white, red, yellow, green, cyan, blue and magenta), which can be changed only by modifying the source file. If a black-and-white plotting device is used, the colors are mapped to differing line thicknesses. See the description of the AutoCad print/plot command.

83.15 Dxy800a

This terminal driver supports the Roland DXY800A plotter. It has no options.

83.16 Eepic

The **eepic** terminal driver supports the extended LaTeX picture environment. It is an alternative to the **latex** driver.

The output of this terminal is intended for use with the "eepic.sty" macro package for LaTeX. To use it, you need "eepic.sty", "epic.sty" and a printer driver that supports the "tpic" \specials. If your printer driver doesn't support those \specials, "eepicemu.sty" will enable you to use some of them. dvips and dvipdfm do support the "tpic" \specials.

Syntax:

```
set terminal eepic {color, dashed, rotate, small, tiny, default, <fontsize>}
```

Options: You can give options in any order you wish. 'color' causes gnuplot to produce \color{...} commands so that the graphs are colored. Using this option, you must include \usepackage{color} in the preambel of your latex document. 'dashed' will allow dashed line types; without this option, only solid lines with varying thickness will be used. 'dashed' and 'color' are mutually exclusive; if 'color' is specified, then 'dashed' will be ignored. 'rotate' will enable true rotated text (by 90 degrees). Otherwise, rotated text will be typeset with letters stacked above each other. If you use this option you must include \usepackage{graphicx} in the preamble. 'small' will use \scriptsize symbols as point markers (Probably does not work with TeX, only LaTeX2e). Default is to use the default math size. 'tiny' uses \scriptscriptstyle symbols. 'default' resets all options to their defaults = no color, no dashed lines, pseudo-rotated (stacked) text, large point symbols. <fontsize> is a number which specifies the font size inside the picture environment; the unit is pt (points), i.e., 10 pt equals approx. 3.5 mm. If fontsize is not specified, then all text inside the picture will be set in \footnotesize.

Notes: Remember to escape the # character (or other chars meaningful to (La-)TeX) by \\ (2 backslashes). It seems that dashed lines become solid lines when the vertices of a plot are too close. (I do not know if that is a general problem with the tpic specials, or if it is caused by a bug in eepic.sty or dvips/dvipdfm.) The default size of an eepic plot is 5x3 inches, which can be scaled by 'set size a,b'. Points, among other things, are drawn using the LaTeX commands "\Diamond", "\Box", etc. These commands no longer belong to the LaTeX2e core; they are included in the latexsym package, which is part of the base distribution and thus part of any LaTeX implementation. Please do not forget to use this package. Instead of latexsym, you can also include the amssymb package. All drivers for LaTeX offer a special way of controlling text positioning: If any text string begins with '{', you also need to include a '}' at the end of the text, and the whole text will be centered both horizontally and vertically. If the text string begins with '[', you need to follow this with a position specification (up to two out of t,b,l,r), ']{', the text itself, and finally '}'. The text itself may be anything LaTeX can typeset as an LR-box. '\rule{}{}'s may help for best positioning.

Examples: set term eepic

```
output graphs as eepic macros inside a picture environment;
\input the resulting file in your LaTeX document.
```

set term eepic color tiny rotate 8

```
eepic macros with \color macros, \scripscriptsize point markers,
true rotated text, and all text set with 8pt.
```

About label positioning: Use gnuplot defaults (mostly sensible, but sometimes not really best):

```
set title '\LaTeX\ -- $ \gamma $'
```

Force centering both horizontally and vertically:

```
set label '{\LaTeX\ -- $ \gamma $}' at 0,0
```

Specify own positioning (top here):

```
set xlabel '[t]{\LaTeX\ -- $ \gamma $}'
```

The other label – account for long ticlabels:

```
set ylabel '[r]{\LaTeX\ -- $ \gamma $\rule{7mm}{0pt}}'
```

83.17 Emf

The **emf** terminal generates an Enhanced Metafile Format file. This file format is recognized by many Windows applications.

Syntax:

```
set terminal emf {color | monochrome} {solid | dashed}
                 {enhanced {noproportional}}
                 {linewidth <LW>} {dashlength <DL>} {size XX,YY}
                 {"<fontname>"} {<fontsize>}    #old syntax
                 {font "<fontname>,<fontsize>"} #new syntax
```

In **monochrome** mode successive line types cycle through dash patterns. In **color** mode successive line types use successive colors, and only after all 8 default colors are exhausted is the dash pattern incremented. **solid** draws all curves with solid lines, overriding any dashed patterns; **linewidth <factor>** multiplies all line widths by this factor. **dashlength <factor>** is useful for thick lines. <fontname> is the name of a font; and **<fontsize>** is the size of the font in points.

The nominal size of the output image defaults to 1024x768 in arbitrary units. You may specify a different nominal size using the **size** option.

Enhanced text mode tries to approximate proportional character spacing. If you are using a monospaced font, or don't like the approximation, you can turn off this correction using the **noproportional** option.

The default settings are **color dashed font "Arial,12" size 1024,768** Selecting **default** sets all options to their default values.

Examples:

```
set terminal emf 'Times Roman Italic' 12
set terminal emf color solid    # no pesky dashes!
```

83.18 Emxvga

The **emxvga**, **emxvesa** and **vgal** terminal drivers support PCs with SVGA, vesa SVGA and VGA graphics boards, respectively. They are intended to be compiled with "emx-gcc" under either DOS or OS/2. They also need VESA and SVGAKIT maintained by Johannes Martin (JMARTIN@GOOFY.ZDV.UNI-MAINZ.DE) with additions by David J. Liu (liu@phri.nyu.edu).

Syntax:

```
set terminal emxvga
set terminal emxvesa {vesa-mode}
set terminal vgal
```

The only option is the vesa mode for **emxvesa**, which defaults to G640x480x256.

83.19 Epslatex

The **epslatex** driver generates output for further processing by LaTeX.

Syntax:

```
set terminal epslatex   {default}
set terminal epslatex   {standalone | input}
                        {oldstyle | newstyle}
                        {level1 | leveldefault}
                        {color | colour | monochrome}
                        {solid | dashed}
```

```
{dashlength | dl <DL>}
{linewidth | lw <LW>}
{rounded | butt}
{clip | noclip}
{palfuncparam <samples>{,<maxdeviation>}}
{size <XX>{unit},<YY>{unit}}
{header <header> | noheader}
{blacktext | colortext | colourtext}
{{font} "fontname{,fontsize}" {<fontsize>}}
```

The epslatex terminal prints a plot as **terminal postscript eps** but transfers the texts to LaTeX instead of including in the PostScript code. Thus, many options are the same as in the **postscript terminal**.

The appearance of the epslatex terminal changed between versions 4.0 and 4.2 to reach better consistency with the postscript terminal: The plot size has been changed from 5 x 3 inches to 5 x 3.5 inches; the character width is now estimated to be 60% of the font size while the old epslatex terminal used 50%; now, the larger number of postscript linetypes and symbols are used. To reach an appearance that is nearly identical to the old one specify the option **oldstyle**. (In fact some small differences remain: the symbol sizes are slightly different, the tics are half as large as in the old terminal which can be changed using **set tics scale**, and the arrows have all features as in the postscript terminal.)

If you see the error message

```
"Can't find PostScript prologue file ... "
```

Please see and follow the instructions in **postscript prologue (p. 181)**.

The option **color** enables color, while **monochrome** prefers black and white drawing elements. Further, **monochrome** uses gray **palette** but it does not change color of objects specified with an explicit **colorspec**. **solid** draws all plots with solid lines, overriding any dashed patterns. **dashlength** or **dl** scales the length of the dashed-line segments by <DL>, which is a floating-point number greater than zero. **linewidth** or **lw** scales all linewidths by <LW>.

By default the generated PostScript code uses language features that were introduced in PostScript Level 2, notably filters and pattern-fill of irregular objects such as filledcurves. PostScript Level 2 features are conditionally protected so that PostScript Level 1 interpreters do not issue errors but, rather, display a message or a PostScript Level 1 approximation. The **level1** option substitutes PostScript Level 1 approximations of these features and uses no PostScript Level 2 code. This may be required by some old printers and old versions of Adobe Illustrator. The flag **level1** can be toggled later by editing a single line in the PostScript output file to force PostScript Level 1 interpretation. In the case of files containing level 2 code, the above features will not appear or will be replaced by a note when this flag is set or when the interpreting program does not indicate that it understands level 2 PostScript or higher.

rounded sets line caps and line joins to be rounded; **butt** is the default, butt caps and mitered joins.

clip tells PostScript to clip all output to the bounding box; **noclip** is the default.

palfuncparam controls how **set palette functions** are encoded as gradients in the output. Analytic color component functions (set via **set palette functions**) are encoded as linear interpolated gradients in the postscript output: The color component functions are sampled at <samples> points and all points are removed from this gradient which can be removed without changing the resulting colors by more than <maxdeviation>. For almost every useful palette you may savely leave the defaults of <samples>=2000 and <maxdeviation>=0.003 untouched.

The default size for postscript output is 10 inches x 7 inches. The default for eps output is 5 x 3.5 inches. The **size** option changes this to whatever the user requests. By default the X and Y sizes are taken to be in inches, but other units are possibly (currently only cm). The BoundingBox of the plot is correctly adjusted to contain the resized image. Screen coordinates always run from 0.0 to 1.0 along the full length of the plot edges as specified by the **size** option. NB: **this is a change from the previously recommended method of using the set size command prior to setting the terminal type**. The old method left the BoundingBox unchanged and screen coordinates did not correspond to the actual limits of the plot.

blacktext forces all text to be written in black even in color mode;

The epslatex driver offers a special way of controlling text positioning: (a) If any text string begins with '{', you also need to include a '}' at the end of the text, and the whole text will be centered both horizontally and vertically by LaTeX. (b) If the text string begins with '[', you need to continue it with: a position specification (up to two out of t,b,l,r,c), '\]{', the text itself, and finally, '}'. The text itself may be anything LaTeX can typeset as an LR-box. \rule{}{}'s may help for best positioning. See also the documentation for the **pslatex**

(p. 182) terminal driver. To create multiline labels, use \shortstack, for example

```
set ylabel '[r]{\shortstack{first line \\ second line}}'
```

The **back** option of **set label** commands is handled slightly different than in other terminals. Labels using 'back' are printed behind all other elements of the plot while labels using 'front' are printed above everything else.

The driver produces two different files, one for the eps part of the figure and one for the LaTeX part. The name of the LaTeX file is taken from the **set output** command. The name of the eps file is derived by replacing the file extension (normally **.tex**) with **.eps** instead. There is no LaTeX output if no output file is given! Remember to close the **output file** before next plot unless in **multiplot** mode.

In your LaTeX documents use '\input{filename}' to include the figure. The **.eps** file is included by the command \includegraphics{...}, so you must also include \usepackage{graphicx} in the LaTeX preamble. If you want to use coloured text (option **textcolour**) you also have to include \usepackage{color} in the LaTeX preamble.

Pdf files can be made from the eps file using 'epstopdf'. If the graphics package is properly configured, the LaTeX files can also be processed by pdflatex without changes, using the pdf files instead of the eps files. The behaviour concerning font selection depends on the header mode. In all cases, the given font size is used for the calculation of proper spacing. When not using the **standalone** mode the actual LaTeX font and font size at the point of inclusion is taken, so use LaTeX commands for changing fonts. If you use e.g. 12pt as font size for your LaTeX document, use '"" 12' as options. The font name is ignored. If using **standalone** the given font and font size are used, see below for a detailed description.

If text is printed coloured is controlled by the TeX booleans \ifGPcolor and \ifGPblacktext. Only if \ifGPcolor is true and \ifGPblacktext is false, text is printed coloured. You may either change them in the generated TeX file or provide them globally in your TeX file, for example by using

```
\newif\ifGPblacktext
\GPblacktexttrue
```

in the preamble of your document. The local assignment is only done if no global value is given.

When using the epslatex terminal give the name of the TeX file in the **set output** command including the file extension (normally ".tex"). The eps filename is generated by replacing the extension by ".eps".

If using the **standalone** mode a complete LaTeX header is added to the LaTeX file; and "-inc" is added to the filename of the eps file. The **standalone** mode generates a TeX file that produces output with the correct size when using dvips, pdfTeX, or VTeX. The default, **input**, generates a file that has to be included into a LaTeX document using the \input command.

If a font other than "" or "default" is given it is interpreted as LaTeX font name. It contains up to three parts, separated by a comma: 'fontname,fontseries,fontshape'. If the default fontshape or fontseries are requested, they can be omitted. Thus, the real syntax for the fontname is '[fontname][,fontseries][,fontshape]'. The naming convention for all parts is given by the LaTeX font scheme. The fontname is 3 to 4 characters long and is built as follows: One character for the font vendor, two characters for the name of the font, and optionally one additional character for special fonts, e.g., 'j' for fonts with old-style numerals or 'x' for expert fonts. The names of many fonts is described in

http://www.tug.org/fontname/fontname.pdf

For example, 'cmr' stands for Computer Modern Roman, 'ptm' for Times-Roman, and 'phv' for Helvetica. The font series denotes the thickness of the glyphs, in most cases 'm' for normal ("medium") and 'bx' or 'b' for bold fonts. The font shape is 'n' for upright, 'it' for italics, 'sl' for slanted, or 'sc' for small caps, in general. Some fonts may provide different font series or shapes.

Examples:

Use Times-Roman boldface (with the same shape as in the surrounding text):

```
set terminal epslatex 'ptm,bx'
```

Use Helvetica, boldface, italics:

```
set terminal epslatex 'phv,bx,it'
```

Continue to use the surrounding font in slanted shape:

```
set terminal epslatex ',,sl'
```

Use small capitals:

```
set terminal epslatex ',,sc'
```

By this method, only text fonts are changed. If you also want to change the math fonts you have to use the "gnuplot.cfg" file or the **header** option, described below.

In standalone mode, the font size is taken from the given font size in the **set terminal** command. To be able to use a specified font size, a file "size<size>.clo" has to reside in the LaTeX search path. By default, 10pt, 11pt, and 12pt are supported. If the package "extsizes" is installed, 8pt, 9pt, 14pt, 17pt, and 20pt are added.

The **header** option takes a string as argument. This string is written into the generated LaTeX file. If using the **standalone** mode, it is written into the preamble, directly before the \begin{document} command. In the **input** mode, it is placed directly after the \begingroup command to ensure that all settings are local to the plot.

Examples:

Use T1 fontencoding, change the text and math font to Times-Roman as well as the sans-serif font to Helvetica:

```
set terminal epslatex standalone header \
"\\usepackage[T1]{fontenc}\n\\usepackage{mathptmx}\n\\usepackage{helvet}"
```

Use a boldface font in the plot, not influencing the text outside the plot:

```
set terminal epslatex input header "\\bfseries"
```

If the file "gnuplot.cfg" is found by LaTeX it is input in the preamble the LaTeX document, when using **standalone** mode. It can be used for further settings, e.g., changing the document font to Times-Roman, Helvetica, and Courier, including math fonts (handled by "mathptmx.sty"):

```
\usepackage{mathptmx}
\usepackage[scaled=0.92]{helvet}
\usepackage{courier}
```

The file "gnuplot.cfg" is loaded before the header information given by the **header** command. Thus, you can use **header** to overwrite some of settings performed using "gnuplot.cfg"

83.20 Epson_180dpi

This driver supports a family of Epson printers and derivatives.

epson_180dpi and **epson_60dpi** are drivers for Epson LQ-style 24-pin printers with resolutions of 180 and 60 dots per inch, respectively.

epson_lx800 is a generic 9-pin driver appropriate for printers like the Epson LX-800, the Star NL-10 and NX-1000, the PROPRINTER, and so forth.

nec_cp6 is generic 24-pin driver that can be used for printers like the NEC CP6 and the Epson LQ-800.

The **okidata** driver supports the 9-pin OKIDATA 320/321 Standard printers.

The **starc** driver is for the Star Color Printer.

The **tandy_60dpi** driver is for the Tandy DMP-130 series of 9-pin, 60-dpi printers.

The **dpu414** driver is for the Seiko DPU-414 thermal printer.

nec_cp6 has the options:

Syntax:

```
set terminal nec_cp6 {monochrome | colour | draft}
```

which defaults to monochrome.

dpu414 has the options:

Syntax:

```
set terminal dpu414 {small | medium | large} {normal | draft}
```

which defaults to medium (=font size) and normal. Preferred combinations are **medium normal** and **small draft**.

83.21 Excl

The **excl** terminal driver supports Talaris printers such as the EXCL Laser printer and the 1590. It has no options.

83.22 Fig

The **fig** terminal device generates output in the Fig graphics language.

Syntax:

```
set terminal fig {monochrome | color}
                 {landscape | portrait}
                 {small | big | size <xsize> <ysize>}
                 {metric | inches}
                 {pointsmax <max_points>}
                 {solid | dashed}
                 {font <fontname>} {fontsize <fsize>}
                 {textnormal | {textspecial texthidden textrigid}}
                 {{thickness|linewidth} <units>}
                 {depth <layer>}
                 {version <number>}
```

monochrome and **color** determine whether the picture is black-and-white or **color**. **small** and **big** produce a 5x3 or 8x5 inch graph in the default **landscape** mode and 3x5 or 5x8 inches in **portrait** mode. **size** sets (overrides) the size of the drawing area to <xsize>*<ysize> in units of inches or centimeters depending on the **inches** or **metric** setting in effect. The latter settings is also used as default units for editing with "xfig".

pointsmax <max_points> sets the maximum number of points per polyline.

solid inhibits automatic usage of **dash**ed lines when solid linestyles are used up, which otherwise occurs.

fontsize sets the size of the text font to <fsize> points. **textnormal** resets the text flags and selects postscript fonts, **textspecial** sets the text flags for LaTeX specials, **texthidden** sets the hidden flag and **textrigid** the rigid flag.

depth sets the default depth layer for all lines and text. The default depth is 10 to leave room for adding material with "xfig" on top of the plot.

version sets the format version of the generated fig output. Currently only versions 3.1 and 3.2 are supported.

thickness sets the default line thickness, which is 1 if not specified. Overriding the thickness can be achieved by adding a multiple of 100 to the **linetype** value for a **plot** command. In a similar way the **depth** of plot elements (with respect to the default depth) can be controlled by adding a multiple of 1000 to <linetype>. The depth is then <layer> + <linetype>/1000 and the thickness is (<linetype>%1000)/100 or, if that is zero, the default line thickness. **linewidth** is a synonym for **thickness**.

Additional point-plot symbols are also available with the **fig** driver. The symbols can be used through **pointtype** values % 100 above 50, with different fill intensities controlled by <pointtype> % 5 and outlines in black (for <pointtype> % 10 < 5) or in the current color. Available symbols are

```
50 - 59:  circles
60 - 69:  squares
70 - 79:  diamonds
80 - 89:  upwards triangles
90 - 99:  downwards triangles
```

The size of these symbols is linked to the font size. The depth of symbols is by default one less than the depth for lines to achieve nice error bars. If <pointtype> is above 1000, the depth is <layer> + <pointtype>/1000-1. If <pointtype>%1000 is above 100, the fill color is (<pointtype>%1000)/100-1.

Available fill colors are (from 1 to 9): black, blue, green, cyan, red, magenta, yellow, white and dark blue (in monochrome mode: black for 1 to 6 and white for 7 to 9).

See **plot with (p. 77)** for details of <linetype> and <pointtype>.

The **big** option is a substitute for the **bfig** terminal in earlier versions, which is no longer supported.

Examples:

```
set terminal fig monochrome small pointsmax 1000  # defaults

plot 'file.dat' with points linetype 102 pointtype 759
```

would produce circles with a blue outline of width 1 and yellow fill color.

```
plot 'file.dat' using 1:2:3 with err linetype 1 pointtype 554
```

would produce errorbars with black lines and circles filled red. These circles are one layer above the lines (at depth 9 by default).

To plot the error bars on top of the circles use

```
plot 'file.dat' using 1:2:3 with err linetype 1 pointtype 2554
```

83.23 Ggi

The **ggi** driver can run on different targets as X or svgalib.

Syntax:

```
set terminal ggi [acceleration <integer>] [[mode] {mode}]
```

In X the window cannot be resized using window manager handles, but the mode can be given with the mode option, e.g.:

```
- V1024x768
- V800x600
- V640x480
- V320x200
```

Please refer to the ggi documentation for other modes. The 'mode' keyword is optional. It is recommended to select the target by environment variables as explained in the libggi manual page. To get DGA on X, you should for example

```
bash> export GGI_DISPLAY=DGA
csh>  setenv GGI_DISPLAY DGA
```

'acceleration' is only used for targets which report relative pointer motion events (e.g. DGA) and is a strictly positive integer multiplication factor for the relative distances. The default for acceleration is 7.

Examples:

```
set term ggi acc 10
set term ggi acc 1 mode V1024x768
set term ggi V1024x768
```

83.24 Gif

Syntax:

```
set terminal gif
       {{no}transparent} {rounded|butt}
       {linewidth <lw>} {dashlength <dl>}
       {tiny | small | medium | large | giant}
       {font "<face> {,<pointsize>}"} {{no}enhanced}
       {size <x>,<y>} {{no}crop}
       {animate {delay <d>} {loop <n>} {{no}optimize}}
       {<background_color>}
```

PNG, JPEG and GIF images are created using the external library libgd. GIF plots may be viewed interactively by piping the output to the 'display' program from the ImageMagick package as follows:

```
set term gif
set output '| display gif:-'
```

You can view the output from successive plot commands interactively by typing <space> in the display window. To save the current plot to a file, left click in the display window and choose **save**.

transparent instructs the driver to make the background color transparent. Default is **notransparent**.

The **linewidth** and **dashlength** options are scaling factors that affect all lines drawn, i.e. they are multiplied by values requested in various drawing commands.

butt instructs the driver to use a line drawing method that does not overshoot the desired end point of a line. This setting is only applicable for line widths greater than 1. This setting is most useful when drawing horizontal or vertical lines. Default is **rounded**.

The details of font selection are complicated. Two equivalent simple examples are given below:

```
set term gif font arial 11
set term gif font "arial,11"
```

For more information please see the separate section under **fonts (p. 27)**.

The **animate** option is available only if your local gd library supports the creation of animated gifs. The default delay between display of successive images may be specified in units of 1/100 second (default 5). The actual delay may vary depending on the program used as a viewer. Number of animation loops can be specified, default 0 means infinity. An animation sequence is terminated by the next **set output** or **set term** command. The **optimize** option has two effects on the animation.

1) A single color map is used for the entire animation. This requires that all colors used in any frame of the animation are already defined in the first frame.

2) If possible, only the portions of a frame that differ from the previous frame are stored in the animation file. This space saving may not be possible if the animation uses transparency.

Both of these optimizations are intended to produce a smaller output file, but the decrease in size is probably only significant for long animations or very small frame sizes. The **nooptimize** option turns off both of the effects just described. Each frame is stored in its entirety along with a private color map. Note that it is possible to post-process a non-optimized animation using external utilities, and this post-processing can yield a smaller file than gnuplot's internal optimization mode. The default is **nooptimize**.

The output plot size <x,y> is given in pixels — it defaults to 640x480. Please see additional information under **canvas (p. 16)** and **set size (p. 125)**. Blank space at the edges of the finished plot may be trimmed using the **crop** option, resulting in a smaller final image size. Default is **nocrop**.

The background color must be given in the form 'xrrggbb', where x is the literal character 'x' and 'rrggbb' are the red, green and blue components in hexadecimal. For example, 'x00ff00' is green. The specification of additional colors other than the background is deprecated.

83.24.1 Examples

```
set terminal gif medium size 640,480 xffffff
```

Use the medium size built-in non-scaleable, non-rotatable font. Use white (xffffff) for the non-transparent background.

```
set terminal gif font arial 14 enhanced
```

Searches for a scalable font with face name 'arial' and sets the font size to 14pt. Please see **fonts (p. 27)** for details of how the font search is done. Because this is a scalable font, we can use enhanced text mode.

```
set term gif animate transparent opt delay 10 size 200,200 x000000
load "animate2.dem"
```

Open the gif terminal for creation of an animated gif file. The individual frames of the animation sequence are created by the script file animate2.dem from the standard collection of demos.

83.25 Gnugraph(GNU plotutils)

The **gnugraph** driver produces device-independent output in the GNU plot graphics language. The default size of the PostScript results generated by "plot2ps" is 5 x 3 inches; this can be increased up to about 8.25 x 8.25 by **set size**.

Syntax:

```
set terminal gnugraph {"<fontname>"} {<fontsize>}
                      {type <pt>} {size "<size>"}
```

which defaults to 10-point "Courier".

For **type**, the following options are accepted: **X**, **pnm**, **gif**, **ai**, **ps**, **cgm**, **fig**, **pcl5**, **hpgl**, **tek**, and **meta** (default). The **size** option (default is a4) is passed straight through to plotutils, it's the user's responsibility to provide correct values. Details can be found in the plotutils documentation.

Examples:

```
set terminal gnugraph type hpgl size "a4"
set terminal gnugraph size "a4,xoffset=-5mm,yoffset=2.0cm" type pnm
```

There is a non-GNU version of the **gnugraph** driver which cannot be compiled unless this version is left out.

83.26 Gpic

The **gpic** terminal driver generates GPIC graphs in the Free Software Foundations's "groff" package. The default size is 5 x 3 inches. The only option is the origin, which defaults to (0,0).

Syntax:

```
set terminal gpic {<x> <y>}
```

where **x** and **y** are in inches.

A simple graph can be formatted using

```
groff -p -mpic -Tps file.pic > file.ps.
```

The output from pic can be pipe-lined into eqn, so it is possible to put complex functions in a graph with the **set label** and **set {x/y}label** commands. For instance,

```
set ylab '@space 0 int from 0 to x alpha ( t ) roman d t@'
```

will label the y axis with a nice integral if formatted with the command:

```
gpic filename.pic | geqn -d@@ -Tps | groff -m[macro-package] -Tps
    > filename.ps
```

Figures made this way can be scaled to fit into a document. The pic language is easy to understand, so the graphs can be edited by hand if need be. All co-ordinates in the pic-file produced by **gnuplot** are given as x+gnuplotx and y+gnuploty. By default x and y are given the value 0. If this line is removed with an editor in a number of files, one can put several graphs in one figure like this (default size is 5.0x3.0 inches):

```
.PS 8.0
x=0;y=3
copy "figa.pic"
x=5;y=3
copy "figb.pic"
x=0;y=0
copy "figc.pic"
x=5;y=0
copy "figd.pic"
.PE
```

This will produce an 8-inch-wide figure with four graphs in two rows on top of each other.

One can also achieve the same thing by the command

```
set terminal gpic x y
```

for example, using

```
.PS 6.0
copy "trig.pic"
.PE
```

83.27 Gpr

The **gpr** terminal driver supports the Apollo Graphics Primitive Resource for a fixed-size window. It has no options.

If a variable window size is desired, use the **apollo** terminal instead.

83.28 Grass

The **grass** terminal driver gives **gnuplot** capabilities to users of the GRASS geographic information system. Contact grassp-list@moon.cecer.army.mil for more information. Pages are written to the current frame of the GRASS Graphics Window. There are no options.

83.29 Hercules

These drivers supports PC monitors with autodetected graphics boards. They can be used only when compiled with Zortech C/C++. None have options.

83.30 Hp2623a

The **hp2623a** terminal driver supports the Hewlett Packard HP2623A. It has no options.

83.31 Hp2648

The **hp2648** terminal driver supports the Hewlett Packard HP2647 and HP2648. It has no options.

83.32 Hp500c

The **hp500c** terminal driver supports the Hewlett Packard HP DeskJet 500c. It has options for resolution and compression.

Syntax:

```
set terminal hp500c {<res>} {<comp>}
```

where **res** can be 75, 100, 150 or 300 dots per inch and **comp** can be "rle", or "tiff". Any other inputs are replaced by the defaults, which are 75 dpi and no compression. Rasterization at the higher resolutions may require a large amount of memory.

83.33 Hpgl

The **hpgl** driver produces HPGL output for devices like the HP7475A plotter. There are two options which can be set: the number of pens and **eject**, which tells the plotter to eject a page when done. The default is to use 6 pens and not to eject the page when done.

The international character sets ISO-8859-1 and CP850 are recognized via **set encoding iso_8859_1** or **set encoding cp850** (see **set encoding (p. 96)** for details).

Syntax:

```
set terminal hpgl {<number_of_pens>} {eject}
```

The selection

```
set terminal hpgl 8 eject
```

is equivalent to the previous **hp7550** terminal, and the selection

```
set terminal hpgl 4
```

is equivalent to the previous **hp7580b** terminal.

The **pcl5** driver supports plotters such as the Hewlett-Packard Designjet 750C, the Hewlett-Packard Laserjet III, and the Hewlett-Packard Laserjet IV. It actually uses HPGL-2, but there is a name conflict among the terminal devices. It has several options which must be specified in the order indicated below:

Syntax:

```
set terminal pcl5 {mode <mode>} {<plotsize>}
    {{color {<number_of_pens>}} | monochrome} {solid | dashed}
    {font <font>} {size <fontsize>} {pspoints | nopspoints}
```

<mode> is **landscape** or **portrait**. <plotsize> is the physical plotting size of the plot, which is one of the following: **letter** for standard (8 1/2" X 11") displays, **legal** for (8 1/2" X 14") displays, **noextended** for (36" X 48") displays (a letter size ratio) or, **extended** for (36" X 55") displays (almost a legal size ratio). **color** is for multi-pen (i.e. color) plots, and <number_of_pens> is the number of pens (i.e. colors) used in color plots. **monochrome** is for one (e.g. black) pen plots. **solid** draws all lines as solid lines, or **dashed** will draw lines with different dashed and dotted line patterns. <font> is **stick**, **univers**, **cg_times**, **zapf_dingbats**, **antique_olive**, **arial**, **courier**, **garamond_antigua**, **letter_gothic**, **cg_omega**, **albertus**, **times_new_roman**, **clarendon**,

coronet, **marigold**, **truetype_symbols**, or **wingdings**. <fontsize> is the font size in points. The point type selection can be the standard default set by specifying **nopspoints**, or the same set of point types found in the postscript terminal by specifying **pspoints**.

Note that built-in support of some of these options is printer device dependent. For instance, all the fonts are supposedly supported by the HP Laserjet IV, but only a few (e.g. univers, stick) may be supported by the HP Laserjet III and the Designjet 750C. Also, color obviously won't work on the the laserjets since they are monochrome devices.

Defaults: landscape, noextended, color (6 pens), solid, univers, 12 point,
```
and nopspoints.
```

With **pcl5** international characters are handled by the printer; you just put the appropriate 8-bit character codes into the text strings. You don't need to bother with **set encoding**.

HPGL graphics can be imported by many software packages.

83.34 Hpljii

The **hpljii** terminal driver supports the HP Laserjet Series II printer. The **hpdj** driver supports the HP DeskJet 500 printer. These drivers allow a choice of resolutions.

Syntax:
```
set terminal hpljii | hpdj {<res>}
```

where **res** may be 75, 100, 150 or 300 dots per inch; the default is 75. Rasterization at the higher resolutions may require a large amount of memory.

The **hp500c** terminal is similar to **hpdj**; **hp500c** additionally supports color and compression.

83.35 Hppj

The **hppj** terminal driver supports the HP PaintJet and HP3630 printers. The only option is the choice of font.

Syntax:
```
set terminal hppj {FNT5X9 | FNT9X17 | FNT13X25}
```

with the middle-sized font (FNT9X17) being the default.

83.36 Imagen

The **imagen** terminal driver supports Imagen laser printers. It is capable of placing multiple graphs on a single page.

Syntax:
```
set terminal imagen {<fontsize>} {portrait | landscape}
                    {[<horiz>,<vert>]}
```

where **fontsize** defaults to 12 points and the layout defaults to **landscape**. <**horiz**> and <**vert**> are the number of graphs in the horizontal and vertical directions; these default to unity.

Example:
```
set terminal imagen portrait [2,3]
```

puts six graphs on the page in three rows of two in portrait orientation.

83.37 Jpeg

Syntax:
```
set terminal jpeg
       {{no}interlace}
       {linewidth <lw>} {dashlength <dl>} {rounded|butt}
       {tiny | small | medium | large | giant}
       {font "<face> {,<pointsize>}"} {{no}enhanced}
       {size <x>,<y>} {{no}crop}
       {<background_color>}
```

PNG, JPEG and GIF images are created using the external library libgd. In most cases, PNG is to be preferred for single plots, and GIF for animations. Both are loss-less image formats, and produce better image quality than the lossy JPEG format. This is in particular noticeable for solid color lines against a solid background, i.e. exactly the sort of image typically created by gnuplot.

The **interlace** option creates a progressive JPEG image. Default is **nointerlace**.

The **linewidth** and **dashlength** options are scaling factors that affect all lines drawn, i.e. they are multiplied by values requested in various drawing commands.

butt instructs the driver to use a line drawing method that does not overshoot the desired end point of a line. This setting is only applicable for line widths greater than 1. This setting is most useful when drawing horizontal or vertical lines. Default is **rounded**.

The details of font selection are complicated. Two equivalent simple examples are given below:

```
set term jpeg font arial 11
set term jpeg font "arial,11"
```

For more information please see the separate section under **fonts (p. 27)**.

The output plot size <x,y> is given in pixels — it defaults to 640x480. Please see additional information under **canvas (p. 16)** and **set size (p. 125)**. Blank space at the edges of the finished plot may be trimmed using the **crop** option, resulting in a smaller final image size. Default is **nocrop**.

The background color must be given in the form 'xrrggbb', where x is the literal character 'x' and 'rrggbb' are the red, green and blue components in hexadecimal. For example, 'x00ff00' is green. The specification of additional colors other than the background is deprecated.

83.38 Kyo

The **kyo** and **prescribe** terminal drivers support the Kyocera laser printer. The only difference between the two is that **kyo** uses "Helvetica" whereas **prescribe** uses "Courier". There are no options.

83.39 Latex

Syntax:

```
set terminal {latex | emtex} {default | {courier|roman} {<fontsize>}}
             {size <XX>{unit}, <YY>{unit}} {rotate | norotate}
```

By default the plot will inherit font settings from the embedding document. You have the option of forcing either Courier (cmtt) or Roman (cmr) fonts instead. In this case you may also specify a fontsize. Unless your driver is capable of building fonts at any size (e.g. dvips), stick to the standard 10, 11 and 12 point sizes.

METAFONT users beware: METAFONT does not like odd sizes.

All drivers for LaTeX offer a special way of controlling text positioning: If any text string begins with '{', you also need to include a '}' at the end of the text, and the whole text will be centered both horizontally and vertically. If the text string begins with '[', you need to follow this with a position specification (up to two out of t,b,l,r), ']{', the text itself, and finally '}'. The text itself may be anything LaTeX can typeset as an LR-box. '\rule{}{}'s may help for best positioning.

Points, among other things, are drawn using the LaTeX commands "\Diamond" and "\Box". These commands no longer belong to the LaTeX2e core; they are included in the latexsym package, which is part of the base distribution and thus part of any LaTeX implementation. Please do not forget to use this package. Other point types use symbols from the amssymb package.

The default size for the plot is 5 inches by 3 inches. The **size** option changes this to whatever the user requests. By default the X and Y sizes are taken to be in inches, but other units are possible (currently only cm).

If 'rotate' is specified, rotated text, especially a rotated y-axis label, is possible (the packages graphics or graphicx are needed). The 'stacked' y-axis label mechanism is then deactivated.

Examples: About label positioning: Use gnuplot defaults (mostly sensible, but sometimes not really best):

```
set title '\LaTeX\ -- $ \gamma $'
```

Force centering both horizontally and vertically:

```
set label '{\LaTeX\ -- $ \gamma $}' at 0,0
```

Specify own positioning (top here):

```
set xlabel '[t]{\LaTeX\ -- $ \gamma $}'
```

The other label – account for long ticlabels:

```
set ylabel '[r]{\LaTeX\ -- $ \gamma $\rule{7mm}{0pt}}'
```

83.40 Linux

The **linux** driver has no additional options to specify. It looks at the environment variable GSVGAMODE for the default mode; if not set, it uses 1024x768x256 as default mode or, if that is not possible, 640x480x16 (standard VGA).

83.41 Macintosh

Syntax:

```
set terminal macintosh {singlewin | multiwin} {vertical | novertical}
                       {size <width>, <height> | default}
```

'singlewin' limits the output to a single window and is useful for animations. 'multiwin' allows multiple windows. 'vertical' is only valid under the gx option. With this option, rotated text will be drawn vertically. novertical turns this option off. size <width>, <height> overrides the graph size set in the preferences dialog until it is cleared with either 'set term mac size default' or 'set term mac default'.

'set term mac size default' sets the window size settings to those set in the preferences dialog.

'set term mac default' sets all options to their default values. Default values: nogx, multiwin, novertical.

If you generate graphs under the multiwin option and then switch to singlewin, the next plot command will cause one more window to be created. This new window will be reused as long as singlewin is in effect. If you switch back to multiwin, generate some graphs, and then switch to singlewin again, the orginal 'singlewin' window will be resused if it is still open. Otherwise a new 'singlewin' window will be created. The 'singlewin' window is not numbered.

83.42 Mf

The **mf** terminal driver creates an input file to the METAFONT program. Thus a figure may be used in the TeX document in the same way as is a character.

To use a picture in a document, the METAFONT program must be run with the output file from **gnuplot** as input. Thus, the user needs a basic knowledge of the font creating process and the procedure for including a new font in a document. However, if the METAFONT program is set up properly at the local site, an unexperienced user could perform the operation without much trouble.

The text support is based on a METAFONT character set. Currently the Computer Modern Roman font set is input, but the user is in principal free to choose whatever fonts he or she needs. The METAFONT source files for the chosen font must be available. Each character is stored in a separate picture variable in METAFONT. These variables may be manipulated (rotated, scaled etc.) when characters are needed. The drawback is the interpretation time in the METAFONT program. On some machines (i.e. PC) the limited amount of memory available may also cause problems if too many pictures are stored.

The **mf** terminal has no options.

83.42.1 METAFONT Instructions

- Set your terminal to METAFONT:

```
set terminal mf
```

- Select an output-file, e.g.:

```
set output "myfigures.mf"
```

- Create your pictures. Each picture will generate a separate character. Its default size will be 5*3 inches. You can change the size by saying **set size 0.5,0.5** or whatever fraction of the default size you want to have.

- Quit **gnuplot**.

- Generate a TFM and GF file by running METAFONT on the output of **gnuplot**. Since the picture is quite large (5*3 in), you will have to use a version of METAFONT that has a value of at least 150000 for memmax. On Unix systems these are conventionally installed under the name bigmf. For the following assume that the command virmf stands for a big version of METAFONT. For example:

- Invoke METAFONT:

```
virmf '&plain'
```

- Select the output device: At the METAFONT prompt ('*') type:

```
\mode:=CanonCX;     % or whatever printer you use
```

- Optionally select a magnification:

```
mag:=1;             % or whatever you wish
```

- Input the **gnuplot**-file:

```
input myfigures.mf
```

On a typical Unix machine there will usually be a script called "mf" that executes virmf '&plain', so you probably can substitute mf for virmf &plain. This will generate two files: mfput.tfm and mfput.$$$gf (where $$$ indicates the resolution of your device). The above can be conveniently achieved by typing everything on the command line, e.g.: virmf '&plain' '\mode:=CanonCX; mag:=1; input myfigures.mf' In this case the output files will be named myfigures.tfm and myfigures.300gf.

- Generate a PK file from the GF file using gftopk:

```
gftopk myfigures.300gf myfigures.300pk
```

The name of the output file for gftopk depends on the DVI driver you use. Ask your local TeX administrator about the naming conventions. Next, either install the TFM and PK files in the appropriate directories, or set your environment variables properly. Usually this involves setting TEXFONTS to include the current directory and doing the same thing for the environment variable that your DVI driver uses (no standard name here...). This step is necessary so that TeX will find the font metric file and your DVI driver will find the PK file.

- To include your pictures in your document you have to tell TeX the font:

```
\font\gnufigs=myfigures
```

Each picture you made is stored in a single character. The first picture is character 0, the second is character 1, and so on... After doing the above step, you can use the pictures just like any other characters. Therefore, to place pictures 1 and 2 centered in your document, all you have to do is:

```
\centerline{\gnufigs\char0}
\centerline{\gnufigs\char1}
```

in plain TeX. For LaTeX you can, of course, use the picture environment and place the picture wherever you wish by using the \makebox and \put macros.

This conversion saves you a lot of time once you have generated the font; TeX handles the pictures as characters and uses minimal time to place them, and the documents you make change more often than the pictures do. It also saves a lot of TeX memory. One last advantage of using the METAFONT driver is that the DVI file really remains device independent, because no \special commands are used as in the eepic and tpic drivers.

83.43 Mgr

The **mgr** terminal driver supports the Mgr Window system. It has no options.

83.44 Mif

The **mif** terminal driver produces Frame Maker MIF format version 3.00. It plots in MIF Frames with the size 15*10 cm, and plot primitives with the same pen will be grouped in the same MIF group. Plot primitives in a **gnuplot** page will be plotted in a MIF Frame, and several MIF Frames are collected in one large MIF Frame. The MIF font used for text is "Times".

Several options may be set in the MIF 3.00 driver.

Syntax:

```
set terminal mif {color | colour | monochrome} {polyline | vectors}
                 {help | ?}
```

colour plots lines with line types >= 0 in colour (MIF sep. 2–7) and **monochrome** plots all line types in black (MIF sep. 0). **polyline** plots curves as continuous curves and **vectors** plots curves as collections of vectors. **help** and **?** print online help on standard error output — both print a short description of the usage; **help** also lists the options.

Examples:

```
set term mif colour polylines    # defaults
set term mif                     # defaults
set term mif vectors
set term mif help
```

83.45 Mp

The **mp** driver produces output intended to be input to the Metapost program. Running Metapost on the file creates EPS files containing the plots. By default, Metapost passes all text through TeX. This has the advantage of allowing essentially any TeX symbols in titles and labels.

Syntax:

```
set term mp {color | colour | monochrome}
            {solid | dashed}
            {notex | tex | latex}
            {magnification <magsize>}
            {psnfss | psnfss-version7 | nopsnfss}
            {prologues <value>}
            {a4paper}
            {amstex}
            {"<fontname>"} {<fontsize>}
```

The option **color** causes lines to be drawn in color (on a printer or display that supports it), **monochrome** (or nothing) selects black lines. The option **solid** draws solid lines, while **dashed** (or nothing) selects lines with different patterns of dashes. If **solid** is selected but **color** is not, nearly all lines will be identical. This may occasionally be useful, so it is allowed.

The option **notex** bypasses TeX entirely, therefore no TeX code can be used in labels under this option. This is intended for use on old plot files or files that make frequent use of common characters like **$** and **%** that require special handling in TeX.

The option **tex** sets the terminal to output its text for TeX to process.

The option **latex** sets the terminal to output its text for processing by LaTeX. This allows things like \frac for fractions which LaTeX knows about but TeX does not. Note that you must set the environment variable TEX to the name of your LaTeX executable (normally latex) if you use this option or use **mpost –tex=<name of LaTeX executable> ....** Otherwise metapost will try and use TeX to process the text and it won't work.

Changing font sizes in TeX has no effect on the size of mathematics, and there is no foolproof way to make such a change, except by globally setting a magnification factor. This is the purpose of the **magnification** option. It must be followed by a scaling factor. All text (NOT the graphs) will be scaled by this factor. Use this if you have math that you want at some size other than the default 10pt. Unfortunately, all math will be the same size, but see the discussion below on editing the MP output. **mag** will also work under **notex** but there seems no point in using it as the font size option (below) works as well.

The option **psnfss** uses postscript fonts in combination with LaTeX. Since this option only makes sense, if LaTeX is being used, the **latex** option is selected automatically. This option includes the following packages for LaTeX: inputenc(latin1), fontenc(T1), mathptmx, helvet(scaled=09.2), courier, latexsym and textcomp.

The option **psnfss-version7** uses also postscript fonts in LaTeX (option **latex** is also automatically selected), but uses the following packages with LaTeX: inputenc(latin1), fontenc(T1), times, mathptmx, helvet and courier.

The option **nopsnfss** is the default and uses the standard font (cmr10 if not otherwise specified).

The option **prologues** takes a value as an additional argument and adds the line **prologues:=<value>** to the metapost file. If a value of **2** is specified metapost uses postscript fonts to generate the eps-file, so that the result can be viewed using e.g. ghostscript. Normally the output of metapost uses TeX fonts and therefore has to be included in a (La)TeX file before you can look at it.

The option **noprologues** is the default. No additional line specifying the prologue will be added.

The option **a4paper** adds a **[a4paper]** to the documentclass. Normally letter paper is used (default). Since this option is only used in case of LaTeX, the **latex** option is selected automatically.

The option **amstex** automatically selects the **latex** option and includes the following LaTeX packages: amsfonts, amsmath(intlimits). By default these packages are not included.

A name in quotes selects the font that will be used when no explicit font is given in a **set label** or **set title**. A name recognized by TeX (a TFM file exists) must be used. The default is "cmr10" unless **notex** is selected, then it is "pcrr8r" (Courier). Even under **notex**, a TFM file is needed by Metapost. The file **pcrr8r.tfm** is the name given to Courier in LaTeX's psnfss package. If you change the font from the **notex** default, choose a font that matches the ASCII encoding at least in the range 32-126. **cmtt10** almost works, but it has a nonblank character in position 32 (space).

The size can be any number between 5.0 and 99.99. If it is omitted, 10.0 is used. It is advisable to use **magstep** sizes: 10 times an integer or half-integer power of 1.2, rounded to two decimals, because those are the most available sizes of fonts in TeX systems.

All the options are optional. If font information is given, it must be at the end, with size (if present) last. The size is needed to select a size for the font, even if the font name includes size information. For example, **set term mp "cmtt12"** selects cmtt12 shrunk to the default size 10. This is probably not what you want or you would have used cmtt10.

The following common ascii characters need special treatment in TeX:

```
$, &, #, %, _;  |, <, >;  ^, ~,  \, {, and }
```

The five characters $, #, &, _, and % can simply be escaped, e.g., **\$**. The three characters <, >, and | can be wrapped in math mode, e.g., **$<$**. The remainder require some TeX work-arounds. Any good book on TeX will give some guidance.

If you type your labels inside double quotes, backslashes in TeX code need to be escaped (doubled). Using single quotes will avoid having to do this, but then you cannot use **\n** for line breaks. As of this writing, version 3.7 of gnuplot processes titles given in a **plot** command differently than in other places, and backslashes in TeX commands need to be doubled regardless of the style of quotes.

Metapost pictures are typically used in TeX documents. Metapost deals with fonts pretty much the same way TeX does, which is different from most other document preparation programs. If the picture is included in a LaTeX document using the graphics package, or in a plainTeX document via epsf.tex, and then converted to PostScript with dvips (or other dvi-to-ps converter), the text in the plot will usually be handled correctly. However, the text may not appear if you send the Metapost output as-is to a PostScript interpreter.

83.45.1 Metapost Instructions

- Set your terminal to Metapost, e.g.:

```
set terminal mp mono "cmtt12" 12
```

- Select an output-file, e.g.:

```
set output "figure.mp"
```

- Create your pictures. Each plot (or multiplot group) will generate a separate Metapost beginfig...endfig group. Its default size will be 5 by 3 inches. You can change the size by saying **set size 0.5,0.5** or whatever fraction of the default size you want to have.

- Quit gnuplot.

- Generate EPS files by running Metapost on the output of gnuplot:

```
mpost figure.mp  OR  mp figure.mp
```

The name of the Metapost program depends on the system, typically **mpost** for a Unix machine and **mp** on many others. Metapost will generate one EPS file for each picture.

- To include your pictures in your document you can use the graphics package in LaTeX or epsf.tex in plainTeX:

```
\usepackage{graphics} % LaTeX
\input epsf.tex       % plainTeX
```

If you use a driver other than dvips for converting TeX DVI output to PS, you may need to add the following line in your LaTeX document:

```
\DeclareGraphicsRule{*}{eps}{*}{}
```

Each picture you made is in a separate file. The first picture is in, e.g., figure.0, the second in figure.1, and so on.... To place the third picture in your document, for example, all you have to do is:

```
\includegraphics{figure.2} % LaTeX
\epsfbox{figure.2}         % plainTeX
```

The advantage, if any, of the mp terminal over a postscript terminal is editable output. Considerable effort went into making this output as clean as possible. For those knowledgeable in the Metapost language, the default line types and colors can be changed by editing the arrays **lt**[] and **col**[]. The choice of solid vs dashed lines, and color vs black lines can be change by changing the values assigned to the booleans **dashedlines** and **colorlines**. If the default **tex** option was in effect, global changes to the text of labels can be achieved by editing the **vebatimtex...etex** block. In particular, a LaTeX preamble can be added if desired, and then LaTeX's built-in size changing commands can be used for maximum flexibility. Be sure to set the appropriate MP configuration variable to force Metapost to run LaTeX instead of plainTeX.

83.46 Next

Several options may be set in the next driver.

Syntax:

```
set terminal next {<mode>} {<type> } {<color>} {<dashed>}
           {"<fontname>"} {<fontsize>} title {"<newtitle>"}
```

where <mode> is **default**, which sets all options to their defaults; <type> is either **new** or **old**, where **old** invokes the old single window; <color> is either **color** or **monochrome**; <dashed> is either **solid** or **dashed**; "<fontname>" is the name of a valid PostScript font; <fontsize> is the size of the font in PostScript points; and <title> is the title for the GnuTerm window. Defaults are **new**, **monochrome**, **dashed**, "Helvetica", 14pt.

Examples:

```
set term next default
set term next 22
set term next color "Times-Roman" 14
set term next color "Helvetica" 12 title "MyPlot"
set term next old
```

Pointsizes may be changed with **set linestyle**.

83.47 Openstep (next)

Several options may be set in the openstep (next) driver.

Syntax:

```
set terminal openstep {<mode>} {<type> } {<color>} {<dashed>}
           {"<fontname>"} {<fontsize>} title {"<newtitle>"}
```

where <mode> is **default**, which sets all options to their defaults; <type> is either **new** or **old**, where **old** invokes the old single window; <color> is either **color** or **monochrome**; <dashed> is either **solid** or **dashed**; "<fontname>" is the name of a valid PostScript font; <fontsize> is the size of the font in PostScript points; and <title> is the title for the GnuTerm window. Defaults are **new**, **monochrome**, **dashed**, "Helvetica", 14pt.

Examples:

```
set term openstep default
set term openstep 22
set term openstep color "Times-Roman" 14
set term openstep color "Helvetica" 12 title "MyPlot"
set term openstep old
```

Pointsizes may be changed with **set linestyle**.

83.48 Pbm

Syntax:

```
set terminal pbm {<fontsize>} {<mode>} {size <x>,<y>}
```

where <fontsize> is **small**, **medium**, or **large** and <mode> is **monochrome**, **gray** or **color**. The default plot size is 640 pixels wide and 480 pixels high. The output size is white-space padded to the nearest multiple of 8 pixels on both x and y. This empty space may be cropped later if needed.

The output of the **pbm** driver depends upon <mode>: **monochrome** produces a portable bitmap (one bit per pixel), **gray** a portable graymap (three bits per pixel) and **color** a portable pixmap (color, four bits per pixel).

The output of this driver can be used with various image conversion and manipulation utilities provided by NETPBM. Based on Jef Poskanzer's PBMPLUS package, NETPBM provides programs to convert the above PBM formats to GIF, TIFF, MacPaint, Macintosh PICT, PCX, X11 bitmap and many others. Complete information is available at http://netpbm.sourceforge.net/.

Examples:

```
set terminal pbm small monochrome                # defaults
set terminal pbm color medium size 800,600
set output '| pnmrotate 45 | pnmtopng > tilted.png'  # uses NETPBM
```

83.49 Pdf

This terminal produces files in the Adobe Portable Document Format (PDF), useable for printing or display with tools like Acrobat Reader

Syntax:

```
set terminal pdf {monochrome|color|colour}
                 {{no}enhanced}
                 {fname "<font>"} {fsize <fontsize>}
                 {font "<fontname>{,<fontsize>}"}
                 {linewidth <lw>} {rounded|butt}
                 {solid|dashed} {dl <dashlength>}}
                 {size <XX>{unit},<YY>{unit}}
```

The default is to use a different color for each line type. Selecting **monochome** will use black for all linetypes, in which case you probably want to select **dashed** to distinguish line types. Even in in mono mode you can still use explicit colors for filled areas or linestyles.

where <font> is the name of the default font to use (default Helvetica) and <fontsize> is the font size (in points, default 12). For help on which fonts are available or how to install new ones, please see the documentation for your local installation of pdflib.

The **enhanced** option enables enhanced text processing features (subscripts, superscripts and mixed fonts). See **enhanced (p. 20)**.

The width of all lines in the plot can be increased by the factor <n> specified in **linewidth**. Similarly **dashlength** is a multiplier for the default dash spacing.

rounded sets line caps and line joins to be rounded; **butt** is the default, butt caps and mitered joins.

The default size for PDF output is 5 inches by 3 inches. The **size** option changes this to whatever the user requests. By default the X and Y sizes are taken to be in inches, but other units are possible (currently only cm).

83.50 Pdfcairo

The **pdfcairo** terminal device generates output in pdf. The actual drawing is done via cairo, a 2D graphics library, and pango, a library for laying out and rendering text.

Syntax:

```
set term pdfcairo
            {{no}enhanced} {mono|color} {solid|dashed}
            {font <font>}
            {linewidth <lw>} {rounded|butt} {dashlength <dl>}
            {size <XX>{unit},<YY>{unit}}
```

This terminal supports an enhanced text mode, which allows font and other formatting commands (subscripts, superscripts, etc.) to be embedded in labels and other text strings. The enhanced text mode syntax is shared with other gnuplot terminal types. See **enhanced (p. 20)** for more details.

The width of all lines in the plot can be modified by the factor <lw> specified in **linewidth**. The default linewidth is 0.25 points. (1 "PostScript" point = 1/72 inch = 0.353 mm)

rounded sets line caps and line joins to be rounded; **butt** is the default, butt caps and mitered joins.

The default size for the output is 5 inches x 3 inches. The **size** option changes this to whatever the user requests. By default the X and Y sizes are taken to be in inches, but other units are possibly (currently only cm). Screen coordinates always run from 0.0 to 1.0 along the full length of the plot edges as specified by the **size** option.

<font> is in the format "FontFace,FontSize", i.e. the face and the size comma-separated in a single string. FontFace is a usual font face name, such as 'Arial'. If you do not provide FontFace, the pdfcairo terminal will use 'Sans'. FontSize is the font size, in points. If you do not provide it, the pdfcairo terminal will use a size of 6 points.

```
For example :
   set term pdfcairo font "Arial,12"
   set term pdfcairo font "Arial" # to change the font face only
   set term pdfcairo font ",12" # to change the font size only
   set term pdfcairo font "" # to reset the font name and size
```

The fonts are retrieved from the usual fonts subsystems. Under Windows, those fonts are to be found and configured in the entry "Fonts" of the control panel. Under UNIX, they are handled by "fontconfig".

Pango, the library used to layout the text, is based on utf-8. Thus, the pdfcairo terminal has to convert from your encoding to utf-8. The default input encoding is based on your 'locale'. If you want to use another encoding, make sure gnuplot knows which one you are using. See **encoding (p. 96)** for more details.

Pango may give unexpected results with fonts that do not respect the unicode mapping. With the Symbol font, for example, the pdfcairo terminal will use the map provided by http://www.unicode.org/ to translate character codes to unicode. Note that "the Symbol font" is to be understood as the Adobe Symbol font, distributed with Acrobat Reader as "SY______.PFB". Alternatively, the OpenSymbol font, distributed with OpenOffice.org as "opens___.ttf", offers the same characters. Microsoft has distributed a Symbol font ("symbol.ttf"), but it has a different character set with several missing or moved mathematic characters. If you experience problems with your default setup (if the demo enhancedtext.dem is not displayed properly for example), you probably have to install one of the Adobe or OpenOffice Symbol fonts, and remove the Microsoft one. Other non-conform fonts, such as "wingdings" have been observed working.

The rendering of the plot cannot be altered yet. To obtain the best output possible, the rendering involves two mechanisms : antialiasing and oversampling. Antialiasing allows to display non-horizontal and non-vertical lines smoother. Oversampling combined with antialiasing provides subpixel accuracy, so that gnuplot can draw a line from non-integer coordinates. This avoids wobbling effects on diagonal lines ('plot x' for example).

83.51 Pm

The **pm** terminal driver provides an OS/2 Presentation Manager window in which the graph is plotted. The window is opened when the first graph is plotted. This window has its own online help as well as facilities for printing, copying to the clipboard and some line type and color adjustments. The **multiplot** option is supported.

Syntax:

```
set terminal pm {server {n}} {persist} {widelines} {enhanced} {"title"}
```

If **persist** is specified, each graph appears in its own window and all windows remain open after **gnuplot** exits. If **server** is specified, all graphs appear in the same window, which remains open when **gnuplot** exits. This option takes an optional numerical argument which specifies an instance of the server process. Thus multiple server windows can be in use at the same time.

If **widelines** is specified, all plots will be drawn with wide lines. If **enhanced** is specified, sub- and superscripts and multiple fonts are enabled (see **enhanced text (p. 20)** for details). Font names for the core PostScript fonts may be abbreviated to a single letter (T/H/C/S for Times/Helvetica/Courier/Symbol).

If **title** is specified, it will be used as the title of the plot window. It will also be used as the name of the server instance, and will override the optional numerical argument.

Linewidths may be changed with **set linestyle**.

83.52 Png

Syntax:

```
set terminal png
       {{no}transparent} {{no}interlace}
       {{no}truecolor} {rounded|butt}
       {linewidth <lw>} {dashlength <dl>}
       {tiny | small | medium | large | giant}
       {font "<face> {,<pointsize>}"} {{no}enhanced}
       {size <x>,<y>} {{no}crop}
       {<background_color>}
```

PNG, JPEG and GIF images are created using the external library libgd. PNG plots may be viewed interactively by piping the output to the 'display' program from the ImageMagick package as follows:

```
set term png
set output '| display png:-'
```

You can view the output from successive plot commands interactively by typing <space> in the display window. To save the current plot to a file, left click in the display window and choose **save**.

transparent instructs the driver to make the background color transparent. Default is **notransparent**.

interlace instructs the driver to generate interlaced PNGs. Default is **nointerlace**.

The **linewidth** and **dashlength** options are scaling factors that affect all lines drawn, i.e. they are multiplied by values requested in various drawing commands.

By default output png images use 256 indexed colors. The **truecolor** option instead creates TrueColor images with 24 bits of color information per pixel. Transparent fill styles require the **truecolor** option. See **fillstyle** **(p. 127)**. A transparent background is possible in either indexed or TrueColor images.

butt instructs the driver to use a line drawing method that does not overshoot the desired end point of a line. This setting is only applicable for line widths greater than 1. This setting is most useful when drawing horizontal or vertical lines. Default is **rounded**.

The details of font selection are complicated. Two equivalent simple examples are given below:

```
set term png font arial 11
set term png font "arial,11"
```

For more information please see the separate section under **fonts** **(p. 27)**.

The output plot size <x,y> is given in pixels — it defaults to 640x480. Please see additional information under **canvas** **(p. 16)** and **set size** **(p. 125)**. Blank space at the edges of the finished plot may be trimmed using the **crop** option, resulting in a smaller final image size. Default is **nocrop**.

The background color must be given in the form 'xrrggbb', where x is the literal character 'x' and 'rrggbb' are the red, green and blue components in hexadecimal. For example, 'x00ff00' is green. The specification of additional colors other than the background is deprecated.

83.52.1 Examples

```
set terminal png medium size 640,480 xffffff
```

Use the medium size built-in non-scaleable, non-rotatable font. Use white (xffffff) for the non-transparent background.

```
set terminal png font arial 14 size 800,600
```

Searches for a scalable font with face name 'arial' and sets the font size to 14pt. Please see **fonts** **(p. 27)** for details of how the font search is done.

```
set terminal png transparent truecolor enhanced
```

Use 24 bits of color information per pixel, with a transparent background. Use the **enhanced text** mode to control the layout of strings to be printed.

83.53 Pngcairo

The **pngcairo** terminal device generates output in png. The actual drawing is done via cairo, a 2D graphics library, and pango, a library for laying out and rendering text.

Syntax:

```
set term pngcairo
             {{no}enhanced} {mono|color} {solid|dashed}
             {{no}transparent} {{no}crop} {font <font>}
             {linewidth <lw>} {rounded|butt} {dashlength <dl>}
             {size <XX>{unit},<YY>{unit}}
```

This terminal supports an enhanced text mode, which allows font and other formatting commands (subscripts, superscripts, etc.) to be embedded in labels and other text strings. The enhanced text mode syntax is shared with other gnuplot terminal types. See **enhanced (p. 20)** for more details.

The width of all lines in the plot can be modified by the factor <lw> specified in **linewidth**. The default linewidth is 0.25 points. (1 "PostScript" point = 1/72 inch = 0.353 mm)

rounded sets line caps and line joins to be rounded; **butt** is the default, butt caps and mitered joins.

The default size for the output is 5 inches x 3 inches. The **size** option changes this to whatever the user requests. By default the X and Y sizes are taken to be in inches, but other units are possibly (currently only cm). Screen coordinates always run from 0.0 to 1.0 along the full length of the plot edges as specified by the **size** option.

<font> is in the format "FontFace,FontSize", i.e. the face and the size comma-separated in a single string. FontFace is a usual font face name, such as 'Arial'. If you do not provide FontFace, the pngcairo terminal will use 'Sans'. FontSize is the font size, in points. If you do not provide it, the pngcairo terminal will use a size of 6 points.

```
For example :
   set term pngcairo font "Arial,12"
   set term pngcairo font "Arial" # to change the font face only
   set term pngcairo font ",12" # to change the font size only
   set term pngcairo font "" # to reset the font name and size
```

The fonts are retrieved from the usual fonts subsystems. Under Windows, those fonts are to be found and configured in the entry "Fonts" of the control panel. Under UNIX, they are handled by "fontconfig".

Pango, the library used to layout the text, is based on utf-8. Thus, the pngcairo terminal has to convert from your encoding to utf-8. The default input encoding is based on your 'locale'. If you want to use another encoding, make sure gnuplot knows which one you are using. See **encoding (p. 96)** for more details.

Pango may give unexpected results with fonts that do not respect the unicode mapping. With the Symbol font, for example, the pngcairo terminal will use the map provided by http://www.unicode.org/ to translate character codes to unicode. Note that "the Symbol font" is to be understood as the Adobe Symbol font, distributed with Acrobat Reader as "SY______.PFB". Alternatively, the OpenSymbol font, distributed with OpenOffice.org as "opens___.ttf", offers the same characters. Microsoft has distributed a Symbol font ("symbol.ttf"), but it has a different character set with several missing or moved mathematic characters. If you experience problems with your default setup (if the demo enhancedtext.dem is not displayed properly for example), you probably have to install one of the Adobe or OpenOffice Symbol fonts, and remove the Microsoft one. Other non-conform fonts, such as "wingdings" have been observed working.

The rendering of the plot cannot be altered yet. To obtain the best output possible, the rendering involves two mechanisms : antialiasing and oversampling. Antialiasing allows to display non-horizontal and non-vertical lines smoother. Oversampling combined with antialiasing provides subpixel accuracy, so that gnuplot can draw a line from non-integer coordinates. This avoids wobbling effects on diagonal lines ('plot x' for example).

83.54 Postscript

Several options may be set in the **postscript** driver.

Syntax:

```
set terminal postscript {default}
set terminal postscript {landscape | portrait | eps}
                        {enhanced | noenhanced}
                        {defaultplex | simplex | duplex}
```

```
{fontfile [add | delete] "<filename>"
 | nofontfiles} {{no}adobeglyphnames}
{level1 | leveldefault}
{color | colour | monochrome}
{solid | dashed}
{dashlength | dl <DL>}
{linewidth | lw <LW>}
{rounded | butt}
{clip | noclip}
{palfuncparam <samples>{,<maxdeviation>}}
{size <XX>{unit},<YY>{unit}}
{blacktext | colortext | colourtext}
{{font} "fontname{,fontsize}" {<fontsize>}}
```

If you see the error message

```
"Can't find PostScript prologue file ... "
```

Please see and follow the instructions in **postscript prologue (p. 181)**.

landscape and **portrait** choose the plot orientation. **eps** mode generates EPS (Encapsulated PostScript) output, which is just regular PostScript with some additional lines that allow the file to be imported into a variety of other applications. (The added lines are PostScript comment lines, so the file may still be printed by itself.) To get EPS output, use the **eps** mode and make only one plot per file. In **eps** mode the whole plot, including the fonts, is reduced to half of the default size.

enhanced enables enhanced text mode features (subscripts, superscripts and mixed fonts). See **enhanced (p. 20)** for more information. **blacktext** forces all text to be written in black even in color mode;

Duplexing in PostScript is the ability of the printer to print on both sides of the same sheet of paper. With **defaultplex**, the default setting of the printer is used; with **simplex** only one side is printed; **duplex** prints on both sides (ignored if your printer can't do it).

"<**fontname**>" is the name of a valid PostScript font; and <**fontsize**> is the size of the font in PostScript points. In addition to the standard postscript fonts, an oblique version of the Symbol font, useful for mathematics, is defined. It is called "Symbol-Oblique".

default sets all options to their defaults: **landscape**, **monochrome**, **dashed**, **dl 1.0**, **lw 1.0**, **defaultplex**, **noenhanced**, "Helvetica" and 14pt. Default size of a PostScript plot is 10 inches wide and 7 inches high. The option **color** enables color, while **monochrome** prefers black and white drawing elements. Further, **monochrome** uses gray **palette** but it does not change color of objects specified with an explicit **colorspec**. **solid** draws all plots with solid lines, overriding any dashed patterns. **dashlength** or **dl** scales the length of the dashed-line segments by <DL>, which is a floating-point number greater than zero. **linewidth** or **lw** scales all linewidths by <LW>.

By default the generated PostScript code uses language features that were introduced in PostScript Level 2, notably filters and pattern-fill of irregular objects such as filledcurves. PostScript Level 2 features are conditionally protected so that PostScript Level 1 interpreters do not issue errors but, rather, display a message or a PostScript Level 1 approximation. The **level1** option substitutes PostScript Level 1 approximations of these features and uses no PostScript Level 2 code. This may be required by some old printers and old versions of Adobe Illustrator. The flag **level1** can be toggled later by editing a single line in the PostScript output file to force PostScript Level 1 interpretation. In the case of files containing level 2 code, the above features will not appear or will be replaced by a note when this flag is set or when the interpreting program does not indicate that it understands level 2 PostScript or higher.

rounded sets line caps and line joins to be rounded; **butt** is the default, butt caps and mitered joins.

clip tells PostScript to clip all output to the bounding box; **noclip** is the default.

palfuncparam controls how **set palette functions** are encoded as gradients in the output. Analytic color component functions (set via **set palette functions**) are encoded as linear interpolated gradients in the postscript output: The color component functions are sampled at <samples> points and all points are removed from this gradient which can be removed without changing the resulting colors by more than <maxdeviation>. For almost every useful palette you may savely leave the defaults of <samples>=2000 and <maxdeviation>=0.003 untouched.

The default size for postscript output is 10 inches x 7 inches. The default for eps output is 5 x 3.5 inches. The **size** option changes this to whatever the user requests. By default the X and Y sizes are taken to be in inches, but other units are possibly (currently only cm). The BoundingBox of the plot is correctly adjusted to

contain the resized image. Screen coordinates always run from 0.0 to 1.0 along the full length of the plot edges as specified by the **size** option. NB: **this is a change from the previously recommended method of using the set size command prior to setting the terminal type**. The old method left the BoundingBox unchanged and screen coordinates did not correspond to the actual limits of the plot.

Fonts listed by **fontfile** or **fontfile add** encapsulate the font definitions of the listed font from a postscript Type 1 or TrueType font file directly into the gnuplot output postscript file. Thus, the enclosed font can be used in labels, titles, etc. See the section **postscript fontfile (p. 180)** for more details. With **fontfile delete**, a fontfile is deleted from the list of embedded files. **nofontfiles** cleans the list of embedded fonts.

Examples:

```
set terminal postscript default       # old postscript
set terminal postscript enhanced      # old enhpost
set terminal postscript landscape 22  # old psbig
set terminal postscript eps 14        # old epsf1
set terminal postscript eps 22        # old epsf2
set size 0.7,1.4; set term post portrait color "Times-Roman" 14
set term post "VAGRoundedBT_Regular" 14 fontfile "bvrr8a.pfa"
```

Linewidths and pointsizes may be changed with **set style line**.

The **postscript** driver supports about 70 distinct pointtypes, selectable through the **pointtype** option on **plot** and **set style line**.

Several possibly useful files about **gnuplot**'s PostScript are included in the /docs/psdoc subdirectory of the **gnuplot** distribution and at the distribution sites. These are "ps_symbols.gpi" (a **gnuplot** command file that, when executed, creates the file "ps_symbols.ps" which shows all the symbols available through the **postscript** terminal), "ps_guide.ps" (a PostScript file that contains a summary of the enhanced syntax and a page showing what the octal codes produce with text and symbol fonts), "ps_file.doc" (a text file that contains a discussion of the organization of a PostScript file written by **gnuplot**), and "ps_fontfile_doc.tex" (a LaTeX file which contains a short documentation concerning the encapsulation of LaTeX fonts with a glyph table of the math fonts).

A PostScript file is editable, so once **gnuplot** has created one, you are free to modify it to your heart's desire. See the **editing postscript (p. 180)** section for some hints.

83.54.1 Editing postscript

The PostScript language is a very complex language — far too complex to describe in any detail in this document. Nevertheless there are some things in a PostScript file written by **gnuplot** that can be changed without risk of introducing fatal errors into the file.

For example, the PostScript statement "/Color true def" (written into the file in response to the command **set terminal postscript color**), may be altered in an obvious way to generate a black-and-white version of a plot. Similarly line colors, text colors, line weights and symbol sizes can also be altered in straight-forward ways. Text (titles and labels) can be edited to correct misspellings or to change fonts. Anything can be repositioned, and of course anything can be added or deleted, but modifications such as these may require deeper knowledge of the PostScript language.

The organization of a PostScript file written by **gnuplot** is discussed in the text file "ps_file.doc" in the docs/ps subdirectory of the gnuplot source distribution.

83.54.2 Postscript fontfile

The **fontfile** or **fontfile add** option takes one file name as argument and encapsulates this file into the postscript output in order to make this font available for text elements (labels, tic marks, titles, etc.). The **fontfile delete** option also takes one file name as argument. It deletes this file name from the list of encapsulated files.

The postscript terminal understands some font file formats: Type 1 fonts in ASCII file format (extension ".pfa"), Type 1 fonts in binary file format (extension ".pfb"), and TrueType fonts (extension ".ttf"). Pfa files are understood directly, pfb and ttf files are converted on the fly if appropriate conversion tools are installed (see below). You have to specify the full filename including the extension. Each **fontfile** option takes exact one font file name. This option can be used multiple times in order to include more than one font file.

The font file is searched in the working directory and in all directories listed in the fontpath which is determined by **set fontpath**. In addition, the fontpath can be set using the environment variable GNUPLOT_FONTPATH. If this is not set a system dependent default search list is used. See **set fontpath (p. 97)** for more details.

For using the encapsulated font file you have to specify the font name (which normally is not the same as the file name). When embedding a font file by using the **fontfile** option in interactive mode, the font name is printed on the screen. E.g.

```
Font file 'p052004l.pfb' contains the font 'URWPalladioL-Bold'. Location:
/usr/lib/X11/fonts/URW/p052004l.pfb
```

When using pfa or pfb fonts, you can also find it out by looking into the font file. There is a line similar to "/FontName /URWPalladioL-Bold def". The middle string without the slash is the fontname, here "URWPalladioL-Bold". For TrueType fonts, this is not so easy since the font name is stored in a binary format. In addition, they often have spaces in the font names which is not supported by Type 1 fonts (in which a TrueType is converted on the fly). The font names are changed in order to eliminate the spaces in the fontnames. The easiest way to find out which font name is generated for use with gnuplot, start gnuplot in interactive mode and type in "set terminal postscript fontfile '<filename.ttf>'".

For converting font files (either ttf or pfb) to pfa format, the conversion tool has to read the font from a file and write it to standard output. If the output cannot be written to standard output, on-the-fly conversion is not possible.

For pfb files "pfbtops" is a tool which can do this. If this program is installed on your system the on the fly conversion should work. Just try to encapsulate a pfb file. If the compiled in program call does not work correctly you can specify how this program is called by defining the environment variable GNUPLOT_PFBTOPFA e.g. to "pfbtops %s". The **%s** will be replaced by the font file name and thus has to exist in the string.

If you don't want to do the conversion on the fly but get a pfa file of the font you can use the tool "pfb2pfa" which is written in simple c and should compile with any c compiler. It is available from many ftp servers, e.g.

ftp://ftp.dante.de/tex-archive/fonts/utilities/ps2mf/

In fact, "pfbtopfa" and "pfb2ps" do the same job. "pfbtopfa" puts the resulting pfa code into a file, whereas "pfbtops" writes it to standard output.

TrueType fonts are converted into Type 1 pfa format, e.g. by using the tool "ttf2pt1" which is available from

http://ttf2pt1.sourceforge.net/

If the builtin conversion does not work, the conversion command can be changed by the environment variable GNUPLOT_TTFTOPFA. For usage with ttf2pt1 it may be set to "ttf2pt1 -a -e -W 0 %s - ". Here again, **%s** stands for the file name.

For special purposes you also can use a pipe (if available for your operating system). Therefore you start the file name definition with the character "<" and append a program call. This program has to write pfa data to standard output. Thus, a pfa file may be accessed by **set fontfile** "**< cat garamond.pfa**".

For example, including Type 1 font files can be used for including the postscript output in LaTeX documents. The "european computer modern" font (which is a variant of the "computer modern" font) is available in pfb format from any CTAN server, e.g.

ftp://ftp.dante.de/tex-archive/fonts/ps-type1/cm-super/

For example, the file "sfrm1000.pfb" contains the normal upright fonts with serifs in the design size 10pt (font name "SFRM1000"). The computer modern fonts, which are still necessary for mathematics, are available from

ftp://ftp.dante.de/tex-archive/fonts/cm/ps-type1/bluesky

With these you can use any character available in TeX. However, the computer modern fonts have a strange encoding. (This is why you should not use cmr10.pfb for text, but sfrm1000.pfb instead.) The usage of TeX fonts is shown in one of the demos. The file "ps_fontfile_doc.tex" in the /docs/psdoc subdirectory of the **gnuplot** source distribution contains a table with glyphs of the TeX mathfonts.

If the font "CMEX10" is embedded (file "cmex10.pfb") gnuplot defines the additional font "CMEX10-Baseline". It is shifted vertically in order to fit better to the other glyphs (CMEX10 has its baseline at the top of the symbols).

83.54.3 Postscript prologue

Each PostScript output file includes a %%Prolog section and possibly some additional user-defined sections containing, for example, character encodings. These sections are copied from a set of PostScript prologue files which are either compiled in the gnuplot executable or stored elsewhere on your computer. This behaviour and the default directory where these files live are controlled at the time gnuplot is built. However, you can control this either by defining an environment variable GNUPLOT_PS_DIR or by using the gnuplot command

set loadpath. See **set loadpath (p. 107)**.

83.54.4 Postscript adobeglyphnames

This setting is only relevant to PostScript output with UTF-8 encoding. It controls the names used to describe characters with Unicode entry points higher than 0x00FF. That is, all characters outside of the Latin1 set. In general unicode characters do not have a unique name; they have only a unicode identification code. However, Adobe have a recommended scheme for assigning names to certain ranges of characters (extended Latin, Greek, etc). Some fonts use this scheme, others do not. By default, gnuplot will use the Adobe glyph names. E.g. the lower case Greek letter alpha will be called /alpha. If you specific **noadobeglyphnames** then instead gnuplot will use /uni03B1 to describe this character. If you get this setting wrong, the character may not be found even if it is present in the font. It is probably always correct to use the default for Adobe fonts, but for other fonts you may have to try both settings. See also **fontfile (p. 180)**.

83.55 Pslatex and pstex

The **pslatex** driver generates output for further processing by LaTeX, while the **pstex** driver generates output for further processing by TeX. **pslatex** uses \specials understandable by dvips and xdvi. Figures generated by **pstex** can be included in any plain-based format (including LaTeX).

Syntax:

```
set terminal [pslatex | pstex] {default}
set terminal [pslatex | pstex]
                        {rotate | norotate}
                        {oldstyle | newstyle}
                        {auxfile | noauxfile}
                        {level1 | leveldefault}
                        {color | colour | monochrome}
                        {solid | dashed}
                        {dashlength | dl <DL>}
                        {linewidth | lw <LW>}
                        {rounded | butt}
                        {clip | noclip}
                        {palfuncparam <samples>{,<maxdeviation>}}
                        {size <XX>{unit},<YY>{unit}}
                        {<font_size>}
```

If you see the error message

```
"Can't find PostScript prologue file ... "
```

Please see and follow the instructions in **postscript prologue (p. 181)**.

The option **color** enables color, while **monochrome** prefers black and white drawing elements. Further, **monochrome** uses gray **palette** but it does not change color of objects specified with an explicit **colorspec**. **solid** draws all plots with solid lines, overriding any dashed patterns. **dashlength** or **dl** scales the length of the dashed-line segments by <DL>, which is a floating-point number greater than zero. **linewidth** or **lw** scales all linewidths by <LW>.

By default the generated PostScript code uses language features that were introduced in PostScript Level 2, notably filters and pattern-fill of irregular objects such as filledcurves. PostScript Level 2 features are conditionally protected so that PostScript Level 1 interpreters do not issue errors but, rather, display a message or a PostScript Level 1 approximation. The **level1** option substitutes PostScript Level 1 approximations of these features and uses no PostScript Level 2 code. This may be required by some old printers and old versions of Adobe Illustrator. The flag **level1** can be toggled later by editing a single line in the PostScript output file to force PostScript Level 1 interpretation. In the case of files containing level 2 code, the above features will not appear or will be replaced by a note when this flag is set or when the interpreting program does not indicate that it understands level 2 PostScript or higher.

rounded sets line caps and line joins to be rounded; **butt** is the default, butt caps and mitered joins.

clip tells PostScript to clip all output to the bounding box; **noclip** is the default.

palfuncparam controls how **set palette functions** are encoded as gradients in the output. Analytic color component functions (set via **set palette functions**) are encoded as linear interpolated gradients in the postscript

output: The color component functions are sampled at <samples> points and all points are removed from this gradient which can be removed without changing the resulting colors by more than <maxdeviation>. For almost every useful palette you may savely leave the defaults of <samples>=2000 and <maxdeviation>=0.003 untouched.

The default size for postscript output is 10 inches x 7 inches. The default for eps output is 5 x 3.5 inches. The **size** option changes this to whatever the user requests. By default the X and Y sizes are taken to be in inches, but other units are possibly (currently only cm). The BoundingBox of the plot is correctly adjusted to contain the resized image. Screen coordinates always run from 0.0 to 1.0 along the full length of the plot edges as specified by the **size** option. NB: **this is a change from the previously recommended method of using the set size command prior to setting the terminal type**. The old method left the BoundingBox unchanged and screen coordinates did not correspond to the actual limits of the plot.

if **rotate** is specified, the y-axis label is rotated. <font_size> is the size (in pts) of the desired font.

If **auxfile** is specified, it directs the driver to put the PostScript commands into an auxiliary file instead of directly into the LaTeX file. This is useful if your pictures are large enough that dvips cannot handle them. The name of the auxiliary PostScript file is derived from the name of the TeX file given on the **set output** command; it is determined by replacing the trailing **.tex** (actually just the final extent in the file name) with **.ps** in the output file name, or, if the TeX file has no extension, **.ps** is appended. The **.ps** is included into the **.tex** file by a \special{psfile=...} command. Remember to close the **output file** before next plot unless in **multiplot** mode.

Gnuplot versions prior to version 4.2 generated plots of the size 5 x 3 inches using the ps(la)tex terminal while the current version generates 5 x 3.5 inches to be consistent with the postscript eps terminal. In addition, the character width is now estimated to be 60% of the font size while the old epslatex terminal used 50%. To reach the old format specify the option **oldstyle**.

The pslatex driver offers a special way of controlling text positioning: (a) If any text string begins with '{', you also need to include a '}' at the end of the text, and the whole text will be centered both horizontally and vertically by LaTeX. (b) If the text string begins with '[', you need to continue it with: a position specification (up to two out of t,b,l,r), ']{', the text itself, and finally, '}'. The text itself may be anything LaTeX can typeset as an LR-box. \rule{}{}'s may help for best positioning.

The options not described here are identical to the **Postscript terminal**. Look there if you want to know what they do.

Examples:

```
set term pslatex monochrome dashed rotate       # set to defaults
```

To write the PostScript commands into the file "foo.ps":

```
set term pslatex auxfile
set output "foo.tex"; plot ...; set output
```

About label positioning: Use gnuplot defaults (mostly sensible, but sometimes not really best):

```
set title '\LaTeX\ -- $ \gamma $'
```

Force centering both horizontally and vertically:

```
set label '{\LaTeX\ -- $ \gamma $}' at 0,0
```

Specify own positioning (top here):

```
set xlabel '[t]{\LaTeX\ -- $ \gamma $}'
```

The other label – account for long ticlabels:

```
set ylabel '[r]{\LaTeX\ -- $ \gamma $\rule{7mm}{0pt}}'
```

Linewidths and pointsizes may be changed with **set style line**.

83.56 Pstricks

The **pstricks** driver is intended for use with the "pstricks.sty" macro package for LaTeX. It is an alternative to the **eepic** and **latex** drivers. You need "pstricks.sty", and, of course, a printer that understands PostScript, or a converter such as Ghostscript.

PSTricks is available via anonymous ftp from the /pub directory at Princeton.edu. This driver definitely does not come close to using the full capability of the PSTricks package.

Syntax:

```
set terminal pstricks {hacktext | nohacktext} {unit | nounit}
```

The first option invokes an ugly hack that gives nicer numbers; the second has to do with plot scaling. The defaults are **hacktext** and **nounit**.

83.57 Qms

The **qms** terminal driver supports the QMS/QUIC Laser printer, the Talaris 1200 and others. It has no options.

83.58 Regis

The **regis** terminal device generates output in the REGIS graphics language. It has the option of using 4 (the default) or 16 colors.

Syntax:

```
set terminal regis {4 | 16}
```

83.59 Rgip

The **rgip** and **uniplex** terminal drivers support RGIP metafiles. They can combine several graphs on a single page, but only one page is allowed in a given output file.

Syntax:

```
set terminal rgip | uniplex {portrait | landscape}
                            {[<horiz>,<vert>]} {<fontsize>}
```

permissible values for the font size are in the range 1–8, with the default being 1. The default layout is landscape. Graphs are placed on the page in a **horiz**x**vert** grid, which defaults to [1,1].

Example:

```
set terminal uniplex portrait [2,3]
```

puts six graphs on a page in three rows of two in portrait orientation.

83.60 Sun

The **sun** terminal driver supports the SunView window system. It has no options.

83.61 Svg

This terminal produces files in the W3C Scalable Vector Graphics format.

Syntax:

```
set terminal svg {size <x>,<y> {|fixed|dynamic}}
                 {{no}enhanced}
                 {fname "<font>"} {fsize <fontsize>}
                 {font "<fontname>{,<fontsize>}"}
                 {fontfile <filename>}
                 {rounded|butt} {solid|dashed} {linewidth <lw>}
```

where <x> and <y> are the size of the SVG plot to generate, **dynamic** allows a svg-viewer to resize plot, whereas the default setting, **fixed**, will request an absolute size.

linewidth <w> increases the width of all lines used in the figure by a factor of <w>.

<font> is the name of the default font to use (default Arial) and <fontsize> is the font size (in points, default 12). SVG viewing programs may substitute other fonts when the file is displayed.

The svg terminal supports an enhanced text mode, which allows font and other formatting commands to be embedded in labels and other text strings. The enhanced text mode syntax is shared with other gnuplot terminal types. See **enhanced (p. 20)** for more details.

SVG allows you to embed fonts directly into an SVG document, or to provide a hypertext link to the desired font. The **fontfile** option specifies a local file which is copied into the <defs> section of the resulting SVG output file. This file may either itself contain a font, or may contain the records necessary to create a hypertext reference to the desired font. Gnuplot will look for the requested file using the directory list in the GNUPLOT_FONTPATH environmental variable. NB: You must embed an svg font, not a TrueType or PostScript font.

83.62 Svga

The **svga** terminal driver supports PCs with SVGA graphics. It can only be used if it is compiled with DJGPP. Its only option is the font.

Syntax:

```
set terminal svga {"<fontname>"}
```

83.63 Tek40

This family of terminal drivers supports a variety of VT-like terminals. **tek40xx** supports Tektronix 4010 and others as well as most TEK emulators. **vttek** supports VT-like tek40xx terminal emulators. The following are present only if selected when gnuplot is built: **kc-tek40xx** supports MS-DOS Kermit Tek4010 terminal emulators in color; **km-tek40xx** supports them in monochrome. **selanar** supports Selanar graphics. **bitgraph** supports BBN Bitgraph terminals. None have any options.

83.64 Tek410x

The **tek410x** terminal driver supports the 410x and 420x family of Tektronix terminals. It has no options.

83.65 Texdraw

The **texdraw** terminal driver supports the LaTeX texdraw environment. It is intended for use with "texdraw.sty" and "texdraw.tex" in the texdraw package.

Points, among other things, are drawn using the LaTeX commands "\Diamond" and "\Box". These commands no longer belong to the LaTeX2e core; they are included in the latexsym package, which is part of the base distribution and thus part of any LaTeX implementation. Please do not forget to use this package.

It has no options.

83.66 Tgif

Tgif is an X11-based drawing tool — it has nothing to do with GIF.

The **tgif** driver supports different pointsizes (with **set pointsize**), different label fonts and font sizes (e.g. **set label "Hallo" at x,y font "Helvetica,34"**) and multiple graphs on the page. The proportions of the axes are not changed.

Syntax:

```
set terminal tgif {portrait | landscape | default} {<[x,y]>}
                  {monochrome | color}
                  {{linewidth | lw} <LW>}
                  {solid | dashed}
                  {font "<fontname>"} {<fontsize>}
```

where <[x,y]> specifies the number of graphs in the x and y directions on the page, **color** enables color, **linewidth** scales all linewidths by <LW>, "<fontname>" is the name of a valid PostScript font, and <fontsize> specifies the size of the PostScript font. **defaults** sets all options to their defaults: **portrait**, **[1,1]**, **color**, **linwidth 1.0**, **dashed**, **"Helvetica"**, and **18**.

The **solid** option is usually prefered if lines are colored, as they often are in the editor. Hardcopy will be black-and-white, so **dashed** should be chosen for that.

Multiplot is implemented in two different ways.

The first multiplot implementation is the standard gnuplot multiplot feature:

```
set terminal tgif
set output "file.obj"
set multiplot
set origin x01,y01
set size  xs,ys
plot ...
     ...
set origin x02,y02
plot ...
unset multiplot
```

See **set multiplot (p. 111)** for further information.

The second version is the [x,y] option for the driver itself. The advantage of this implementation is that everything is scaled and placed automatically without the need for setting origins and sizes; the graphs keep their natural x/y proportions of 3/2 (or whatever is fixed by **set size**).

If both multiplot methods are selected, the standard method is chosen and a warning message is given.

Examples of single plots (or standard multiplot):

```
set terminal tgif                  # defaults
set terminal tgif "Times-Roman" 24
set terminal tgif landscape
set terminal tgif landscape solid
```

Examples using the built-in multiplot mechanism:

```
set terminal tgif portrait [2,4]  # portrait; 2 plots in the x-
                                  # and 4 in the y-direction
set terminal tgif [1,2]           # portrait; 1 plot in the x-
                                  # and 2 in the y-direction
set terminal tgif landscape [3,3] # landscape; 3 plots in both
                                  # directions
```

83.67 Tkcanvas

This terminal driver generates Tk canvas widget commands based on Tcl/Tk (default) or Perl. To use it, rebuild **gnuplot** (after uncommenting or inserting the appropriate line in "term.h"), then

```
gnuplot> set term tkcanvas {perltk} {interactive}
gnuplot> set output 'plot.file'
```

After invoking "wish", execute the following sequence of Tcl/Tk commands:

```
% source plot.file
% canvas .c
% pack .c
% gnuplot .c
```

Or, for Perl/Tk use a program like this:

```
use Tk;
my $top = MainWindow->new;
my $c = $top->Canvas->pack;
my $gnuplot = do "plot.pl";
$gnuplot->($c);
MainLoop;
```

The code generated by **gnuplot** creates a procedure called "gnuplot" that takes the name of a canvas as its argument. When the procedure is called, it clears the canvas, finds the size of the canvas and draws the plot in it, scaled to fit.

For 2-dimensional plotting (**plot**) two additional procedures are defined: "gnuplot_plotarea" will return a list containing the borders of the plotting area "xleft, xright, ytop, ybot" in canvas screen coordinates, while the ranges of the two axes "x1min, x1max, y1min, y1max, x2min, x2max, y2min, y2max" in plot coordinates can be

obtained calling "gnuplot_axisranges". If the "interactive" option is specified, mouse clicking on a line segment will print the coordinates of its midpoint to stdout. Advanced actions can happen instead if the user supplies a procedure named "user_gnuplot_coordinates", which takes the following arguments: "win id x1s y1s x2s y2s x1e y1e x2e y2e x1m y1m x2m y2m", the name of the canvas and the id of the line segment followed by the coordinates of its start and end point in the two possible axis ranges; the coordinates of the midpoint are only filled for logarithmic axes.

The current version of **tkcanvas** supports neither **multiplot** nor **replot**.

83.68 Tpic

The **tpic** terminal driver supports the LaTeX picture environment with tpic \specials. It is an alternative to the **latex** and **eepic** terminal drivers. Options are the point size, line width, and dot-dash interval.

Syntax:

```
set terminal tpic <pointsize> <linewidth> <interval>
```

where **pointsize** and **linewidth** are integers in milli-inches and **interval** is a float in inches. If a non-positive value is specified, the default is chosen: pointsize = 40, linewidth = 6, interval = 0.1.

All drivers for LaTeX offer a special way of controlling text positioning: If any text string begins with '{', you also need to include a '}' at the end of the text, and the whole text will be centered both horizontally and vertically by LaTeX. — If the text string begins with '[', you need to continue it with: a position specification (up to two out of t,b,l,r), ']{', the text itself, and finally, '}'. The text itself may be anything LaTeX can typeset as an LR-box. \rule{}{}'s may help for best positioning.

Examples: About label positioning: Use gnuplot defaults (mostly sensible, but sometimes not really best):

```
set title '\LaTeX\ -- $ \gamma $'
```

Force centering both horizontally and vertically:

```
set label '{\LaTeX\ -- $ \gamma $}' at 0,0
```

Specify own positioning (top here):

```
set xlabel '[t]{\LaTeX\ -- $ \gamma $}'
```

The other label – account for long ticlabels:

```
set ylabel '[r]{\LaTeX\ -- $ \gamma $\rule{7mm}{0pt}}'
```

83.69 Unixpc

The **unixpc** terminal driver supports AT&T 3b1 and AT&T 7300 Unix PC. It has no options.

83.70 Unixplot

The **unixplot** terminal driver generates output in the Unix "plot" graphics language. It has no options.

This terminal cannot be compiled if the GNU version of plot is to be used; in that case, use the **gnugraph** terminal instead.

83.71 Vgagl

The **vgagl** driver is a fast linux console driver with full mouse and pm3d support. It looks at the environment variable SVGALIB_DEFAULT_MODE for the default mode; if not set, it uses a 256 color mode with the highest available resolution.

Syntax:

```
set terminal vgagl \
             background [red] [[green] [blue]] \
             [uniform | interpolate] \
             [mode]
```

The color mode can also be given with the mode option. Both Symbolic names as G1024x768x256 and integers are allowed. The **background** option takes either one or three integers in the range [0, 255]. If only one integers is supplied, it is taken as gray value for the background. If three integers are present, the background gets the corresponding color. The (mutually exclusive) options **interpolate** and **uniform** control if color interpolation is done while drawing triangles (on by default).

To get high resolution modes, you will probably have to modify the configuration file of libvga, usually /etc/vga/libvga.conf. Using the VESA fb is a good choice, but this needs to be compiled in the kernel.

The vgagl driver uses the first *available* vga mode from the following list:

```
- the driver which was supplied when setting vgagl, e.g. `set term vgagl
  G1024x768x256` would first check, if the G1024x768x256 mode is available.
- the environment variable SVGALIB_DEFAULT_MODE
- G1024x768x256
- G800x600x256
- G640x480x256
- G320x200x256
- G1280x1024x256
- G1152x864x256
- G1360x768x256
- G1600x1200x256
```

83.72 VWS

The **VWS** terminal driver supports the VAX Windowing System. It has no options. It will sense the display type (monochrome, gray scale, or color.) All line styles are plotted as solid lines.

83.73 Vx384

The **vx384** terminal driver supports the Vectrix 384 and Tandy color printers. It has no options.

83.74 Windows

Three options may be set in the **windows** terminal driver.

Syntax:

```
set terminal windows {color | monochrome}
                     {enhanced | noenhanced}
                     {{font} "fontname{,fontsize}" {<fontsize>}}
                     {title "Plot Window Title"}
                     {size <width>,<height>}
                     {position <x>,<y>}
                     {close}
```

where **color** and **monochrome** select colored or mono output, **enhanced** enables enhanced text mode features (subscripts, superscripts and mixed fonts). See **enhanced** **(p. 20)** for more information. "<**fontname**>" is the name of a valid Windows font, and <**fontsize**> is the size of the font in points. **size** defines the width and height of the window in pixel and **position** the origin of the window i.e. the position of the top left corner on the screen (again in pixel). Both these options override the default settings from the WGNUPLOT.INI file (see below).

Other options may be set with the graph-menu or the initialization file.

The Windows version normally terminates immediately as soon as the end of any files given as command line arguments is reached (i.e. in non-interactive mode), unless you specify **-** as the last command line option. It will also not show the text-window at all, in this mode, only the plot. By giving the optional argument **-persist** (same as for gnuplot under x11; former Windows-only options **/noend** or **-noend** are still accepted as well), will not close gnuplot. Contrary to gnuplot on other operating systems, gnuplot's interactive command line is accessible after the -persist option.

The plot window remains open even when the gnuplot driver is changed to a different device. The plot window can be close with **set term windows close**

83.74.1 Graph-menu

The **gnuplot graph** window has the following options on a pop-up menu accessed by pressing the right mouse button or selecting **Options** from the system menu:

Bring to Top when checked brings the graph window to the top after every plot.

Color when checked enables color linestyles. When unchecked it forces monochrome linestyles.

Copy to Clipboard copies a bitmap and an enhanced Metafile picture.

Save as EMF... allows the user to save the current graph window as enhanced metafile

Background... sets the window background color.

Choose Font... selects the font used in the graphics window.

Line Styles... allows customization of the line colors and styles.

Print... prints the graphics windows using a Windows printer driver and allows selection of the printer and scaling of the output. The output produced by **Print** is not as good as that from **gnuplot**'s own printer drivers.

Update wgnuplot.ini saves the current window locations, window sizes, text window font, text window font size, graph window font, graph window font size, background color and linestyles to the initialization file **WGNUPLOT.INI**.

83.74.2 Printing

In order of preference, graphs may be be printed in the following ways.

1. Use the **gnuplot** command **set terminal** to select a printer and **set output** to redirect output to a file.

2. Select the **Print...** command from the **gnuplot graph** window. An extra command **screendump** does this from the text window.

3. If **set output "PRN"** is used, output will go to a temporary file. When you exit from **gnuplot** or when you change the output with another **set output** command, a dialog box will appear for you to select a printer port. If you choose OK, the output will be printed on the selected port, passing unmodified through the print manager. It is possible to accidentally (or deliberately) send printer output meant for one printer to an incompatible printer.

83.74.3 Text-menu

The **gnuplot text** window has the following options on a pop-up menu accessed by pressing the right mouse button or selecting **Options** from the system menu:

Copy to Clipboard copies marked text to the clipboard.

Paste copies text from the clipboard as if typed by the user.

Choose Font... selects the font used in the text window.

System Colors when selected makes the text window honor the System Colors set using the Control Panel. When unselected, text is black or blue on a white background.

Update wgnuplot.ini saves the current text window location, text window size, text window font and text window font size to the initialisation file **WGNUPLOT.INI**.

MENU BAR

If the menu file **WGNUPLOT.MNU** is found in the same directory as WGNUPLOT.EXE, then the menu specified in **WGNUPLOT.MNU** will be loaded. Menu commands:

[Menu] starts a new menu with the name on the following line.

[EndMenu] ends the current menu.

[–] inserts a horizontal menu separator.

[|] inserts a vertical menu separator.

[Button] puts the next macro on a push button instead of a menu.

Macros take two lines with the macro name (menu entry) on the first line and the macro on the second line. Leading spaces are ignored. Macro commands:

[INPUT] — Input string with prompt terminated by [EOS] or {ENTER}

[EOS] — End Of String terminator. Generates no output.

[OPEN] — Get name of file to open from list box, with title of list box terminated by [EOS], followed by default filename terminated by [EOS] or {ENTER}.

[SAVE] — Get name of file to save. Similar to [OPEN]

Macro character substitutions:

{ENTER} — Carriage Return '\r'

{TAB} — Tab '\011'

{ESC} — Escape '\033'

{^A} — '\001'

...

{^_} — '\031'

Macros are limited to 256 characters after expansion.

83.74.4 Wgnuplot.ini

Windows **gnuplot** will read some of its options from the **[WGNUPLOT]** section of **WGNUPLOT.INI** in user's %APPDATA% directory. A sample **WGNUPLOT.INI** file:

```
[WGNUPLOT]
TextOrigin=0 0
TextSize=640 150
TextFont=Terminal,9
GraphOrigin=0 150
GraphSize=640 330
GraphFont=Arial,10
GraphColor=1
GraphToTop=1
GraphBackground=255 255 255
Border=0 0 0 0 0
Axis=192 192 192 2 2
Line1=0 0 255 0 0
Line2=0 255 0 0 1
Line3=255 0 0 0 2
Line4=255 0 255 0 3
Line5=0 0 128 0 4
```

The **GraphFont** entry specifies the font name and size in points. The five numbers given in the **Border**, **Axis** and **Line** entries are the **Red** intensity (0–255), **Green** intensity, **Blue** intensity, **Color Linestyle** and **Mono Linestyle**. **Linestyles** are 0=SOLID, 1=DASH, 2=DOT, 3=DASHDOT, 4=DASHDOTDOT. In the sample **WGNUPLOT.INI** file above, Line 2 is a green solid line in color mode, or a dashed line in monochrome mode. The default line width is 1 pixel. If **Linestyle** is negative, it specifies the width of a SOLID line in pixels. Line1 and any linestyle used with the **points** style must be SOLID with unit width.

83.75 Wxt

The **wxt** terminal device generates output in a separate window. The window is created by the wxWidgets library, where the 'wxt' comes from. The actual drawing is done via cairo, a 2D graphics library, and pango, a library for laying out and rendering text.

Syntax:

```
set term wxt {<n>}
             {size <width>,<height>}
             {{no}enhanced}
             {font <font>}
             {title "title"}
             {{no}persist}
             {{no}raise}
```

```
                    {{no}ctrl}
                    {close}
```

Multiple plot windows are supported: **set terminal wxt <n>** directs the output to plot window number n.

The default window title is based on the window number. This title can also be specified with the keyword "title".

Plot windows remain open even when the **gnuplot** driver is changed to a different device. A plot window can be closed by pressing the letter 'q' while that window has input focus, by choosing **close** from a window manager menu, or with **set term wxt <n> close**.

The size of the plot area is given in pixels, it defaults to 640x384. In addition to that, the actual size of the window also includes the space reserved for the toolbar and the status bar. When you resize a window, the plot is immediately scaled to fit in the new size of the window. Unlike other interactive terminals, the **wxt** terminal scales the whole plot, including fonts and linewidths, and keeps its global aspect ratio constant, leaving an empty space painted in gray. If you type **replot**, click the **replot** icon in the terminal toolbar or type a new **plot** command, the new plot will completely fit in the window and the font size and the linewidths will be reset to their defaults.

The active plot window (the one selected by **set term wxt <n>**) is interactive. Its behaviour is shared with other terminal types. See **mouse (p. 109)** for details. It also has some extra icons, which are supposed to be self-explanatory.

This terminal supports an enhanced text mode, which allows font and other formatting commands (subscripts, superscripts, etc.) to be embedded in labels and other text strings. The enhanced text mode syntax is shared with other gnuplot terminal types. See **enhanced (p. 20)** for more details.

<font> is in the format "FontFace,FontSize", i.e. the face and the size comma-separated in a single string. FontFace is a usual font face name, such as 'Arial'. If you do not provide FontFace, the wxt terminal will use 'Sans'. FontSize is the font size, in points. If you do not provide it, the wxt terminal will use a size of 10 points.

```
 For example :
    set term wxt font "Arial,12"
    set term wxt font "Arial" # to change the font face only
    set term wxt font ",12" # to change the font size only
    set term wxt font "" # to reset the font name and size
```

The fonts are retrieved from the usual fonts subsystems. Under Windows, those fonts are to be found and configured in the entry "Fonts" of the control panel. Under UNIX, they are handled by "fontconfig".

Pango, the library used to layout the text, is based on utf-8. Thus, the wxt terminal has to convert from your encoding to utf-8. The default input encoding is based on your 'locale'. If you want to use another encoding, make sure gnuplot knows which one you are using. See **encoding (p. 96)** for more details.

Pango may give unexpected results with fonts that do not respect the unicode mapping. With the Symbol font, for example, the wxt terminal will use the map provided by http://www.unicode.org/ to translate character codes to unicode. Pango will do its best to find a font containing this character, looking for your Symbol font, or other fonts with a broad unicode coverage, like the DejaVu fonts. Note that "the Symbol font" is to be understood as the Adobe Symbol font, distributed with Acrobat Reader as "SY______.PFB". Alternatively, the OpenSymbol font, distributed with OpenOffice.org as "opens___.ttf", offers the same characters. Microsoft has distributed a Symbol font ("symbol.ttf"), but it has a different character set with several missing or moved mathematic characters. If you experience problems with your default setup (if the demo enhancedtext.dem is not displayed properly for example), you probably have to install one of the Adobe or OpenOffice Symbol fonts, and remove the Microsoft one. Other non-conform fonts, such as "wingdings" have been observed working.

The rendering of the plot can be altered with a dialog available from the toolbar. To obtain the best output possible, the rendering involves three mechanisms : antialiasing, oversampling and hinting. Antialiasing allows to display non-horizontal and non-vertical lines smoother. Oversampling combined with antialiasing provides subpixel accuracy, so that gnuplot can draw a line from non-integer coordinates. This avoids wobbling effects on diagonal lines ('plot x' for example). Hinting avoids the blur on horizontal and vertical lines caused by oversampling. The terminal will snap these lines to integer coordinates so that a one-pixel-wide line will actually be drawn on one and only one pixel.

By default, the window is raised to the top of your desktop when a plot is drawn. This can be controlled with the keyword "raise". The keyword "persist" will prevent gnuplot from exiting before you explicitely close all the plot windows. Finally, by default the key <space> raises the gnuplot console window, and 'q' closes the plot window. The keyword "ctrl" allows you to replace those bindings by <ctrl>+<space> and <ctrl>+'q',

respectively. These three keywords (raise, persist and ctrl) can also be set and remembered between sessions through the configuration dialog.

83.76 X11

gnuplot provides the **x11** terminal type for use with X servers. This terminal type is set automatically at startup if the **DISPLAY** environment variable is set, if the **TERM** environment variable is set to **xterm**, or if the **-display** command line option is used.

Syntax:

```
set terminal x11 {<n> | window "<string>"}
                 {title "<string>"}
                 {{no}enhanced} {font <fontspec>}
                 {linewidth LW} {solid|dashed}
                 {{no}persist} {{no}raise} {{no}ctrlq}
                 {close}
                 {size XX,YY} {position XX,YY}
set terminal x11 {reset}
```

Multiple plot windows are supported: **set terminal x11 <n>** directs the output to plot window number n. If n is not 0, the terminal number will be appended to the window title (unless a title has been supplied manually) and the icon will be labeled **Gnuplot <n>**. The active window may be distinguished by a change in cursor (from default to crosshair).

The **x11** terminal can connect to X windows previously created by an outside application via the option **window** followed by a string containing the X ID for the window in hexadecimal format. Gnuplot uses that external X window as a container since X does not allow for multiple clients selecting the ButtonPress event. In this way, gnuplot's mouse features work within the contained plot window.

```
set term x11 window "220001e"
```

The x11 terminal supports enhanced text mode (see **enhanced (p. 20)**), subject to the available fonts. In order for font size commands embedded in text to have any effect, the default x11 font must be scalable. Thus the first example below will work as expected, but the second will not.

```
set term x11 enhanced font "arial,15"
set title '{/=20 Big} Medium {/=5 Small}'

set term x11 enhanced font "terminal-14"
set title '{/=20 Big} Medium {/=5 Small}'
```

Plot windows remain open even when the **gnuplot** driver is changed to a different device. A plot window can be closed by pressing the letter q while that window has input focus, or by choosing **close** from a window manager menu. All plot windows can be closed by specifying **reset**, which actually terminates the subprocess which maintains the windows (unless **-persist** was specified). The **close** command can be used to close individual plot windows by number. However, after a **reset**, those plot windows left due to persist cannot be closed with the command **close**. A **close** without a number closes the current active plot window.

The gnuplot outboard driver, gnuplot_x11, is searched in a default place chosen when the program is compiled. You can override that by defining the environment variable GNUPLOT_DRIVER_DIR to point to a different location.

Plot windows will automatically be closed at the end of the session unless the **-persist** option was given.

The options **persist** and **raise** are unset by default, which means that the defaults (persist == no and raise == yes) or the command line options -persist / -raise or the Xresources are taken. If [no]persist or [no]raise are specified, they will override command line options and Xresources. Setting one of these options takes place immediately, so the behaviour of an already running driver can be modified. If the window does not get raised, see discussion in **raise (p. 80)**.

The option **title "<title name>"** will supply the title name of the window for the current plot window or plot window <n> if a number is given. Where (or if) this title is shown depends on your X window manager.

The size option can be used to set the size of the plot window. The size option will only apply to newly created windows.

The position option can be used to set the position of the plot window. The position option will only apply to newly created windows.

The size or aspect ratio of a plot may be changed by resizing the **gnuplot** window.

Linewidths and pointsizes may be changed from within **gnuplot** with **set linestyle**.

For terminal type **x11**, **gnuplot** accepts (when initialized) the standard X Toolkit options and resources such as geometry, font, and name from the command line arguments or a configuration file. See the X(1) man page (or its equivalent) for a description of such options.

A number of other **gnuplot** options are available for the **x11** terminal. These may be specified either as command-line options when **gnuplot** is invoked or as resources in the configuration file ".Xdefaults". They are set upon initialization and cannot be altered during a **gnuplot** session. (except **persist** and **raise**)

83.76.1 X11_fonts

Upon initial startup, the default font is taken from the X11 resources as set in the system or user .Xdefaults file or on the command line.

Example:

```
gnuplot*font: lucidasans-bold-12
```

A new default font may be specified to the x11 driver from inside gnuplot using

```
`set term x11 font "<fontspec>"`
```

The driver first queries the X-server for a font of the exact name given. If this query fails, then it tries to interpret <fontspec> as "<font>,<size>,<slant>,<weight>" and to construct a full X11 font name of the form

```
-*-<font>-<weight>-<s>-*-*-<size>-*-*-*-*-*-<encoding>

<font> is the base name of the font (e.g. Times or Symbol)
<size> is the point size (defaults to 12 if not specified)
<s> is `i` if <slant>=="italic" `o` if <slant>=="oblique" `r` otherwise
<weight> is `medium` or `bold` if explicitly requested, otherwise `*`
<encoding> is set based on the current character set (see `set encoding`).
```

So **set term x11 font "arial,15,italic"** will be translated to -*-arial-*-i-*-*-15-*-*-*-*-*-iso8859-1 (assuming default encoding). The <size>, <slant>, and <weight> specifications are all optional. If you do not specify <slant> or <weight> then you will get whatever font variant the font server offers first. You may set a default enconding via the corresponding X11 resource. E.g.

```
gnuplot*encoding: iso8859-15
```

The driver also recognizes some common PostScript font names and replaces them with possible X11 or TrueType equivalents. This same sequence is used to process font requests from **set label**.

If your gnuplot was built with configuration option –enable-x11-mbfonts, you can specify multi-byte fonts by using the prefix "mbfont:" on the font name. An additional font may be given, separated by a semicolon. Since multi-byte font encodings are interpreted according to the locale setting, you must make sure that the environmental variable LC_CTYPE is set to some appropriate locale value such as ja_JP.eucJP, ko_KR.EUC, or zh_CN.EUC.

Example:

```
set term x11 font 'mbfont:kana14;k14'
      # 'kana14' and 'k14' are Japanese X11 font aliases, and ';'
      # is the separator of font names.
set term x11 font 'mbfont:fixed,16,r,medium'
      # <font>,<size>,<slant>,<weight> form is also usable.
set title '(mb strings)' font 'mbfont:*-fixed-medium-r-normal--14-*'
```

The same syntax applies to the default font in Xresources settings, for example,

```
gnuplot*font: \
    mbfont:-misc-fixed-medium-r-normal--14-*-*-*-c-*-jisx0208.1983-0
```

If gnuplot is built with –enable-x11-mbfonts, you can use two special PostScript font names 'Ryumin-Light-*' and 'GothicBBB-Medium-*' (standard Japanese PS fonts) without the prefix "mbfont:".

83.76.2 Command-line_options

In addition to the X Toolkit options, the following options may be specified on the command line when starting **gnuplot** or as resources in your ".Xdefaults" file (note that **raise** and **persist** can be overridden later by **set term x11 [no]raise [no]persist)**:

'-mono'	forces monochrome rendering on color displays.
'-gray'	requests grayscale rendering on grayscale or color displays. (Grayscale displays receive monochrome rendering by default.)
'-clear'	requests that the window be cleared momentarily before a new plot is displayed.
'-tvtwm'	requests that geometry specifications for position of the window be made relative to the currently displayed portion of the virtual root.
'-raise'	raises plot window after each plot.
'-noraise'	does not raise plot window after each plot.
'-noevents'	does not process mouse and key events.
'-persist'	plot windows survive after main gnuplot program exits.

The options are shown above in their command-line syntax. When entered as resources in ".Xdefaults", they require a different syntax.

Example:

```
gnuplot*gray:  on
gnuplot*ctrlq: on
```

gnuplot also provides a command line option (**-pointsize <v>**) and a resource, **gnuplot*pointsize: <v>**, to control the size of points plotted with the **points** plotting style. The value **v** is a real number (greater than 0 and less than or equal to ten) used as a scaling factor for point sizes. For example, **-pointsize 2** uses points twice the default size, and **-pointsize 0.5** uses points half the normal size.

The **-noevents** switch disables all mouse and key event processing (except for **q** and **<space>** for closing the window). This is useful for programs which use the x11 driver independent of the gnuplot main program.

The **-ctrlq** switch changes the hot-key that closes a plot window from **q** to **<ctrl>q**. This is useful is you are using the keystroke-capture feature **pause mouse keystroke**, since it allows the character **q** to be captured just as all other alphanumeric characters. The **-ctrlq** switch similarly replaces the <space> hot-key with <ctrl><space> for the same reason.

83.76.3 Monochrome_options

For monochrome displays, **gnuplot** does not honor foreground or background colors. The default is black-on-white. **-rv** or **gnuplot*reverseVideo: on** requests white-on-black.

83.76.4 Color_resources

The X11 terminal honors the following resources (shown here with their default values) or the greyscale resources. The values may be color names as listed in the X11 rgb.txt file on your system, hexadecimal RGB color specifications (see X11 documentation), or a color name followed by a comma and an **intensity** value from 0 to 1. For example, **blue, 0.5** means a half intensity blue.

gnuplot*background: white
gnuplot*textColor: black
gnuplot*borderColor: black
gnuplot*axisColor: black
gnuplot*line1Color: red
gnuplot*line2Color: green
gnuplot*line3Color: blue
gnuplot*line4Color: magenta
gnuplot*line5Color: cyan
gnuplot*line6Color: sienna
gnuplot*line7Color: orange
gnuplot*line8Color: coral

The command-line syntax for these is simple only for background, which maps directly to the usual X11 toolkit option "-bg". All others can only be set on the command line by use of the generic "-xrm" resource override option

Examples:

```
gnuplot -background coral
```

to change the background color.

```
gnuplot -xrm 'gnuplot*line1Color:blue'
```

to override the first linetype color.

83.76.5 Grayscale_resources

When **-gray** is selected, **gnuplot** honors the following resources for grayscale or color displays (shown here with their default values). Note that the default background is black.

gnuplot*background: black
gnuplot*textGray: white
gnuplot*borderGray: gray50
gnuplot*axisGray: gray50
gnuplot*line1Gray: gray100
gnuplot*line2Gray: gray60
gnuplot*line3Gray: gray80
gnuplot*line4Gray: gray40
gnuplot*line5Gray: gray90
gnuplot*line6Gray: gray50
gnuplot*line7Gray: gray70
gnuplot*line8Gray: gray30

83.76.6 Line_resources

gnuplot honors the following resources for setting the width (in pixels) of plot lines (shown here with their default values.) 0 or 1 means a minimal width line of 1 pixel width. A value of 2 or 3 may improve the appearance of some plots.

gnuplot*borderWidth: 2
gnuplot*axisWidth: 0
gnuplot*line1Width: 0
gnuplot*line2Width: 0
gnuplot*line3Width: 0
gnuplot*line4Width: 0
gnuplot*line5Width: 0
gnuplot*line6Width: 0
gnuplot*line7Width: 0
gnuplot*line8Width: 0

gnuplot honors the following resources for setting the dash style used for plotting lines. 0 means a solid line. A two-digit number **jk** (**j** and **k** are >= 1 and <= 9) means a dashed line with a repeated pattern of **j** pixels on followed by **k** pixels off. For example, '16' is a dotted line with one pixel on followed by six pixels off. More elaborate on/off patterns can be specified with a four-digit value. For example, '4441' is four on, four off, four on, one off. The default values shown below are for monochrome displays or monochrome rendering on color or grayscale displays. Color displays default to dashed:off

```
gnuplot*dashed: off
gnuplot*borderDashes: 0
gnuplot*axisDashes: 16
gnuplot*line1Dashes: 0
gnuplot*line2Dashes: 42
gnuplot*line3Dashes: 13
gnuplot*line4Dashes: 44
gnuplot*line5Dashes: 15
gnuplot*line6Dashes: 4441
gnuplot*line7Dashes: 42
gnuplot*line8Dashes: 13
```

83.76.7 X11 pm3d_resources

Choosing the appropriate visual class and number of colors is a crucial point in X11 applications and a bit awkward, since X11 supports six visual types in different depths.

By default **gnuplot** uses the default visual of the screen. The number of colors which can be allocated depends on the visual class chosen. On a visual class with a depth > 12bit, gnuplot starts with a maximal number of 0x200 colors. On a visual class with a depth > 8bit (but <= 12 bit) the maximal number of colors is 0x100, on <= 8bit displays the maximum number of colors is 240 (16 are left for line colors).

Gnuplot first starts to allocate the maximal number of colors as stated above. If this fails, the number of colors is reduced by the factor 2 until gnuplot gets all colors which are requested. If dividing **maxcolors** by 2 repeatedly results in a number which is smaller than **mincolors gnuplot** tries to install a private colormap. In this case the window manager is responsible for swapping colormaps when the pointer is moved in and out the x11 driver's window.

The default for **mincolors** is maxcolors / (num_colormaps > 1 ? 2 : 8), where num_colormaps is the number of colormaps which are currently used by gnuplot (usually 1, if only one x11 window is open).

Some systems support multiple (different) visual classes together on one screen. On these systems it might be necessary to force gnuplot to use a specific visual class, e.g. the default visual might be 8bit PseudoColor but the screen would also support 24bit TrueColor which would be the preferred choice.

The information about an Xserver's capabilities can be obtained with the program **xdpyinfo**. For the visual names below you can choose one of StaticGray, GrayScale, StaticColor, PseudoColor, TrueColor, DirectColor. If an Xserver supports a requested visual type at different depths, **gnuplot** chooses the visual class with the highest depth (deepest). If the requested visual class matches the default visual and multiple classes of this type are supported, the default visual is preferred.

Example: on an 8bit PseudoColor visual you can force a private color map by specifying **gnuplot*maxcolors: 240** and **gnuplot*mincolors: 240**.

```
gnuplot*maxcolors: integer
gnuplot*mincolors: integer
gnuplot*visual: visual name
```

83.76.8 X11 other_resources

By default the contents of the current plot window are exported to the X11 clipboard in response to X events in the window. Setting the resource 'gnuplot*exportselection' to 'off' or 'false' will disable this.

By default text rotation is done using a method that is fast, but can corrupt nearby colors depending on the background. If this is a problem, you can set the resource 'gnuplot.fastrotate' to 'off'

```
gnuplot*exportselection: off
gnuplot*fastrotate: on
gnuplot*ctrlq: off
```

83.77 Xlib

The **xlib** terminal driver supports the X11 Windows System. It generates gnuplot_x11 commands, but sends them to the output file specified by **set output '<filename>'**. **set term x11** is equivalent to **set output "|gnuplot_x11 -noevents"; set term xlib.** **xlib** takes the same set of options as **x11**.

Part V

Graphical User Interfaces

Several graphical user interfaces have been written for **gnuplot** and one for win32 is included in this distribution.

Bruce Ravel (ravel@phys.washington.edu) has written a gnuplot-mode for GNU emacs and XEmacs based on the earlier gnuplot.el file by Gershon Elber. While the gnuplot CVS repository has its own copy the most recent version of this package is available from

`http://feff.phys.washington.edu/~ ravel/software/gnuplot-mode/`

For Python, Tk/Tcl, and other front-ends, check the links on

`http://gnuplot.sourceforge.net/links.html`

Part VI

Bugs

Bugs reported since the current release as well as older ones may be located via the official distribution site on SourceForge.

Please e-mail bug reports to the gnuplot-bugs mailing list. Or upload the report to the gnuplot web site on SourceForge. Please give complete information on the version of gnuplot you are using and, if possible, a test script that demonstrates the bug. See **Seeking-assistance (p. 15)**.

The sections below list problems known to be present in gnuplot version 4.4 at the time of release. Some of these are actually bugs in external support libraries and may have been fixed independent of any changes in gnuplot.

84 Gnuplot limitations

Floating point exceptions (floating point number too large/small, divide by zero, etc.) may occasionally be generated by user defined functions. Some of the demos in particular may cause numbers to exceed the floating point range. Whether the system ignores such exceptions (in which case **gnuplot** labels the corresponding point as undefined) or aborts **gnuplot** depends on the compiler/runtime environment.

The gamma, bessel, and erf functions do not work for complex arguments.

Only one color palette at a time is active for any given x11 plot window. This means that multiplots whose constituent plots use different palettes will not display correctly in x11.

Coordinates specified as "time" wrap at 24 hours, and have a precision limited to 1 second. This is in particular a limitation in using time format to handle geographic coordinates.

Error bars are not handled properly in polar/spherical coordinate plot modes.

The 'nohidden3d' option that is supposed to exempt individual plots from the global property 'set hidden3d' does not work for parametric curves.

85 External libraries

External library GD (used by PNG/JPEG/GIF drivers): Versions of libgd through 2.0.33 contain various bugs in mapping the characters of Adobe's Symbol font. Also it is possible to trigger a library segfault if an anti-aliased line crosses an upper corner of the canvas.

External library PDFlib (used by PDF driver): Gnuplot can be linked against libpdf versions 4, 5, or 6. However, these versions differ in their handling of piped I/O. Therefore gnuplot scripts using piped output to PDF may work only for some versions of PDFlib.

External library svgalib (used by linux and vgagl driver): Requires gnuplot to be suid root (bad!) and has many bugs that are specific to the video card or graphics driver used in X11.

Internationalization (locale settings): Gnuplot uses the C runtime library routine setlocale() to control locale-specific formatting of input and output number, times, and date strings. The locales available, and the level of support for locale features such as "thousands' grouping separator", depend on the internationalization support provided by your individual machine.

Part VII

Index

Index

.gnuplot, 22

abs, 12
acos, 12
acosh, 12
acsplines, 58
adobeglyphnames, 171
aed512, 139
aed767, 139
aifm, 139
all, 68
amiga, 140
angles, 71, 113
apollo, 140
Aqua, 140
aqua, 140
arg, 12
arrow, 72, 116
arrowstyle, 37, 115
ascii, 137
asin, 12
asinh, 12
atan, 12
atan2, 12
atanh, 12
automated, 33
autoscale, 64, 73
avs, 54
axes, 8, 17, 22, 51

backquotes, 23
bars, 28–30, 37–39, 63, 75
batch/interactive, 4, 7, 41, 49, 71
BE, 140
be, 140
besj0, 12
besj1, 12
bessel, 186
besy0, 12
besy1, 12
bezier, 58
binary, 52, 54, 83, 137
binary examples, 56
bind, 20, 70, 75, 99
bitwise operators, 14
bmargin, 75
border, 75, 90, 121
boxerrorbars, 26, 77
boxes, 27, 28, 77
boxwidth, 29, 76
boxxyerrorbars, 28
branch, 46
bugs, 186

call, 40, 49
candlesticks, 28, 30
canvas, 5, 154, 158, 166
cbdata, 132
cbdtics, 132
cblabel, 133
cbmtics, 133
cbrange, 18, 19, 106, 108, 118, 133
cbtics, 133
cd, 40
ceil, 12
center, 34, 55
cgi, 143
cgm, 143
circle, 6, 29, 103
circles, 6, 29
clabel, 77, 79, 80, 93
clear, 40
clip, 37, 77
cntrparam, 22, 78, 80, 114, 138
color resources, 183
colorbox, 19, 79, 106–108, 133
colornames, 18, 19, 80, 118
colors, 18, 67, 110, 118
colorspec, 18, 95, 102, 118, 119, 123
column, 13
columnheader, 9, 65, 92
command line editing, 7
command line options, 183
command-line-editing, 69
command-line-options, 21
commands, 39
comments, 4, 8, 49
commentschars, 8, 82
compatibility, 6
contour, 22, 39, 77, 79, 80, 90, 119, 138
coordinates, 8, 72, 94, 95, 102–104, 116, 121, 122, 125, 128
copyright, 3
corel, 146
cos, 12
cosh, 12
csplines, 58
cumulative, 6, 59

data, 35, 51, 81
data file, 51
datafile, 22, 42, 51, 73, 80, 90, 137
datastrings, 8, 35, 66
date specifiers, 88
debug, 146
decimalsign, 5, 83, 85, 87, 97
defined, 13
degrees, 71
depthorder, 39, 107, 108
dgrid3d, 6, 84, 114, 119, 138
division, 10
dospc, 146
dots, 29
dpu414, 151
dumb, 146
dummy, 74, 85

dx, 34, 55
dxf, 147
dxy800a, 147
dy, 34, 55

edf, 54
editing, 7
editing postscript, 169
eepic, 147
egalib, 156
egamono, 156
ehf, 54
ellipse, 6, 103
elliptic integrals, 13
emf, 148
emtex, 158
emxvesa, 148
emxvga, 148
encoding, 5, 9, 85, 97, 156, 165, 167, 180
encodings, 85
enhanced, 9, 140, 164, 165, 167, 168, 173, 177, 180, 181
environment, 10
eps, 17
epslatex, 148
epson 180dpi, 151
epson 60dpi, 151
epson lx800, 151
equal, 115
erf, 12
erfc, 12
error estimates, 43, 44
error state, 15, 70
errorbars, 51, 62
errorlines, 51, 63
errors, 15
evaluate, 41
every, 56
example, 57
examples, 61
excl, 151
exists, 13, 23
exit, 41
exp, 12
exponentiation, 14
expressions, 10, 68, 89

factorial, 13
failsafe, 34
fig, 152
file, 51
fill, 27–29, 31
filledcurves, 29
fillstyle, 5, 28, 29, 67, 102, 116, 119, 166
financebars, 28, 30
fit, 10, 15, 41, 43, 61, 139
fit parameters, 43
fitting, 43
flipx, 34
floating point exceptions, 81, 186
floor, 12
fontfile, 17, 169, 171
fontpath, 86, 169
fonts, 16, 17, 154, 158, 166, 182
format, 86, 119, 122, 125, 126, 129
format specifiers, 87
fortran, 81
fpe trap, 81
frequency, 58
fsteps, 30
function, 89
functions, 11, 16

gamma, 12, 186
gamma correction, 112
gd, 16
general, 52, 112, 135
ggi, 153
gif, 16, 153
glossary, 17, 56
gnugraph, 154
gpbin, 135
gpic, 155
gpr, 155
gprintf, 23, 87, 95
graph menu, 178
grass, 155
grayscale resources, 184
grid, 89
grid data, 80, 84, 135, 137
gui's, 186
guidelines, 45

hcgi, 143
heatmap, 33
help, 47
help desk, 4
hercules, 156
hidden3d, 39, 90, 92
histeps, 30
histograms, 31
history, 48, 69
historysize, 91
hotkey, 20
hotkeys, 20
hp2623a, 156
hp2648, 156
hp500c, 156
hpdj, 157
hpgl, 156
hpljii, 157
hppj, 157

ibeta, 12
if, 21, 48, 70
igamma, 12
imag, 12
image, 33, 36
imagen, 157
impulses, 34
increment, 118
index, 19, 52, 57
int, 12

internationalization, 186
interval, 118
introduction, 3
inverf, 12
invnorm, 12
isosamples, 22, 90, 91, 114, 137, 138
iteration, 32, 33, 48, 49, 65, 71, 138

jpeg, 16, 157

kdensity, 6, 59
key, 66, 92
kyo, 158

label, 35, 94, 99
labels, 9, 35, 52, 95, 99
lambertw, 12
latex, 158
lc, 18
least squares, 41
legend, 92
lgamma, 12
libgd, 186
license, 3
line, 18, 73, 80, 116
line editing, 7
line resources, 184
linecolor, 18
lines, 35, 36
linespoints, 36, 118
linestyle, 18, 35, 118
linetype, 18, 35, 67
linewidth, 35, 118
linux, 159
lmargin, 96
load, 49
loadpath, 96, 171
locale, 5, 83, 85, 97, 186
log, 12, 77
log10, 12
logscale, 97
lower, 49
lp, 36

macintosh, 159
macros, 16, 23, 41
map, 39, 107
mapping, 97, 113
margin, 75, 96, 98, 100, 114, 123
Marquardt, 41
matrix, 52, 53, 56, 136, 137
metafont, 159
metapost, 161
mf, 159
mgr, 160
mif, 160
missing, 81
mixing macros backquotes, 24
modulo, 14
monochrome options, 183
mouse, 20, 21, 98, 180
mousing, 98
mp, 161
multi branch, 46
multi-branch, 42, 46
multiplot, 41, 100, 175
mx2tics, 101
mxtics, 101, 102
my2tics, 102
mytics, 102
mztics, 102

NaN, 11, 16, 61
nec cp6, 151
negation, 13
negative, 110
new features, 5
newhistogram, 33
NeXT, 163
next, 163
noarrow, 72
noautoscale, 73
noborder, 75
nocbdtics, 132
nocbmtics, 133
nocbtics, 133
noclip, 77
nocontour, 80
nodgrid3d, 84
nofpe trap, 81
nogrid, 89
nohidden3d, 90, 186
nohistorysize, 91
nokey, 92
nolabel, 94
nologscale, 97
nomouse, 98
nomultiplot, 100
nomx2tics, 101
nomxtics, 101
nomy2tics, 102
nomytics, 102
nomztics, 102
nooffsets, 104
noparametric, 105
nopolar, 113
norm, 12
nosurface, 119
notimestamp, 121
nox2dtics, 124
nox2mtics, 124
nox2tics, 125
nox2zeroaxis, 125
noxdtics, 125
noxmtics, 126
noxtics, 128
noxzeroaxis, 130
noy2dtics, 131
noy2mtics, 131
noy2tics, 131
noy2zeroaxis, 131
noydtics, 131
noymtics, 131

noytics, 131
noyzeroaxis, 132
nozdtics, 132
nozmtics, 133
noztics, 133
nozzeroaxis, 132

object, 102
offsets, 104
okidata, 151
one's complement, 13
OpenStep, 163
Openstep, 163
openstep, 163
operator precedence, 13
operators, 13
options, 4
origin, 41, 100, 104
output, 104

palette, 18, 67, 80, 95, 106, 108, 109, 118, 119, 123, 133
parametric, 74, 105
pause, 49
pbm, 164
pcl5, 156
pdf, 16, 164, 186
pdfcairo, 164
pi, 16
placement, 92
plot, 50, 51, 69, 134
plotting, 22
pm, 165
pm3d, 39, 72, 80, 105, 108, 116, 119
pm3d resources, 185
png, 16, 120, 166
pngcairo, 167
pointinterval, 36, 118
points, 36
pointsize, 67, 113
polar, 22, 113
polygon, 6, 103
pop, 120
positive, 110
postscript, 17, 120, 167, 169
practical guidelines, 45
prescribe, 158
print, 68
printing, 178
prologue, 10, 149, 168, 170, 171
pseudocolumns, 19, 57, 61, 62
pslatex, 150, 171
pstex, 171
pstricks, 172
punctuation, 24
push, 120
pwd, 68

qms, 173
quit, 68
quotes, 3, 25

raise, 69, 181
rand, 12
random, 13
range frame, 130
rangelimited, 130
ranges, 41, 64
real, 12
rectangle, 102
refresh, 5, 51, 60, 69
regis, 173
replot, 60, 69
reread, 48, 70
reset, 70, 71
restore, 127
rgbalpha, 5, 33
rgbcolor, 18, 19
rgbformulae, 110
rgbimage, 33
rgip, 173
rmargin, 114
rotate, 34, 55
rrange, 114

samples, 58, 90, 92, 114, 119
save, 70
sbezier, 58
seeking assistance, 4
Seeking-assistance, 186
separator, 61, 82
set, 71
sgn, 12
shell, 133, 138
show, 71
sin, 12
sinh, 12
size, 41, 100, 105, 114, 154, 158, 166
skip, 56
smooth, 6, 58, 114
space, 20
special filenames, 59
special-filenames, 23
specifiers, 87
specify, 24
splot, 50, 69, 90, 113, 134
sprintf, 12, 23, 95
sqrt, 12
ssvgalib, 156
starc, 151
start, 22
start-up, 10
starting values, 46
startup, 22
statistical overview, 44
steps, 36
strcol, 13
strftime, 12
string operators, 14
stringcolumn, 13
strings, 22, 95
strlen, 12
strptime, 12
strstrt, 12

style, 61, 63, 66, 95
styles, 67, 115–117
substitution, 23, 25, 97
substr, 12
substring, 12, 23
sun, 173
surface, 80, 119, 138
svg, 173
svga, 174
svgalib, 156, 186
syntax, 3, 24, 87, 93, 123, 126
system, 12, 138

table, 119
tan, 12
tandy 60dpi, 151
tanh, 12
tc, 18
tek40, 174
tek410x, 174
term, 71, 139
terminal, 139
terminals, 120
termoption, 120
ternary, 14
test, 19, 67, 138
texdraw, 174
text, 25, 95, 165
text menu, 178
textcolor, 18
tgif, 174
thru, 60
tics, 120
ticscale, 121
ticslevel, 121
time specifiers, 88
Time/date, 122, 125
time/date, 25
timecolumn, 13
timefmt, 8, 64, 88, 95, 121, 125, 186
timestamp, 121
tips, 47
title, 9, 92, 122
tkcanvas, 175
tm hour, 13
tm mday, 13
tm min, 13
tm mon, 13
tm sec, 13
tm wday, 13
tm yday, 13
tm year, 13
tmargin, 123
tpic, 176
trange, 123
transparency, 34
transparent, 117

unary, 13
undefine, 138
uniplex, 173
unique, 58
unixpc, 176
unixplot, 176
unset, 138
update, 43, 139
urange, 123
user defined, 15
user-defined, 50
using, 9, 15, 26, 42, 60, 63, 106
UTF 8, 85, 171

valid, 13
variable, 27, 28, 35, 57
variables, 15, 16, 20, 21, 43, 50
vectors, 36
vgagl, 176
vgal, 148
vgalib, 156
vgamono, 156
view, 123, 130, 134
volatile, 5, 51
vrange, 124
vttek, 174
VWS, 177
vx384, 177

wgnuplot.ini, 179
windows, 177
with, 63, 66, 92, 113, 115, 152
word, 12
words, 12
writeback, 127
wxt, 16, 179

X resources, 182–185
X11, 181
x11, 4, 181
x11 fonts, 182
x11 mouse, 100
x2data, 124
x2dtics, 124
x2label, 124
x2mtics, 124
x2range, 124
x2tics, 125
x2zeroaxis, 125
xdata, 8, 95, 122, 124, 125, 130–132
xdtics, 124, 125, 131, 132
xerrorbars, 37
xerrorlines, 38
xfig, 152
xlabel, 124, 125, 131, 133
xlib, 185
xmtics, 124, 126, 131, 133
xrange, 74, 113, 114, 123, 124, 127, 131, 133
xterm, 174
xticlabels, 9, 62
xtics, 76, 87, 90, 101, 121, 125, 128, 131, 133
xyerrorbars, 37
xyerrorlines, 38
xyplane, 8, 121, 124, 130, 132, 134

xzeroaxis, 130

y2data, 130
y2dtics, 131
y2label, 131
y2mtics, 131
y2range, 131
y2tics, 131
y2zeroaxis, 131
ydata, 131
ydtics, 131
yerrorbars, 37
yerrorlines, 38
ylabel, 131
ymtics, 131
yrange, 131
ytics, 131
yzeroaxis, 132

zdata, 132
zdtics, 132
zero, 132
zeroaxis, 125, 130–132
zlabel, 133
zmtics, 133
zrange, 133
ztics, 133
zzeroaxis, 132
